# All-in-one disulfide bridging enables the generation of antibody conjugates with modular cargo loading†

**DOI:** 10.1039/d2sc02198f

**Published:** 2022-07-20

**Authors:** Friederike M. Dannheim, Stephen J. Walsh, Carolina T. Orozco, Anders Højgaard Hansen, Jonathan D. Bargh, Sophie E. Jackson, Nicholas J. Bond, Jeremy S. Parker, Jason S. Carroll, David R. Spring

**Affiliations:** Yusuf Hamied Department of Chemistry, University of Cambridge Cambridge CB2 1EW UK spring@ch.cam.ac.uk; Cancer Research UK Cambridge Institute, University of Cambridge Cambridge CB2 0RE UK Jason.carroll@cruk.cam.ac.uk; Department of Chemistry, Technical University of Denmark (DTU) 2800 Kgs. Lyngby Denmark; Analytical Sciences, Biopharmaceutical Development, R&D, AstraZeneca Granta Park Cambridge CB21 6GH UK; Early Chemical Development, Pharmaceutical Development, R&D, AstraZeneca Macclesfield SK10 2NA UK

## Abstract

Antibody–drug conjugates (ADCs) are valuable therapeutic entities which leverage the specificity of antibodies to selectively deliver cytotoxins to antigen-expressing targets such as cancer cells. However, current methods for their construction still suffer from a number of shortcomings. For instance, using a single modification technology to modulate the drug-to-antibody ratio (DAR) in integer increments while maintaining homogeneity and stability remains exceptionally challenging. Herein, we report a novel method for the generation of antibody conjugates with modular cargo loading from native antibodies. Our approach relies on a new class of disulfide rebridging linkers, which can react with eight cysteine residues, thereby effecting all-in-one bridging of all four interchain disulfides in an IgG1 antibody with a single linker molecule. Modification of the antibody with the linker in a 1 : 1 ratio enabled the modulation of cargo loading in a quick and selective manner through derivatization of the linker with varying numbers of payload attachment handles to allow for attachment of either 1, 2, 3 or 4 payloads (fluorescent dyes or cytotoxins). Assessment of the biological activity of these conjugates demonstrated their exceptional stability in human plasma and utility for cell-selective cytotoxin delivery or imaging/diagnostic applications.

## Abstract 2

Tetra-divinylpyrimidine (TetraDVP) linkers offer a method for the generation of antibody conjugates with modular cargo loading and excellent stability *via* all-in-one disulfide bridging.

## Graphical abstract

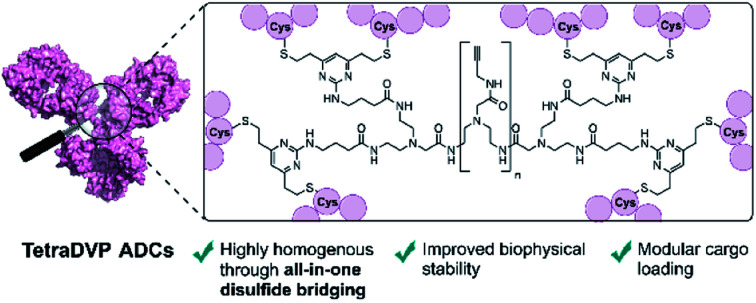

## Introduction

The selective delivery of drugs to malignant cells without undesired toxicity towards healthy tissues is one of the primary goals in the development of modern cancer therapeutics. Antibody–drug conjugates (ADCs) are a promising class of therapeutics which aims to address this need by harnessing the exquisite specificity of antibodies for cancer-associated antigens to selectively deliver highly cytotoxic payloads to malignant sites.^1–3^ The utility and timeliness of this therapeutic strategy has been showcased by the Food and Drug Administration (FDA) approval of eleven ADC drugs, eight of which were approved within the past five years.^4,5^

Despite the evident clinical success of ADCs, current methods for their construction still suffer from various shortcomings. All of the currently FDA-approved ADCs are synthesised from IgG1 or IgG4 antibodies *via* modification of the >50 surface exposed lysine residues or the eight cysteine residues which can be accessed through reduction of the antibody's four interchain disulfide bonds. Due to the high abundance of these reactive residues, defined modification of each antibody molecule is inherently challenging and results in highly heterogeneous ADCs with varying conjugation sites and wide distributions of drug loading (drug–antibody ratio, DAR) on each antibody.^6–9^ Conjugation site and DAR have repeatedly been shown to have a significant effect on both the safety and efficacy of ADCs, and heterogeneity in either of these aspects generally has a negative impact on ADC performance.^10–14^

While these shortcomings were tolerated in early generation ADCs, the current clinical and preclinical landscape contains an increased proportion of ADCs synthesised *via* site-selective modification methods.^6,15–17^ Extensive research has been conducted on the site-selective modification of antibodies *via* modification of antibody glycans, incorporation of unnatural amino acids with bioorthogonal functionality or fine-tuning of local microenvironments around certain natural amino acids.^18–27^ While these approaches indeed produce ADCs with well-defined attachment points and DAR, methods that can site-selectively modify native antibodies to generate homogeneous ADCs without the need for genetic/glycan engineering are desirable. Furthermore, even those methods capable of generating homogeneous ADCs are usually limited to producing ADCs with even DAR values (2, 4, 6, *etc.*) as any modification made on one of the heavy/light chains is always mirrored on the other heavy/light chain. While some groups have succeeded in the generation of homogenous ADCs with odd DAR values – such as White *et al.* who generated a DAR 1 ADC with a modified pyrrolobenzodiazepine (PBD) dimer payload containing two maleimide handles – the applied methods usually rely on engineered antibodies and bespoke payloads limiting their widespread applicability.^28^ Therefore, methods that enable access to the full scope of integer DAR values ≥ 1 in a simple and tuneable manner are of interest.

**Fig. 1:**
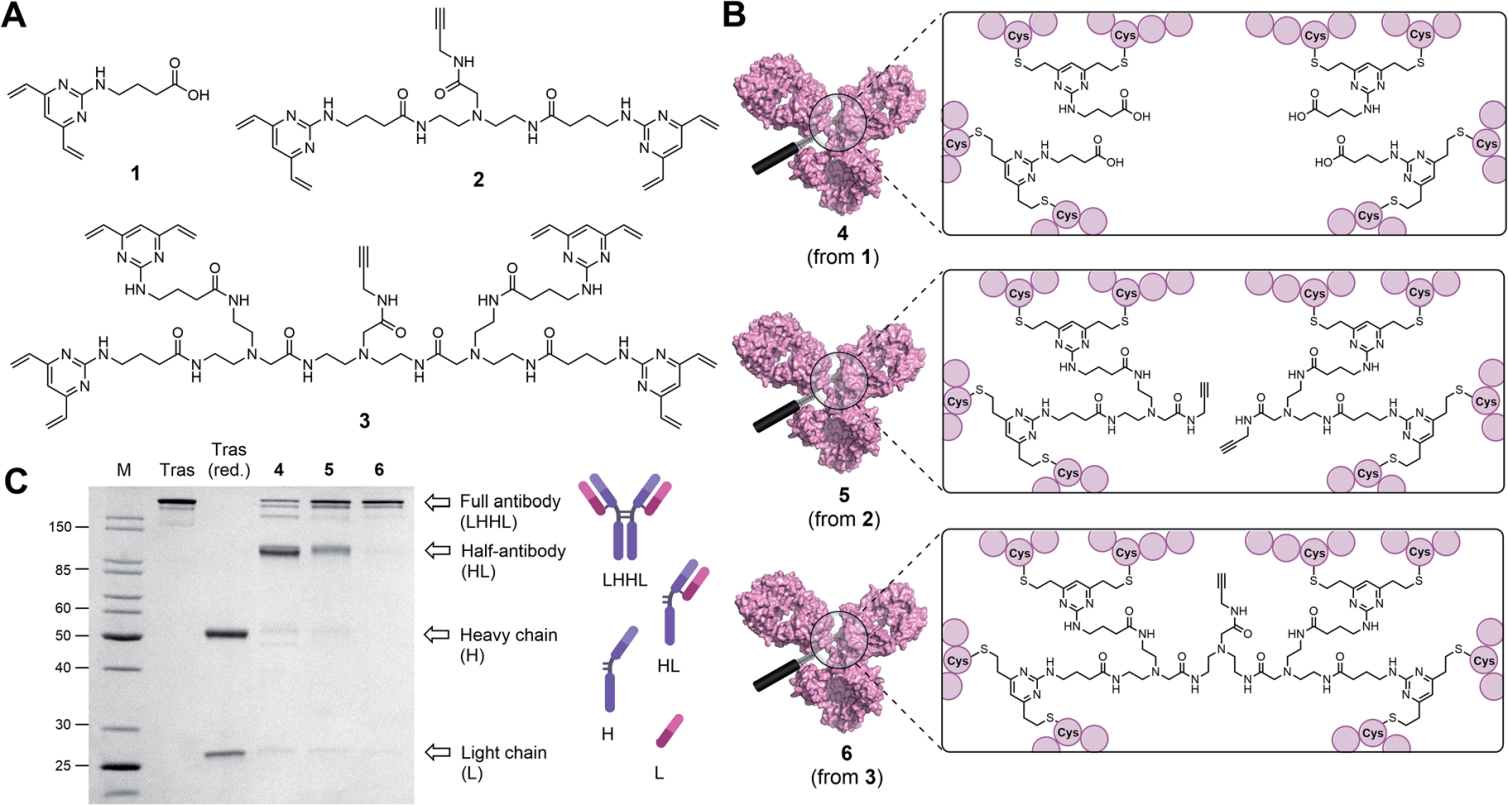
Antibody modifications with DVP (1), BisDVP (2) or TetraDVP (3) bridging reagents lead to conjugates with varying homogeneity. (A) Structures of bridging reagents. (B) Structures of antibody–linker-conjugates. (C) Homogeneity analysis of antibody–linker conjugates by SDS-PAGE; M = molecular weight marker, Tras = trastuzumab, Tras (red.) = reduced trastuzumab. The band at ∼150 kDa indicates fully rebridged antibody (LHHL); the band at ∼75 kDa indicates half-antibody species (HL) caused by non-native rebridging.

Disulfide bridging linkers have emerged as an attractive class of reagents with the potential to generate homogenous ADCs through the modification of native antibodies.^29–31^ These linkers contain two cysteine-reactive groups that may undergo reaction with reduced interchain disulfides in an IgG molecule to effect covalent rebridging of the antibody chains in a site-selective fashion. Next-generation maleimides (NGMs),^32,33^ pyridazinediones,^34^ bissulfones,^35^ divinylpyrimidine (DVP)^36–39^ and a variety of other reagents^40–44^ have been used to modify antibodies in this way. However, the utility of this approach is currently hampered by the formation of fragmented “half-antibody” species during bioconjugation, which is the result of non-native intrachain cross-linking of the cysteine residues in the hinge region of the antibody (Fig. 1C).^29,30^ The consequent loss of covalent linkages between the antibody heavy chains has been implicated with reduced antibody stability; therefore the development of methods which abrogate half-antibody formation is an important prerequisite for the widespread application of disulfide rebridging for ADC synthesis.^45^ In addition, rebridging reagents are also limited in the drug loading that can be achieved with most standard reagents obtaining DARs of either 2 or 4.

Herein, we report the development of a novel class of disulfide bridging linkers, which can react with eight cysteine residues, therefore effecting all-in-one bridging of all four interchain disulfides in an IgG1 antibody. Disulfide bridging with these tetra-divinylpyrimidine (TetraDVP) linkers completely abrogates half-antibody formation and simultaneously enables modulation of drug loading. As such, a toolbox of four different TetraDVP reagents was synthesised and used to generate antibody conjugates with cargo loadings of 1, 2, 3 and 4.

## Results and discussion

Classical disulfide rebridging reagents, including the DVP reagents developed in our group, typically react with two cysteine residues. As reduction of the interchain disulfides of a human IgG1 antibody yields eight reactive cysteine residues, most standard reagents therefore have a scope of DAR values which is limited to multiples of four. We hypothesised that increasing the number of cysteine reactive groups on each linker would allow us to decrease the linker-to-antibody ratio and thus expand the scope of DAR values attainable. Furthermore, we anticipated that this approach could reduce the risk of losing the covalent link between the two antibody heavy chains that is often observed with standard disulfide rebridging reagents, and thus improve the stability of the overall construct. To test this hypothesis, we chose to investigate the reactivity of three different linkers (1–3) with varying numbers of cysteine reactive DVP motifs (Fig. 1A). DVP linker 1 was designed to react with two cysteine residues and synthesised *via* the known procedure by Bargh *et al.*^46^ Based on structural data of IgG1 molecules for which crystal structures have been obtained,^47^ and guided by complementary work by Chudasama and co-workers,^48^ we estimated that the maximum distance between any two interchain disulfides is approximately 20 Å, and any linker intended to connect multiple disulfides should be designed with this distance requirement in mind. Thus, BisDVP 2 and TetraDVP 3 were designed to react with four and eight cysteine residues, respectively, and synthesised from 1 in a convergent manner (see ESI† for full synthetic details).

With the desired linkers in hand, the anti-HER2 IgG1 trastuzumab was chosen as a model antibody to evaluate the bioconjugation efficiency of the various DVP scaffolds. Trastuzumab is a constituent of the FDA-approved ADCs Kadcyla® and Enhertu® – both of which are approved for the treatment of HER2-positive breast cancer – thus making it a model system of acute clinical relevance. Accordingly, interchain disulfide bonds in trastuzumab were reduced with tris(2-carboxyethyl)phosphine hydrochloride (TCEP) for one hour, followed by addition of 1, 2 or 3 and incubation at 37 °C for four hours to yield antibody–linker conjugates 4, 5 and 6 (Fig. 1B). Subsequent analysis of the reactions by liquid chromatography-mass spectrometry (LC-MS) and sodium dodecyl sulfate-polyacrylamide gel electrophoresis (SDS-PAGE) showed that DVP conjugate 4 comprised four DVP linkers bound to each antibody and consisted primarily of half-antibody species with only a minor amount of the full antibody observed (Fig. 1C and S2†). This is consistent with prior observations with these types of linkers.^36,37^ In contrast, BisDVP conjugate 5 was comprised of two linker molecules bound to each antibody and contained a significantly larger amount of the desired full antibody species, although the presence of half-antibody was still noticeable (Fig. 1C and S3†). This suggests that reducing the linker-to-antibody ratio by increasing the number of cysteine reactive groups per linker molecule indeed increases the probability of reforming the covalent bond between the antibody heavy chains during rebridging. Finally, the reaction with TetraDVP 3 yielded >95% conversion to the full antibody with only trace amounts of half-antibody detectable (Fig. 1C and S4†). Analysis by LC-MS revealed TetraDVP conjugate 6 to have an antibody-to-linker ratio of one – suggesting that the TetraDVP linker is indeed capable of completely rebridging all four antibody chains through a single linker molecule. The only detectable side product of the reaction is a minor amount of an antibody species with a molecular weight of ∼126 kDa (visible as a faint band just below the main band for conjugate 6 in Fig. 1C), which likely corresponds to a conjugate in which one of the antibody light chains is not covalently linked to the rest of the antibody. Side products containing multiple linker molecules were not observed. Thus, to the best of our knowledge, this constitutes the first example of the controlled modification of a native antibody with a rebridging reagent in a 1 : 1 ratio.

Due to the exceptional homogeneity of conjugate 6, we decided that TetraDVP 3 was the most promising linker candidate to take forward for further studies. To explore the robustness of this all-in-one bridging approach, we repeated the bioconjugation reaction with TetraDVP 3 under a wide range of reaction conditions. It was found that the reaction proceeded efficiently at protein concentrations between 1–5 mg mL^−1^ and temperatures between 4–37 °C, although a slight decrease in reactivity was observed at lower temperatures (Fig. S5†). Furthermore, the reaction could be conducted with as little of 1.5 molar equivalents of linker and 2.5% of organic co-solvent and while maintaining excellent product homogeneity and >90% protein recovery after removal of unreacted small molecule reagents. These results demonstrate that the TetraDVP reaction is both robust and economic.

Since the four interchain disulfides utilised for rebridging are a highly conserved feature across all human IgG1 antibodies,^49^ we envisioned that the TetraDVP reaction should be easily applicable to other antibodies of this isotype. To test this hypothesis, we reacted TetraDVP 3 with the anti-CD30 IgG1 brentuximab, which is the constituent antibody of the FDA-approved ADC Adcetris®.^50^ Gratifyingly, the reaction proceeded with high efficiency and homogeneity comparable to that achieved with trastuzumab without the need for any further optimisation of reaction conditions (Fig. S6 and S8†). This indicates that the TetraDVP approach may indeed be a general method which can be applied to different IgG1 antibodies with little to no need for case-by-case optimisation.

**Fig. 2:**
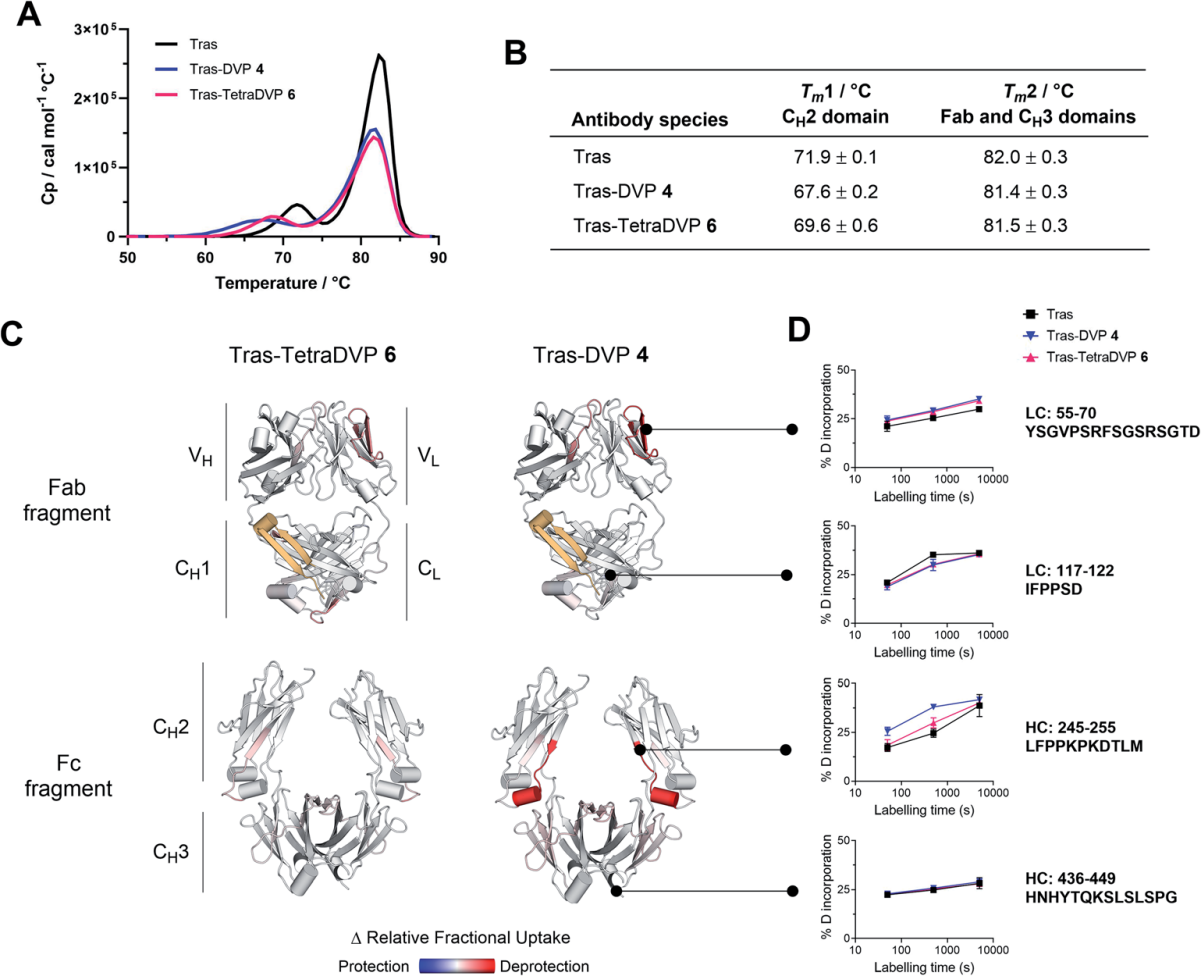
Biophysical stability analysis of trastuzumab (Tras), trastuzumab–DVP conjugate 4 and trastuzumab–TetraDVP conjugate 6. (A) Differential scanning calorimetry results of unmodified, DVP– and TetraDVP–linked trastuzumab. (B) Average of the melting temperatures of the antibody variants for duplicate measurements for Tras–DVP and Tras–TetraDVP, triplicates for Tras. The errors are the standard deviations of the repeats. (C) Crystal structures (Fab: 1N8Z; Fc: 3AVE) coloured according to the relative change in fractional deuterium exchange for Tras–TetraDVP and Tras–DVP compared to Tras; the differences plotted are significant differences assessed by a *t*-test, with *p*-value < 0.01. The yellow region represents the residues that were missing in the peptide map. Reduced exchange is shown in blue, no change in white, and increased exchange in red. (D) Details of the uptake plots for selected regions; the error bars represent a Student's *t* distribution with 95% confidence interval based on triplicates.

In light of these encouraging results, we sought to investigate the effects of TetraDVP conjugation on the biophysical stability of the parent antibody. A recent report by Bahou *et al.* suggests that conjugates with a low half-antibody content may possess increased biophysical stability compared to conjugates containing a high percentage of half-antibody species.^45^ Therefore, we were interested to explore if our TetraDVP conjugate possessed superior stability over conjugates made *via* rebridging of only two cysteine residues per linker. For this investigation, the stabilities of trastuzumab, DVP conjugate 4 and TetraDVP conjugate 6 were compared using differential scanning calorimetry (DSC), chemical denaturation and hydrogen-deuterium exchange mass spectrometry (HDX-MS). Initially, the thermal stability of the three antibody species was assessed by DSC. Each of the antibody species displayed two unfolding transitions, the first corresponding to the unfolding of the C_H_2 domain and the second corresponding to the unfolding of the C_H_3 domain and the Fab region (Fig. 2A).^51,52^ The melting temperatures of the two transitions show that both DVP and TetraDVP conjugation are associated with mild destabilisation of the C_H_2 domain, as evidenced by a decrease in *T*_m_1 (Fig. 2B). This decrease in melting temperature was seen to be less pronounced for TetraDVP conjugate 6 compared to DVP conjugate 4, indicating that the prevention of half antibody formation does indeed lead to an increase in biophysical stability. The appearance of a small shoulder on the peak corresponding to the second transition for both conjugates also indicates a possible minor destabilisation of the constant domains of the Fab region. This observation is supported by thermodynamic and kinetic stability data obtained from chemical denaturation experiments carried out on F(ab′)_2_ fragments obtained by enzymatic digestion of trastuzumab or conjugate 6 (Fig. S12, S13 and Table S1†). The observed destabilisation is likely caused by strain imposed on the cysteine residues present in the constant domains of the Fab; however, as the overall differences in stability between the unmodified antibody and the conjugates are minor, they are unlikely to have a pronounced effect on the pharmacological behaviour of the antibody. To verify that the DVP and TetraDVP modifications do not negatively affect antigen binding, the HER2 binding affinity of trastuzumab, DVP conjugate 4 and TetraDVP conjugate 6 was measured by biolayer interferometry (BLI). All antibody species were shown to have similarly high binding affinity for HER2 (Fig. S14†), showcasing that the conjugation does not disrupt the targeting capabilities of trastuzumab to a significant extent.

To further investigate the impact of conjugation on antibody structure, trastuzumab, DVP conjugate 4 and TetraDVP conjugate 6 were analysed by HDX-MS. HDX-MS is a technique which probes the molecular dynamics of the native state of proteins, thus enabling the detection and localisation of structural differences between modified and unmodified antibodies. Peptide mapping of trastuzumab was achieved with high coverage (light chain: 98.6%, heavy chain: 90.9%) using online pepsin digestion (Fig. S15†). Unfortunately, a portion of the C_H_1 domain (residues 201 to 237) evaded peptide mapping, and thus no conclusions can be drawn regarding the stability of this region. The analysed data of the remaining regions showed the largest increase in deuterium uptake for DVP conjugate 4 to be located in the C_H_2 domain, more specifically in the first β-strand and the short α-helix in the loop following the hinge region, as well as in a β-strand at the heart of the C_H_2 domain (Fig. 2C, D and S16†). Relative to DVP conjugate 4, TetraDVP-modified trastuzumab (6) appeared to exhibit no destabilisation of the β-strand at the beginning of the C_H_2 domain, suggesting that the two linkers impose different strains on this domain. These data corroborate the findings of the DSC analysis and indicate that TetraDVP conjugates may have increased stability compared to conjugates made *via* rebridging of two cysteine residues. Overall, it should be noted that the modest destabilising effects caused by both DVP and TetraDVP conjugation compare favourably to those caused by other clinically successful site-selective antibody modification approaches or mutations in the C_H_2 domain,^53–55^ showcasing the merits of this form of cysteine modification.

**Fig. 3:**
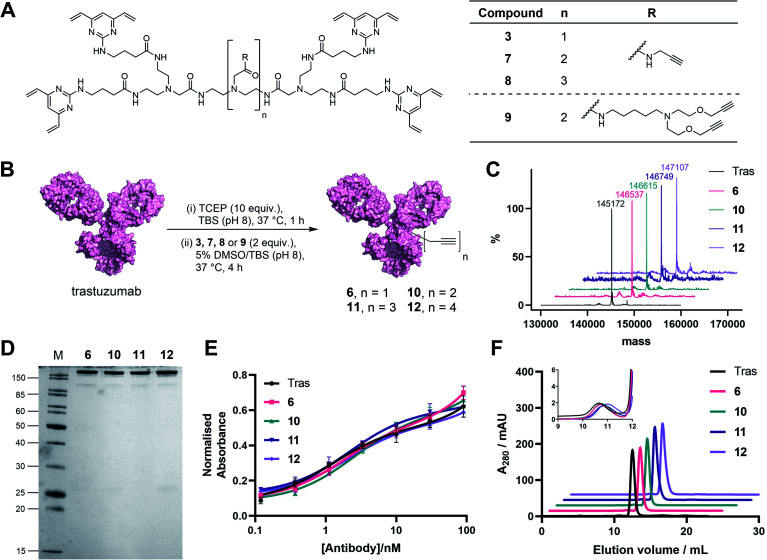
Generation and analysis of TetraDVP–trastuzumab conjugates. (A) Structures of TetraDVP linkers 3, 7, 8 and 9 containing one, two, three or four alkyne moieties, respectively. (B) Reaction of trastuzumab with TetraDVPs 3, 7, 8 and 9. TBS = tris-buffered saline. (C) LC-MS analysis of trastuzumab and conjugates 6, 10, 11 and 12. Samples were deglycosylated with PNGase F prior to analysis. (D) Analysis of conjugates 6, 10, 11 and 12 by SDS-PAGE under reducing conditions; M = molecular weight marker. (E) Binding affinity comparison of trastuzumab, 6, 10, 11 and 12 by ELISA. Error bars represent the standard deviation of biological quadruplicates. (F) Size-exclusion chromatography (SEC) analysis of trastuzumab and TetraDVP conjugates.

In light of these encouraging results, we aimed to increase the utility of TetraDVPs by creating a panel of TetraDVP reagents with varying numbers of alkyne groups to enable the generation of ADCs with modular drug-to-antibody ratios (DAR). Accordingly, we designed linkers 7, 8 and 9 which contain two, three or four alkyne handles, respectively, and should thus enable the controlled attachment of two, three or four azide-containing payloads *via* copper-catalysed azide–alkyne cycloaddition (CuAAC) (Fig. 3A). The linkers were synthesised *via* a universal route analogous to the synthesis of TetraDVP 3, and subsequently reacted with reduced trastuzumab for four hours at 37 °C (Fig. 3B; see ESI† for full synthetic details). Analysis of these reactions by SDS-PAGE and LC-MS revealed excellent conversion to conjugates 10, 11 and 12 (Fig. 3C). Despite slight variations in linker length, all three linkers displayed comparable reactivity to 3 and generated conjugates with outstanding homogeneity (Fig. 3D and S9–S11†), thus showcasing that the all-in-one bridging approach is not restricted to a single linker scaffold and supporting the viability of TetraDVPs for the construction of ADCs with modular DAR.

Prior to attempting functionalisation of the TetraDVP conjugates with azide-containing payloads, we sought to investigate the conjugates' aggregation behaviour and antigen binding ability. Modification of biomolecules with hydrophobic entities such as ADC linkers often induces protein aggregation and can inhibit antigen binding if the modification is located in close proximity to the antigen-binding sites.^13^ To investigate if TetraDVP reagents increase protein aggregation levels, conjugates 6, 10, 11 and 12 were analysed by size-exclusion chromatography (SEC). Gratifyingly, all conjugates displayed comparable aggregation levels to unmodified trastuzumab (<1.5%) (Fig. 3F and S17†), indicating that TetraDVPs do not induce antibody aggregation. Next, to investigate the modified antibodies' ability to recognise the target antigen HER2, we measured the HER2-binding affinity of trastuzumab, 6, 10, 11 and 12*via* enzyme-linked immunosorbent assays (ELISA). All antibody–linker conjugates retained nearly identical affinity for the HER2 receptor compared to unmodified trastuzumab (Fig. 3E). This data – alongside the BLI data obtained for conjugate 6 – suggests that the antibody's antigen recognition ability is not negatively impacted by the TetraDVP modifications.

**Fig. 4:**
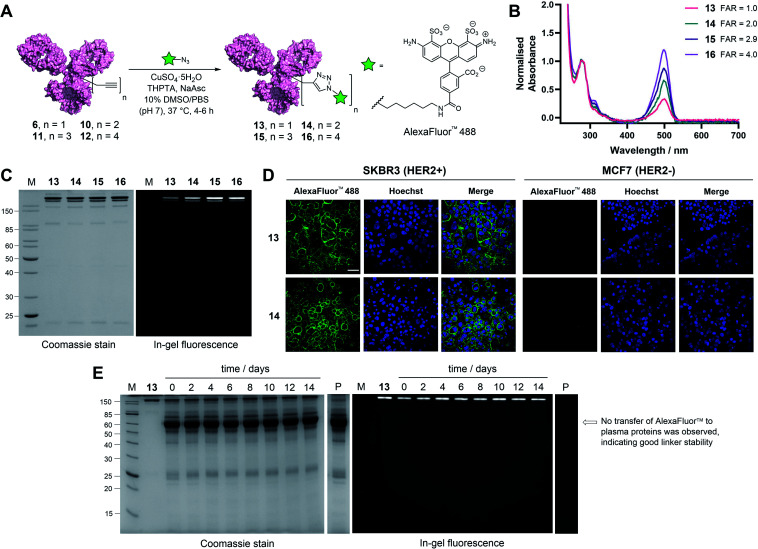
Functional modification and stability analysis of TetraDVP-modified trastuzumab. (A) Reaction of TetraDVP conjugates 6, 10, 11 and 12 with AlexaFluor™ 488 azide to form fluorescent conjugates 13, 14, 15 and 16. PBS = phosphate-buffered saline. (B) UV/vis spectra of conjugates 13, 14, 15 and 16. Absorbance was normalised at 280 nm. Absorbance at 495 nm was used to calculate the fluorophore-to-antibody ratio (FAR). (C) Analysis of conjugates 13, 14, 15 and 16 by SDS-PAGE under reducing conditions; M = molecular weight marker. Left gel is after Coomassie staining, right gel is in-gel fluorescence measured before staining. (D) Live cell microscopy images of HER2-positive SKBR3 cells and HER2-negative MCF7 cells after treatment with 13 or 14 shows selective labelling of antigen-positive cells. Scale bar represents 50 μm. (E) Stability analysis of conjugate 13 in human plasma by SDS-PAGE; M = molecular weight marker, P = human plasma, days of incubation are depicted above the representative lane. Left gel is after Coomassie staining, right gel is in-gel fluorescence measured before staining. No transfer of AlexaFluor™ 488 to human serum albumin (66.5 kDa, indicated by the arrow) or any other plasma proteins is observed over the 14 day incubation period.

With these promising results in hand, we sought to investigate the application of TetraDVP conjugates as biological imaging and diagnostic agents. Thus, conjugates 6, 10, 11 and 12 were reacted with AlexaFluor™ 488 azide in the presence of CuSO_4_·5H_2_O, tris(benzyltriazolylmethyl)amine (THPTA) and sodium ascorbate for 4–6 hours to generate antibody–fluorophore conjugates (AFCs) 13, 14, 15 and 16 (Fig. 4A). UV-vis analysis confirmed excellent conversion to the desired products with measured fluorophore-to-antibody ratios (FAR) of 1.0, 2.0, 2.9 or 4.0 correlating with the increasing number of alkyne moieties in the linker (Fig. 4B), thus validating all-in-one bridging as a high precision approach for the generation of functional antibody conjugates with modular cargo loading. Furthermore, analysis of the conjugates by SDS-PAGE with in-gel fluorescence showed a visible difference in fluorescence between the conjugates depending on FAR, which highlights the utility of this approach for imaging applications where precise tuning of fluorescence intensity is required (Fig. 4C).

To further explore the applicability of TetraDVP conjugates as imaging agents, HER2-positive SKBR3 cells and HER2-negative MCF7 cells were treated with fluorescent conjugates 13 or 14 for one hour at 4 °C. At the end of the incubation period, the cells were washed to remove any unbound antibody species, followed by incubation in replenished growth media for a further 3.5 hours. Live cell confocal microscopy imaging of the cells showed that both conjugates efficiently labelled and underwent internalisation by HER2-positive but not HER2-negative cells (Fig. 4D and S18†). These results provide further evidence that the TetraDVP modification does not negatively affect antigen binding or internalisation and showcases the potential of TetraDVP conjugates for the imaging of live cells in an antigen-dependent manner.

Having established that TetraDVP reagents can generate highly homogenous and functional bioconjugates, we next sought to evaluate the stability of the TetraDVP linkage under physiological conditions. Maleimides are the most commonly employed bioconjugation reagents in approved and clinical-stage ADCs. However, ADCs synthesised using these reagents suffer from instability in circulation and have been shown to transfer their payloads to plasma proteins after administration.^56,57^ This instability can be detrimental for drug development, as cytotoxic molecules which are released from the antibody prematurely have the potential to non-selectively harm healthy tissues and cause serious adverse effects in patients. To investigate if our TetraDVP conjugates exhibit any such stability issues, AFC 13 was incubated in human plasma at 37 °C for 14 days. This time frame was chosen as it resembles the half-life of a human IgG1 antibody in circulation.^58,59^ Throughout the incubation period, aliquots were taken every two days and analysed by SDS-PAGE with in-gel fluorescence detection. Pleasingly, we observed no transfer of the fluorescent payload to plasma proteins over the entire duration of the study (Fig. 4E). Repeating the experiment with conjugates 14, 15 and 16 yielded similar results (Fig. S19†). We can thus postulate that the TetraDVP modification is stable under physiological conditions and suitable for the construction of ADCs for clinical applications.

**Fig. 5:**
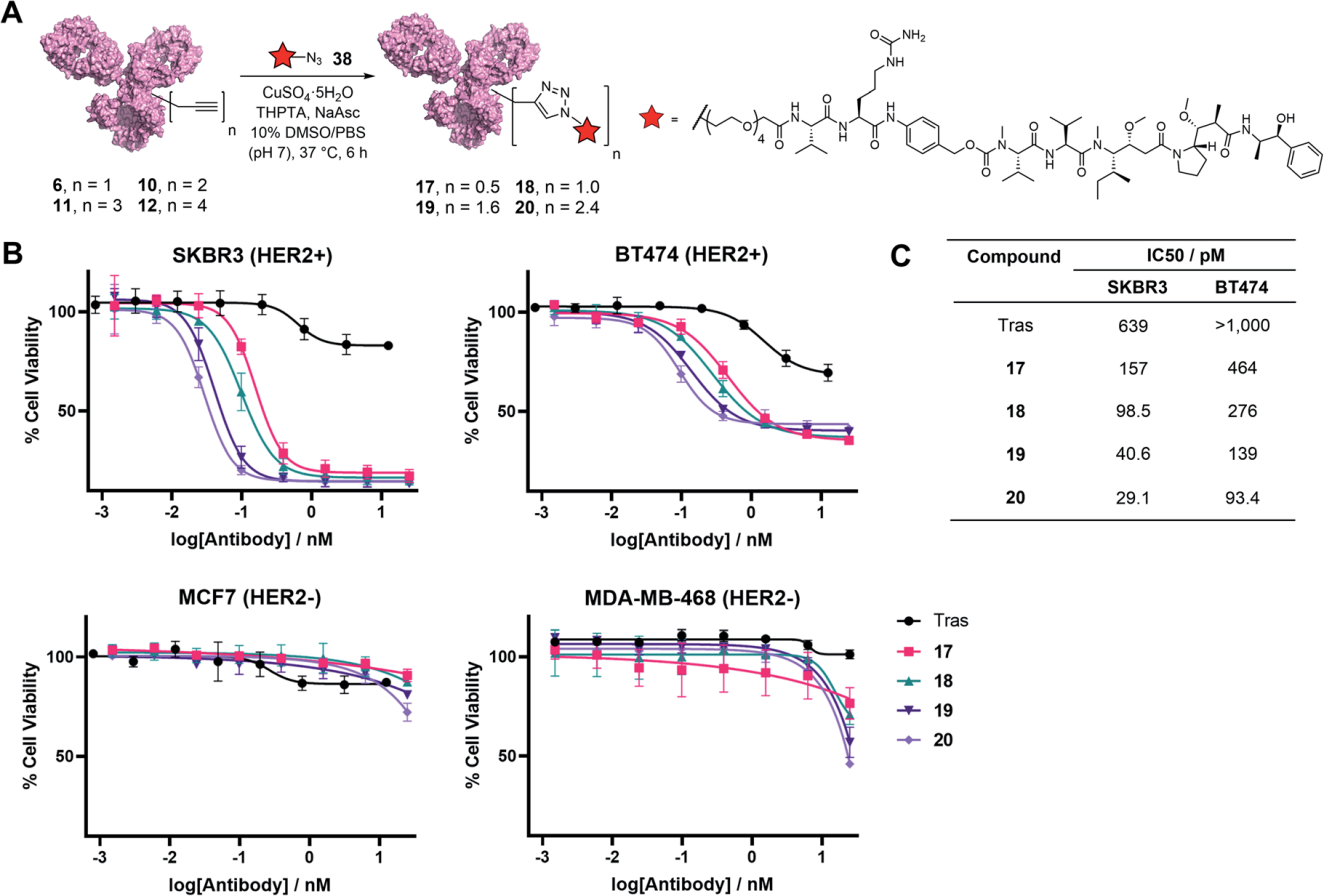
(A) Reaction of TetraDVP conjugates 6, 10, 11 and 12 with azide-functionalised MMAE 38 to form ADCs 17, 18, 19 and 20. PBS = phosphate-buffered saline. (B) Cytotoxicity of TetraDVP ADCs in HER2-positive (SKBR3 and BT474) and HER2-negative (MCF7 and MDA-MB-468) cell lines. Viability data shows the mean of three independent experiments and error bars represent the standard error of the mean (SEM). (C) Calculated IC_50_ values for TetraDVP ADCs in SKBR3 and BT474 cell lines.

Finally, to determine the capabilities of the TetraDVP methodology to generate bioactive ADCs, functionalisation of antibody–linker conjugates 6, 10, 11 and 12 with a cytotoxic warhead was undertaken. For this purpose, we chose to utilise monomethyl auristatin E (MMAE) as a payload. MMAE is a highly potent antimitotic agent that has found widespread utility in ADC development and constitutes the cytotoxic component of the FDA-approved ADCs Adcetris®, Polivy® and Padcev®. To enable the attachment of MMAE to TetraDVP conjugates, an azide-functionalised MMAE payload containing a cathepsin-cleavable valine–citrulline motif (38) was designed and synthesised from unfunctionalised MMAE in 2 steps (see ESI† for full synthetic details). The incorporation of a cleavable spacer was deemed important, as it allows for the traceless release of the MMAE payload from the antibody and the TetraDVP linker following internalisation by the target cell. Accordingly, payload 38 was reacted with conjugates 6, 10, 11 and 12 in the presence of CuSO_4_·5H_2_O, THPTA and sodium ascorbate for 6 hours to yield ADCs 17, 18, 19 and 20, as determined by hydrophobic interaction chromatography (HIC) (Fig. 5A and S20–S23†). While the corresponding CuAAC with AlexaFluor™ 488 azide proceeded with >95% conversion, the reaction of 6, 10, 11 and 12 with 38 displayed moderately diminished conversion to the desired ADCs. Increasing reaction time, concentration or reagent stoichiometry did not significantly affect conversion. We hypothesise that the increased steric bulk of the Val-Cit-PABC-MMAE payload compared to AlexaFluor™ 488 may impede efficient reactivity with the alkyne groups on the antibody. This limitation could possibly be remedied by adjusting the linker length and/or flexibility of the current TetraDVP design, thus indicating an area for future optimisation. Analytical SEC analysis showed approximately 95% monomer content for all ADCs (Fig. S24†), which is comparable to other ADCs containing MMAE. These data support the viability of TetraDVP ADCs for therapeutic applications.

Finally, evaluation of the *in vitro* cytotoxicity of ADC 17, 18, 19 and 20 was undertaken against a panel of HER2-positive (SKBR3 and BT474) and HER2-negative (MCF7 and MDA-MB-468) cell lines. All ADCs displayed a significant increase in concentration-dependent cytotoxicity in HER2-positive cells compared to trastuzumab alone (Fig. 5B). In contrast, the proliferation of HER2-negative cells was not significantly affected compared to vehicle control, thus confirming the selectivity for HER2-positive cells. Notably, the antiproliferative effect of the ADCs on HER2-positive cells correlated with DAR – higher DAR ADCs displayed an increase in cytotoxicity relative to lower DAR ADCs (Fig. 5C). This demonstrates that increased steric bulk with increasing DAR does not impede payload release and provides further evidence of the value of DAR tunability offered by this method.

## Conclusions

In conclusion, we have developed a novel bioconjugation platform for the generation of antibody conjugates from native antibodies. This first-in-class technology enables the functional bridging of all four disulfides in an IgG1 molecule, allowing for the modification of antibodies with linkers in a 1 : 1 ratio without the need for antibody/glycan engineering while also preventing the formation of half-antibody species detrimental to antibody stability. Moreover, small modifications of the linker scaffold allowed for unprecedented modulation of cargo loading. We have demonstrated that the modified antibodies possess improved biophysical stability over analogous conjugates made *via* standard disulfide rebridging and were fully stable in human plasma. Finally, we assessed the bioactivity of the ADCs in HER2-positive and HER2-negative cell lines, demonstrating good potency and excellent selectivity for HER2-positive cells. We envision that this all-in-one disulfide bridging technology should be applicable to any human IgG1 antibody as the four disulfides used for rebridging are highly conserved across this isotype.^49^ While the efficiency of the final payload attachment step requires further optimisation, we believe this technology to carry great potential as a conjugation method. The ability to easily investigate different antibody-payload-DAR combinations on the same antibody scaffold without the need for protein engineering offers to lower the cost and resource requirements for future ADC development and optimisation.

## Data availability

All data supporting this study are included in the paper and provided as ESI† accompanying this paper at the journal's website.

## Author contributions

Small molecule synthesis was performed by FMD, SJW, AHH and JDB. All other experiments were performed by FMD, SJW and CTO. The initial draft of the paper was written by FMD. DRS, JSC, JSP, NJB and SEJ supervised this project. All authors contributed to data interpretation and manuscript writing.

## Conflicts of interest

FMD, SJW and DRS are inventors on a patent application relating to the use of TetraDVP linkers for ADC synthesis.

## Supplementary Material

SC-013-D2SC02198F-s001
