# Supplementary material for: All-in-one disulfide bridging enables the generation of antibody conjugates with modular cargo loading
Source: Chem Sci. 2022 Jul 20;13(30):8781–90. doi: 10.1039/d2sc02198f (PMC9350601; doi:10.1039/d2sc02198f)
Supplement: SC-013-D2SC02198F-s001 [file SC-013-D2SC02198F-s001.pdf]

Electronic Supplementary Material (ESI) for Chemical Science.
This journal is © The Royal Society of Chemistry 2022

All-In-One Disulfide Bridging enables the Generation of Antibody Conjugates with Modular Cargo Loading

Friederike M. Dannheim[1], Stephen J. Walsh[1,2], Carolina T. Orozco[1], Anders Højgaard Hansen[1,3], Jonathan D. Bargh[1], Sophie E. Jackson[1], Nicholas J. Bond[4], Jeremy S. Parker[5], Jason S. Carroll[2]*, David R. Spring[1]*

[1]Department of Chemistry, University of Cambridge, Cambridge, UK [2]Cancer Research UK Cambridge Institute, Cambridge, UK [3]Department of Chemistry, Technical University of Denmark (DTU), Kongens Lyngby, Denmark [4]Analytical Sciences, Biopharmaceutical Development, R&D, AstraZeneca, Cambridge, UK [5]Early Chemical Development, Pharmaceutical Development, R&D, AstraZeneca, Macclesfield, UK

Supplementary Information

Table of contents

General Experimental Details 3
Chemical Synthesis 5
Bioconjugation 22
Thermal stability 32
Chemical denaturation experiments 32
  Generation of $F(ab')_2$ and Fc fragments from full length IgG 32
  Thermodynamic stability 32
  Kinetic stability 35
Biolayer interferometry (BLI) 38
Hydrogen-deuterium exchange mass spectrometry (HDX-MS) 40
Size-Exclusion Chromatography (SEC) of TetraDVP conjugates 45
Enzyme-linked immunosorbent assay (ELISA) 46
Preparation of antibody-fluorophore conjugates (AFCs) 46
Live cell microscopy 47
Stability Analysis 48
Preparation of antibody-drug conjugates (ADCs) 49
Hydrophobic interaction chromatography (HIC) 49
Size-Exclusion Chromatography (SEC) of ADCs 52
In vitro cytotoxicity 53
  Cell lines 53
  Cell viability 53
NMR Spectra 54
HPLC traces 76
HDX uptake plots 77
  Light chain 77
  Heavy chain 84
References 98

General Experimental Details

All solvents and reagents were used as received unless otherwise stated. Ethyl acetate, methanol, dichloromethane, acetonitrile and toluene were distilled from calcium hydride. Diethyl ether was distilled from a mixture of lithium aluminium hydride and calcium hydride. Petroleum ether (PE) refers to the fraction between 40 – 60 °C upon distillation. Tetrahydrofuran was dried using Na wire and distilled from a mixture of lithium aluminium hydride and calcium hydride with triphenylmethane as indicator.

Non-aqueous reactions were conducted under a stream of dry nitrogen using oven-dried glassware. Temperatures of 0 °C were maintained using an ice-water bath. Room temperature (rt) refers to ambient temperature.

Yields refer to spectroscopically and chromatographically pure compounds unless otherwise stated. Reactions were monitored by thin layer chromatography (TLC) or liquid chromatography mass spectroscopy (LC-MS). TLC was performed using glass plates pre-coated with Merck silica gel 60 F_{254} and visualized by quenching of UV fluorescence (λ_{max} = 254 nm) or by staining with potassium permanganate. Retention factors (R_f) are quoted to 0.01. LC-MS was carried out using a Waters ACQUITY H-Class UPLC with an ESCi Multi-Mode Ionisation Waters SQ Detector 2 spectrometer using MassLynx 4.2 software; ESI refers to the electrospray ionisation technique; LC system: solvent A: 2 mM NH_4OAc in H_2O/MeCN (95:5); solvent B: MeCN; solvent C: 2% formic acid; column: ACQUITY UPLC® CSH C18 (2.1 mm × 50 mm, 1.7 µm, 130 Å) at 40 °C; gradient: 5 – 95 % B with constant 5 % C over 1 min at flow rate of 0.6 mL/min; detector: PDA eλ Detector 220 – 800 nm, interval 1.2 nm.

Flash column chromatography was carried out using slurry-packed Merck 9385 Kieselgel 60 SiO_2 (230-400 mesh) or Combiflash Rf200 automated chromatography system with Redisep® reverse-phase C18-silica flash columns (20-40 µm).

Analytical high performance liquid chromatography (HPLC) was performed on Agilent 1260 Infinity machine, using a Supelcosil™ ABZ+PLUS column (150 mm × 4.6 mm, 3 µm) with a linear gradient system (solvent A: 0.05% (v/v) TFA in H_2O; solvent B: 0.05% (v/v) TFA in MeCN) over 20 min at a flow rate of 1 mL/min, and UV detection (λ_{max} = 220 – 254 nm).

Infrared (IR) spectra were recorded neat on a Perkin-Elmer Spectrum One spectrometer with internal referencing. Selected absorption maxima (ν_{max}) are reported in wavenumbers (cm^{-1}) with peak intensity reported as follows: w = weak; m = medium; s = strong.

^{1}H and ^{13}C nuclear magnetic resonance (NMR) were recorded using an internal deuterium lock on Bruker DPX-400 (400 MHz, 101 MHz), Bruker Avance 400 QNP (400 MHz, 101 MHz) and Bruker Avance 500 Cryo Ultrashield (500 MHz, 126 MHz). Tetramethylsilane was used as an internal standard. In ^{1}H NMR, chemical shifts (δ_H) are reported in parts per million (ppm),

to the nearest 0.01 ppm and are referenced to the residual non-deuterated solvent peak ($CDCl_3$: 7.26, CD_3OD: 3.31). Coupling constants (*J*) are reported in Hertz (Hz) to the nearest 0.1 Hz. Data are reported as follows: chemical shift, multiplicity (s = singlet; d = doublet; t = triplet; q = quartet; quint = quintet; m = multiplet; or as a combination of these, e.g. dd, dt etc.), integration and coupling constant(s). In ^{13}C NMR, chemical shifts (δ_C) are quoted in ppm, to the nearest 0.1 ppm, and are referenced to the residual non-deuterated solvent peak ($CDCl_3$: 77.16, CD_3OD: 49.00).

High resolution mass spectrometry (HRMS) measurements were recorded with a Micromass Q-TOF mass spectrometer or a Waters LCT Premier Time of Flight mass spectrometer. Mass values are reported within the error limits of ±5 ppm mass units. ESI refers to the electrospray ionisation technique.

Protein LC–MS was performed on a Xevo G2-S TOF mass spectrometer coupled to an Acquity UPLC system using an Acquity UPLC BEH300 C4 column (1.7 µm, 2.1 × 50 mm). H_2O with 0.1% formic acid (solvent A) and 95% MeCN and 5% H_2O with 0.1% formic acid (solvent B) were used as the mobile phase at a flow rate of 0.2 mL/min. The gradient was programmed as follows: 95% A for 0.93 min, then a gradient to 100% B over 4.28 min, then 100% B for 1.04 minutes, then a gradient to 95% A over 1.04 min. The electrospray source was operated with a capillary voltage of 2.0 kV and a cone voltage of 190 V. Nitrogen was used as the desolvation gas at a total flow of 850 L/h. Total mass spectra were reconstructed from the ion series using the MaxEnt algorithm preinstalled on MassLynx software (v4.2 from Waters) according to the manufacturer's instructions. Trastuzumab samples were deglycosylated with PNGase F (New England Biolabs) prior to LC-MS analysis.

Chemical Synthesis

Scheme S1. Synthesis of DVP 1.

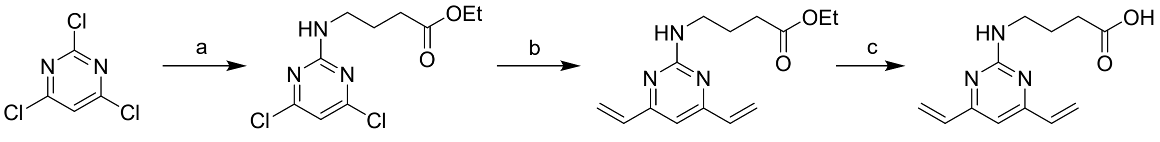

Reagents and conditions: (a) 4-aminobutyric acid ethyl ester hydrochloride, Et_3N, MeOH, rt, 3 h, 37%; (b) potassium vinyltrifluoroborate, $PdCl_2(dppf){\cdot}CH_2Cl_2$, K_2CO_3, THF/H_2O, 70 °C, 14 h, 89%; (c) $LiOH{\cdot}H_2O$, THF/H_2O, rt, 24 h, 100%.

Ethyl 4-((4,6-dichloropyrimidin-2-yl)amino)butanoate (21)

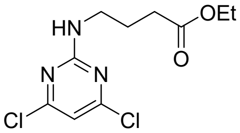

To a solution of 4-aminobutyric acid ethyl ester hydrochloride (2.01 g, 12.0 mmol) and triethylamine (4.18 mL, 30.0 mmol) in methanol (100 mL) was added dropwise 2,4,6-trichloropyrimidine (1.15 mL, 10.0 mmol). After addition, the reaction mixture was kept stirring at room temperature for 3 h before being concentrated *in vacuo*. The residue was purified by column chromatography (0-20% EtOAc/PE) to provide the title compound as a white crystalline solid (1.02 g, 3.67 mmol, 37%). $\mathbf{R_f}$ 0.55 (SiO_2, 30% EtOAc/PE); $\mathbf{\nu_{max}}$ (neat/cm^{-1}) 1747 (s, C=O), 1562 (m, C=C); $\mathbf{\delta_H}$ (400 MHz, CD_3OD) 6.66 (s, 1H), 4.12 (q, 2H, J = 7.1 Hz), 3.40 (t, 2H, J = 6.8 Hz), 2.38 (t, 2H, J = 7.3 Hz), 1.88 (quint, 2H, J = 7.1 Hz), 1.24 (t, 3H, J = 7.1 Hz); $\mathbf{\delta_C}$ (101 MHz, CD_3OD) 175.1, 163.4, 163.1/162.8, 108.8, 61.6, 41.5, 32.3, 25.5, 14.5; **LRMS** (ESI) m/z found $[M+H]^+$ 278.4.

These data are consistent with those previously reported.[1]

Ethyl 4-((4,6-divinylpyrimidin-2-yl)amino)butanoate (22)

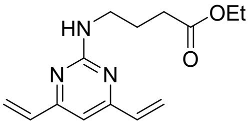

A mixture of ethyl 4-((4,6-dichloropyrimidin-2-yl)amino)butanoate **21** (757 mg, 2.72 mmol), potassium vinyltrifluoroborate (1.09 g, 8.16 mmol), $PdCl_2(dppf){\cdot}CH_2Cl_2$ (333 mg, 0.408 mmol) and K_2CO_3 (1.13 g, 8.16 mmol) in THF (33 mL) and H_2O (3.3 mL) was heated to 70 °C under nitrogen for 14 h. The reaction mixture was cooled to ambient temperature, filtered through Celite®, and concentrated *in vacuo*. The residue was purified by column chromatography (0-20% EtOAc/PE) to yield the product as a colourless oil (630 mg, 2.41 mmol, 89%). $\mathbf{R_f}$ 0.32 (SiO_2,

20% EtOAc/PE); **ν_{max}** (neat/cm^{-1}) 1743 (s, C=O), 1562 (m, C=C); **δ_H** (400 MHz, $CDCl_3$) 6.52 (s, 1H), 6.57 (dd, 2H, *J* = 10.6, 17.3 Hz), 6.35 (d, 2H, *J* = 17.3 Hz), 5.55 (dd, 2H, *J* = 1.5, 10.6 Hz), 5.16 (t, 1H, *J* = 5.4 Hz), 4.12 (q, 2H, *J* = 7.1 Hz), 3.52 (q, 2H, *J* = 6.6 Hz), 2.40 (t, 2H, *J* = 7.4 Hz), 1.95 (quint, 2H, *J* = 7.1 Hz), 1.23 (t, 3H, *J* = 7.1 Hz); **δ_C** (101 MHz, $CDCl_3$) 173.6, 163.8, 162.8, 136.0, 121.5, 105.9, 60.5, 40.8, 31.9, 25.2, 14.3; **LRMS** (ESI) *m/z* found $[M+H]^+$ 262.6.

These data are consistent with those previously reported.[1]

4-((4,6-divinylpyrimidin-2-yl)amino)butanoic acid (1)

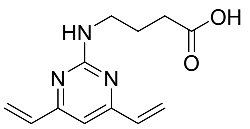

To a solution of ethyl 4-((4,6-divinylpyrimidin-2-yl)amino)butanoate **22** (345 mg, 1.32 mmol) in THF (12 mL) and H_2O (12 mL) at 0 °C was added $LiOH{\cdot}H_2O$ (55.6 mg, 1.32 mmol). The mixture was stirred at room temperature for 24 h, and then diluted with H_2O (30 mL) and washed with Et_2O (30 mL). The aqueous layer was adjusted to pH 4 using 1 M HCl and then extracted with CH_2Cl_2 (5 x 30mL). The organic layer was dried over Na_2SO_4, filtered, and concentrated *in vacuo* to yield the product as a white solid (307 mg, 1.32 mmol, 100%). **ν_{max}** (neat/cm^{-1}) 3277 (m, O-H), 1702 (s, C=O), 1562 (m, C=C); **δ_H** (600 MHz, CD_3OD) 6.70 (s, 1H), 6.61 (dd, 2H, *J* = 10.7, 17.4 Hz), 6.37 (d, 2H, *J* = 17.4 Hz), 5.57 (dd, 2H, *J* = 1.4, 10.7 Hz), 3.48 (t, 2H, *J* = 6.9 Hz), 2.38 (t, 2H, *J* = 7.4 Hz), 1.92 (quint, 2H, J = 7.1 Hz); **δ_C** (101 MHz, CD_3OD) 177.4, 165.4, 164.1, 137.0, 122.1, 105.7, 41.5, 32.4, 26.2; **LRMS** (ESI) *m/z* found $[M+H]^+$ 234.4.

These data are consistent with those previously reported.[1]

Scheme S2. Synthesis of BisDVP 2.

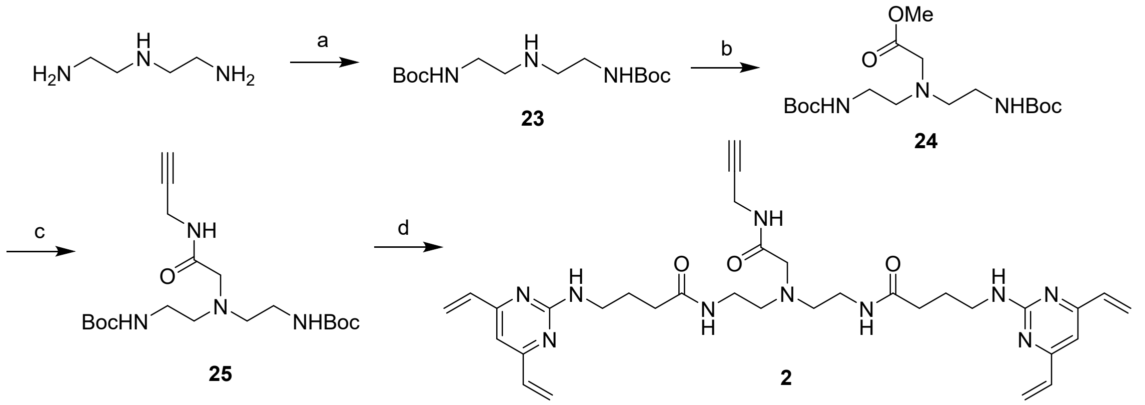

Reagents and conditions: (a) Boc-ON, THF, 0 °C, 1 h, 92%; (b) methyl bromoacetate, DIPEA, DMF, rt, 6 h, 94%; (c) (i) NaOH, MeOH/dioxane, rt, 2 h (ii) Propargylamine, Et_3N, HBTU, DMF, 36 h, 58%; (d) (i) HCl, dioxane/CH_2Cl_2, rt, 6 h (ii) **1**, Et_3N, EDC·HCl, $HOBt{\cdot}H_2O$, DMF, rt, 18 h, 55%.

di-*tert*-butyl (azanediylbis(ethane-2,1-diyl))dicarbamate (23)

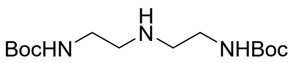

To a solution of diethylenetriamine (2.16 mL, 20.0 mmol) in THF (25 mL) at 0°C was slowly added a solution of Boc-ON (9.92 g, 40.0 mmol) in THF (25 mL). The reaction was stirred at 0 °C under nitrogen for 1 h and then concentrated *in vacuo*. The crude yellow oil was dissolved in CH_2Cl_2 and sequentially washed with 10% aq. NaOH, water and brine. The organic phase was dried over Na_2SO_4 and concentrated *in vacuo*. Purification by column chromatography (10% MeOH/CH_2Cl_2) yielded the product as a colourless oil (5.61 g, 18.5 mmol, 92% yield). **R_f** 0.08 (SiO_2, 10% MeOH/CH_2Cl_2); **$ν_{max}$** (neat/cm^{-1}) 3336 (m, N-H), 2975 (m, C-H), 1687 (s, C=O); **$δ_H$** (400 MHz, $CDCl_3$) 4.93 (br s, 2H), 3.24-3.19 (m, 4H), 2.73 (t, 4H, *J* = 5.7 Hz), 1.44 (s, 18H); **$δ_C$** (101 MHz, $CDCl_3$) 156.3, 79.4, 49.0, 40.4, 28.6; **HRMS** (ESI) *m/z* found $[M+H]^+$ 304.2221, $C_{14}H_{30}O_4N_3^+$ required 304.2231.

These data are consistent with those previously reported.[2]

methyl bis(2-((*tert*-butoxycarbonyl)amino)ethyl)glycinate (24)

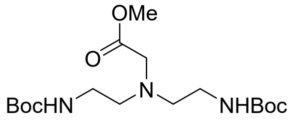

To a solution of di-*tert*-butyl (azanediylbis(ethane-2,1-diyl))dicarbamate **23** (5.61 g, 18.5 mmol) in DMF (80 mL) were added DIPEA (3.86 mL, 22.2 mmol) and methyl bromoacetate (2.61 mL, 27.8 mmol). The reaction was stirred at room temperature for 6 h and then concentrated under a stream of nitrogen. The crude residue was purified by column chromatography (40-50% EtOAc/PE) yielding the product as a colourless oil (6.56 g, 17.5 mmol, 94% yield). **R_f** 0.28 (SiO_2, 50% EtOAc/PE); **$ν_{max}$** (neat/cm^{-1}) 3345 (m, N-H), 2979 (m, C-H), 1740 (s, C=O), 1688 (s, C=O); **$δ_H$** (400 MHz, $CDCl_3$) 5.13 (br s, 2H, NH), 3.70 (s, 3H), 3.37 (s, 2H), 3.15 (q, 4H, *J* = 5.6 Hz), 2.72 (t, 4H, *J* = 5.9 Hz), 1.44 (s, 18H); **$δ_C$** (101 MHz, $CDCl_3$) 172.3, 156.3, 79.3, 55.1, 54.3, 51.8, 38.7, 28.6; **LRMS** (ESI) *m/z* found $[M+H]^+$ 376.5.

These data are consistent with those previously reported.[3]

di-*tert*-butyl (((2-oxo-2-(prop-2-yn-1-ylamino)ethyl)azanediyl)bis(ethane-2,1-diyl))dicarbamate (25)

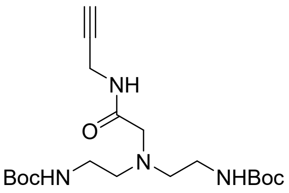

To a solution of methyl bis(2-((*tert*-butoxycarbonyl)amino)ethyl)glycinate **24** (2.0 g, 5.33 mmol) in MeOH (21.6 mL) and 1,4-dioxane (21.6 mL) was added aqueous NaOH (1M, 10.8 mL). The

reaction mixture was stirred at room temperature for 2 h and then concentrated *in vacuo*. The resulting residue was re-dissolved in MeOH, filtered, and concentrated again. The residue was then dissolved in DMF (40 mL), followed by addition of *N*-propargylamine (1.02 mL, 16.0 mmol), triethylamine (2.23 mL, 16.0 mmol) and HBTU (3.03 g, 8.00 mmol). After stirring at room temperature for 36 h, the reaction mixture was diluted with brine (40 mL) and extracted with EtOAc (3 x 40 mL). The combined organic layers were washed with 1 M HCl (40 mL), saturated $NaHCO_3$ (40 mL) and water (40 mL). The organic phase was dried over Na_2SO_4 and concentrated *in vacuo*. Purification by column chromatography (50-100% EtOAc/PE) yielded the product as a pale orange oil (1.24 g, 3.10 mmol, 58% yield). **R$_f$** 0.19 (SiO_2, 80% EtOAc/PE); **ν$_{max}$** (neat/cm^{-1}) 3290 (m, N-H), 2978 (m, C-H), 2112 (m, C≡C), 1657 (s, C=O); **δ$_H$** (600 MHz, CD_3OD) 4.01 (d, 2H, *J* = 2.5 Hz), 3.19 (s, 2H), 3.13 (t, 4H, *J* = 6.2 Hz), 2.60 (t, 4H, *J* = 5.8 Hz), 2.58 (t, 1H, *J* = 2.5 Hz), 1.45 (s, 18H); **δ$_C$** (101 MHz, CD_3OD) 173.8, 158.6, 80.6, 80.2, 72.2, 59.7, 56.2, 39.5, 29.2, 28.8; **HRMS** (ESI) *m/z* found $[M+H]^+$ 399.2593, $C_{19}H_{35}O_5N_4^+$ required 399.2607.

***N,N'*-(((2-oxo-2-(prop-2-yn-1-ylamino)ethyl)azanediyl)bis(ethane-2,1-diyl))bis(4-((4,6-divinylpyrimidin-2-yl)amino)butanamide) (2)**

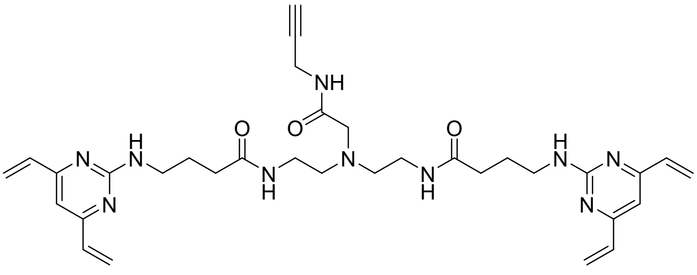

To a solution of **25** (50.0 mg, 0.125 mmol) in CH_2Cl_2 (0.35 mL) at 0 °C was added HCl (4M in dioxane, 0.85 mL). The reaction was stirred under nitrogen for 6 h and then concentrated *in vacuo* to yield the desired amine hydrochloride salt as a white solid.

The amine hydrochloride salt was re-dissolved in DMF (2 mL) and cooled to 0 °C. A solution of 4-((4,6-divinylpyrimidin-2-yl)amino)butanoic acid **1** (58.0 mg, 0.250 mmol), triethylamine (174 µL, 1.25 mmol), EDC·HCl (96.0 mg, 0.500 mmol) and HOBt monohydrate (77.0 mg, 0.500 mmol) in DMF (4 mL) at 0 °C was added. The reaction was stirred under nitrogen for 18 h and then diluted with EtOAc (20 mL), washed with brine (20 mL), dried over Na_2SO_4, and concentrated *in vacuo*. Purification by column chromatography (0-10% MeOH/EtOAc) yielded the product as a colourless oil (43.3 mg, 0.0690 mmol, 55% yield). **R$_f$** 0.17 (SiO_2, 10% MeOH/CH_2Cl_2); **ν$_{max}$** (neat/cm^{-1}) 3291 (m, N-H), 2934 (m, C-H), 2147 (w, C≡C), 1631 (s, C=O), 1539 (s, C=C); **δ$_H$** (600 MHz, CD_3OD) 6.68 (s, 2H), 6.60 (dd, 4H, *J* = 10.7, 17.4 Hz), 6.36 (d, 4H, *J* = 17.4 Hz), 5.56 (dd, 4H, *J* = 1.4, 10.7 Hz), 3.97 (d, 2H, *J* = 2.5 Hz), 3.46 (t, 4H, *J* = 6.8 Hz), 3.24 (t, 4H, *J* = 6.2 Hz), 3.18 (s, 2H), 2.61 (t, 4H, *J* = 6.2 Hz), 2.58 (t, 1H, *J* = 2.5 Hz), 2.31 (t, 4H, *J* = 7.5 Hz), 1.92 (quint, 4H, *J* = 7.1 Hz); **δ$_C$** (101 MHz, CD_3OD) 176.1, 173.7, 165.3, 164.0, 137.1, 122.1, 105.8, 80.6, 72.4, 59.4, 55.8, 41.7, 38.6, 34.7, 29.3, 27.0; **HRMS** (ESI) *m/z* found $[M+H]^+$ 629.3658, $C_{33}H_{45}O_3N_{10}^+$ required 629.3676.

Scheme S3. Synthesis of TetraDVP 3.

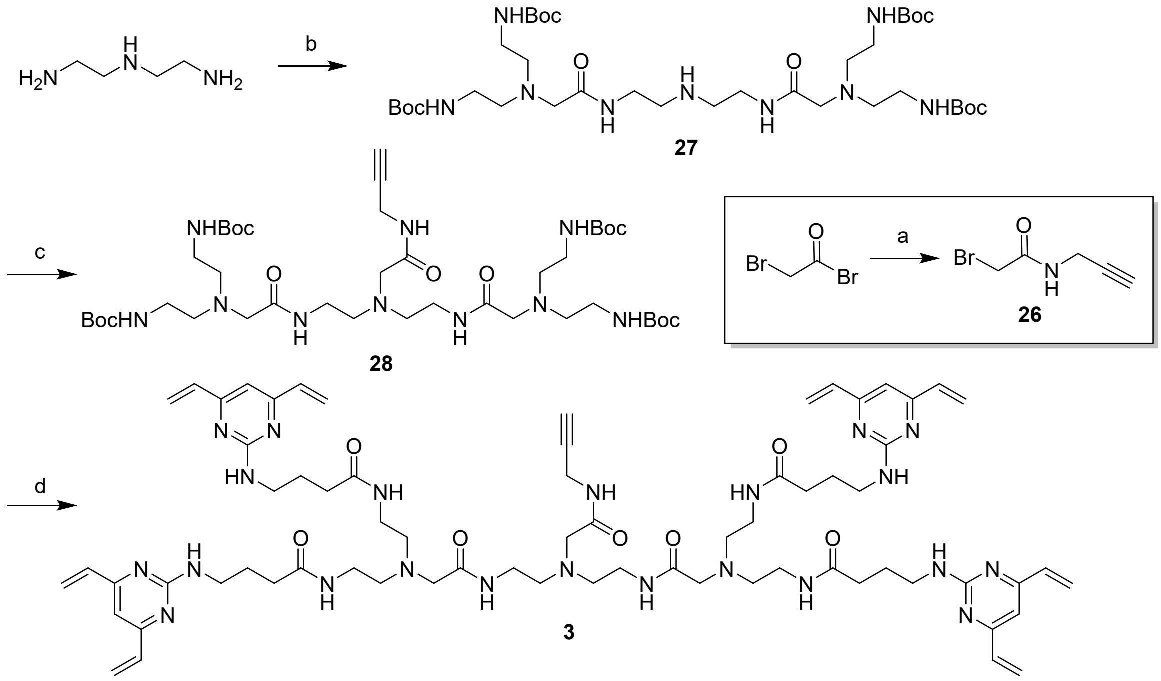

Reagents and conditions: (a) propargylamine, $NaHCO_3$, CH_2Cl_2/H_2O, rt, 93%; (b) **24**, MeOH, 110 °C, 24 h, 78%; (c) **26**, K_2CO_3, MeCN, rt, 24 h, 54%; (d) (i) HCl, dioxane/CH_2Cl_2, rt, 6 h (ii) **1**, Et_3N, HBTU, CH_2Cl_2, rt, 18 h, 28%.

2-bromo-*N*-(prop-2-yn-1-yl)acetamide (26)

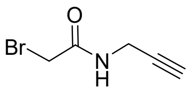

A solution of propargylamine (1.16 mL, 18.2 mmol) in CH_2Cl_2 (33 mL) and sat. $NaHCO_3$ (33 mL) at maximum stirring was cooled to -10 °C and 2-bromoacetyl bromide (2.42 mL, 27.2 mmol) was added dropwise over 15 min. The reaction mixture was allowed to slowly reach rt, and upon completion the reaction mixture was concentrated. Following addition of water (30 mL), the aqueous solution was extracted with EtOAc (2x80 mL) and the combined organic phases were washed with sat. $NaHCO_3$ (30 mL), 5% HCl (30 mL) and brine (30 mL). Combined organic phases were dried over Na_2SO_4 and concentrated *in vacuo* to give the title compound as a light yellow solid (2.97 g, 16.9 mmol, 93%). $\mathbf{R_f}$ 0.69 (SiO_2, 20% MeOH/CH_2Cl_2); $\mathbf{\nu_{max}}$ (neat/cm^{-1}) 3289 (m, N-H), 3071 (m, C-H), 2120 (w, C≡C), 1645 (s, C=O); $\mathbf{\delta_H}$ (400 MHz, $CDCl_3$) 6.68 (s, 1H), 4.09 (dd, *J* = 5.3, 2.6 Hz, 2H), 3.90 (s, 2H), 2.28 (t, *J* = 2.6 Hz, 1H); $\mathbf{\delta_C}$ (100 MHz, $CDCl_3$) 165.1, 78.5, 72.3, 30.0, 28.7; **LRMS** (ESI) *m/z* found $[M+H]^+$ 178.0.

These data are consistent with those previously reported.[4]

Tetra-*N*-Boc-amine backbone, (NH)$_1$ (27)

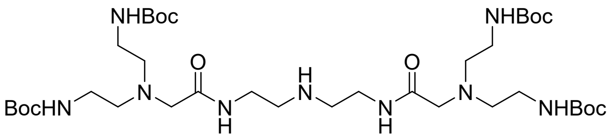

In a pre-dried microwave vial containing methyl bis(2-((*tert*-butoxycarbonyl)amino)ethyl)glycinate **24** (200 mg, 0.530 mmol) dissolved in dry MeOH (0.3 mL) was added diethylenetriamine (10.7 µL, 0.0980 mmol), the vial was capped, flushed with nitrogen, and stirred over night at 110 °C. The reaction mixture was concentrated and purified by column chromatography (0-10% MeOH/CH_2Cl_2) to give the desired compound as a colourless oil (60.6 mg, 0.0770 mmol, 78%). **R$_f$** 0.14 (SiO_2, 10% MeOH/CH_2Cl_2); **ν_{max}** (neat/cm^{-1}) 3316 (m, N-H), 2974 (m, C-H), 1687 (s, C=O); **δ_H** (400 MHz, CD_3OD) 3.48 (t, 4H, *J* = 5.8 Hz), 3.21 (s, 4H), 3.14 (t, 8H, *J* = 6.3 Hz), 3.06 (t, 4H, *J* = 5.7 Hz), 2.61 (t, 8H, *J* = 6.3 Hz), 1.45 (s, 36H); **δ_C** (101 MHz, CD_3OD) 175.6, 158.6, 80.2, 59.9, 56.4, 49.5, 39.7, 38.4, 28.9; **HRMS** (ESI) *m/z* found $[M+H]^+$ 790.5425, $C_{36}H_{72}O_{10}N_9^+$ required 790.5402.

Tetra-*N*-Boc-amine backbone, (alkyne)$_1$ (28)

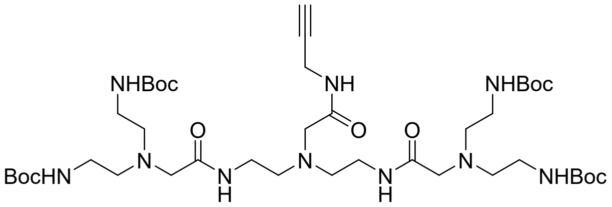

In a pre-dried microwave vial, a suspension of **27** (59.3 mg, 0.0750 mmol) and K_2CO_3 (20.7 mg, 0.150 mmol) in anhydrous MeCN (0.15 mL) was cooled to 0 °C by means of an ice-bath. A solution of **26** (16.5 mg, 0.0940 mmol) in MeCN (0.5 mL) was slowly added to the stirring suspension, after which the reaction was brought to rt and stirred overnight under nitrogen. The reaction mixture was concentrated and purified by flash chromatography (0-10% MeOH/CH_2Cl_2) to give the title compound as a white solid (35.9 mg, 0.0410 mmol, 54%). **R$_f$** 0.30 (SiO_2, 10% MeOH/CH_2Cl_2); **ν_{max}** (neat/cm^{-1}) 3359 (m, N-H), 2985 (m, C-H), 2101 (m, C≡C), 1724 (s, C=O); **δ_H** (600 MHz, $CDCl_3$) 7.79 (s, 2H), 5.70 (s, 4H), 4.04 (dd, 2H, *J* = 2.4, 5.4 Hz), 3.37-3.32 (m, 4H), 3.24 (s, 2H), 3.20-3.14 (m, 8H), 3.14 (s, 4H), 2.75-2.70 (m, 4H), 2.60-2.55 (m, 8H), 2.25-2.22 (m, 1H), 2.10 (s, 1H), 1.42 (s, 36H); **δ_C** (101 MHz, $CDCl_3$) 172.2, 171.5, 156.7, 80.0, 79.4, 71.5, 59.5 (two peaks), 55.8 (two peaks), 38.8, 37.9, 29.0, 28.6; **HRMS** (ESI) *m/z* found $[M+H]^+$ 885.5760, $C_{41}H_{77}O_{11}N_{10}^+$ required 885.5768.

TetraDVP-(alkyne)$_1$ (3)

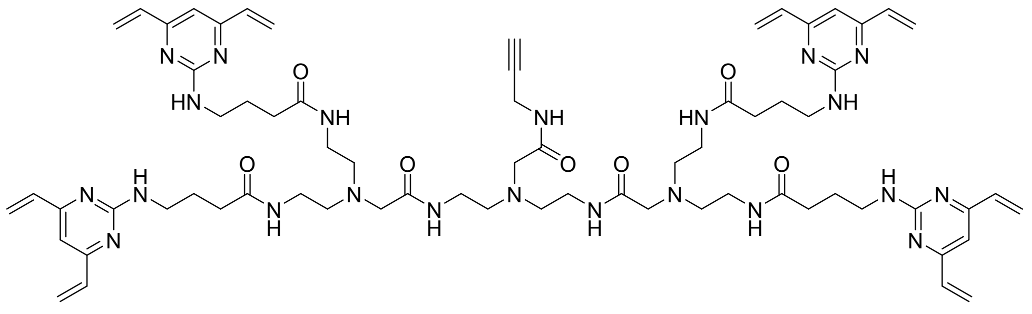

To a solution of **28** (69.0 mg, 0.0780 mmol) in CH_2Cl_2 (0.5 mL) at 0 °C was added HCl (4M in dioxane, 1.0 mL). The reaction was stirred under nitrogen for 6 h and then concentrated *in vacuo* to yield the desired amine hydrochloride salt as a white solid.

The amine hydrochloride salt was re-dissolved in CH_2Cl_2 (1 mL) and cooled to 0 °C. To this solution, a solution of 4-((4,6-divinylpyrimidin-2-yl)amino)butanoic acid **1** (72.8 mg, 0.312 mmol), triethylamine (217 µL, 1.56 mmol) and HBTU (118 mg, 0.312 mmol) in CH_2Cl_2 (2 mL) at 0 °C was added. The reaction was stirred under nitrogen for 18 h and then concentrated *in vacuo*. Purification by column chromatography (2.5-10% MeOH/CH_2Cl_2) yielded the product as a colourless oil (29.6 mg, 0.0220 mmol, 28% over 2 steps). **R_f** 0.15 (SiO_2, 10% MeOH/CH_2Cl_2); **ν_{max}** (neat/cm^{-1}) 3290 (m, N-H), 2944 (m, C-H), 2187 (m, C≡C), 1633 (s, C=O); **δ_H** (600 MHz, CD_3OD) 6.67 (s, 4H), 6.59 (dd, 8H, *J* = 10.7, 17.4 Hz), 6.35 (d, 8H, *J* = 17.4 Hz), 5.55 (dd, 8H, *J* = 1.5, 10.7 Hz), 3.97 (d, 2H, *J* = 2.5), 3.45 (t, 8H, *J* = 6.8 Hz), 3.27 (t, 4H, *J* = 6.5 Hz), 3.24 (t, 8H, *J* = 6.3 Hz), 3.21 (s, 2H), 3.18 (s, 4H), 2.65 (t, 4H, *J* = 6.4 Hz), 2.62-2.59 (m, 1H), 2.60 (t, 8H, *J* = 6.2 Hz), 2.28 (t, 8H, *J* = 7.5 Hz), 1.90 (quint, 8H, *J* = 7.2 Hz); **δ_C** (101 MHz, CD_3OD) 175.9, 174.1, 173.7, 165.3, 164.0, 137.1, 122.1, 105.8, 80.8, 72.5, 59.7, 59.3, 55.9, 55.8, 44.0, 38.7, 38.5, 34.7, 29.4, 27.0; **HRMS** (ESI) *m/z* found $[M+H]^+$ 1345.7930, $C_{69}H_{97}O_7N_{22}^+$ required 1345.7905.

Scheme S4. Synthesis of TetraDVP 7.

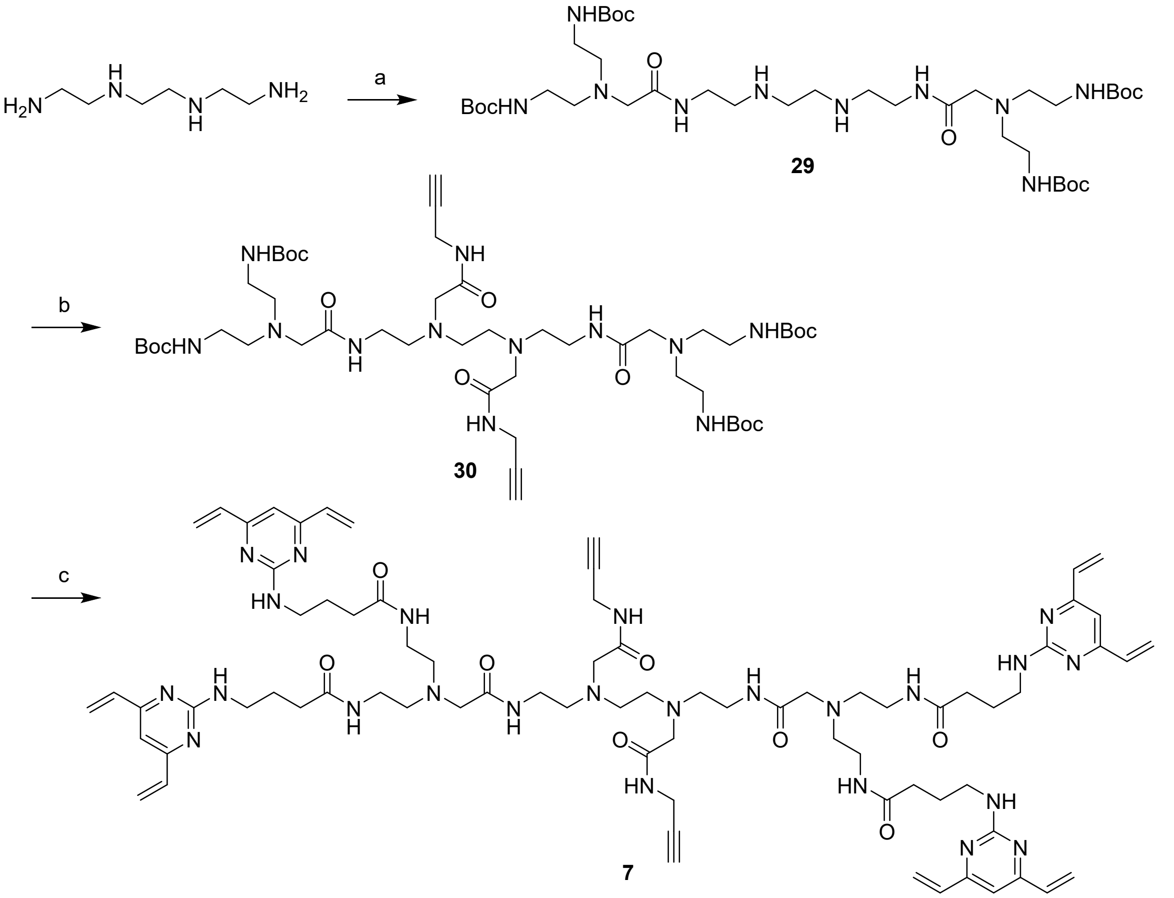

Reagents and conditions: (a) **24**, MeOH, 110 °C, 24 h, 49%; (b) **26**, K_2CO_3, MeCN, rt, 24 h, 62%; (c) (i) HCl, dioxane/CH_2Cl_2, rt, 6 h (ii) **1**, Et_3N, HBTU, CH_2Cl_2, rt, 18 h, 40%.

Tetra-*N*-Boc-amine backbone, $(NH)_2$ (29)

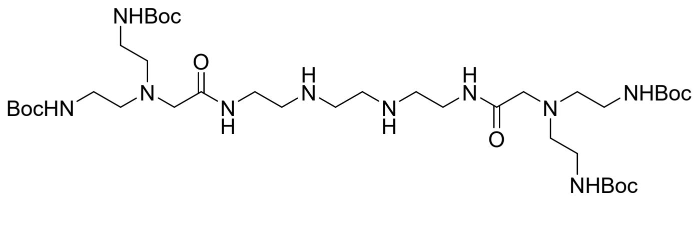

In a pre-dried microwave vial containing methyl bis(2-((*tert*-butoxycarbonyl)amino)ethyl)glycinate **24** (203 mg, 0.540 mmol) dissolved in dry MeOH (0.3 mL) was added triethylenetetramine (14.9 μL, 0.100 mmol), the vial was capped, flushed with nitrogen, and stirred over night at 110 °C. The reaction mixture was concentrated and purified by column chromatography (0-30% MeOH/CH_2Cl_2) to give the desired compound as a colourless oil (40.8 mg, 0.0490 mmol, 49%). **R$_f$** 0.38 (SiO_2, 20% MeOH/CH_2Cl_2); **ν$_{max}$** (neat/cm^{-1}) 3335 (m, N-H), 2980 (m, C-H), 1690 (s, C=O); **δ$_H$** (600 MHz, CD_3OD) 3.38 (t, 4H, *J* = 6.3 Hz), 3.17 (s, 4H), 3.13 (t, 8H, *J* = 6.1 Hz), 2.80-2.77 (m, 4H), 2.76 (s, 4H), 2.60 (t, 8H, *J* = 5.7 Hz), 1.45

(s, 36H); **δ$_C$** (101 MHz, CD_3OD) 174.5, 158.6, 80.2, 60.1, 56.4, 49.7, 49.4, 39.7, 39.7, 28.9; **HRMS** (ESI) *m/z* found $[M+H]^+$ 833.5843, $C_{38}H_{77}O_{10}N_{10}^+$ required 833.5824.

Tetra-*N*-Boc-amine backbone, (alkyne)$_2$ (30)

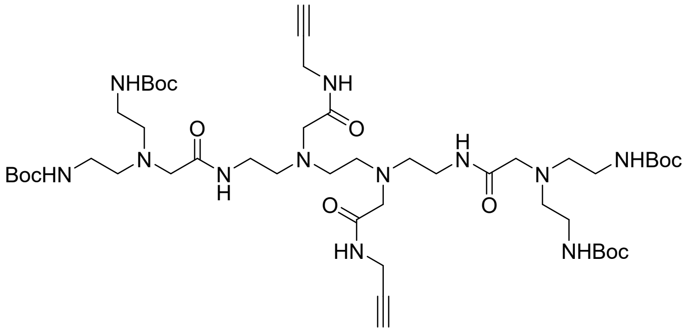

In a pre-dried microwave vial, a suspension of **29** (39.2 mg, 0.0470 mmol) and K_2CO_3 (26.0 mg, 0.188 mmol) in anhydrous MeCN (0.1 mL) was cooled to 0 °C by means of an ice-bath. A solution of **26** (20.8 mg, 0.118 mmol) in MeCN (0.4 mL) was slowly added to the stirring suspension, after which the reaction was brought to rt and stirred overnight under nitrogen. The reaction mixture was concentrated and purified by flash chromatography (0-10% MeOH/CH_2Cl_2) to give the title compound as a white solid (30.0 mg, 0.0290 mmol, 62%). **R$_f$** 0.18 (SiO_2, 10% MeOH/CH_2Cl_2); **ν$_{max}$** (neat/cm^{-1}) 3296 (m, N-H), 2978 (m, C-H), 2157 (m, C≡C), 1654 (s, C=O); **δ$_H$** (600 MHz, CD_3OD) 4.20 (s, 4H), 4.08 (d, 4H, *J* = 2.5 Hz), 3.80 (s, 4H), 3.57 (t, 4H, *J* = 6.0 Hz), 3.51-3.44 (m, 16H), 3.27 (s, 4H), 3.21-3.17 (m, 4H), 2.72 (t, 2H, *J* = 2.5 Hz), 1.47 (s, 36H); **δ$_C$** (101 MHz, CD_3OD) 169.8, 166.3, 159.1, 81.3, 80.3, 73.2, 57.0, 56.1, 56.0, 55.9, 53.3, 37.0, 36.7, 30.0, 28.8; **HRMS** (ESI) *m/z* found $[M+H]^+$ 1023.6547, $C_{48}H_{87}O_{12}N_{12}^+$ required 1023.6566.

TetraDVP-(alkyne)$_2$ (7)

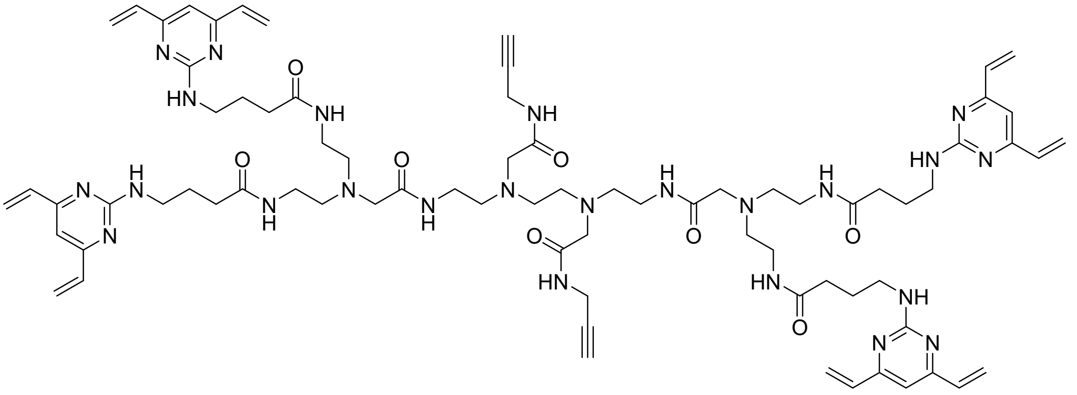

To a solution of **30** (79.8 mg, 0.0780 mmol) in CH_2Cl_2 (0.5 mL) at 0 °C was added HCl (4M in dioxane, 1.0 mL). The reaction was stirred under nitrogen for 6 h and then concentrated *in vacuo* to yield the desired amine hydrochloride salt as a white solid.

The amine hydrochloride salt was re-dissolved in CH_2Cl_2 (1 mL) and cooled to 0 °C. To this solution was added a solution of 4-((4,6-divinylpyrimidin-2-yl)amino)butanoic acid **1** (72.8 mg, 0.312 mmol), triethylamine (217 µL, 1.56 mmol) and HBTU (118 mg, 0.312 mmol) in CH_2Cl_2 (2

mL) at 0 °C. The reaction was stirred under nitrogen for 18 h and then concentrated *in vacuo*. Purification by reverse phase flash column chromatography (35-70% solvent B in solvent A. Solvent A: 100 mM NH_4OH (aq). Solvent B: MeCN) and lyophilisation yielded the product as a colourless oil (46.1 mg, 0.0310 mmol, 40% over 2 steps). $\mathbf{v_{max}}$ (neat/cm^{-1}) 3289 (m, N-H), 2938 (m, C-H), 2186 (m, C≡C), 1637 (s, C=O); $\mathbf{\delta_H}$ (600 MHz, CD_3OD) 6.67 (s, 4H), 6.59 (dd, 8H, *J* = 10.6, 17.4 Hz), 6.35 (d, 8H, *J* = 17.3 Hz), 5.56 (dd, 8H, *J* = 1.2, 10.7 Hz), 3.98 (d, 4H, *J* = 2.3), 3.45 (t, 8H, *J* = 6.7 Hz), 3.27 (t, 4H, *J* = 6.8 Hz), 3.24 (t, 8H, *J* = 6.4 Hz), 3.18 (s, 4H), 3.17 (s, 4H), 2.64-2.57 (m, 4H), 2.64-2.57 (m, 2H), 2.64-2.57 (m, 8H), 2.60 (s, 4H), 2.29 (t, 8H, *J* = 7.5 Hz), 1.91 (quint, 8H, *J* = 7.1 Hz); $\mathbf{\delta_C}$ (101 MHz, CD_3OD) 175.9, 174.1, 173.7, 165.3, 164.0, 137.1, 122.1, 105.8, 80.9, 72.4, 59.7, 59.3, 55.8 (two peaks), 54.4, 41.7, 38.7, 38.4, 34.7, 29.4, 27.0; **HRMS** (ESI) *m/z* found $[M+H]^+$ 1483.8687, $C_{76}H_{107}O_8N_{24}^+$ required 1483.8698.

Scheme S5. Synthesis of TetraDVP 8.

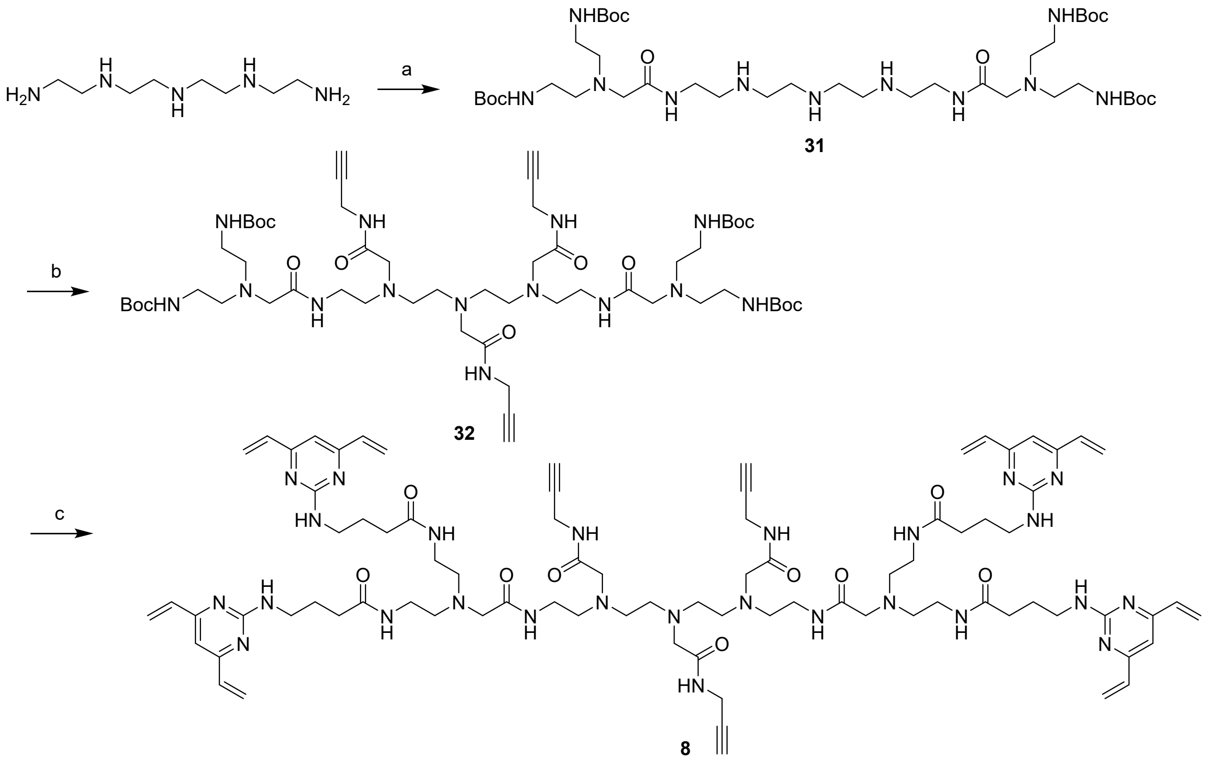

Reagents and conditions: (a) **24**, MeOH, 110 °C, 24 h, 44%; (b) **26**, K_2CO_3, MeCN, rt, 24 h, 49%; (c) (i) HCl, dioxane/CH_2Cl_2, rt, 1 h (ii) **1**, DIPEA, BTFFH, CH_2Cl_2, rt, 15 h, 20%.

Tetra-*N*-Boc-amine backbone, $(NH)_3$ (31)

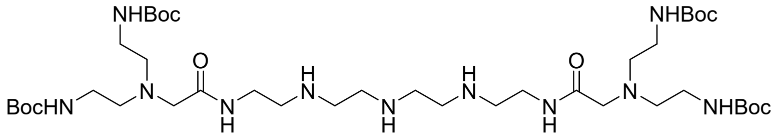

In a pre-dried microwave vial containing methyl bis(2-((*tert*-butoxycarbonyl)amino)ethyl)glycinate **24** (964 mg, 2.57 mmol) dissolved in dry MeOH (1 mL) was added tetraethylenepentamine (90.0 µL, 0.475 mmol), the vial was capped, flushed with nitrogen, and stirred over night at 110 °C. The reaction mixture was concentrated and purified by column chromatography (0-20% MeOH/CH_2Cl_2) to give the desired compound as a white foam (184 mg, 0.210 mmol, 44%). $\mathbf{R_f}$ 0.06 (BuOH:H_2O:MeCN, 0.8/0.1/0.1); $\mathbf{\nu_{max}}$ (neat/cm^{-1}) 3330 (m, N-H), 2980 (m, C-H), 1689 (s, C=O); $\mathbf{\delta_H}$ (400 MHz, $CDCl_3$) 3.40 – 3.30 (m, 4H), 3.16 – 3.09 (m, 8H), 3.07 (s, 4H), 2.83 – 2.66 (m, 12H), 2.60 – 2.52 (m, 8H), 1.40 (s, 36H); $\mathbf{\delta_C}$ (100 MHz, $CDCl_3$) 171.5, 156.6, 79.3, 59.2, 55.4, 48.6 (three peaks), 38.7, 38.6, 28.6; **HRMS** (ESI) *m/z* found $[M+H]^+$ 876.6284, $C_{40}H_{82}N_{11}NaO_{10}^+$ required 876.6246.

Tetra-*N*-Boc-amine backbone, $(alkyne)_3$ (32)

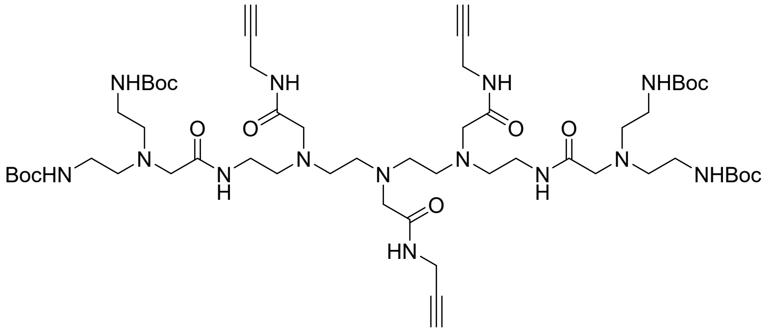

In a pre-dried microwave vial, a suspension of **31** (184 mg, 0.21 mmol) and K_2CO_3 (174 mg, 1.26 mmol) in anhydrous MeCN (0.26 mL) was cooled to 0 °C by means of an ice-bath. A solution of **26** (anh. MeCN, 0.5 M, 1.58 mL) was slowly added to the stirring suspension, after which the reaction was brought to rt and stirred overnight under nitrogen. The reaction mixture was concentrated, re-dissolved in CH_2Cl_2 (40 mL), the organic phase washed with water (2x10 mL), brine (20 mL), dried over Na_2SO_4, and purified by flash chromatography (5-10% MeOH/CH_2Cl_2) to give the title compound as a white foam (119 mg, 0.102 mmol, 49%). $\mathbf{R_f}$ 0.5 (SiO_2, 20% MeOH/CH_2Cl_2); $\mathbf{\nu_{max}}$ (neat/cm^{-1}) 3309 (m, N-H), 2928 (m, C-H), 2158 (m, C≡C), 1658 (s, C=O); $\mathbf{\delta_H}$ (400 MHz, CD_3OD) 4.05 (d, *J* = 2.6 Hz, 4H), 4.03 (d, *J* = 2.5 Hz, 2H), 3.39 – 3.32 (m, 7H), 3.27 (s, 4H), 3.25 (s, 2H), 3.20 (s, 4H), 3.17 – 3.13 (m, 8H), 2.77-2.67 (m, 8H), 2.66-2.58 (m, 8H), 1.47 (s, 36H); $\mathbf{\delta_C}$ (100 MHz, CD_3OD) 174.3, 173.7, 173.6, 158.4, 80.9, 80.8, 80.1, 72.6, 72.3, 60.2, 59.3, 59.2, 56.5, 55.7, 54.4, 54.3, 39.7, 38.5, 29.5, 29.3, 28.9; **HRMS** (ESI) *m/z* found $[M+H]^+$ 1161.7365, $C_{55}H_{97}O_{13}N_{14}^+$ required 1161.7360.

TetraDVP-(alkyne)$_3$ (8)

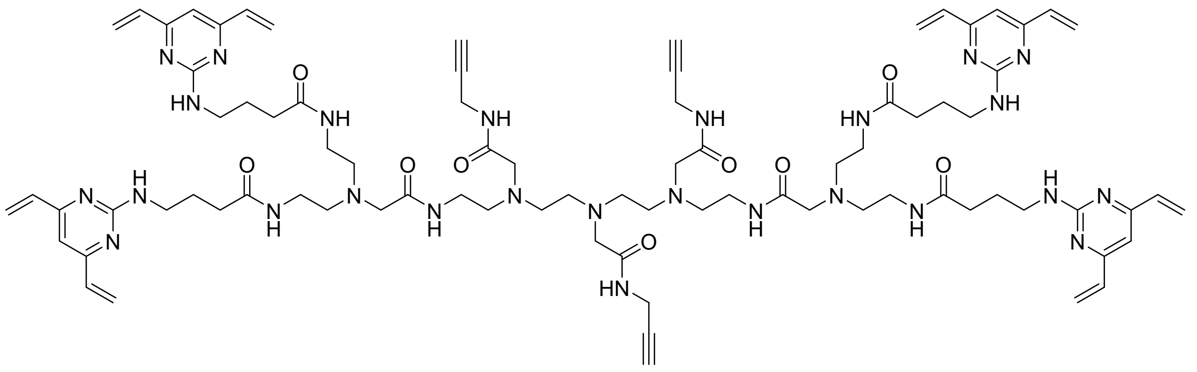

To a solution of **32** (25.0 mg, 22.0 μmol) in CH_2Cl_2 (150 μL) at 0 °C was added HCl (4 M in dioxane, 270 uL). The reaction was brought to rt and stirred for 1 h. The reaction mixture was concentrated to give the desired amine hydrochloride salt as a white solid.

The amine hydrochloride salt (23.0 mg, 22.0 μmol) was suspended in CH_2Cl_2 (150 μL), cooled to 0 °C by means of an ice-bath, and added first DIPEA (74.0 μL, 0.420 mmol) and then **1** (25.0 mg, 0.110 mmol). The solution was stirred at rt for 10 min, after which a solution of BTFFH in CH_2Cl_2 (0.6 M, 205 μL) was added dropwise over 3 h during maximum stirring. After complete addition of BTFFH the reaction was stirred for an additional 12 h at rt, concentrated, and purified by reverse phase flash column chromatography (35-70% solvent B in solvent A. Solvent A: 100 mM NH_4OH (aq). Solvent B: MeCN) to yield the desired product after lyophilization as a pale yellow solid (6.70 mg, 41.4 μmol, 20% over 2 steps). **ν_{max}** (neat/cm^{-1}) 3305 (m, N-H), 2970 (m, C-H), 2156 (m, C≡C), 1637 (s, C=O); **δ_H** (400 MHz, CD_3OD) 6.68 (s, 4H), 6.60 (dd, *J* = 17.4, 10.7 Hz, 8H), 6.36 (d, *J* = 17.3 Hz, 8H), 5.56 (dd, *J* = 10.6, 1.6 Hz, 8H), 4.02 – 3.96 (m, 6H), 3.45 (t, *J* = 6.8 Hz, 8H), 3.35 (s, 4H), 3.29 – 3.15 (m, 21H), 2.67 – 2.56 (m, 20H), 2.29 (t, *J* = 7.5 Hz, 8H), 1.91 (quint, *J* = 7.0 Hz, 8H); **δ_C** (126 MHz, CD_3OD) 175.9, 174.1, 173.8, 173.7, 165.3, 164.0, 137.1, 122.1, 105.8, 81.0 (two peaks), 72.5, 72.4, 59.8, 59.4, 59.3, 55.9, 55.8, 54.5 (two peaks), 54.3 (two peaks), 41.7, 38.7, 38.4, 34.7, 29.4, 29.3, 27.0; **HRMS** (ESI) *m/z* found $[M+H]^+$ 1621.9491, $C_{83}H_{117}N_{26}O_9^+$required 1621.9497.

Scheme S6. Synthesis of TetraDVP 9.

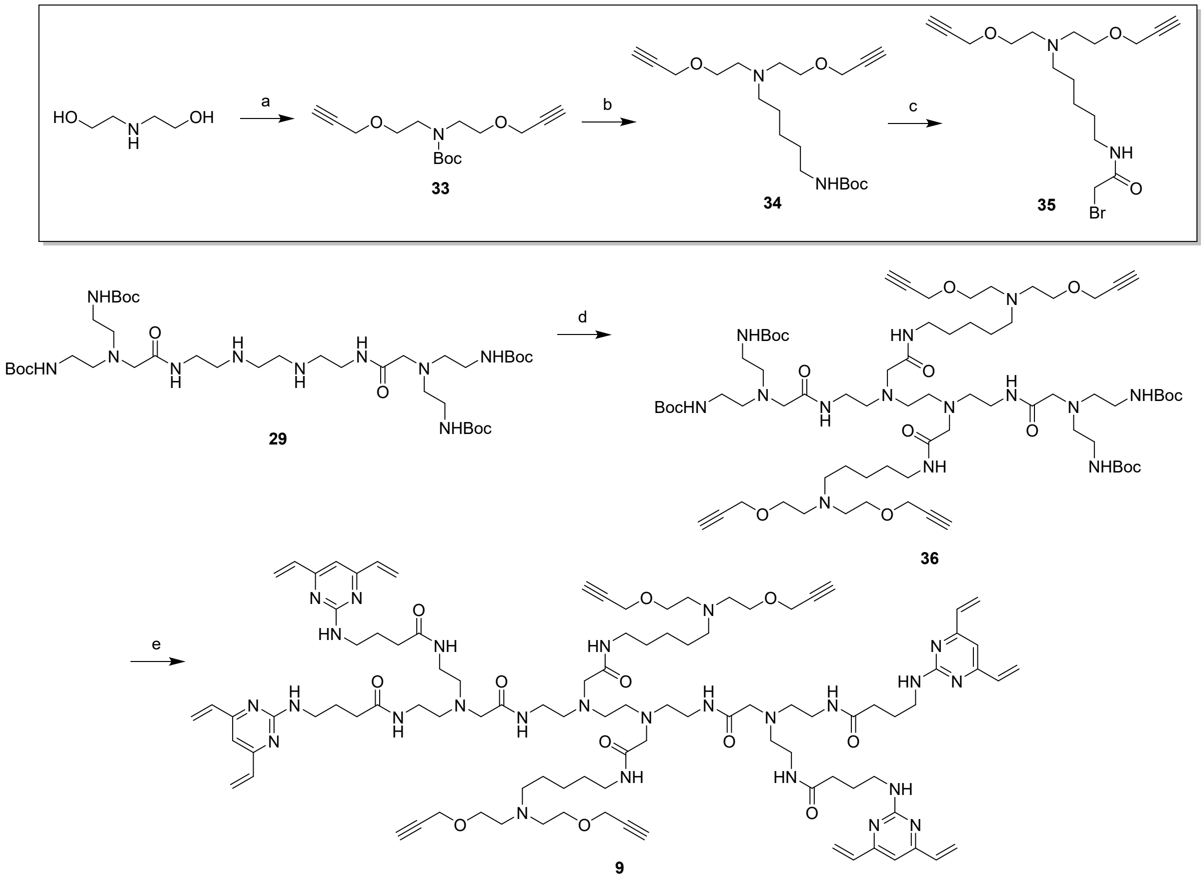

Reagents and conditions: (a) (i) di-*tert*-butyl dicarbonate, CH_2Cl_2, rt, 12 h (ii) NaOH, tetrabutylammonium bisulfate, propargyl bromide, toluene/H_2O, rt, 48 h, 35%; (b) (i) HCl, dioxane/CH_2Cl_2, rt, 2 h (ii) Na_2CO_3, *tert*-Butyl (5-bromopentyl)carbamate, MeCN, 70 °C, 48 h, 66%; (c) (i) HCl, dioxane/CH_2Cl_2, rt, 2 h (ii) K_2CO_3, bromoacetyl bromide, MeCN, -10°C, 1 h, 55%; (d) **35**, K_2CO_3, MeCN, rt, 48 h, 45%; (e) (i) HCl, dioxane/CH_2Cl_2, rt, 2 h (ii) **1**, Et_3N, HBTU, CH_2Cl_2, rt, 18 h, 10%.

tert-butyl bis(2-(prop-2-yn-1-yloxy)ethyl)carbamate (33)

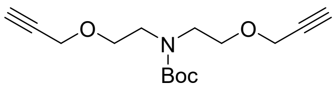

A solution of diethanolamine (2.44 g, 23.0 mmol) in CH_2Cl_2 (25 mL) was cooled to 0 °C by means of an ice-bath and a solution of di-*tert*-butyl dicarbonate (6.33 g, 29.0 mmol) in anh. CH_2Cl_2 (5 mL) as slowly added. The reaction was stirred for 12 h at rt, diluted in CH_2Cl_2 (40 mL), added water (40 mL), and the aqueous phase was extracted twice with CH_2Cl_2 (2x20 mL). The combined organic phases were washed with brine, dried over Na_2SO_4, and concentrated to give *tert*-butyl bis(2-hydroxyethyl)carbamate as a clear oil, which was used directly in the next step without further purification.

To a 50% $NaOH_{(aq)}$ solution (11 mL) was sequentially added *tert*-butyl bis(2-hydroxyethyl)carbamate in toluene (11 mL), tetrabutylammonium bisulfate (16.0 mg, 47.0

μmol), and propargyl bromide (80 w% in toluene, 8.70 mL, 78.0 mmol), and the reaction was stirred at rt for 48 h under nitrogen atmosphere. The organic layer was isolated, concentrated and the desired compound was purified by flash chromatography (50% EtOAc/hexane) to give the title compound as a yellow viscous oil (2.30 g, 7.57 mmol, 35% over 2 steps). $\mathbf{R_f}$ 0.60 (SiO_2, 50% EtOAc/hexane); $\mathbf{\nu_{max}}$ (neat/cm^{-1}) 2975 (m, C-H), 2117 (m, C≡C), 1686 (s, C=O); $\mathbf{\delta_H}$ (400 MHz, $CDCl_3$) 4.13 (d, *J* = 2.4 Hz, 4H), 3.66 – 3.57 (m, 4H), 3.52 – 3.38 (m, 4H), 2.41 (t, *J* = 2.4 Hz, 2H), 1.45 (s, 9H); $\mathbf{\delta_C}$ (100 MHz, $CDCl_3$) 155.6, 79.9, 79.8, 74.5, 68.7, 58.3, 47.9, 28.6; **LRMS** (ESI) *m/z* found $[M+Na]^+$ 304.2.

These data are consistent with those previously reported.[5,6]

***tert*-butyl (5-(bis(2-(prop-2-yn-1-yloxy)ethyl)amino)pentyl)carbamate (34)**

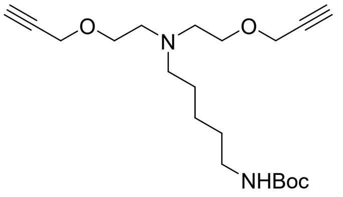

A solution of **33** (281 mg, 1.00 mmol) in CH_2Cl_2 (1.5 mL) was cooled to 0 °C, followed by addition of HCl (4 M in dioxane, 3 mL). The reaction was brought to rt and stirred for 2 h. The reaction mixture was concentrated to give the desired amine hydrochloride salt as a white solid.

The solid was re-suspended in MeCN (10 mL). Sodium carbonate (1.06 g, 10.0 mmol) was added. After stirring for 5 min, a solution of *tert*-Butyl (5-bromopentyl)carbamate (399 mg, 1.50 mmol) in MeCN (2 mL) was added. The reaction mixture was then refluxed at 70 °C for 48 h. Subsequently, the reaction mixture was diluted with brine and extracted with EtOAc (3x). The combined organic layers were dried over Na_2SO_4, concentrated, and purified by flash chromatography (30-50% EtOAc/hexane) to give the title compound as a pale yellow oil (242 mg, 66% over 2 steps). $\mathbf{R_f}$ 0.21 (SiO_2, 80% EtOAc/PE); $\mathbf{\nu_{max}}$ (neat/cm^{-1}) 3342 (m, N-H), 2938 (m, C-H), 2111 (m, C≡C), 1676 (s, C=O); $\mathbf{\delta_H}$ (400 MHz, $CDCl_3$) 4.54 (br s, 1H), 4.16 (d, *J* = 2.3 Hz, 4H), 3.60 (t, *J* = 6.0 Hz, 4H), 3.14 – 3.06 (m, 2H), 2.73 (t, *J* = 6.0 Hz, 4H), 2.55 – 2.49 (m, 2H), 2.42 (t, *J* = 2.2 Hz, 2H), 1.51 – 1.42 (m, 4H), 1.44 (s, 9H), 1.30 (quint, *J* = 7.6 Hz, 2H); $\mathbf{\delta_C}$ (100 MHz, $CDCl_3$) 156.0, 79.9, 79.0, 74.3, 68.3, 58.2, 55.0, 53.8, 40.5, 29.9, 28.4, 26.7, 24.5; **HRMS** (ESI) *m/z* found $[M+H]^+$ 367.2587, $C_{20}H_{34}O_4N_2^+$ required 367.2597.

N-(5-(bis(2-(prop-2-yn-1-yloxy)ethyl)amino)pentyl)-2-bromoacetamide (35)

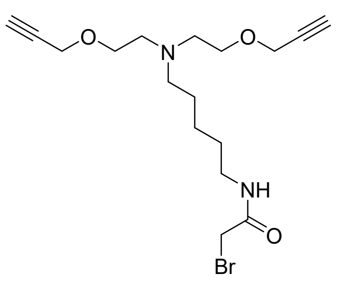

To a solution of **34** (100 mg, 0.270 mmol) in CH_2Cl_2 (0.5 mL) at 0 °C was added HCl (4M in dioxane, 1 mL). The reaction was stirred under nitrogen for 2 h and then concentrated *in vacuo* to yield the desired amine hydrochloride salt as a white solid.

The hydrochloride salt was re-dissolved in a mixture of CH_2Cl_2 (1 mL) and sat. $NaHCO_3$ (1 mL) and cooled to -10 °C, followed by dropwise addition of 2-bromoacetyl bromide (35.3 µL, 0.405 mmol). The reaction mixture was stirred for 1 h, and then diluted with brine and extracted with CH_2Cl_2 (x3). Combined organic phases were dried over Na_2SO_4 and concentrated *in vacuo.* Purification by flash chromatography (0-5% MeOH/CH_2Cl_2) yielded the title compound as a pale yellow oil (57.3 mg, 0.148 mmol, 55% over 2 steps). **R_f** 0.41 (SiO_2, 10% MeOH/CH_2Cl_2); **ν_{max}** (neat/cm^{-1}) 3219 (m, N-H), 2928 (m, C-H), 2111 (m, C≡C), 1672 (s, C=O); **δ_H** (400 MHz, $CDCl_3$) 6.55 (br s, 1H), 4.15 (d, *J* = 2.4 Hz, 4H), 3.87 (s, 2H), 3.62 (t, *J* = 5.5 Hz, 4H), 3.27 (q, 2H, *J* = 6.7 Hz), 2.78 – 2.75 (m, 4H), 2.58 – 2.54 (m, 2H), 2.43 (t, *J* = 2.4 Hz, 2H), 1.55 (quint, *J* = 7.4 Hz, 2H), 1.50 (quint, *J* = 7.4 Hz, 2H), 1.33 (quint, *J* = 7.6 Hz, 2H); **δ_C** (100 MHz, $CDCl_3$) 165.5, 79.7, 74.8, 67.8, 58.4, 55.0, 53.9, 40.2, 29.8, 29.5, 29.1, 24.5; **HRMS** (ESI) *m/z* found $[M+H]^+$ 387.1273, $C_{17}H_{28}O_3N_2Br^+$ required 387.1283.

Tetra-N-Boc-amine backbone, $(alkyne)_4$ (36)

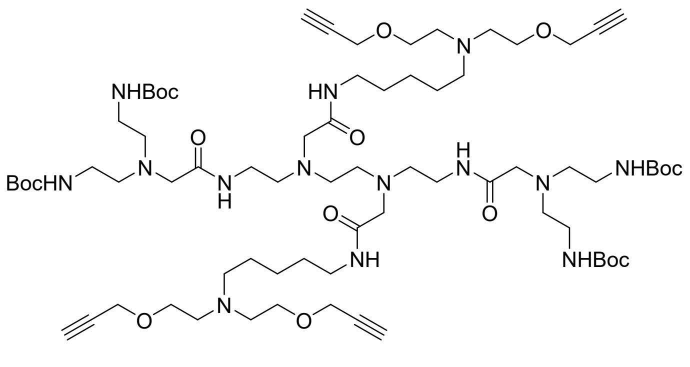

To a solution of **29** (49.3 mg, 59.2 µmol) in MeCN (1 mL) was added anh. K_2CO_3 (32.7 mg, 237 µmol), followed by a solution of *N*-(5-(bis(2-(prop-2-yn-1-yloxy)ethyl)amino)pentyl)-2-bromoacetamide **35** (57.3 mg, 148 µmol) in MeCN (0.5 mL). The reaction was stirred at room temperature for 48 h. The reaction mixture was concentrated, and the desired compound purified by flash chromatography (0%-15% MeOH/CH_2Cl_2) to give the title compound as a clear oil (38.8 mg, 26.6 µmol, 45%). **R_f** 0.35 (15% MeOH/CH_2Cl_2); **ν_{max}** (neat/cm^{-1}) 3283 (m, N-

H), 2936 (m, C-H), 2155 (m, C≡C), 1654 (s, C=O); **δ_H** (400 MHz, CD_3OD) 4.17 (s, 4H), 3.66 (t, *J* = 5.5 Hz, 8H), 3.32 – 3.29 (m, 8H), 3.26 – 3.21 (m, 4H), 3.20 (s, 4H), 3.16 (s, 4H), 3.13 (t, *J* = 5.8 Hz, 8H), 2.89 – 2.84 (m, 8H), 2.70 – 2.65 (m, 12H), 2.59 (t, *J* = 5.7 Hz, 8H), 1.61 – 1.50 (m, 8H), 1.44 (s, 36H), 1.38 – 1.30 (m, 4H); **δ_C** (126 MHz, CD_3OD) 174.3, 173.8, 158.5, 80.7, 80.2, 76.2, 68.3, 60.2, 59.6, 59.0, 56.5 (two peaks), 56.0 (two peaks), 54.6, 40.3, 39.7, 38.6, 30.5, 29.0, 26.8, 25.8; **HRMS** (ESI) *m/z* found $[M+H]^+$ 1445.9692, $C_{72}H_{129}N_{14}O_{16}^+$ required 1445.9711.

TetraDVP-(alkyne)$_4$ (9)

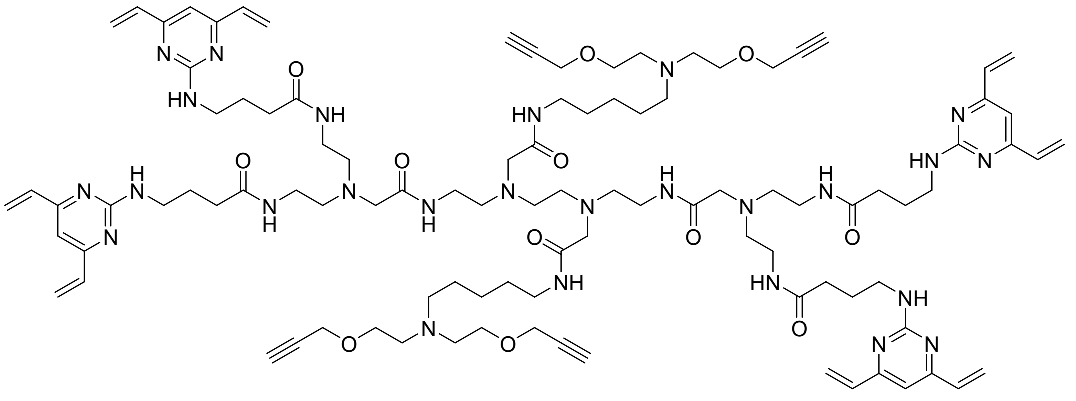

To a solution of **36** (13.0 mg, 8.99 µmol) in CH_2Cl_2 (0.25 mL) at 0 °C was added HCl (4M in dioxane, 0.5 mL). The reaction was stirred under nitrogen for 2 h and then concentrated *in vacuo* to yield the desired amine hydrochloride salt as a white solid.

The amine hydrochloride salt was re-dissolved in CH_2Cl_2 (0.25 mL) and cooled to 0 °C. To this solution was added a solution of 4-((4,6-divinylpyrimidin-2-yl)amino)butanoic acid **1** (12.6 mg, 53.9 µmol), triethylamine (25.0 µL, 180 µmol) and HBTU (20.5 mg, 53.9 µmol) in CH_2Cl_2 (0.25 mL) at 0 °C. The reaction was stirred under nitrogen for 18 h and then concentrated *in vacuo*. Purification by reverse phase flash column chromatography (35-70% solvent B in solvent A. Solvent A: 100 mM NH_4OH (aq). Solvent B: MeCN) and lyophilisation yielded the product as a white solid (1.80 mg, 0.940 µmol, 10% over 2 steps). **ν_{max}** (neat/cm^{-1}) 3299 (m, N-H), 2928 (m, C-H), 2153 (m, C≡C), 1645 (s, C=O); **δ_H** (400 MHz, CD_3OD) 6.69 (s, 4H), 6.60 (dd, *J* = 17.4, 10.7 Hz, 8H), 6.36 (d, *J* = 17.3 Hz, 8H), 5.57 (dd, *J* = 10.7, 1.5 Hz, 8H), 4.23 (d, *J* = 2.4 Hz, 8H), 3.83 (t, *J* = 4.9 Hz, 8H), 3.45 (t, *J* = 6.8 Hz, 8H), 3.30 – 3.15 (m, 32H), 3.00 (t, *J* = 2.3 Hz, 4H), 2.65 – 2.60 (m, 24H), 2.29 (t, *J* = 7.5 Hz, 8H), 1.91 (quint, *J* = 7.1 Hz, 8H), 1.56 (quint, *J* = 7.5 Hz, 4H), 1.39 – 1.30 (m, 8H); **δ_C** (126 MHz, CD_3OD) 175.9, 174.2 (two peaks), 165.3, 164.0, 137.2, 122.2, 105.9, 89.9, 77.2, 68.8, 59.9 (two peaks), 59.2, 56.1, 56.0, 55.3, 54.0, 47.1, 41.8, 40.4, 38.8 (two peaks), 34.7, 30.2, 26.8, 27.1, 25.0; **HRMS** (ESI) *m/z* found $[M+H]^+$ 1906.1851, $C_{100}H_{149}N_{26}O_{12}^+$ required 1906.1848.

Scheme S7. Synthesis of azide-PEG4-Val-Cit-PABC-MMAE 38.

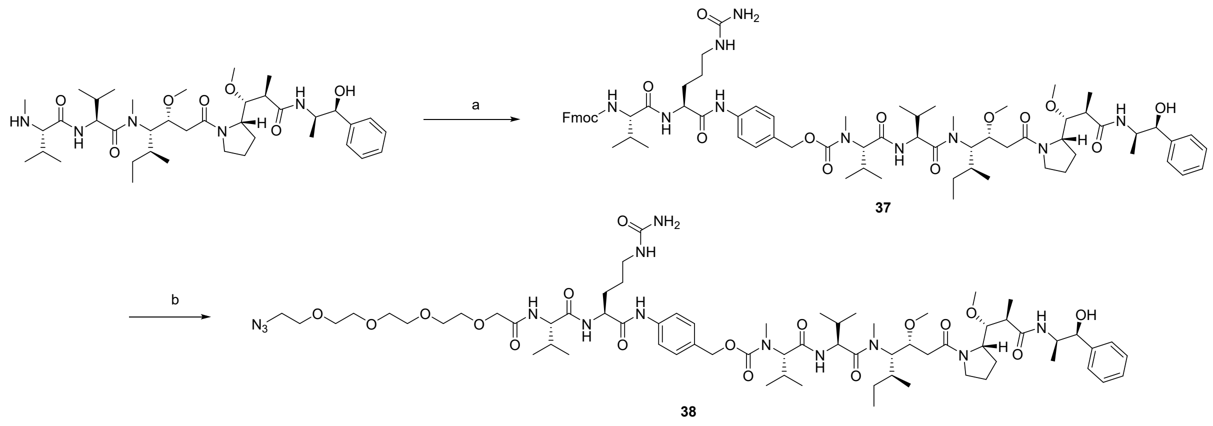

Reagents and conditions: (a) Fmoc-Val-Cit-PABC-PNP, HOBt·H_2O, DIPEA, pyridine, DMF, 24 h, 96%; (b) (i) piperidine, DMF, 2 h (ii) N_3-PEG_4-CO_2H, HBTU, DIPEA, DMF, 18 h, 66%.

Fmoc-Val-Cit-PABC-MMAE (37)

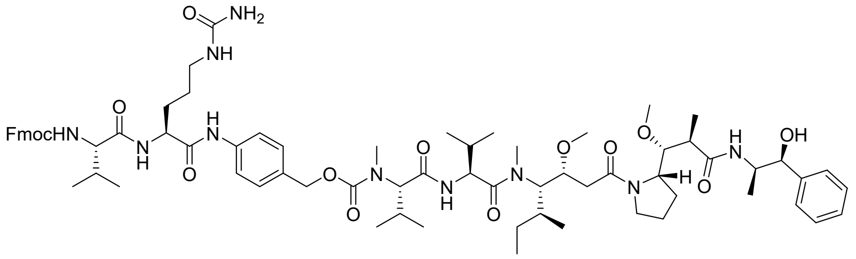

A solution of Fmoc-Val-Cit-PABC-PNP (21.6 mg, 30.1 μmol), MMAE (30.0 mg, 39.1 μmol), HOBt·H_2O (10.1 mg, 60.0 μmol), DIPEA (15.7 μL, 90.0 μmol) and pyridine (24.3 μL, 300 μmol) in DMF (1 mL) was stirred at rt for 24 h. Upon completion, the reaction mixture was purified by automated reverse phase FCC (10-70% solvent B in solvent A. Solvent A: 0.1 M NH4OH (aq). Solvent B: MeCN) and lyophilised to yield the desired compound (39.0 mg, 29.0 μmol, 96%) as a white solid. **HPLC** (5-95% solvent B in solvent A) retention time 13.189 min; **LRMS** (ESI) *m/z* found $[M+Na]^+$ 1368.6, $C_{73}H_{104}N_{10}{}^{23}Na_1O_{14}{}^+$ required 1367.8.

N_3-PEG_4-Val-Cit-PABC-MMAE (38)

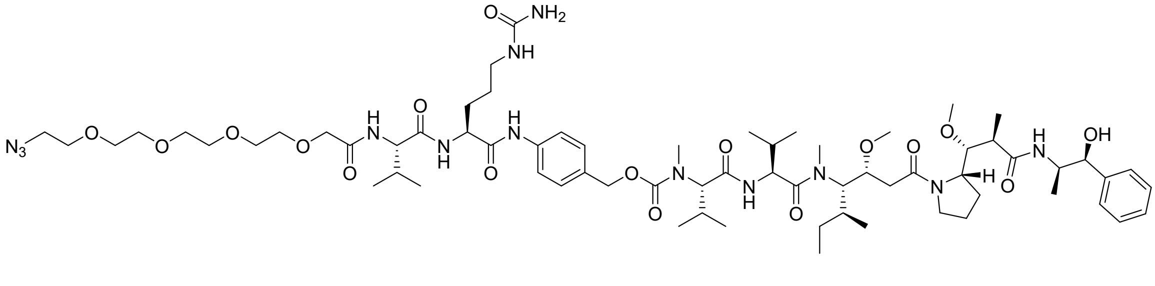

To a solution of Fmoc-Val-Cit-PABC-MMAE (**37**) (20.0 mg, 14.9 μmol) in DMF (2 mL) was added piperidine (7.30 μL, 74.3 μmol) and the mixture stirred at rt for 2 h. Upon completion, the

reaction was concentrated under a stream of N_2. To the free amine was added HBTU (11.3 mg, 29.8 µmol) and DMF (2 mL) followed by N_3-PEG$_4$-CO_2H (50 µL of a 0.5 M solution in TBME, 22.4 µmol) and DIPEA (7.80 µL, 44.7 µmol) and the reaction mixture stirred at rt for 18 h. Upon completion, the reaction was concentrated under a stream of N_2 and purified by automated reverse phase FCC (10-100% solvent B in solvent A. Solvent A: 0.1 M NH_4OH (aq). Solvent B: MeCN) and lyophilised to yield the title compound (13.6 mg, 9.80 µmol, 66%) as a white solid. **HPLC** (5-95% solvent B in solvent A) retention time 11.319 min; **LRMS** (ESI) *m/z* found $[M+H]^+$ 1282.8, $C_{68}H_{112}N_{13}O_{17}^+$ required 1382.8, *m/z* found $[M+HCOO]^-$ 1428.0, $C_{69}H_{112}N_{13}O_{19}^-$ required 1426.8.

Bioconjugation

Reaction of trastuzumab with DVP 1

To a solution of trastuzumab (11.9 µL, 17 µM, 2.5 mg/mL) in TBS (25 mM Tris HCl pH 8, 25 mM NaCl, 0.5 mM EDTA) was added TCEP (10 equiv.). The mixture was vortexed and incubated at 37 °C for 1 h. A solution of **1** (10 mM in DMSO) was added (final concentration of 340 µM, 20 equiv.) and the reaction mixture incubated at 37 °C for 4 h. The excess reagents were removed by use of a Zeba™ Spin Desalting Column (7,000 MWCO, Thermo Fisher Scientific), followed by repeated diafiltration into PBS using an Amicon-Ultra centrifugal filter (10,000 MWCO, Merck Millipore). LC-MS and SDS-PAGE analysis demonstrated >95% conversion to the bridged conjugate.

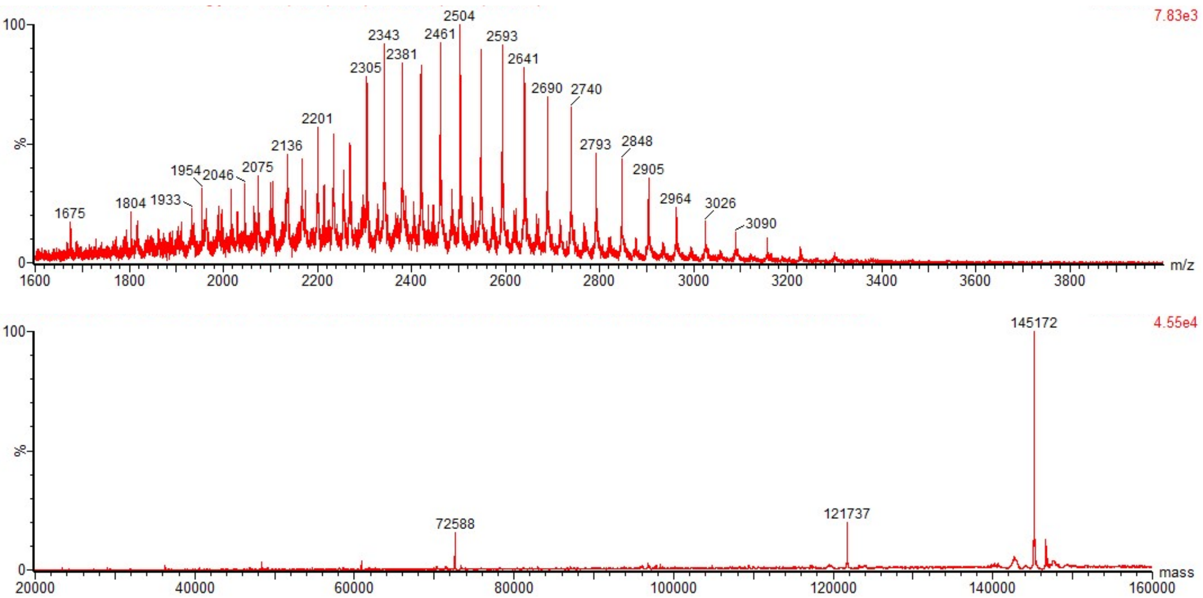

Figure S1. LC-MS of unmodified trastuzumab. Top = non-deconvoluted MS. Bottom = deconvoluted MS; expected 145,160 Da, observed 145,172 Da. Mass differences of up to 50 Da are within the normal expected range for proteins of this size under ESI ionisation conditions. The peaks at 72,588 Da and 121,737 Da may correspond to partially reduced antibody species. Peak intensities are not representative of abundance.

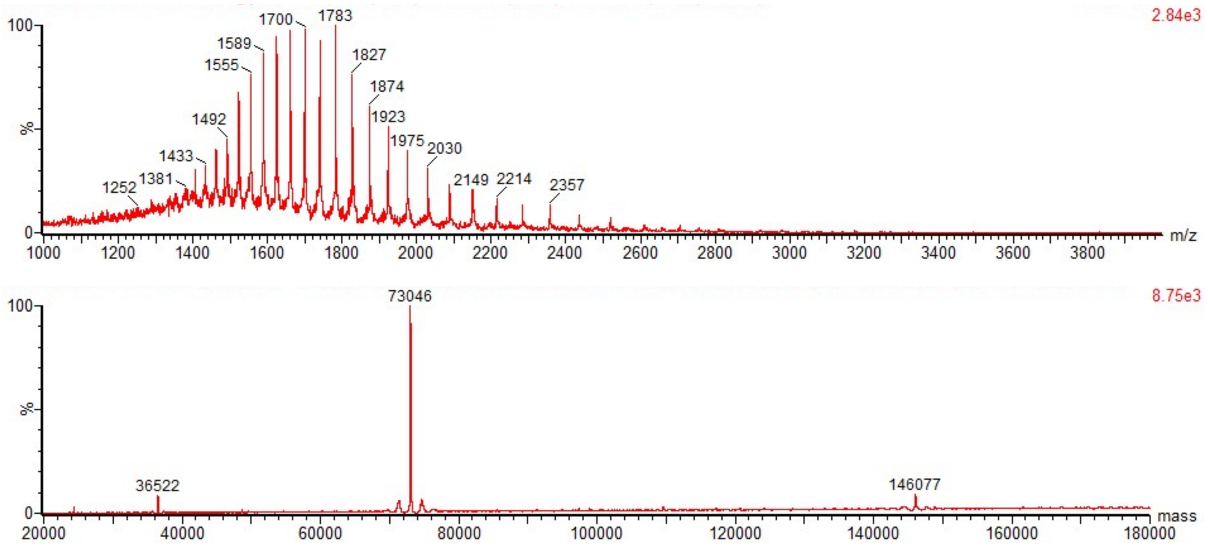

Figure S2. Analysis of the reaction between trastuzumab and **1** by LC-MS. Top = non-deconvoluted MS. Bottom = deconvoluted MS; expected 73,061 Da (half antibody) and 146,123 Da (full antibody), observed 73,046 and 146,077 Da. Mass differences of up to 50 Da are within the normal expected range for proteins of this size under ESI ionisation conditions. Peak intensities are not representative of abundance.

Reaction of trastuzumab with BisDVP 2

To a solution of trastuzumab (11.9 µL, 17 µM, 2.5 mg/mL) in TBS (25 mM Tris HCl pH 8, 25 mM NaCl, 0.5 mM EDTA) was added TCEP (10 equiv.). The mixture was vortexed and incubated at 37 °C for 1 h. A solution of **2** (10 mM in DMSO) was added (final concentration of 170 µM, 10 equiv.) and the reaction mixture incubated at 37 °C for 4 h. The excess reagents were removed by use of a Zeba™ Spin Desalting Column (7,000 MWCO, Thermo Fisher Scientific), followed by repeated diafiltration into PBS using an Amicon-Ultra centrifugal filter (10,000 MWCO, Merck Millipore). LC-MS and SDS-PAGE analysis demonstrated >95% conversion to the bridged conjugate.

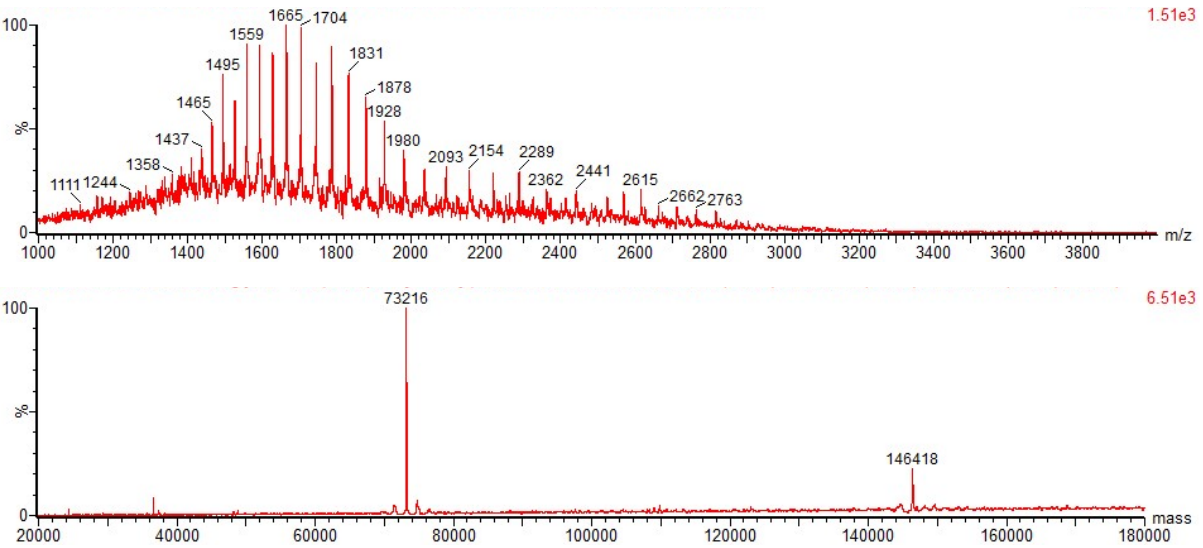

Figure S3. Analysis of the reaction between trastuzumab and **2** by LC-MS. Top = non-deconvoluted MS. Bottom = deconvoluted MS; expected 73,224 Da (half antibody) and 146,448 Da (full antibody), observed 73,216 and 146,418 Da. Mass differences of up to 50 Da are within the normal expected range for proteins of this size under ESI ionisation conditions. Peak intensities are not representative of abundance.

Reaction of trastuzumab with TetraDVP 3

To a solution of trastuzumab (198 µL, 17 µM, 2.5 mg/mL) in TBS (25 mM Tris HCl pH 8, 25 mM NaCl, 0.5 mM EDTA) was added TCEP (10 equiv.). The mixture was vortexed and incubated at 37 °C for 1 h. A solution of **3** (10 mM in DMSO) was added (final concentration of 34 µM, 2 equiv.) and the reaction mixture incubated at 37 °C for 4 h. The excess reagents were removed by use of a Zeba™ Spin Desalting Column (40,000 MWCO, Thermo Fisher Scientific), followed by repeated diafiltration into PBS using an Amicon-Ultra centrifugal filter (10,000 MWCO, Merck Millipore). LC-MS and SDS-PAGE analysis demonstrated >95% conversion to the bridged conjugate.

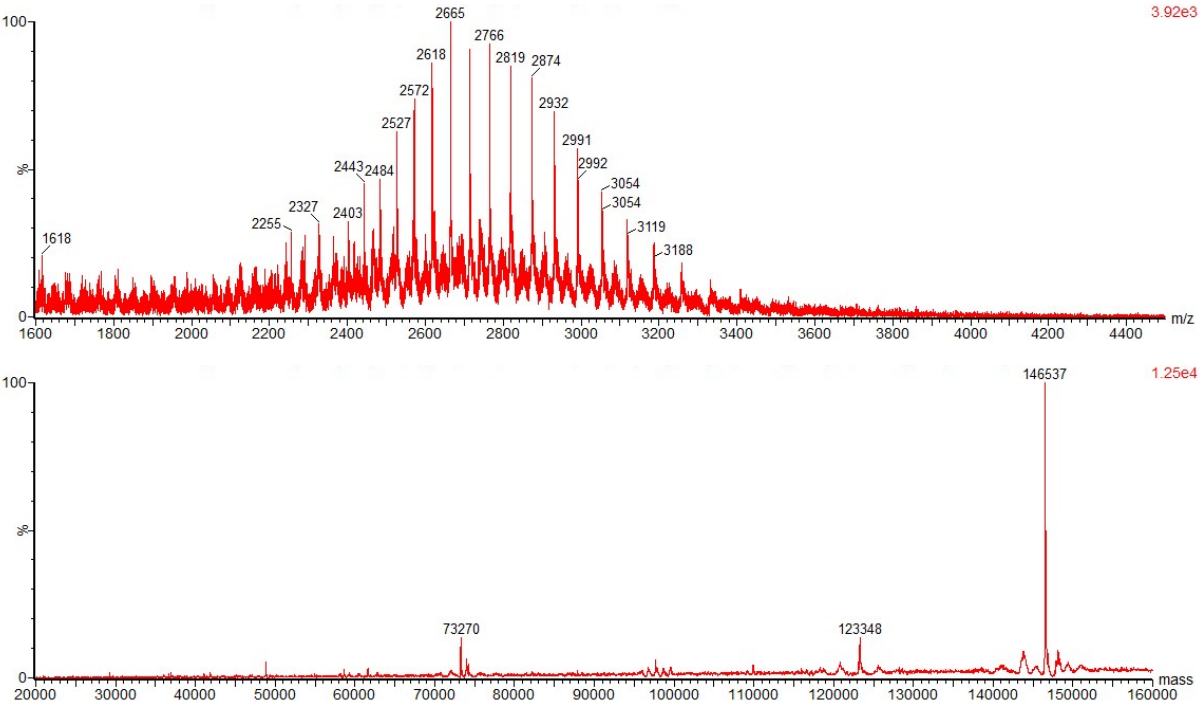

Figure S4. Analysis of the reaction between trastuzumab and **3** by LC-MS. Top = non-deconvoluted MS. Bottom = deconvoluted MS; expected 146,536 Da, observed 146,537 Da. Mass differences of up to 50 Da are within the normal expected range for proteins of this size under ESI ionisation conditions. The peaks at 73,270 Da and 123,348 Da may correspond to partially rebridged antibody species. Peak intensities are not representative of abundance. (Smaller proteins tend to ionise more strongly than big proteins under ESI ionisation conditions and thus appear as more abundant than they are.)

Optimisation of trastuzumab conjugation to TetraDVP 3

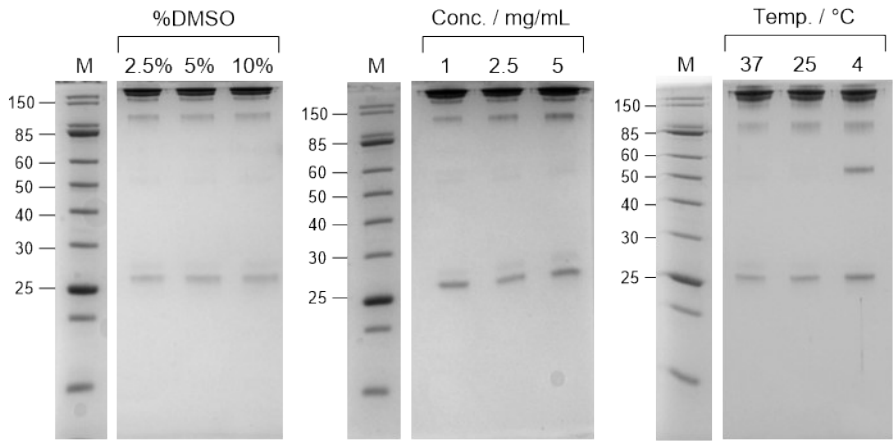

Figure S5. Screening of reaction conditions for the bioconjugation of trastuzumab with TetraDVP **3** by SDS-PAGE. Evaluated factors were amount of organic co-solvent (left), antibody concentration (middle) and reaction temperature (right); M = molecular weight marker.

Reaction of brentuximab with TetraDVP 3

To a solution of brentuximab (50 µL, 17 µM, 2.5 mg/mL) in TBS (25 mM Tris HCl pH 8, 25 mM NaCl, 0.5 mM EDTA) was added TCEP (10 equiv.). The mixture was vortexed and incubated at 37 °C for 1 h. A solution of **3** (10 mM in DMSO) was added (final concentration of 34 µM, 2 equiv.) and the reaction mixture incubated at 37 °C for 2 h. The excess reagents were removed by use of a Zeba™ Spin Desalting Column (40,000 MWCO, Thermo Fisher Scientific), followed by repeated diafiltration into PBS using an Amicon-Ultra centrifugal filter (10,000 MWCO, Merck Millipore). LC-MS and SDS-PAGE analysis demonstrated >95% conversion to the bridged conjugate.

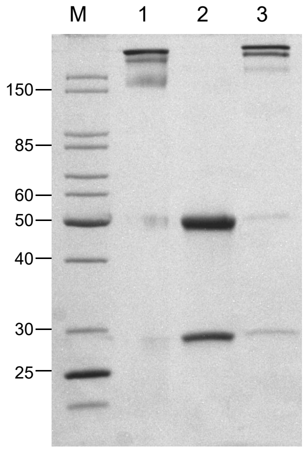

Figure S6. Analysis of the bioconjugation of brentuximab with TetraDVP **3** by SDS-PAGE. Lanes: M = molecular weight marker, 1 = brentuximab, 2 = brentuximab (reduced), 3 = reaction of brentuximab with **3**.

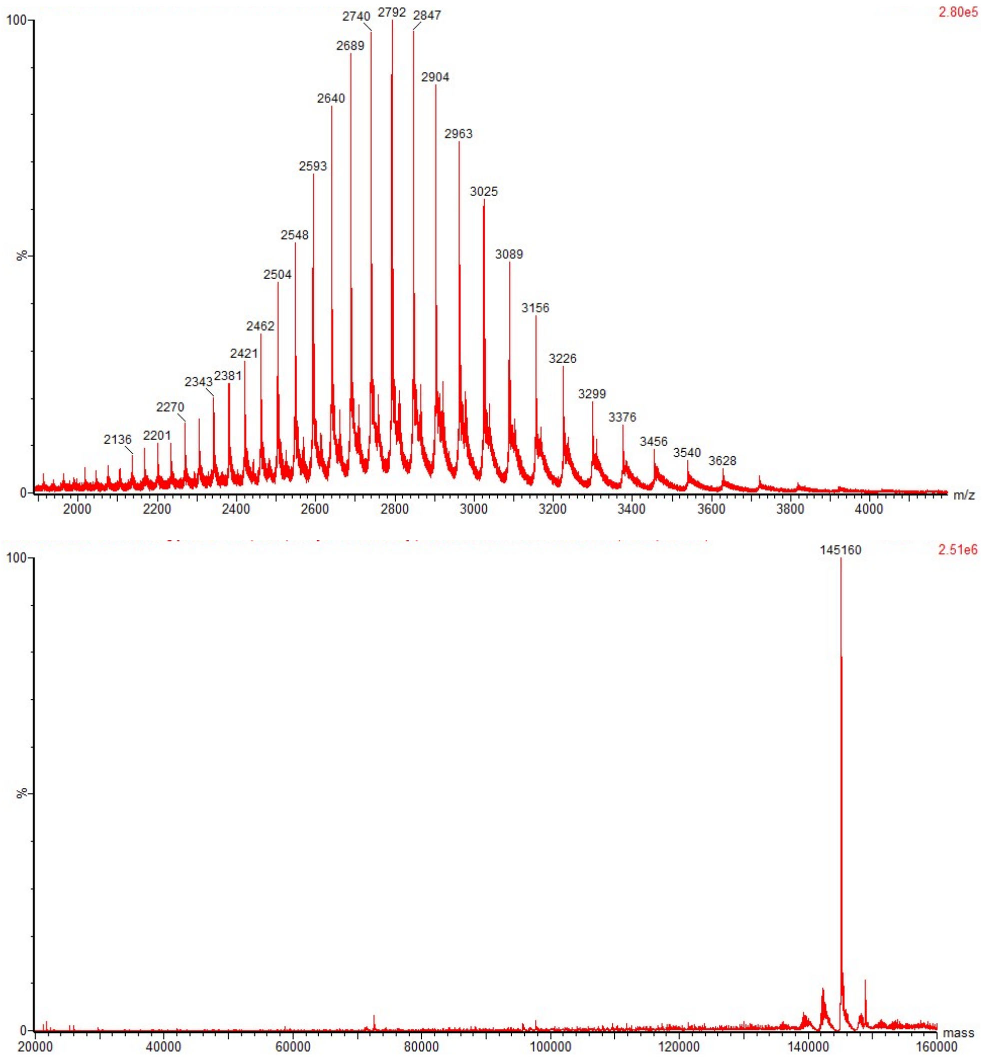

Figure S7. LC-MS of unmodified brentuximab. Top = non-deconvoluted MS. Bottom = deconvoluted MS; expected 145,203 Da, observed 145,160 Da. Mass differences of up to 50 Da are within the normal expected range for proteins of this size under ESI ionisation conditions. Peak intensities are not representative of abundance.

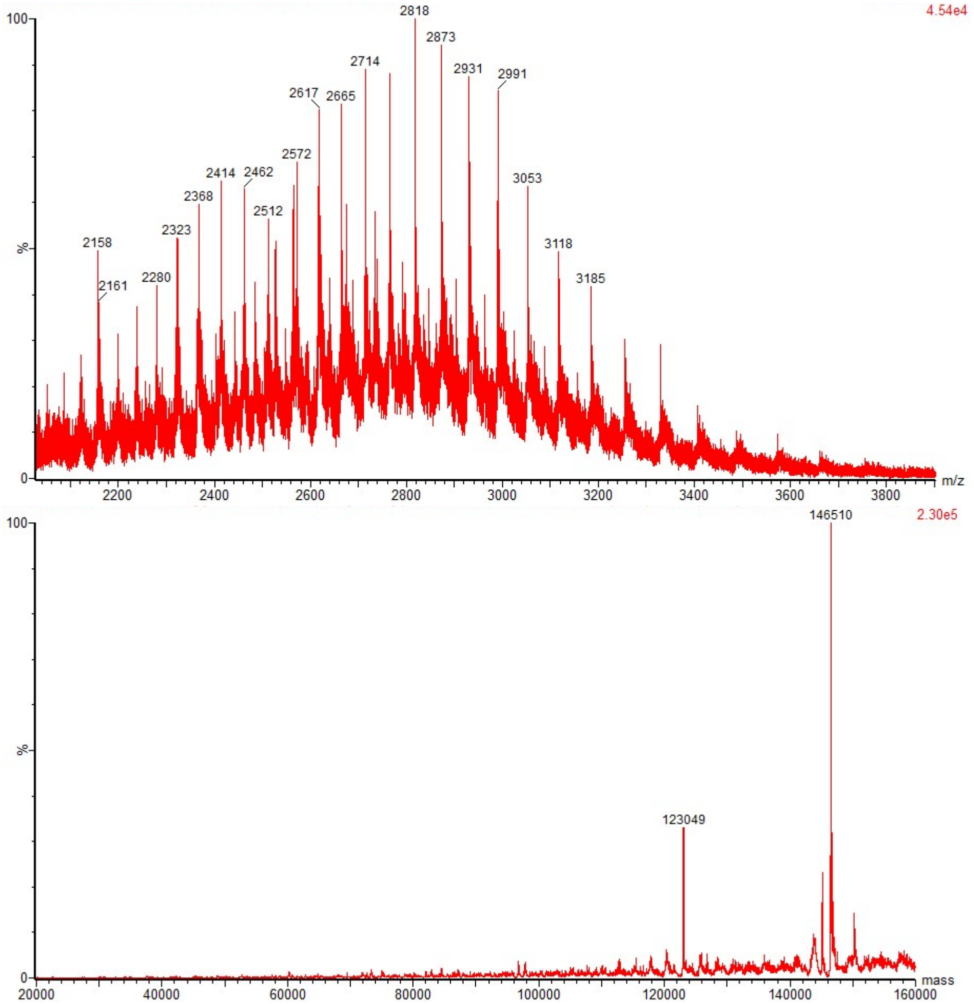

Figure S8. Analysis of the reaction between brentuximab and **3** by LC-MS. Top = non-deconvoluted MS. Bottom = deconvoluted MS; expected 146,549 Da, observed 146,510 Da. Mass differences of up to 50 Da are within the normal expected range for proteins of this size under ESI ionisation conditions. The peak at 123,049 Da may correspond to partially rebridged antibody species. Peak intensities are not representative of abundance. (Smaller proteins tend to ionise more strongly than big proteins under ESI ionisation conditions and thus appear as more abundant than they are.)

Reaction of trastuzumab with TetraDVP 7

To a solution of trastuzumab (198 µL, 17 µM, 2.5 mg/mL) in TBS (25 mM Tris HCl pH 8, 25 mM NaCl, 0.5 mM EDTA) was added TCEP (10 equiv.). The mixture was vortexed and incubated at 37 °C for 1 h. A solution of **7** (10 mM in DMSO) was added (final concentration of 34 µM, 2 equiv.) and the reaction mixture incubated at 37 °C for 4 h. The excess reagents were removed by use of a Zeba™ Spin Desalting Column (40,000 MWCO, Thermo Fisher Scientific), followed by repeated diafiltration into PBS using an Amicon-Ultra centrifugal filter (10,000 MWCO, Merck Millipore). LC-MS and SDS-PAGE analysis demonstrated >95% conversion to the bridged conjugate.

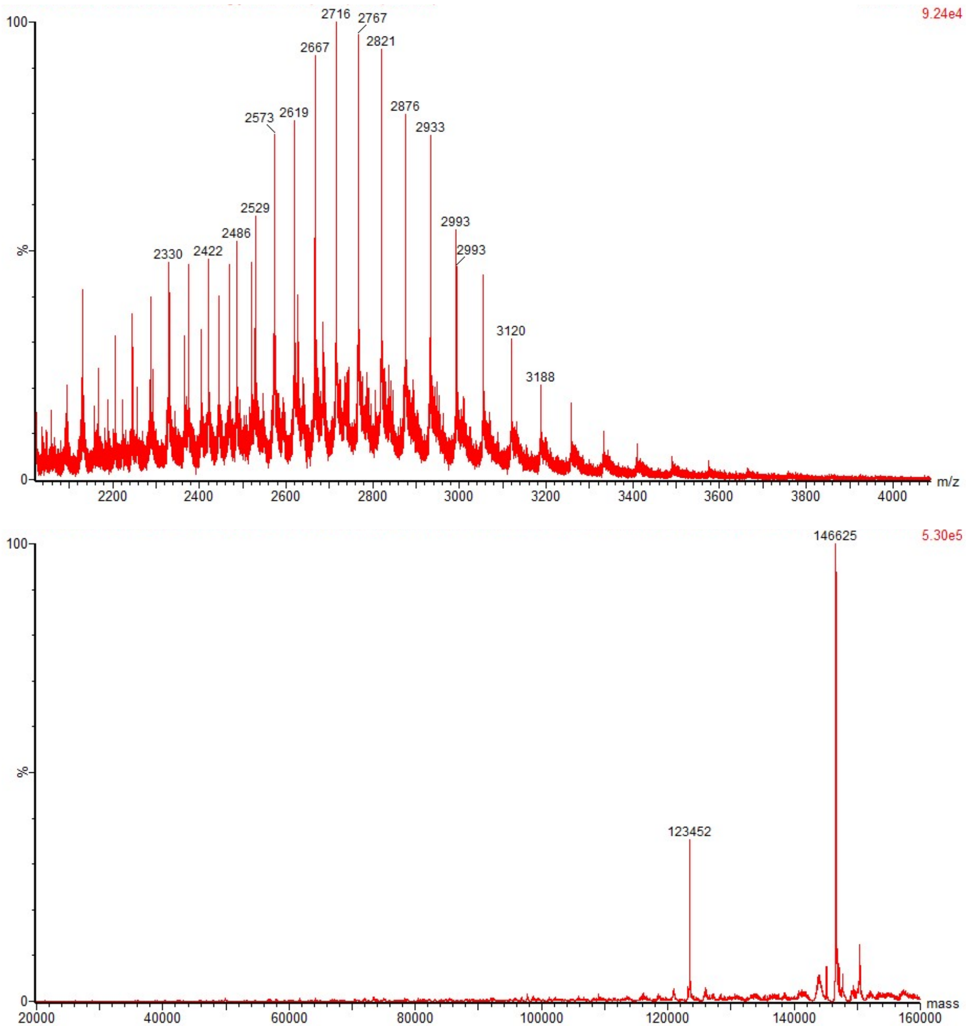

Figure S9. Analysis of the reaction between trastuzumab and **7** by LC-MS. Top = non-deconvoluted MS. Bottom = deconvoluted MS; expected 146,674 Da, observed 146,625 Da. Mass differences of up to 50 Da are within the normal expected range for proteins of this size under ESI ionisation conditions. The peak at 123,452 Da may correspond to partially rebridged antibody species. Peak intensities are not representative of abundance. (Smaller proteins tend to ionise more strongly than big proteins under ESI ionisation conditions and thus appear as more abundant than they are.)

Reaction of trastuzumab with TetraDVP 8

To a solution of trastuzumab (198 µL, 17 µM, 2.5 mg/mL) in TBS (25 mM Tris HCl pH 8, 25 mM NaCl, 0.5 mM EDTA) was added TCEP (10 equiv.). The mixture was vortexed and incubated at 37 °C for 1 h. A solution of **8** (10 mM in DMSO) was added (final concentration of 34 µM, 2 equiv.) and the reaction mixture incubated at 37 °C for 4 h. The excess reagents were removed by use of a Zeba™ Spin Desalting Column (40,000 MWCO, Thermo Fisher Scientific), followed by repeated diafiltration into PBS using an Amicon-Ultra centrifugal filter (10,000 MWCO, Merck Millipore). LC-MS and SDS-PAGE analysis demonstrated >95% conversion to the bridged conjugate.

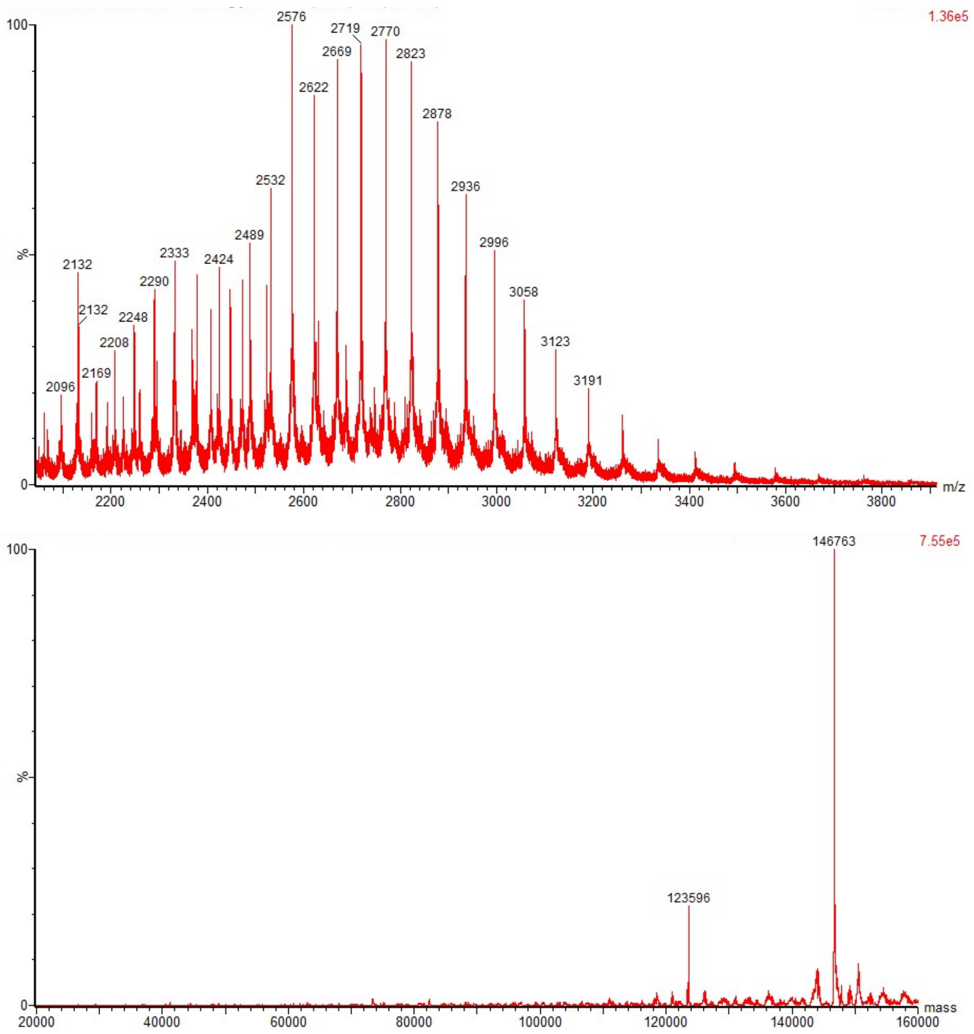

Figure S10. Analysis of the reaction between trastuzumab and **8** by LC-MS. Top = non-deconvoluted MS. Bottom = deconvoluted MS; expected 146,812 Da, observed 146,763 Da. Mass differences of up to 50 Da are within the normal expected range for proteins of this size under ESI ionisation conditions. The peak at 123,596 Da may correspond to partially rebridged antibody species. Peak intensities are not representative of abundance. (Smaller proteins tend to ionise more strongly than big proteins under ESI ionisation conditions and thus appear as more abundant than they are.)

Reaction of trastuzumab with TetraDVP 9

To a solution of trastuzumab (198 µL, 17 µM, 2.5 mg/mL) in TBS (25 mM Tris HCl pH 8, 25 mM NaCl, 0.5 mM EDTA) was added TCEP (10 equiv.). The mixture was vortexed and incubated at 37 °C for 1 h. A solution of **9** (10 mM in DMSO) was added (final concentration of 34 µM, 2 equiv.) and the reaction mixture incubated at 37 °C for 4 h. The excess reagents were removed by use of a Zeba™ Spin Desalting Column (40,000 MWCO, Thermo Fisher Scientific), followed by repeated diafiltration into PBS using an Amicon-Ultra centrifugal filter (10,000 MWCO, Merck Millipore). LC-MS and SDS-PAGE analysis demonstrated >95% conversion to the bridged conjugate.

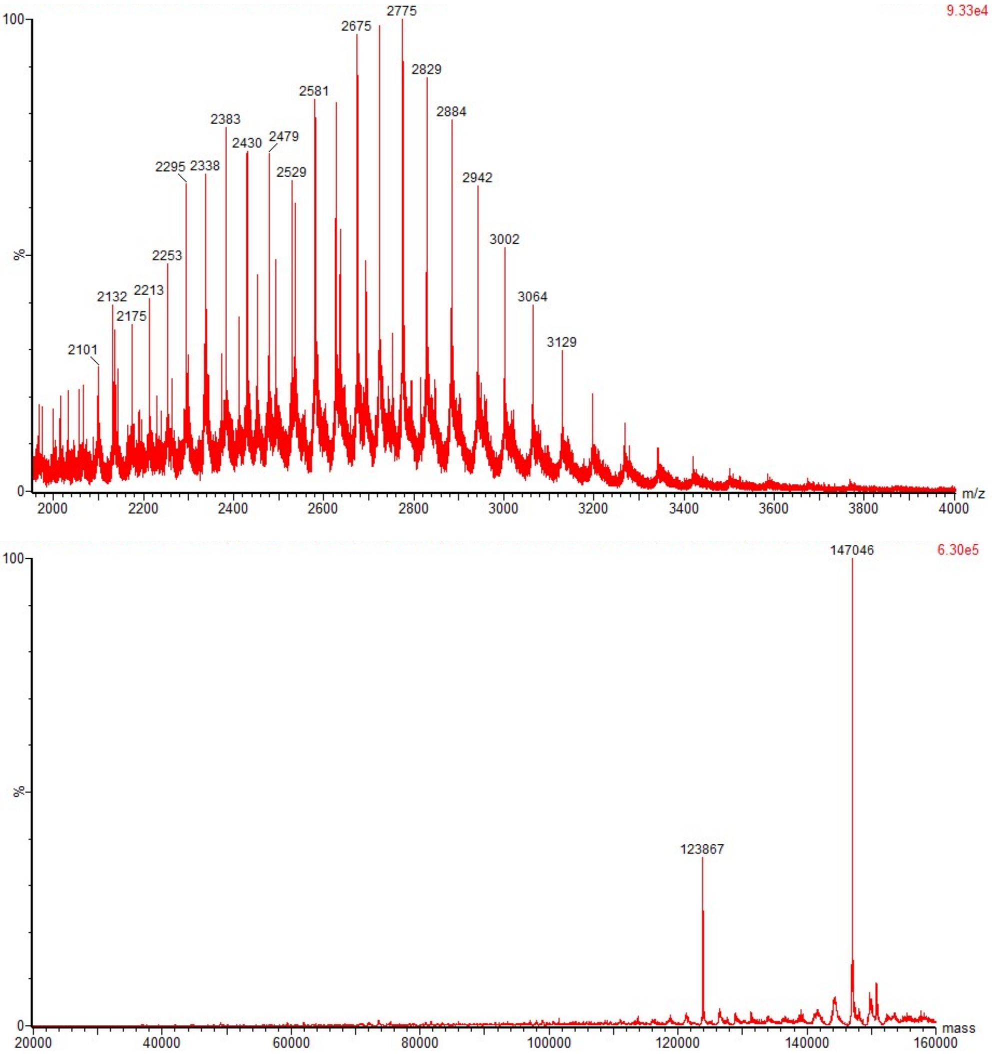

Figure S11. Analysis of the reaction between trastuzumab and **9** by LC-MS. Top = non-deconvoluted MS. Bottom = deconvoluted MS; expected 147,096 Da, observed 147,046 Da. Mass differences of up to 50 Da are within the normal expected range for proteins of this size under ESI ionisation conditions. The peak at 123,867 Da may correspond to partially rebridged antibody species. Peak intensities are not representative of abundance. (Smaller proteins tend to ionise more strongly than big proteins under ESI ionisation conditions and thus appear as more abundant than they are.)

Thermal stability

The thermal denaturation of proteins was monitored by differential scanning calorimetry (DSC), with a Malvern MicroCal VP-DSC instrument. 500 µL of protein at 0.5 mg mL^{-1} in 50 mM Tris pH 7.5 were used for each run. For each protein, the baseline was measured first, which consists of buffer in both cells (buffer versus buffer), followed by protein (buffer versus protein). Several clean-up cycles with water and suitability controls with lysozyme at 3 mg/mL in water were employed before, and after, the antibody experiment. Each protein sample was scanned twice to investigate the thermal reversibility. The temperature was ramped from 25 to 100 °C, increasing by 95 °C h^{-1}. The pre-scan thermostat was set to 2 min, no post-scan thermostat was employed. The data were processed with the Origin version 7.0 SR4 software. The baseline thermogram (buffer versus buffer) was subtracted from the thermogram of the protein (buffer versus protein). Two baselines, one at the beginning and one at the end of the thermogram, were placed to adjust the data, which was then normalized using the concentration of the protein. The unfolding peaks were selected and the thermogram was fitted to the "Non 2-state" model to obtain the melting temperatures (T_m) and the enthalpy of unfolding at the T_m, ΔH_{cal}.

Chemical denaturation experiments

Generation of F(ab')$_2$ and Fc fragments from full length IgG

Unmodified trastuzumab and TetraDVP-modified trastuzumab (**6**) were digested with IdeS (FabRICATOR, Genovis), which cuts below the hinge (CPAPELLG / GPSVF), with a 1 unit : 20 µg ratio overnight at 37 °C. This generated F(ab')$_2$ (two arms and hinge) and Fc fragments (without hinge) were separated by size-exclusion chromatography run in 50 mM Tris pH 7.5 (Superdex G75 30/100 GL and Superdex 200 30/100 GL, GE Healthcare).

Thermodynamic stability

The thermodynamic stability of conjugation products was probed by chemical denaturation curves monitored by tryptophan fluorescence. The thermodynamic stability of each domain was determined for unmodified trastuzumab, TetraDVP conjugate **6**, DVP conjugate **4**, trastuzumab F(ab')$_2$ and TetraDVP-modified F(ab')$_2$ (produced as detailed above) by measuring the change in tryptophan fluorescence after incubation in various concentrations of guanidinium chloride (GdmCl). Each experiment was composed of forty-one points (120 µL total volume per point) of increasing concentrations of GdmCl from 0 to 3.7 M final. 110 µL of denaturant solution was mixed with 10 µL of protein to a final concentration of 1 µM. For

the unfolding curves, the stock protein solution was made in 50 mM Tris pH 7.5. The solutions were dispensed with a liquid handling robot (Microlab®500 Series, ML541C, Hamilton Company). The denaturant solutions mixed with the protein were incubated at 25 °C at different time points until they reached equilibrium (7 days). Each of the forty-one denaturation points were measured in a 100 µL quartz cuvette (Hellma, Precision Cell in Quartz SUPRASIL®, Typ No: 105.250-QS, light path: 10x2 mm, centre: 20 mm). The fluorescence was recorded with a Cary 400 Eclipse Fluorescence Spectrophotometer (Agilent Technologies) thermostatted at 25 °C controlled by a heat block. The samples were excited at 280 nm, the emission was recorded from 300 to 400 nm, with a scan rate of 300 nm/min, excitation and emission band passes were set at 10 nm. The data were analyzed using an average emission wavelength (AEW), which is the arithmetic mean of the wavelengths weighted by the fluorescence intensity at each wavelength. It is calculated as shown in **Equation 1.1.**

$$AEW = \frac{\sum_{i=1}^{N} F_i . \lambda_i}{\sum_{i=1}^{N} F_i}$$ **Eq. 1.1**

where F_i is the intensity of fluorescence at the wavelength i, and λ_i the wavelength.

The trastuzumab $F(ab)_2$ data were then fitted with a two-state model (where only the native and denatured state are significantly populated):

$$F = \frac{(\alpha_N + \beta_N.[den]) + (\alpha_D + \beta_D.[den]).\exp\left(\frac{m_{D-N}}{RT}.([den] - [den]_{50\%})\right)}{1 + \exp\left(\frac{m_{D-N}}{RT}.([den] - [den]_{50\%})\right)}$$ **Eq. 1.2**

where α_N, α_D are the fluorescence of the native and denatured states in H_2O respectively; β_N, β_D are the slopes of native and denatured baselines respectively; m_{D-N} is the m-value between the denatured and native state; ΔG_{D-N}^{H2O} is the difference in Gibbs free energy between the denatured and native states; T the temperature and R the gas constant.

The unmodified, DVP-modified and TetraDVP-modified trastuzumab, and TetraDVP-modified $F(ab)_2$ were fitted to a three-state model (in which an intermediate state between the native and denatured state is sufficiently stable to be populated and observed)[7]:

$$F = \frac{\alpha_N + \beta_N.[den] + \alpha_I.\exp\left(\frac{m_{I-N}}{RT}.([den] - [den]_{50\%\,I-N})\right) + (\alpha_D + \beta_D.[den])\exp\left(\frac{m_{I-N}}{RT}.([den] - [den]_{50\%\,I-N})\right)\exp\left(\frac{m_{D-I}}{RT}.([den] - [den]_{50\%\,D-I})\right)}{1 + \exp\left(\frac{m_{I-N}}{RT}.([den] - [den]_{50\%\,I-N})\right) + \exp\left(\frac{m_{I-N}}{RT}.([den] - [den]_{50\%\,I-N})\right)\exp\left(\frac{m_{D-I}}{RT}.([den] - [den]_{50\%\,D-I})\right)}$$

Eq. 1.3

where $\alpha_N, \alpha_I, \alpha_D$ are the fluorescence of the native, intermediate and denatured states in H_2O respectively; β_N, β_D are the slopes of native and denatured baselines respectively; m_{I-N}, m_{D-I} are the m-values between the intermediate and native state, and denatured and intermediate states respectively; $\Delta G^{H2O}_{I-N}, \Delta G^{H2O}_{D-I}, \Delta G^{H2O}_{D-N}$ are the differences in Gibbs free energy between the intermediate and native states, denatured and intermediate states, and denatured and native states, respectively; T the temperature and R the gas constant.

The following equation can be used normalize the native and denatured baselines:

$$Norm = \frac{(AEW - (\alpha_N + \beta_N.[den])}{(\alpha_D + \beta_D.[den]) - (\alpha_N + \beta_N.[den])}$$

Eq. 1.4

The chemical denaturation curves of the full-length trastuzumab, TetraDVP conjugate **6** and DVP conjugate **4** clearly show that the additional multivalent linker has destabilised the protein, the modified variants unfolding at lower GdmCl concentrations than unmodified trastuzumab (Figure S12A). This is reflected in, and can be attributed to, the destabilization of the native state of the Fab fragment and the C_H2 domain on conjugation (Figure S12A-B). For the Fab fragment, an additional unfolding transition appeared at a lower concentration of GdmCl whilst the other unfolding transition at higher GdmCl concentrations remained unchanged as shown by the $[den]_{50\%}$ and the m-value which are within error of the values calculated for the single transition observed with the non-modified Fab (Figure S12C). Given that the TetraDVP is covalently linked to the four canonical cysteines in the hinge and also to the two cysteines in each Fab arm originally forming the intrachain disulfide bridge between the heavy and light chains, it is unlikely that the linker has affected the stability of the variable domains which are distant from the conjugation sites. Thus, these data suggest that the TetraDVP linker has destabilised the constant domains within the Fab, i.e. the C_L and C_H1 domains.

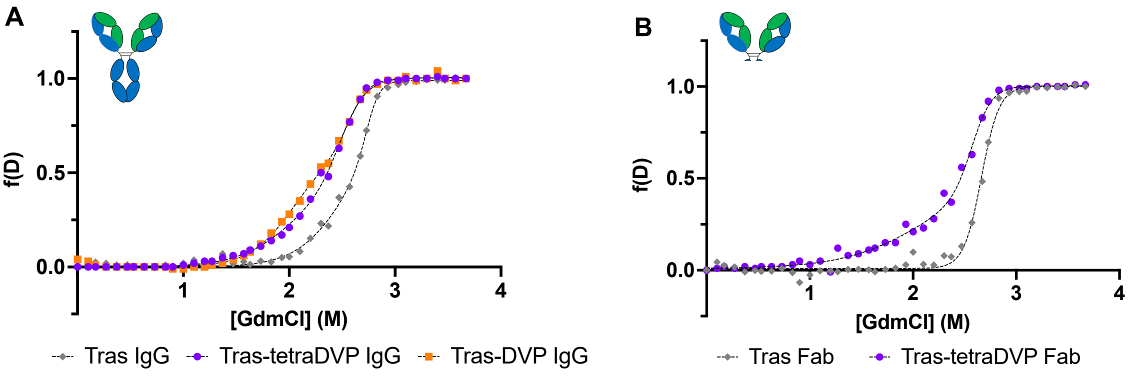

C

	m_{I-N} (kcal mol^{-1} M^{-1})	$[den]_{50\%\ I-N}$ (M)	m_{D-I} (kcal mol^{-1} M^{-1})	$[den]_{50\%\ D-I}$ (M)
Tras IgG	2.5 ± 0.5	2.5 ± 0.2	8.6 ± 1.6	2.73 ± 0.03
Tras-tetraDVP IgG	2.3 ± 0.6	2.0 ± 0.2	4.6 ± 0.4	2.49 ± 0.03
Tras-DVP IgG	2.9 ± 0.3	2.03 ± 0.05	5.9 ± 0.8	2.57 ± 0.03
Tras Fab	-	-	6.9 ± 0.5	2.66 ± 0.01
Tras-tetraDVP Fab	1.5 ± 0.6	2.1 ± 0.5	5.8 ± 0.8	2.56 ± 0.02

Figure S12. Chemical denaturation curves of unmodified and modified trastuzumab. Trastuzumab (grey lozenges), TetraDVP-linked trastuzumab (purple circles), DVP-linked trastuzumab (orange squares). (A) Full-length IgG. (B) Fab domain with hinge (two arms). The samples were denatured in guanidinium chloride, 50 mM Tris pH 7.5, at 25 °C until equilibrium was reached (at least 5 days). (C) Thermodynamic parameters fitted from the unfolding curves: three thermodynamic parameters were obtained: $\Delta G_{X-Y}^{H_2O}$, the difference in Gibbs free energy between two states X and Y in water; m_{X-Y}, the m-value between the two states X and Y, a constant of proportionality describing how much ΔG_{X-Y} changes with denaturant concentration; and $[den]_{50\%\ X-Y}$, the midpoint of denaturation between states X and Y. The errors reported in this table are fitting errors.

Kinetic stability

Unfolding kinetics provide information on the rate at which a native state of a domain unfolds. Native, folded protein was rapidly diluted into a range of chemical denaturant concentrations and the protein unfolding was monitored by the change in intrinsic fluorescence on a stopped-flow spectrophotometer. For these stopped-flow experiments, the native protein and the denaturant solutions were mixed in a 1:10 ratio respectively. Seven stock solutions of guanidinium chloride (GdmCl) were prepared in 50 mM Tris pH 7.5 so that the final concentrations range from 5.5 to 7.0 M GdmCl with an interval of 0.25 M. The stock solutions were 1.1-fold more concentrated than the final solutions, as solutions were diluted by a factor of 10/11 in the rapid mixing step, using 500 µL and 2.5 mL Hamilton syringes. Protein stock solutions of trastuzumab, DVP conjugate **4**, TetraDVP conjugate **6**, trastuzumab Fc, trastuzumab F(ab')$_2$ and TetraDVP-modified F(ab')$_2$ were prepared between 7 and 11 µM to achieve a final concentration between 0.5 to 1 µM after mixing with the denaturant solutions. The unfolding kinetics were monitored with a SX20 stopped-flow spectrometer from Applied Photophysics (software: SX Spectrometer Control Panel Application version 2.2.27). The

temperature of the water bath was set to 25 °C, the excitation wavelength was set to 280 nm, both slit widths were 2 mm. A cut off filter of 320 nm was used. Three longer time traces (30 to 300s sec long depending on the unfolding rates) were acquired to measure the unfolding phases, both at each denaturant concentration.

The unfolding curves were fitted with the software Pro DataViewer version 4.2.27. Depending on the recorded traces and the number of domains unfolding, the fluorescence signal corresponding to the unfolding was fitted with a single (**Equation 2.1**), double-exponential (**Equation 2.2**) or triple-exponential function (**Equation 2.3**).

$$A(t) = A.\exp(-kt) + c \qquad \textbf{Eq. 2.1}$$

$$A(t) = A_1.\exp(-k_1 t) + A_2.\exp(-k_2 t) + c \qquad \textbf{Eq. 2.2}$$

$$A(t) = A_1.\exp(-k_1 t) + A_2.\exp(-k_2 t) + A_3.\exp(-k_3 t) + c \qquad \textbf{Eq. 2.3}$$

where A_1 , A_2 and A_3 are the amplitudes, k_1 ,k_2 and k_3 the respective unfolding rate constants and c the offset.

The natural logarithm of the rate constants was then calculated and plotted versus the corresponding denaturant concentration, and the data fitted with **Equation 2.4**.[8]

$$\ln k_U^{[den]} = \ln k_U^{H_2O} + m_{k_U}[den] \qquad \textbf{Eq. 2.4}$$

Where $k_U^{[den]}$ is the observed unfolding rate constant at the denaturant concentration $[den]$, $k_U^{H_2O}$ is the unfolding rate constant in water and m_{k_U} is the slope of the plot of ln$k_U^{[den]}$ versus denaturant concentration.

Unmodified trastuzumab, TetraDVP conjugate **6** and DVP conjugate **4** (full-length IgGs) each show two unfolding phases at most of the GdmCl concentrations used. The kinetic parameters obtained from the fit of the data to **Equation 2.4** show that the unfolding rate constants of the Fab domain and the fastest of the two phases observed for the full-length IgGs as well as the C_H3 domain and the slowest of the two phases of the full-length IgGs are within error (Figure S13 and Table S1). Overall, the unfolding kinetics data show that the modifications do not cause any significant change in kinetic stability.

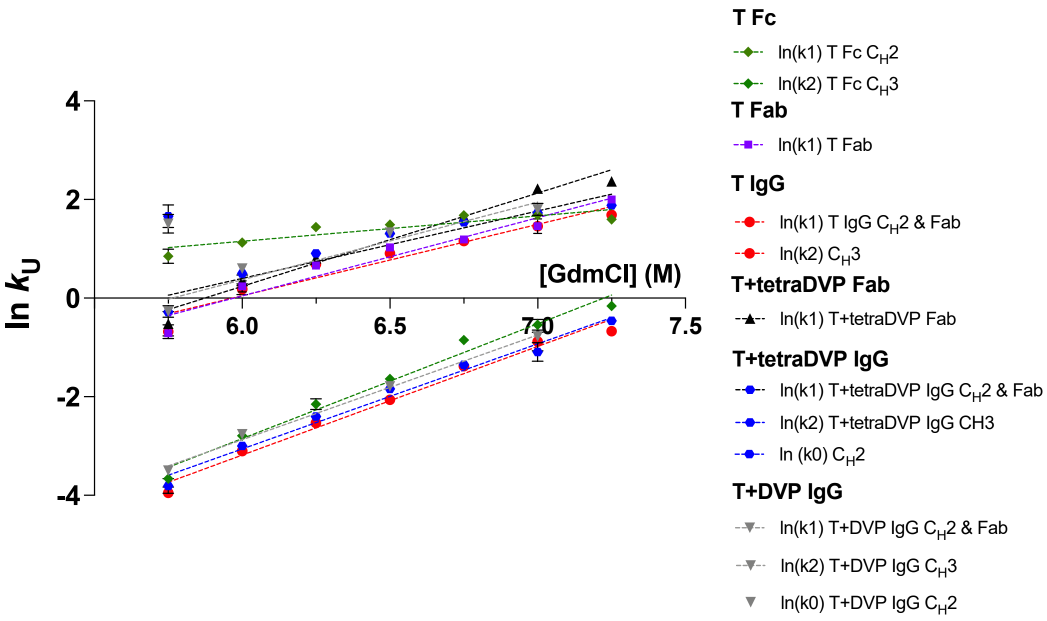

Figure S13. Unfolding kinetics of unmodified and modified forms of trastuzumab. Trastuzumab Fc (green circles), trastuzumab F(ab')$_2$ (purple squares), trastuzumab IgG (red circles), TetraDVP-modified trastuzumab F(ab')$_2$ (black triangles), TetraDVP-modified trastuzumab IgG (conjugate **6**, blue circles), DVP-modified trastuzumab IgG (conjugate **4**, grey triangles). The dotted lines show the best fit of the data to **Equation 2.4**. Error bars represent the standard deviation from triplicate measurements. In several instances, error bars are not visible because they are smaller than the size of the data points.

Table S1: Unfolding kinetic parameters

		m_{kU} (M^{-1})	ln k_U (H_2O)
T Fc	ln(k1) T Fc C_H2	0.5 ± 0.1	-1.9 ± 0.8
	ln(k2) T Fc C_H3	2.3 ± 0.1	-16.8 ± 0.9
T Fab	ln(k1) T Fab	1.6 ± 0.2	-9.5 ± 1.2
T IgG	ln(k1) T IgG C_H2 & Fab	1.4 ± 0.2	-8.6 ± 1.2
	ln(k2) T IgG C_H3	2.2 ± 0.1	-16.4 ± 0.9
T+tetraDVP Fab	ln(k1) T+tetraDVP Fab	1.9 ± 0.2	-11.1 ± 1.0
T+tetraDVP IgG (**6**)	ln(k1) T+tetraDVP IgG C_H2 & Fab	1.4 ± 0.2	-7.8 ± 1.1
	ln(k2) T+tetraDVP IgG C_H3	2.1 ± 0.1	-15.8 ± 0.8
T+DVP IgG (**4**)	ln(k1) T+DVP IgG C_H2 & Fab	1.6 ± 0.3	-9.1 ± 1.9
	ln(k_2) T+DVP IgG C_H3	2.1 ± 0.1	-15.6 ± 0.7

Biolayer interferometry (BLI)

Binding assays were performed on an OctetRED384 (ForteBio). All samples were prepared in DPBS (Sigma-Aldrich) with 0.1 % BSA (bovine serum albumin solution, 30% BSA in DPBS, sterile-filtered, BioXtra, Sigma-Aldrich) and 0.02% Tween 20 (TWEEN® 20 for molecular biology, Sigma-Aldrich). Serial dilutions were prepared for HER2: 0.9, 1.7, 3.4, 6.9 nM. Anti-human IgG Fc Capture (AHC) Biosensors (ForteBio) were used to immobilise trastuzumab conjugates at 32 nM. Before starting the BLI experiment, biosensors were incubated in DPBS with 0.1% BSA and 0.02% Tween 20 for 10 min. For the measurements, the biosensors were first incubated for one minute in DPBS with 0.1% BSA and 0.02% Tween 20 for the first baseline. Trastuzumab was then loaded onto the biosensors until reaching a displacement of 0.8-1 nm. The biosensors were then incubated in DPBS with 0.1% BSA and 0.02% Tween 20 for the second baseline for one minute, and subsequently moved to wells containing the serial dilutions of HER2 for the association step, and finally moved to DPBS with 0.1% BSA and 0.02% Tween 20 for the dissociation step. The association and dissociation results were fit to a 1:1 model using a global fitting.

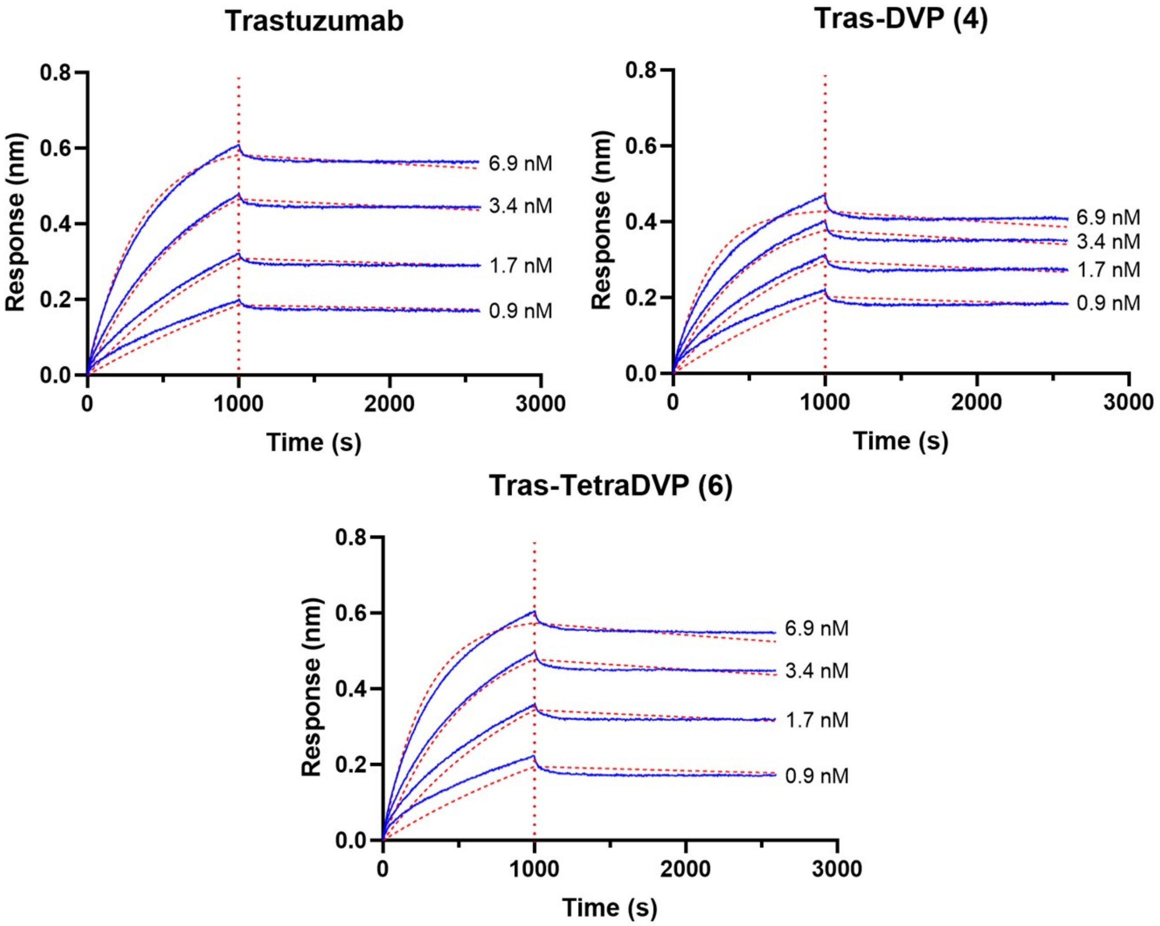

	K_D (M)	k_{on} (M^{-1} s^{-1})	k_{off} (s^{-1})
**Tras**	(9.0 ± 0.2) x 10^{-11}	(4.46 ± 0.02) x 10^{5}	(3.99 ± 0.07) x 10^{-5}
**Tras-DVP**	(1.06 ± 0.01) x 10^{-10}	(6.12 ± 0.04) x 10^{5}	(6.5 ± 0.1) x 10^{-5}
**Tras-TetraDVP**	(1.05 ± 0.02) x 10^{-10}	(5.35 ± 0.03) x 10^{5}	(5.6 ± 0.1) x 10^{-5}

Figure S14. Biolayer Interferometry (BLI) curves (in blue) and fitting curves (in red) obtained for trastuzumab, Tras-DVP conjugate **4** and Tras-TetraDVP conjugate **6** with HER2 receptor, together with the K_D constants derived from BLI experiments. Reported error values are fitting errors.

Hydrogen-deuterium exchange mass spectrometry (HDX-MS)

The HDX-MS experiments were conducted on full-length IgGs, unmodified, DVP and tetraDVP linked trastuzumab. For this experiment only, the unmodified trastuzumab was generated at AstraZeneca (expressed in CHO cells and purified on mAbSelect Sure columns, Cytiva – the validity of the use of this trastuzumab batch as the reference for the HDX-MS experiment was confirmed by the extremely high similarity of the deuterium exchange for peptides where no difference of exchange was observed between the unmodified, DVP and tetraDVP conjugated forms – see uptake plots). The peptide map was generated on trastuzumab in equilibration buffer 10 mM potassium phosphate pH 7.5 in H_2O, using a data-dependent acquisition (DDA) MS^2 approach. MS data were acquired using an Orbitrap Fusion (ThermoFisher) over a 300-2000 m/z range, using an AGC target of 200000 and a maximum injection time of 100 ms. MS^2 was acquired in the ion trap in centroid mode, using an AGC target of 10000 and a maximum injection time of 35 ms. Fragmentation was achieved by HCD using a collision energy of 30%. The labelled data were recorded after 50, 500 and 5000 s of incubation at 20 °C in deuterated buffer (10 mM potassium phosphate pD 7.5 in D_2O). On the Leap robot, 7 µL of 10 µM protein in equilibration buffer (10 mM potassium phosphate pH 7.5 in H_2O) was diluted seven-fold at 20 °C into equilibration or labelling buffer (10 mM potassium phosphate pD 7.5 in D_2O), to generate a peptide map or labelled data, respectively. The mixture was then diluted 1:1 with quench buffer at 4 °C (100 mM potassium phosphate, 8 M urea, 0.5 M TCEP, pH 2.5). The quench solution was then injected into a Waters nanoAcquity UPLC system, flowing for four min at 400 µL/min onto the pepsin column at 20 °C for digestion (Waters Enzymate™ BEH Pepsin Column (2.1 x 30 mm, 5 µm)) to the trap (pushed by LC-MS grade H_2O with 0.2% formic acid, and then eluting from the C18 analytical column for 10 minutes, using a 5% to 40 % organic phase (ACN with 0.2% formic acid). The mass spectrometry data were acquired with a MS^1 method (300-2000 m/z range, AGC target of 200000 and maximum injection time of 100 ms). All experiments were run in triplicate.

The peptide map was generated using BioPharmaFinder from equilibration data acquired with a MS^2 method. The recognition of the peptides was based in the fragmented b and y ions, to ensure confidence in the identification, and around the N-linked glycosylation in the C_H2 domain, the peptides including the asparagine at position 297 (in Eu numbering) were expected to carry the most abundant glycosylation expressed in CHO cells, i.e. G0F. The peptides obtained were filtered by confidence score higher than 80%. The exported csv file with the peptides as well as that same non-deuterated data were imported into HDExaminer, to operate a second filtration of the peptides: only the charge state with the highest intensity from the peptide map data was kept per peptide for comparative accuracy between the charge states, selected according to the highest intensity, the sharpest extracted ion chromatogram. After the peptide pool was curated, the labelled data were added, and the D

incorporation per peptide data was then exported as a csv file. To identify which deuterium incorporations were significant and to observe the deuterium exchange for each time point separately and overall, the data processing method used was first described by Dobson, Devine, Phillips *et al.*, 2016[9]. Starting with a csv file containing the D incorporation data, this Matlab-coded method first assesses if the incorporation of deuterium per peptide is significant compared to the control sample (assessed by a t-test, if two-tailed p-value < 0.01), then sums the significant time points per peptide, subsequently converts the peptide D incorporation to amino-acid incorporation by dividing the D incorporation by the maximal number of D that can be exchanged per peptide, subtracting the D incorporation from the control and dividing the amino-acid incorporation by the redundancy and finally normalizing it. The output is the deuterium incorporation for each individual amino acid. The crystal structures of the Fc wild-type (PDB: 3AVE), Fab domain of trastuzumab (PDB: 1N8Z) were coloured with a red-white-blue scale according to the relative incorporation of deuterium per amino acid, red representing deprotection and blue corresponding to protection, compared to the reference (Figure 2C).

A

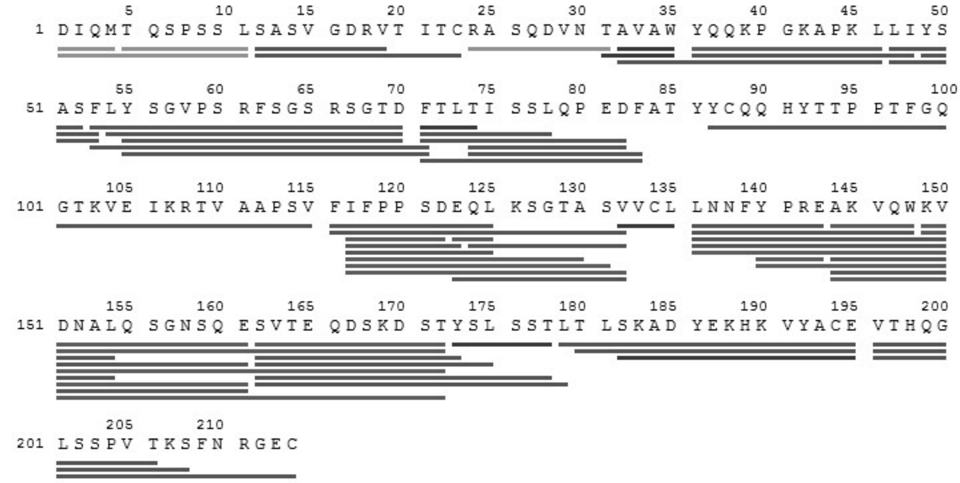

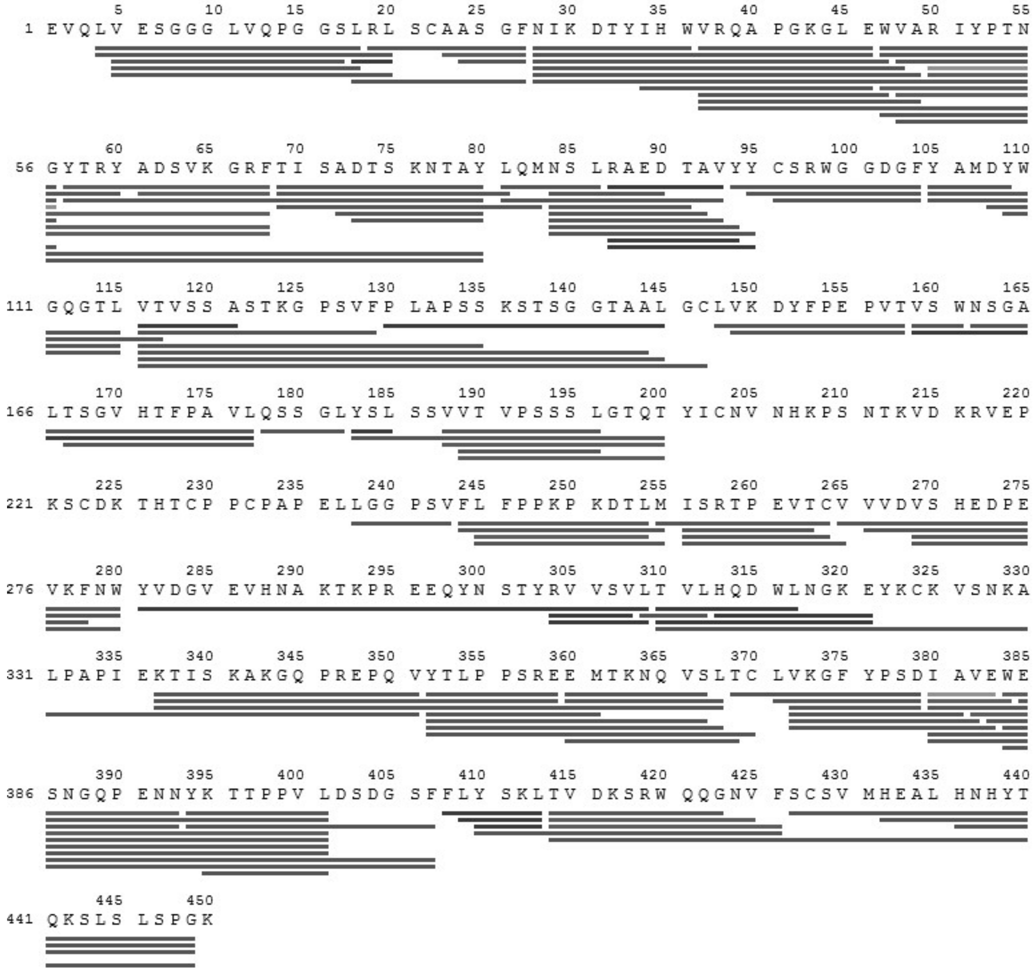

B

Figure S15. Peptide map of the (A) light chain and (B) heavy chain of trastuzumab.

Table S2. Summary of HDX mass spectrometry experimental details.

Dataset	Unmodified trastuzumab (10 µM) – trastuzumab expressed at AstraZeneca<br>DVP-linked trastuzumab (10 µM) – commercial trastuzumab<br>TetraDVP-linked trastuzumab (10 µM) – commercial trastuzumab
HDX reaction details	Equilibration in H_2O: 10 mM potassium phosphate pH 7.5<br>Labelling in D_2O: 10 mM potassium phosphate pD 7.5<br>Both were done at 20 °C<br>Quench buffer in H_2O: 100 mM potassium phosphate, 0.5 M TCEP, 8 M urea pH 2.5
HDX time course	50 s, 500 s, 5000 s
HDX controls	0 s
Number of peptides	LC: 63<br>HC: 140
Sequence coverage	LC: 98.6 %<br>HC: 90.9 %
Average peptide length	LC: 12.8 AA<br>HC: 13.4 AA
Replicates	Technical replicates: 3
Repeatability	Average Student's t-distribution 95% confidence interval for all peptides, all time points and states (#D error):<br>LC: 0.1389<br>HC: 0.1432
Significant differences in HDX	The incorporation of deuterium per peptide is significant compared to the wild-type if p-value < 0.01 (t-test)

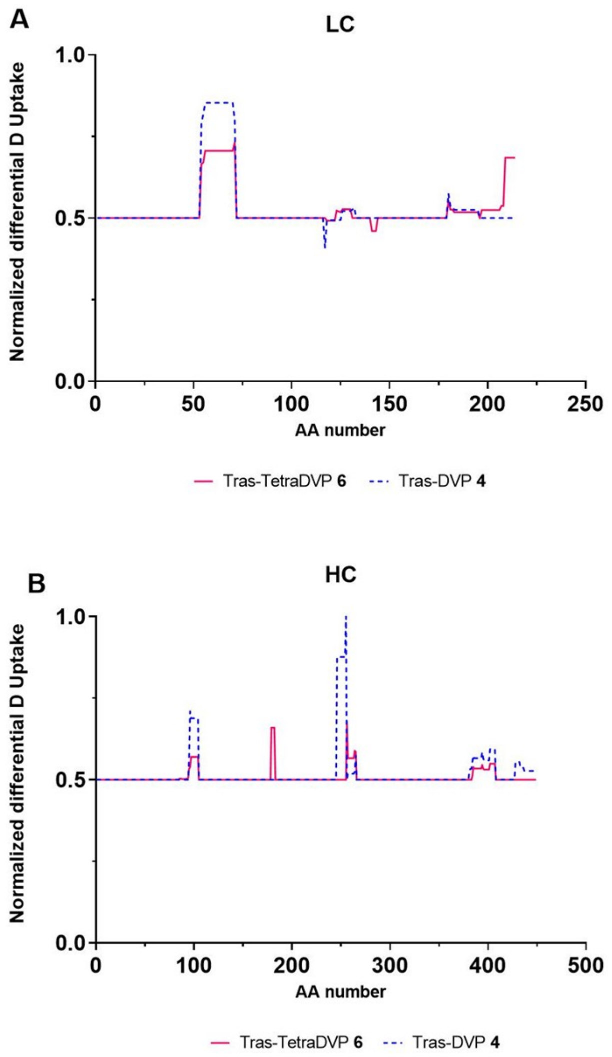

Figure S16. Difference plots showing the regions of trastuzumab that change dynamics after linkage with DVP **1** and TetraDVP **3**. Normalized difference plots of (A) the light chain and (B) the heavy chain, obtained by summing the significant D-incorporations relative to the reference, and subtracting the reference exchange to that of the Tras-TetraDVP and Tras-DVP. The light and heavy chains were normalized with the same maxima.

Size-Exclusion Chromatography (SEC) of TetraDVP conjugates

Size-exclusion chromatography (SEC) was carried out using a Superdex 200 10/300 GL column. Samples were injected at a concentration of 1 mg/mL and eluted with TBS buffer (25 mM Tris HCl pH 8, 200 mM NaCl, 0.5 mM EDTA) at a flow rate of 0.5 mL/min.

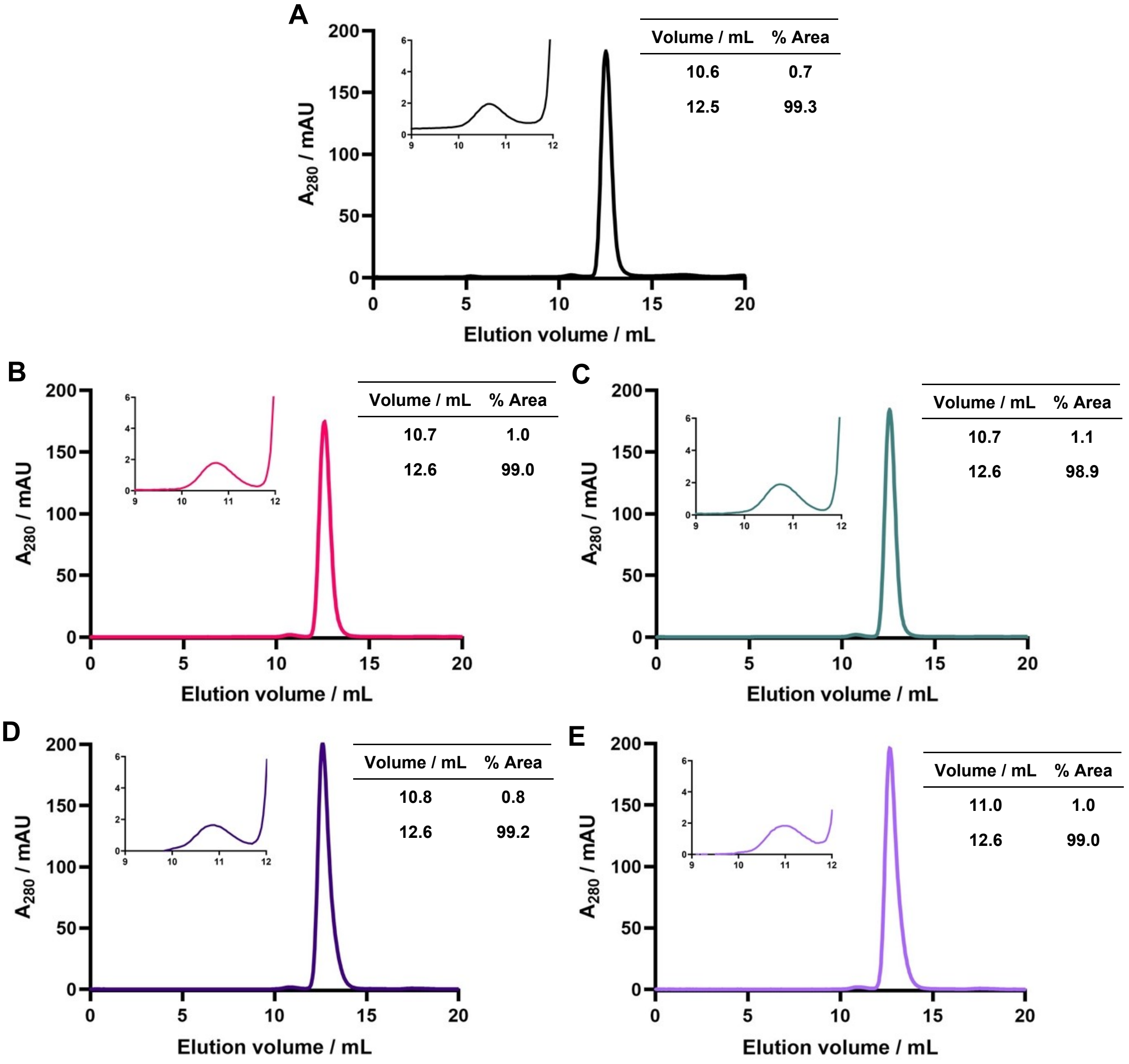

Figure S17. Size-exclusion chromatograms of (A) trastuzumab (B) conjugate **6** (C) conjugate **10** (D) conjugate **11** and (E) conjugate **12**.

Enzyme-linked immunosorbent assay (ELISA)

A 96-well plate was coated with 100 µL of a 0.25 µg/mL solution of HER2 (Sino Biological, His-tagged) overnight at 4 °C. Coating solutions were removed and each well washed with PBS (2 × 200 µL). Each well was then blocked with 1% BSA in PBS (200 µL) for 1 h at room temperature. The blocking solution was then removed and each well washed with PBS (3 × 200 µL). Wells were treated with a serial dilution of trastuzumab and trastuzumab-TetraDVP conjugates **6**, **10**, **11** and **12** in PBS (100 µL of 90 nM, 30 nM, 10 nM, 3.33 nM, 1.11 nM, 0.37 nM, 0.12 nM, 0 nM) and incubated at room temperature for 2 h. The conjugate solutions were removed and each well was washed with 0.1% Tween 20 in PBS (2 × 200 µL) followed by PBS (3 × 200 µL). Next, 100 µL of detection antibody (1:500 dilution of a mouse anti-human IgG-HRP, ThermoFisher) in PBS was added to each well and incubated at room temperature for 1 h. Each well was washed with 0.1% Tween 20 in PBS (2 × 200 µL) followed by PBS (3 × 200 µL). Finally, an OPD solution (100 µL of a solution prepared by dissolving 1 capsule in 9 mL H_2O and 1 mL stable peroxide substrate buffer (10×), ThermoFisher) was added to each well. After 15 minutes, 4M HCl (aq.) (50 µL) was added to each well to quench the reaction. Absorbance at 490 nm and 590 nm was measured using a CLARIOstar Microplate Reader. Measurements were performed in quadruplicate and three independent repeats were performed.

Preparation of antibody-fluorophore conjugates (AFCs)

To a solution of TetraDVP conjugate **6**, **10**, **11** or **12** in PBS were added $CuSO_4 \cdot 5H_2O$ (20 equiv. per alkyne), THPTA (100 equiv. per alkyne), sodium ascorbate (150 equiv. per alkyne) and AlexaFluor™ 488 Azide (Thermo Fisher Scientific) (20 mM in DMSO, 12 equiv. per alkyne). The mixture was vortexed and incubated at 37 °C for 4 h (for **6** or **10**) or 6 h (for **11** or **12**). The excess reagents were removed by filtration through two Zeba™ Spin Desalting Columns (7,000 MWCO, Thermo Fisher Scientific), followed by repeated diafiltration into PBS using an Amicon-Ultra centrifugal filter (10,000 MWCO, Merck Millipore).

Fluorophore-to-antibody ratios (FAR) were determined by UV-vis spectroscopy. Sample buffer was used as blank for baseline correction with extinction coefficients ε_{280} = 215,380 M^{-1} cm^{-1} for trastuzumab and ε_{495} = 71,000 M^{-1} cm^{-1} for AlexaFluor 488™ (AF488). The correction factor for AF488 absorption at 280 nm is 0.11. FAR was calculated using the following formula:

$$FAR = \frac{Abs_{495}/\varepsilon_{495}}{(Abs_{280} - 0.11 \times Abs_{495})/\varepsilon_{280}}$$

Live cell microscopy

SKBR3 or MCF7 cells were seeded at 40,000 cells/well in 8-well chambered µ-slide (Ibidi, 80826) for 48 h at 37 °C with 5% CO_2. Slides were then placed on ice and washed with Ham's F12 Nutrient Mix media containing 10% FBS, 2 mM *L*-glutamine, 50 U/mL penicillin and 50 µg/mL streptomycin (3 × 200 µL). Antibody conjugates **13** and **14** (50 nM), trastuzumab (50 nM) or vehicle (PBS) were added to the cells in complete F12 growth medium and incubated at 4 °C in the dark for 1 h. Cells were placed back on ice and washed with complete F12 growth medium (3 × 200 µL). Complete F12 growth medium (200 µL) was added and the cells incubated for 3.5 h at 37 °C with 5% CO_2. After 3 h incubation, Hoechst 33342 trihydrochloride trihydrate (1 µg/mL, Invitrogen, H3570) was added. Live cell microscopy was performed on an Operetta CLS confocal microscope (Perkin Elmer) with a 40× water objective. Cells were maintained in a humidified atmosphere at 37 °C and 5% CO_2 throughout analysis. Data analysis was performed using ImageJ (Fiji).

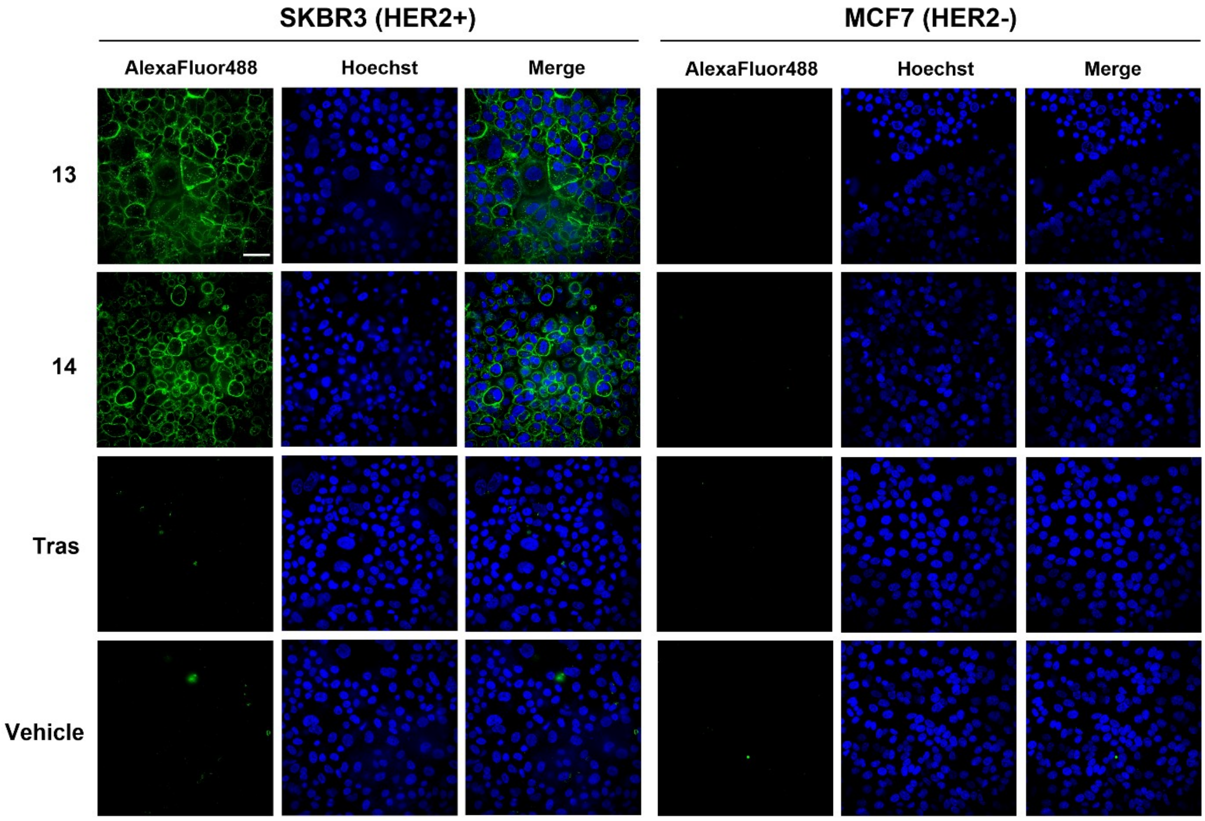

Figure S18. Live cell microscopy images of HER2-positive SKBR3 cells and HER2-negative MCF7 cells after treatment with fluorescent conjugates **13** or **14**, trastuzumab or vehicle control. Scale bar represents 50 µm.

Stability Analysis

To a solution of trastuzumab-AlexaFluor488 conjugate **13**, **14**, **15** or **16** (138 µL, 3.75 µM) in PBS were added 12 µL of reconstituted human plasma (Sigma). The mixture was incubated at 37 °C for 14 days. Aliquots were removed after 0, 2, 4, 6, 8, 10, 12 and 14 days, flash frozen and stored at -80 °C until analysis. SDS-PAGE was followed by in-gel fluorescence and coomassie brilliant blue staining.

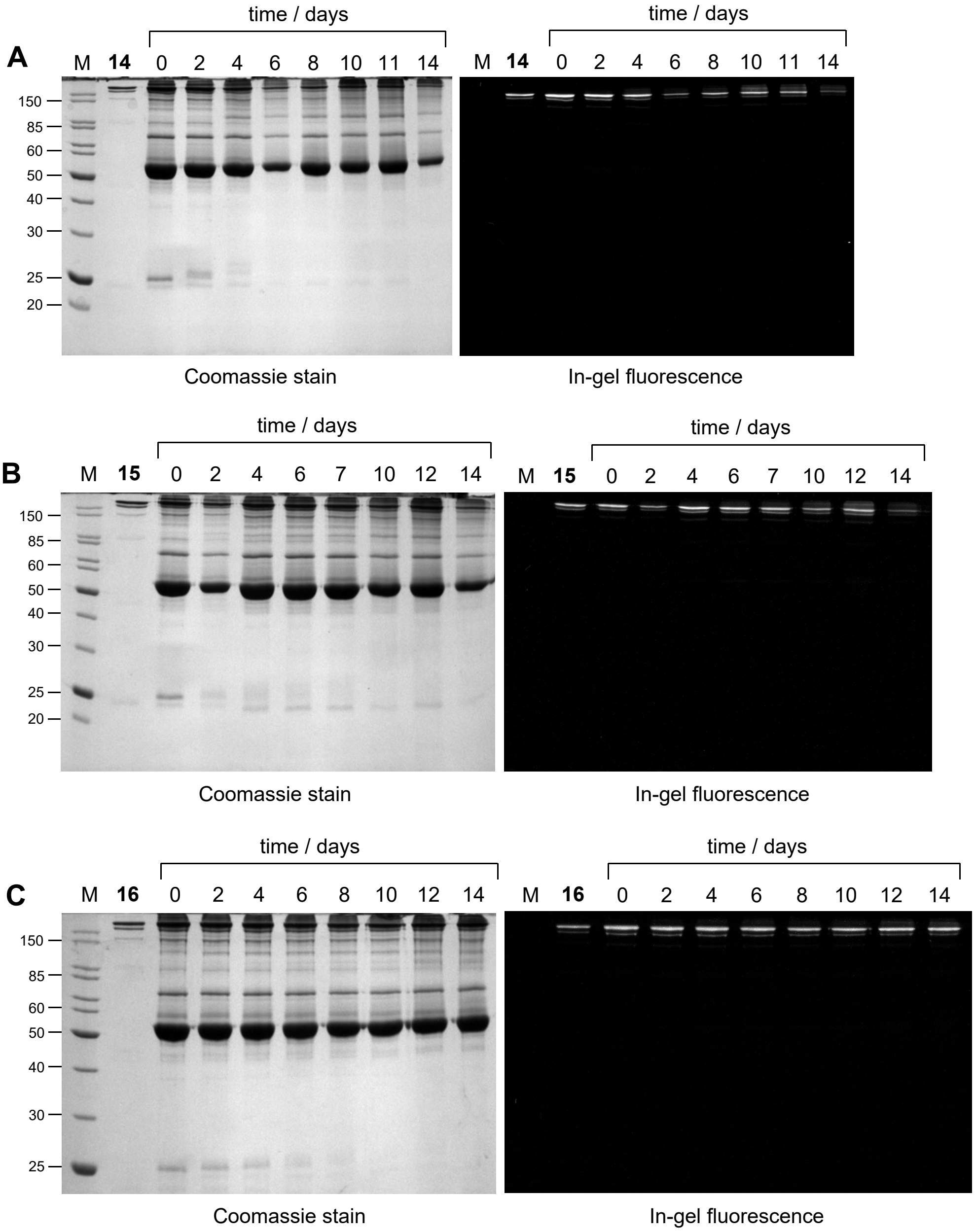

Figure S19. Stability analysis by SDS-PAGE of antibody-fluorophore conjugates human plasma; (A) conjugate **14** (FAR = 2.0); (B) conjugate **15** (FAR = 2.9); (C) conjugate **16** (FAR = 4.0); M = molecular weight marker; days of incubation above the representative lane. Left gel is after coomassie staining, right gel is in-gel fluorescence measured before staining. No significant transfer of AlexaFluor™ 488 to human serum albumin (66.5 kDa) was observed for any of the conjugates over the 14-day incubation period.

Preparation of antibody-drug conjugates (ADCs)

To a solution of TetraDVP conjugate **6**, **10**, **11** or **12** in PBS were added $CuSO_4 \cdot 5H_2O$ (150 equiv.), THPTA (600 equiv.), sodium ascorbate (1000 equiv.) and N_3-PEG_4-Val-Cit-PABC-MMAE **38** (20 mM in DMSO, 100 equiv.). The mixture was vortexed and incubated at 37 °C for 6 h. The excess reagents were removed by filtration through two Zeba™ Spin Desalting Columns (40,000 MWCO, Thermo Fisher Scientific), followed by repeated diafiltration into PBS using an Amicon-Ultra centrifugal filter (10,000 MWCO, Merck Millipore) to yield ADCs **17**-**20**.

Drug-to-antibody ratios (DAR) were determined using hydrophobic interaction chromatography (HIC).

Hydrophobic interaction chromatography (HIC)

Hydrophobic interaction chromatography (HIC) was carried out using a Tosoh Bioscience TSKgel Butyl-NPR column (3.5 cm × 4.6 mm, 2.5 µm particle size) at a flow rate of 0.6 mL/min and a gradient of 0-70% B over 35 column volumes where Solvent A: 1.5 M ammonium sulfate, 25 mM NaPi, pH 7; Solvent B: 25% isopropyl alcohol in 25 mM NaPi, pH 7.

Average DAR for each ADC was calculated as follows, where DAR_n corresponds to the peak area at 280 nm for a given DAR species, with n representing the number of MMAE molecules per antibody for that DAR species.

$$Average\ DAR = \frac{(DAR_1 + 2 \times DAR_2 + 3 \times DAR_3 + 4 \times\ DAR_4 + 5 \times DAR_5)}{(DAR_0 + DAR_1 + DAR_2 + DAR_3 + DAR_4 + DAR_5)}$$

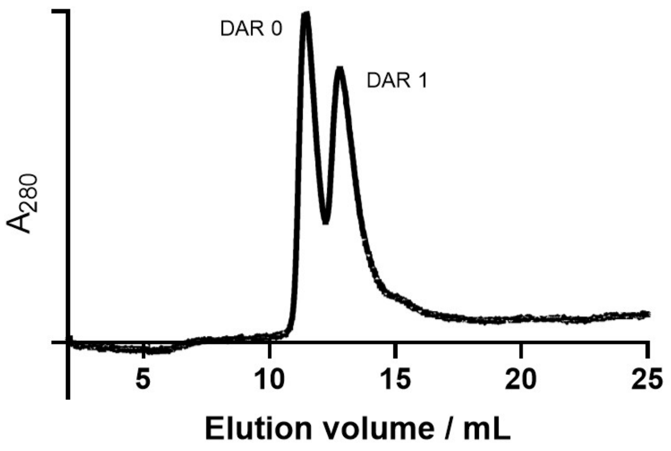

Figure S20. HIC trace of TetraDVP ADC **17** (DAR 0.5).

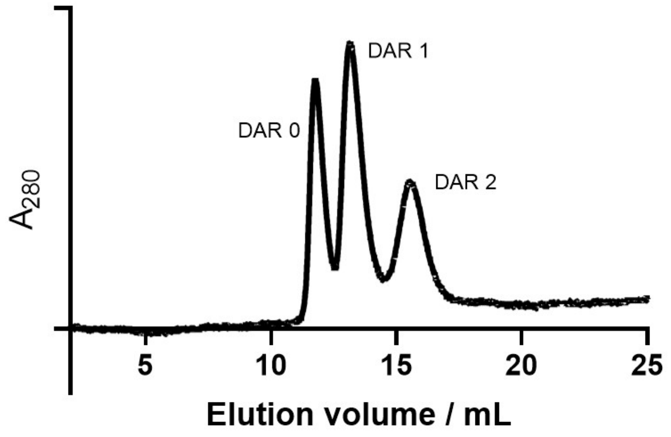

Figure S21. HIC trace of TetraDVP ADC **18** (DAR 1.0).

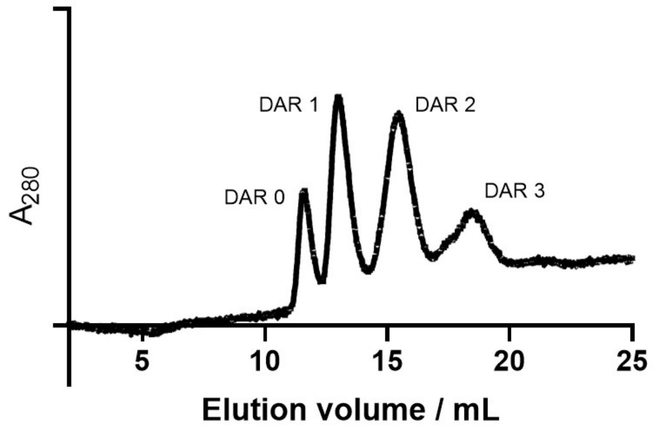

Figure S22. HIC trace of TetraDVP ADC **19** (DAR 1.6).

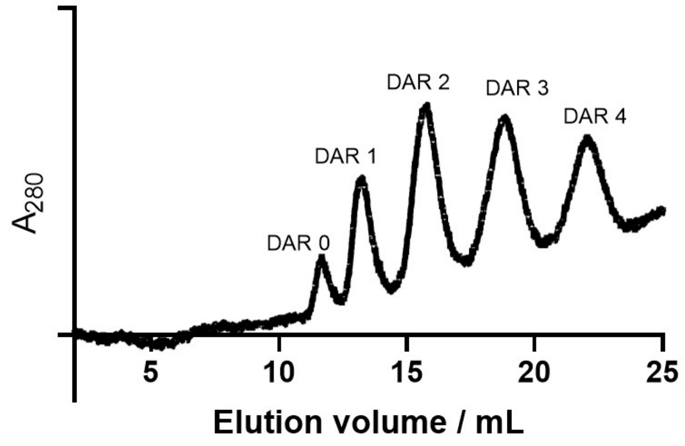

Figure S23. HIC trace of TetraDVP ADC **20** (DAR 2.4).

Size-Exclusion Chromatography (SEC) of ADCs

Size-exclusion chromatography (SEC) was carried out on an Agilent 1200 Series system using a TSKgel G3000SWXL column (30 cm × 7.8 mm, 5 µm particle size) with a mobile phase of PBS (50 mM sodium phosphates, 100 mM NaCl, 0.02% sodium azide, pH 7.0) at a flow rate of 0.5 mL/min over 30 min. 10 µg of trastuzumab or ADC (1-2 mg/mL in PBS) was analysed per run. Samples were analysed *via* absorption at 280 nm.

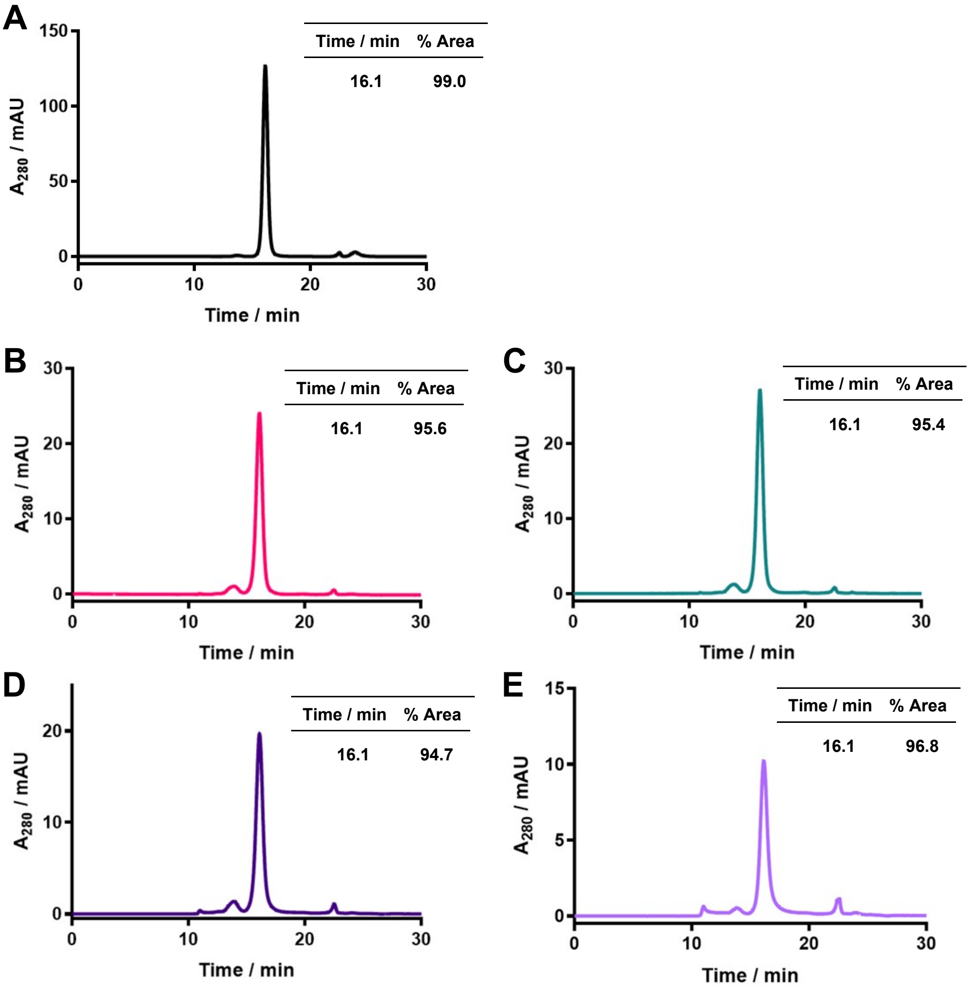

Figure S24. Size-exclusion chromatograms of (A) trastuzumab (B) ADC **17** (C) ADC **18** (D) ADC **19** and (E) ADC **20**.

In vitro cytotoxicity

Cell lines

HER2-positive SKBR3 and BT474 cells were obtained from the American Type Culture Collection (ATCC) and HER2-negative MCF7 and MDA-MB-468 cells were obtained from the European Collection of Authenticated Cell Cultures (ECACC) and ATCC, respectively. SKBR3 cells were maintained in high glucose McCoy's 5A medium, supplemented with 10% heat-inactivated foetal-bovine serum (FBS), GlutaMAX™, 50 U/mL penicillin and 50 µg/mL streptomycin. MCF7 and MDA-MB-468 cells were maintained in Dulbecco's Modified Eagle Medium (DMEM) supplemented with 10% FBS, 2 mM L-glutamine, 50 U/mL penicillin and 50 µg/mL streptomycin. BT474 cells were maintained in RPMI1640 medium supplemented with 10% FBS, 2 mM L-glutamine, 50 U/mL penicillin and 50 µg/mL streptomycin. All cell lines were incubated at 37 °C with 5% CO_2.

Cell viability

Cells were seeded in 96-well plates for 24 h at 37 °C with 5% CO_2. SKBR3 cells were seeded at 15,000 cells/well, BT474 cells were seeded at 20,000 cells/well, MCF7 cells were seeded at 7,500 cells/well and MDA-MB-468 cells were seeded at 10,000 cells/well. Serial dilutions of **17**, **18**, **19**, **20** and trastuzumab were added to the cells in complete growth medium and incubated at 37 °C with 5% CO_2 for 96 h. Cell viability was measured using CellTiter-Glo viability assay (Promega) according to the manufacturer's instructions. Cell viability was plotted as a percentage of untreated cells. Each measurement was taken in triplicate and three independent repeats were performed.

NMR Spectra

Ethyl 4-((4,6-dichloropyrimidin-2-yl)amino)butanoate (21)

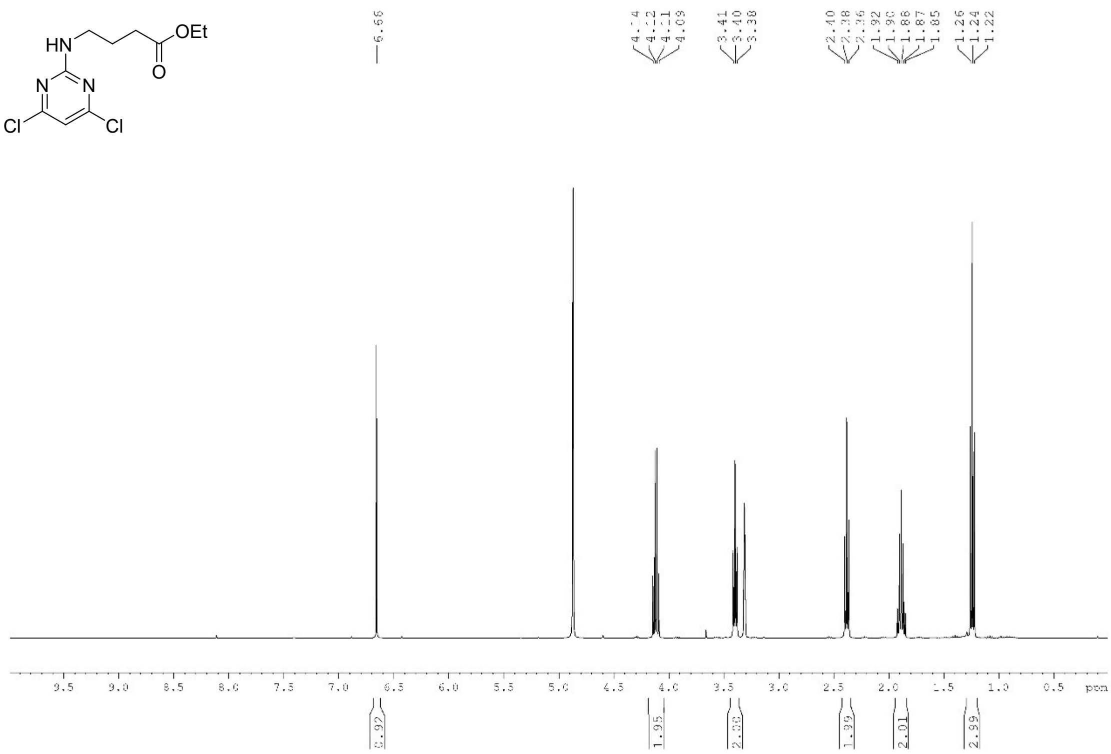

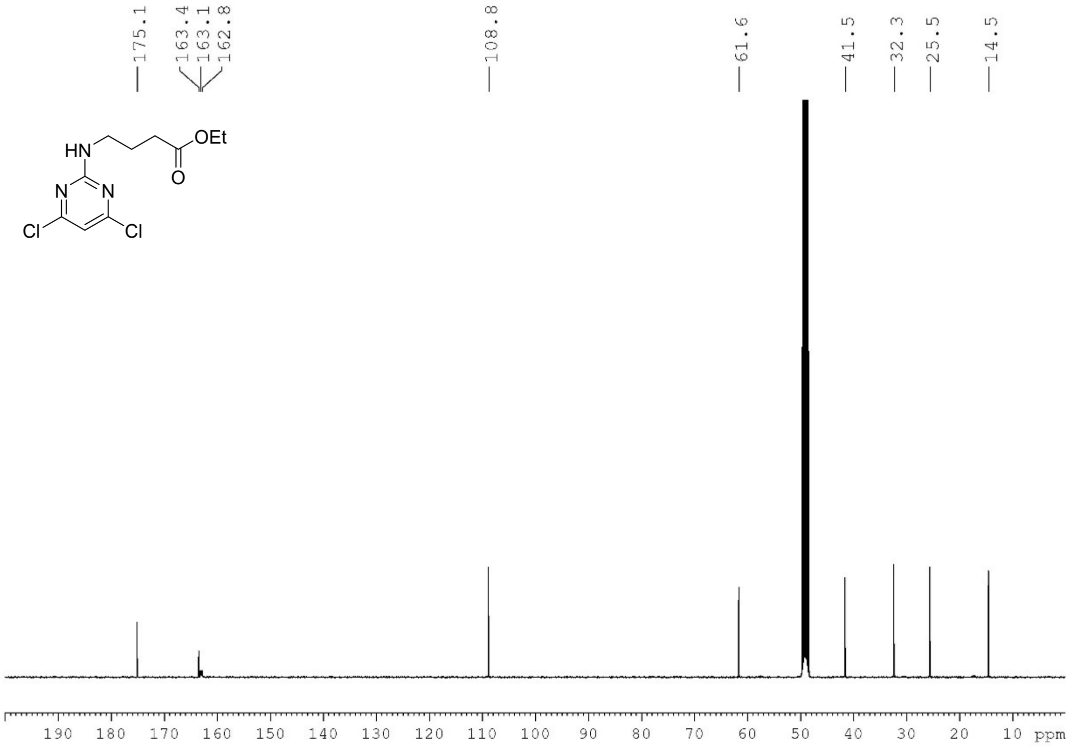

Ethyl 4-((4,6-divinylpyrimidin-2-yl)amino)butanoate (22)

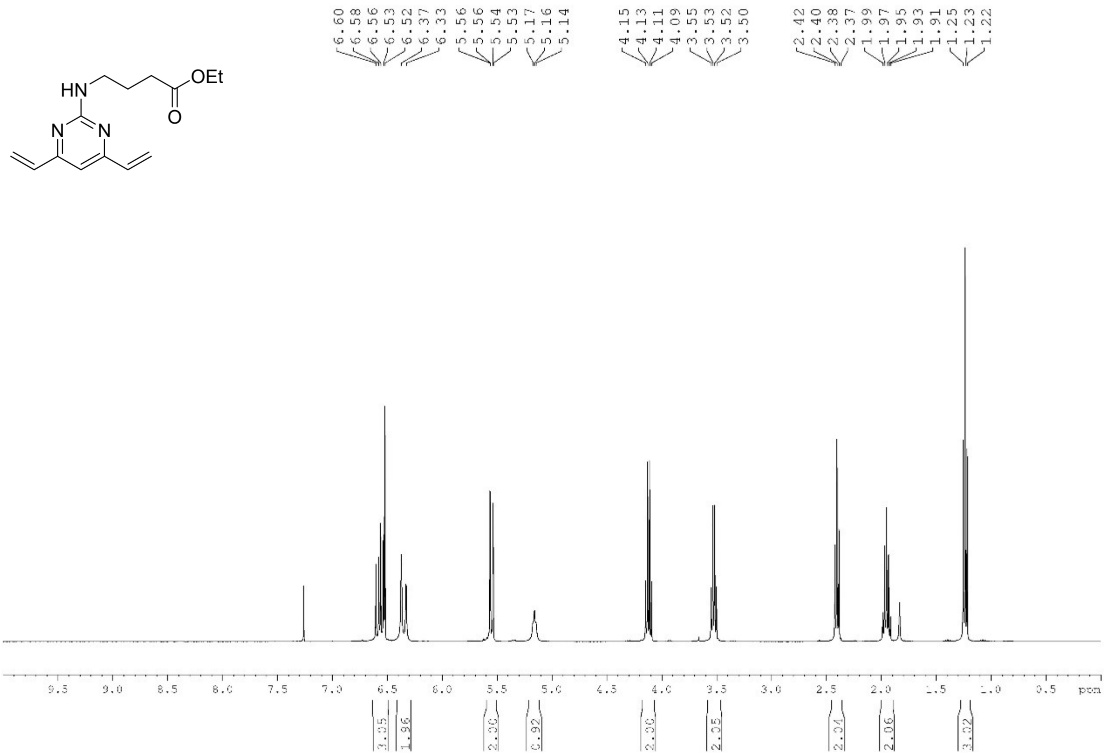

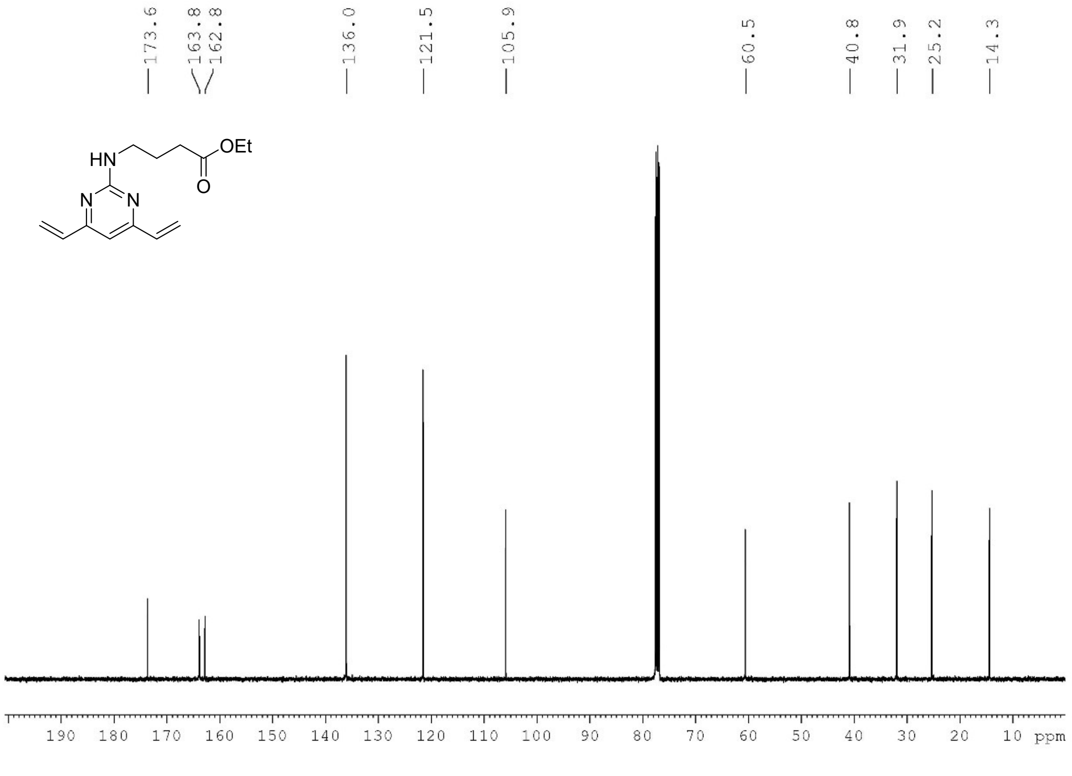

4-((4,6-divinylpyrimidin-2-yl)amino)butanoic acid (1)

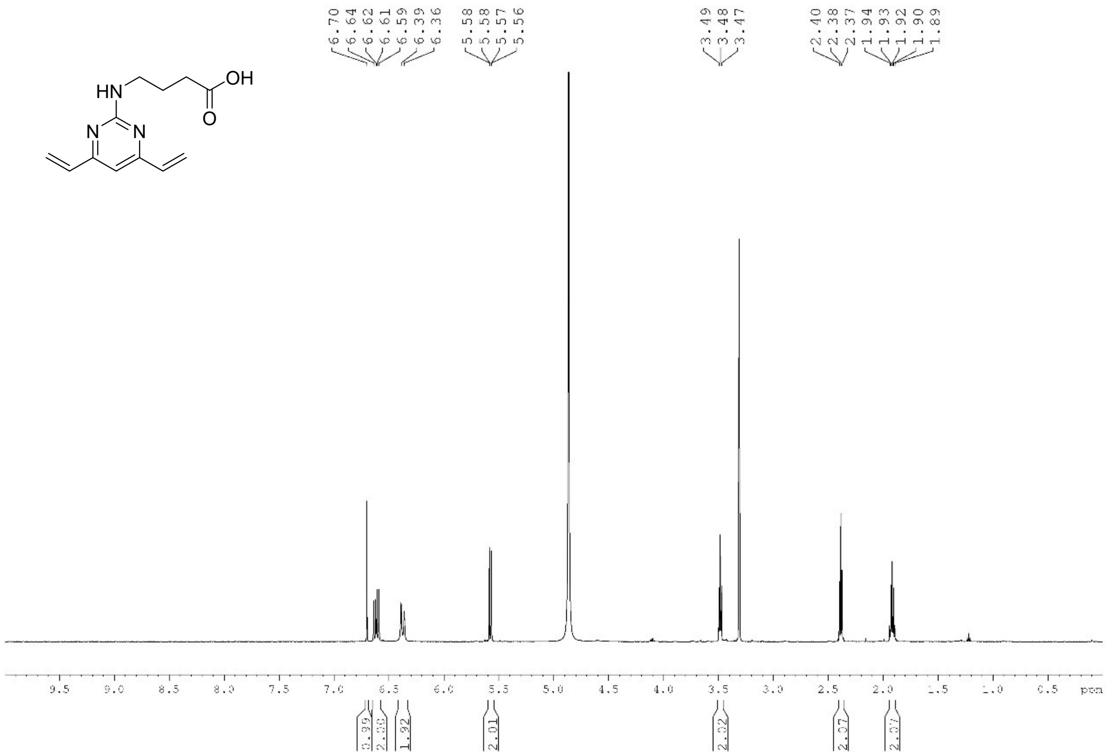

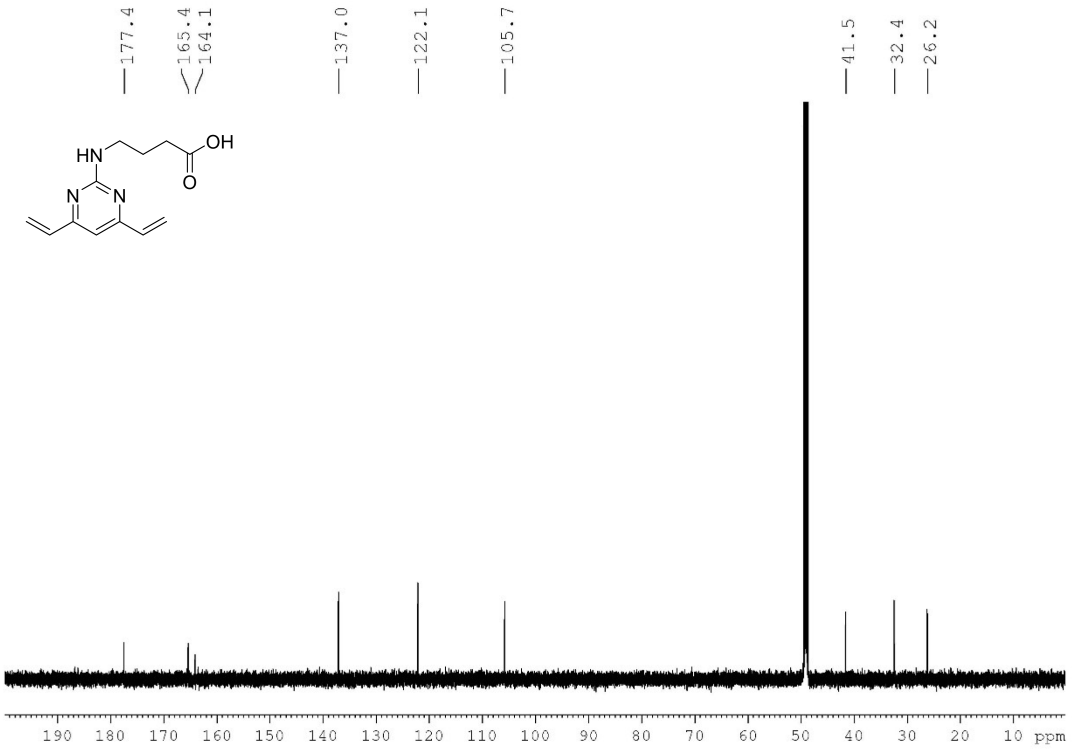

di-*tert*-butyl (azanediylbis(ethane-2,1-diyl))dicarbamate (23)

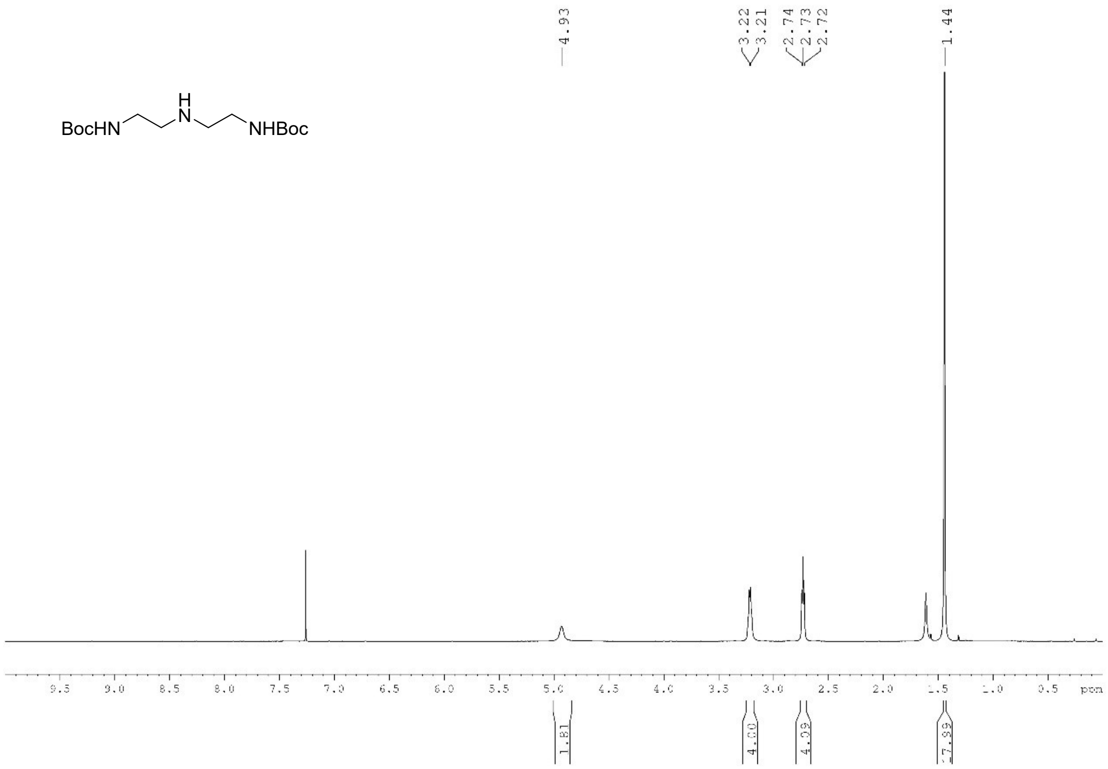

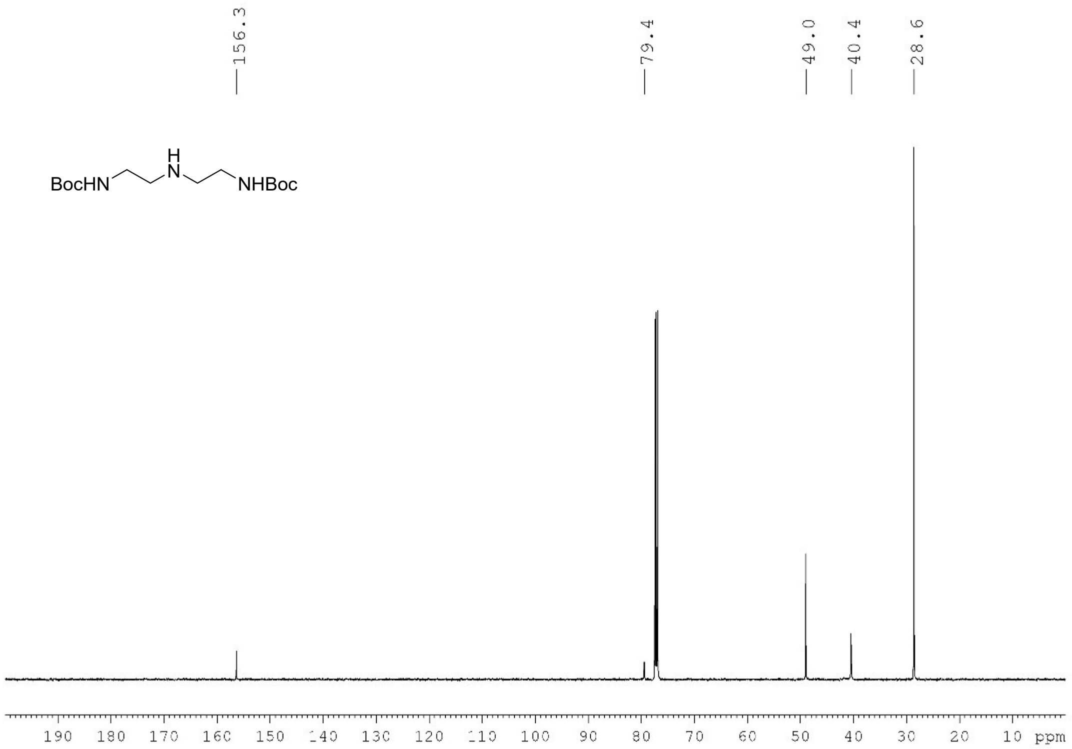

methyl bis(2-((*tert*-butoxycarbonyl)amino)ethyl)glycinate (24)

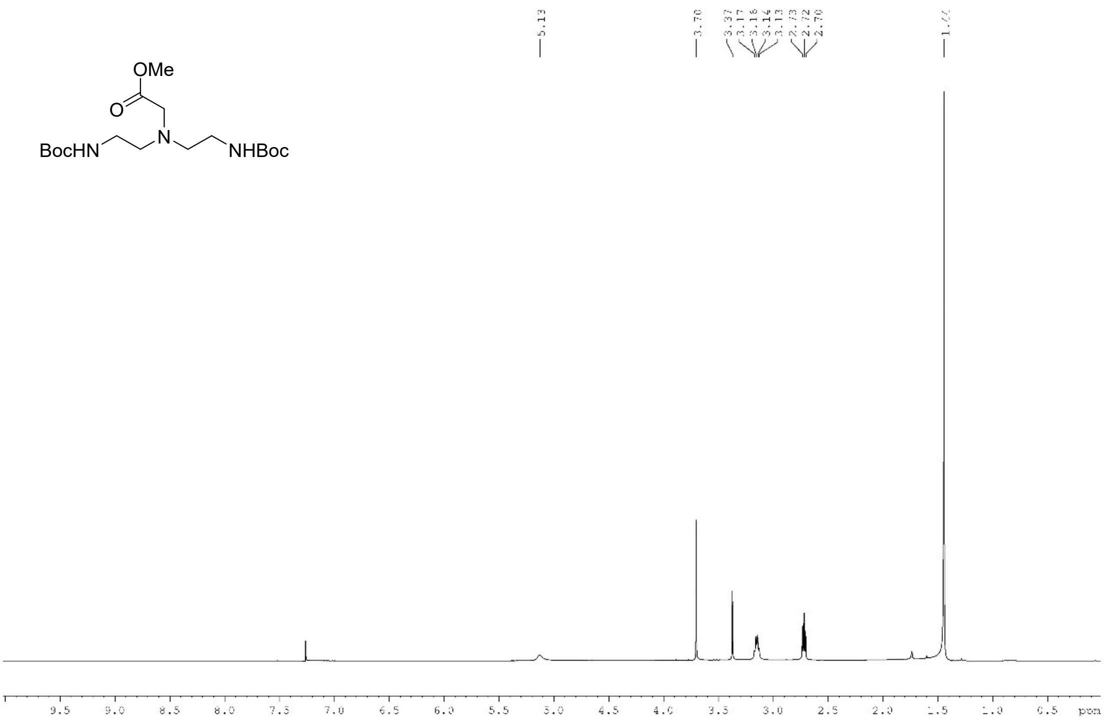

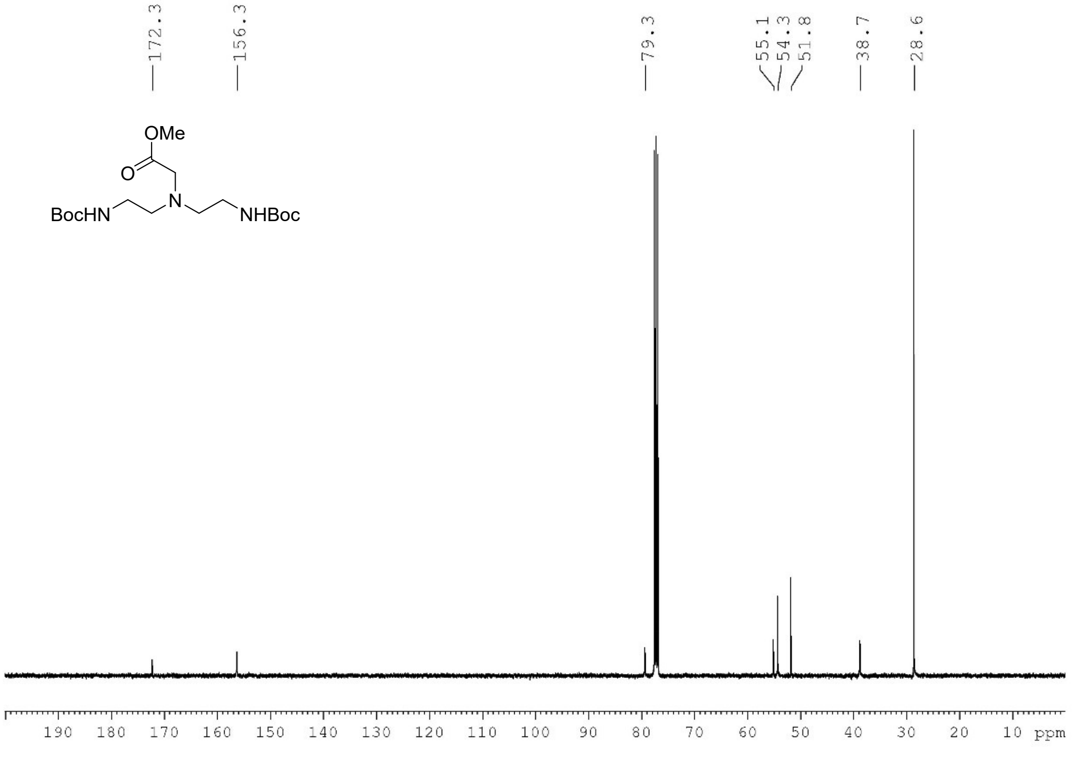

di-*tert*-butyl (((2-oxo-2-(prop-2-yn-1-ylamino)ethyl)azanediyl)bis(ethane-2,1-diyl))dicarbamate (25)

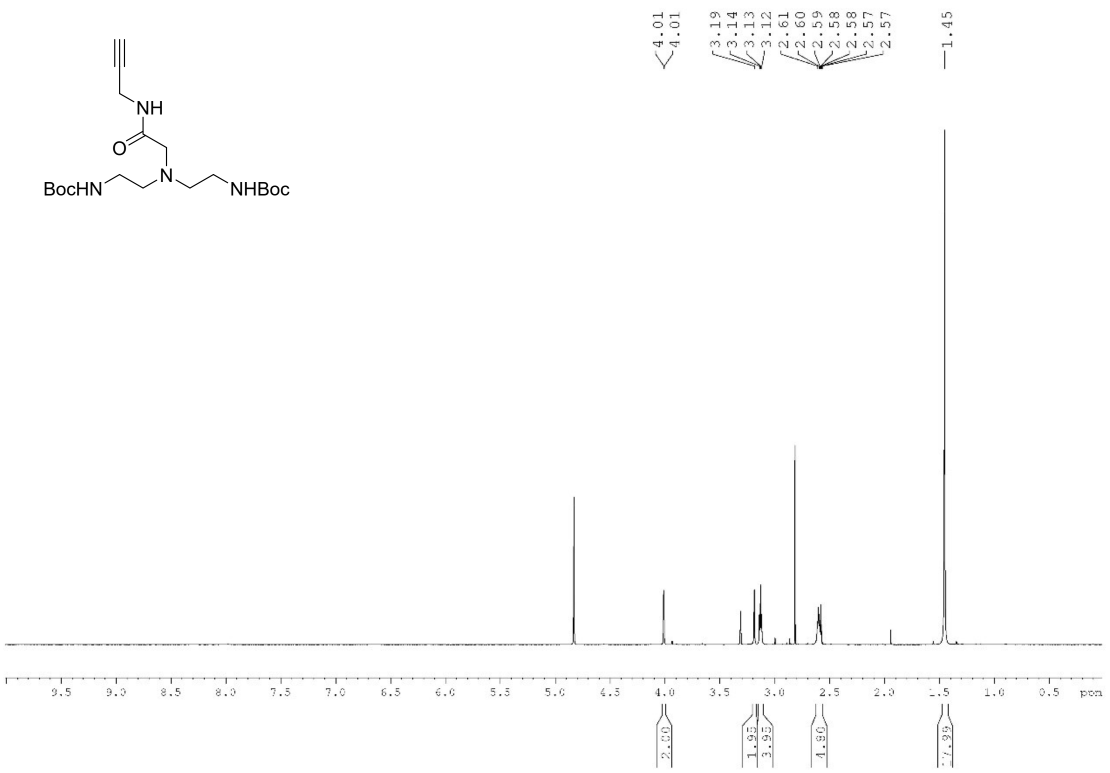

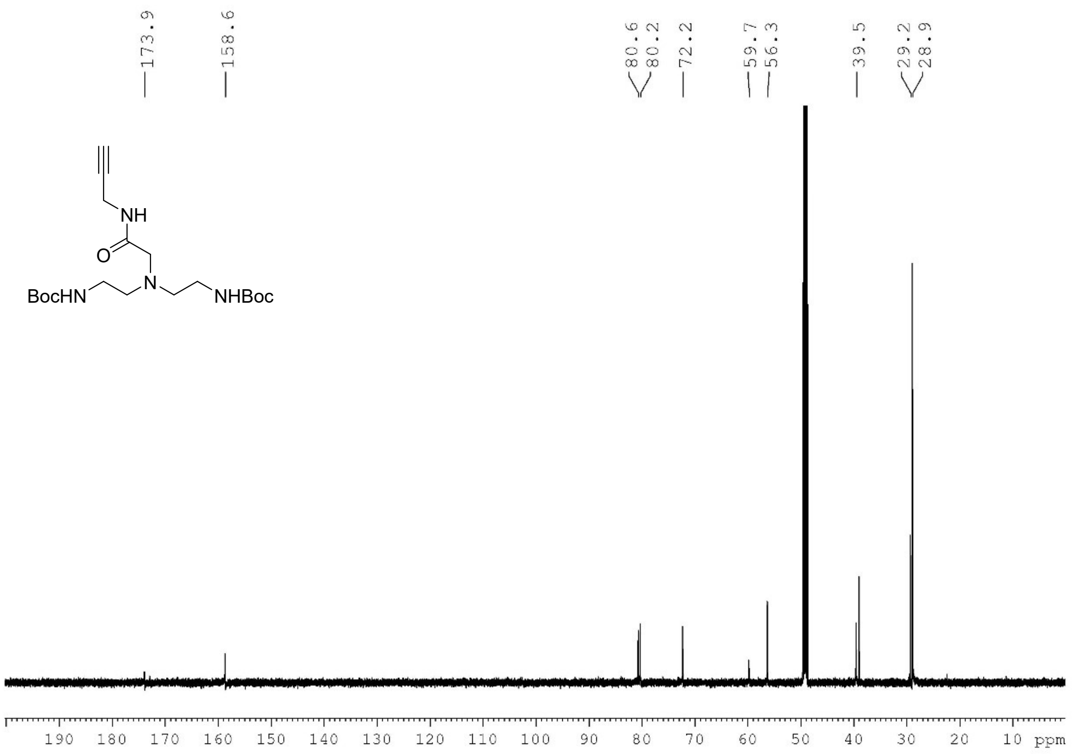

***N*,*N*'-(((2-oxo-2-(prop-2-yn-1-ylamino)ethyl)azanediyl)bis(ethane-2,1-diyl))bis(4-((4,6-divinylpyrimidin-2-yl)amino)butanamide) (2)**

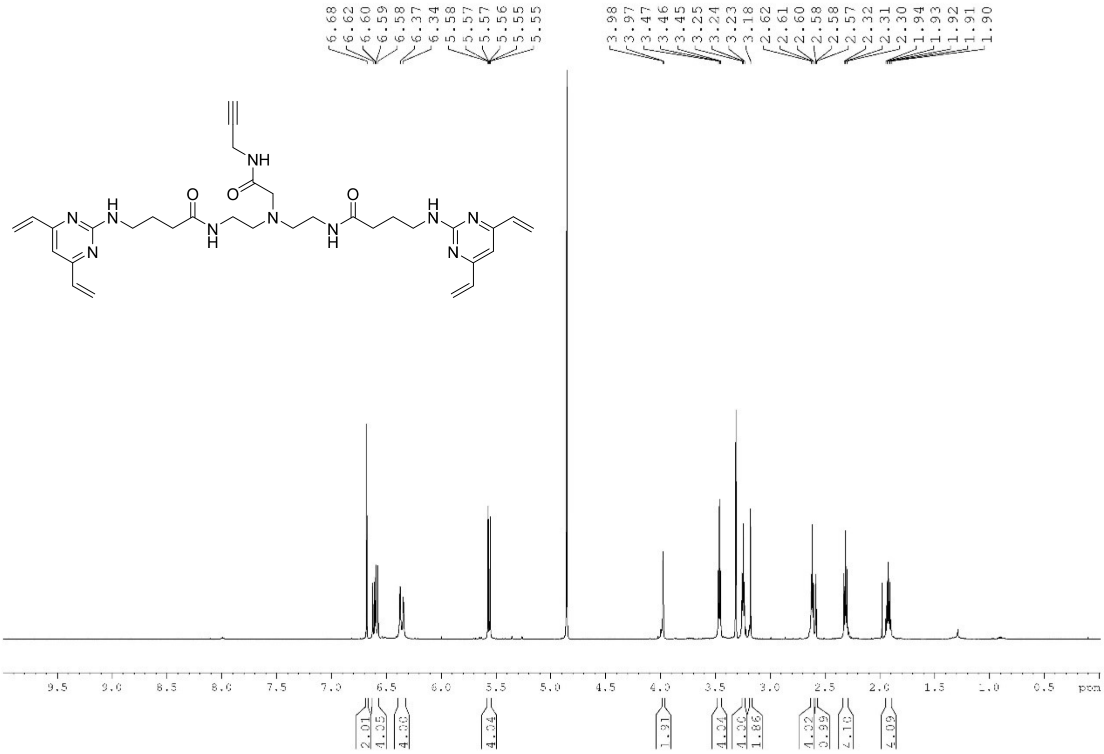

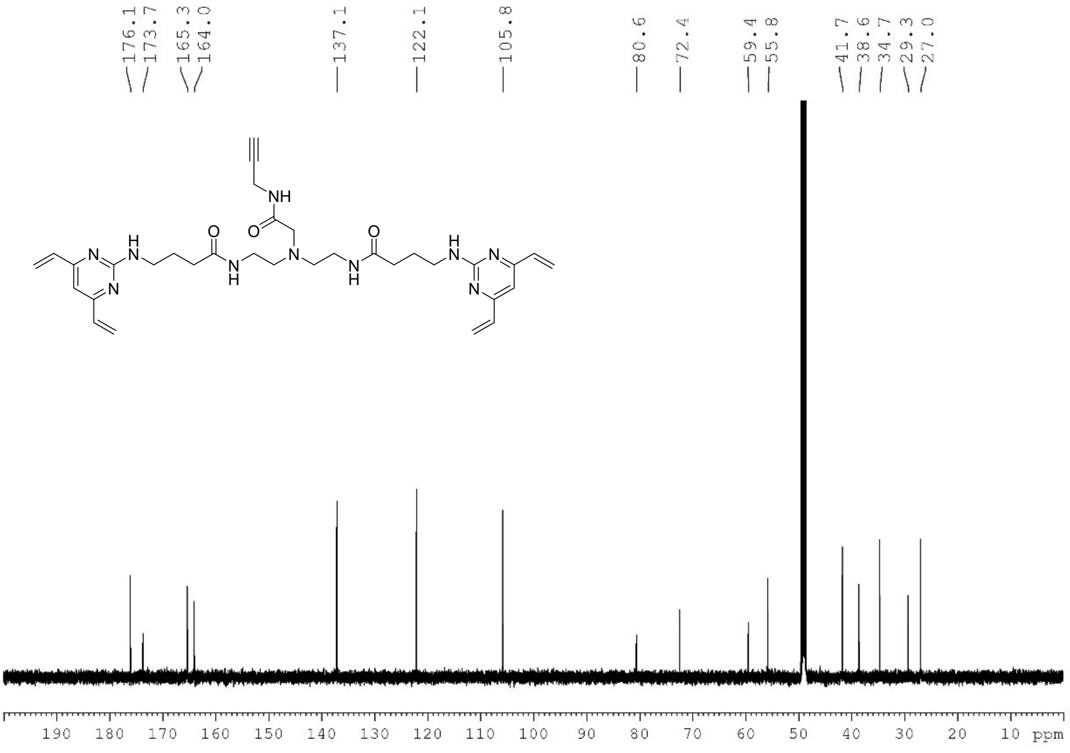

2-bromo-*N*-(prop-2-yn-1-yl)acetamide (26)

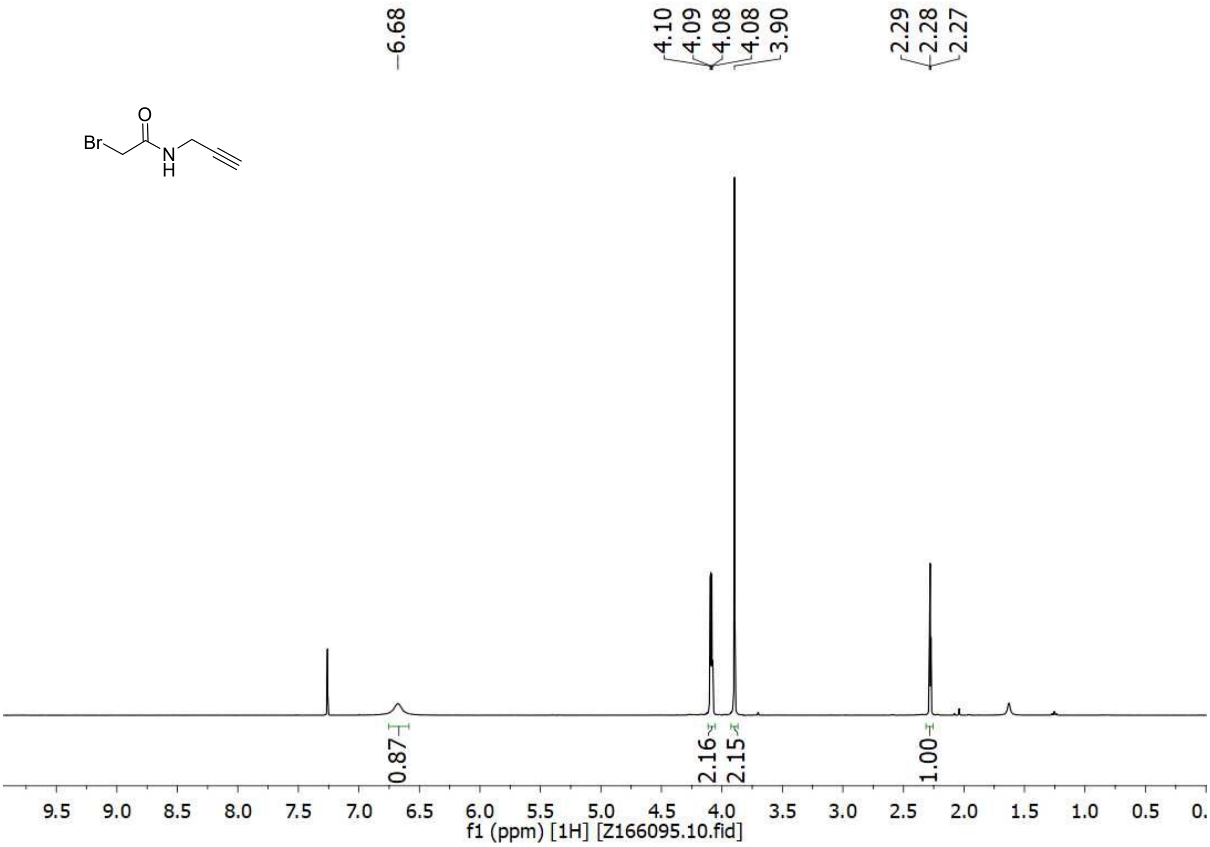

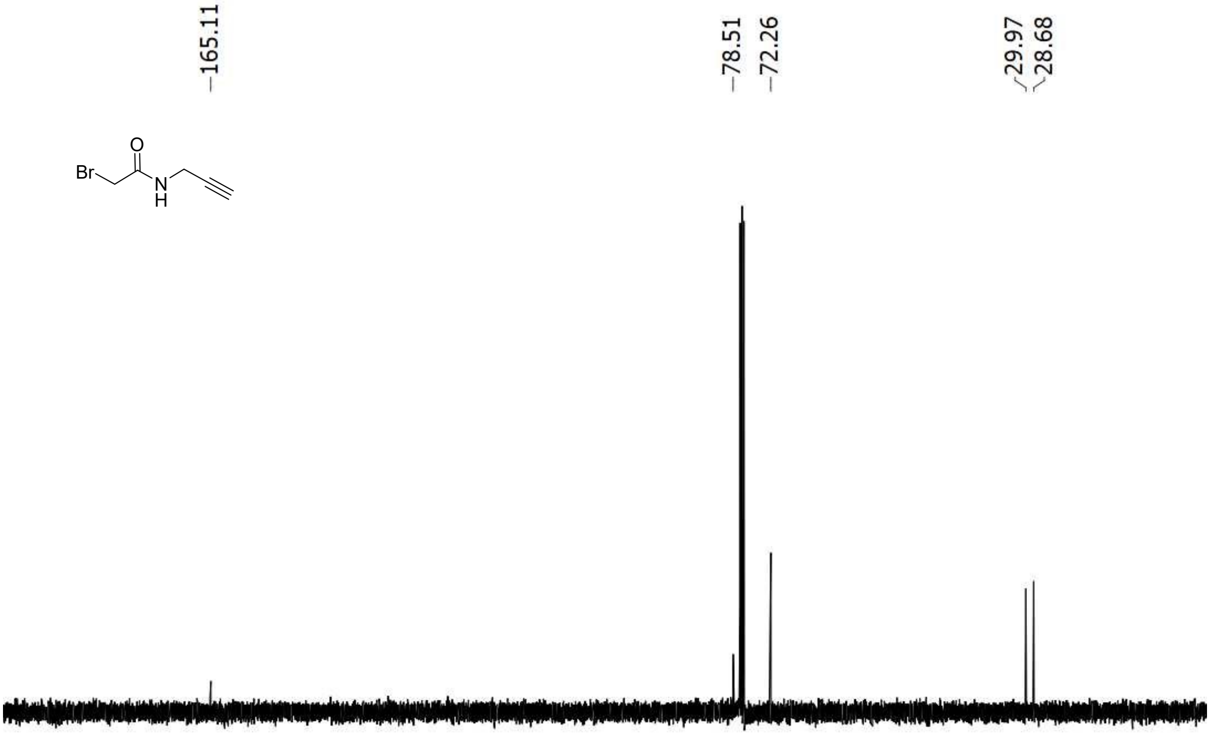

Tetra-*N*-Boc-amine backbone, $(NH)_1$ (27)

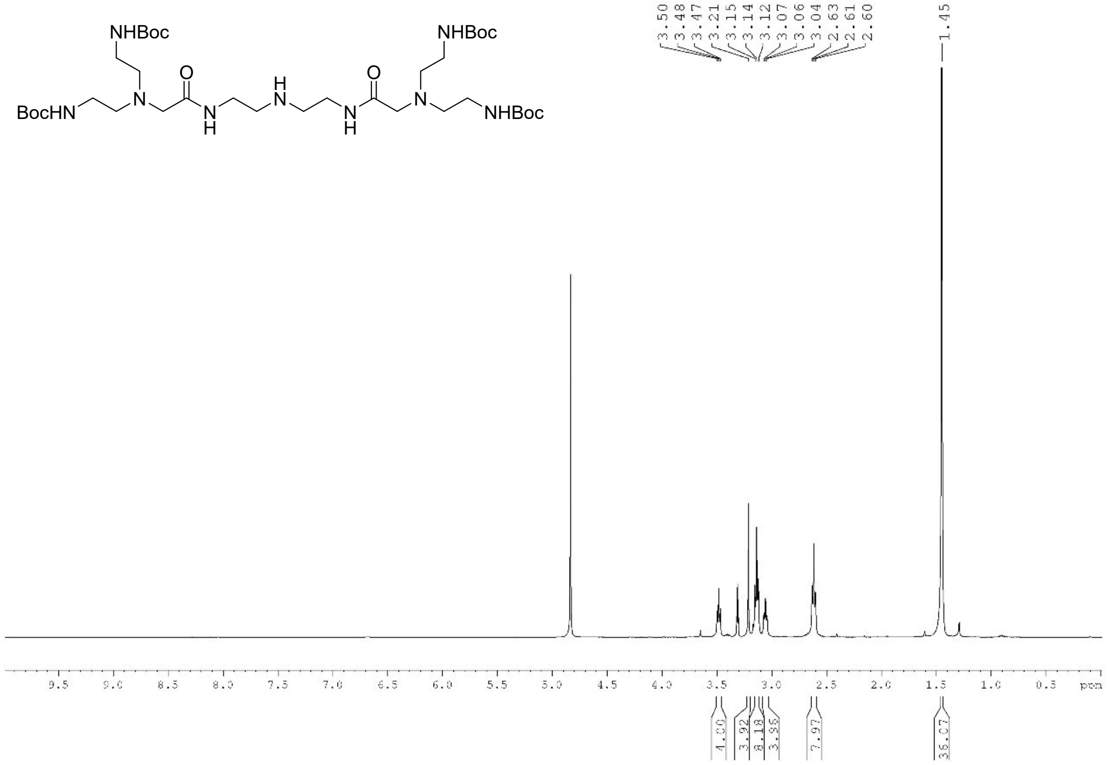

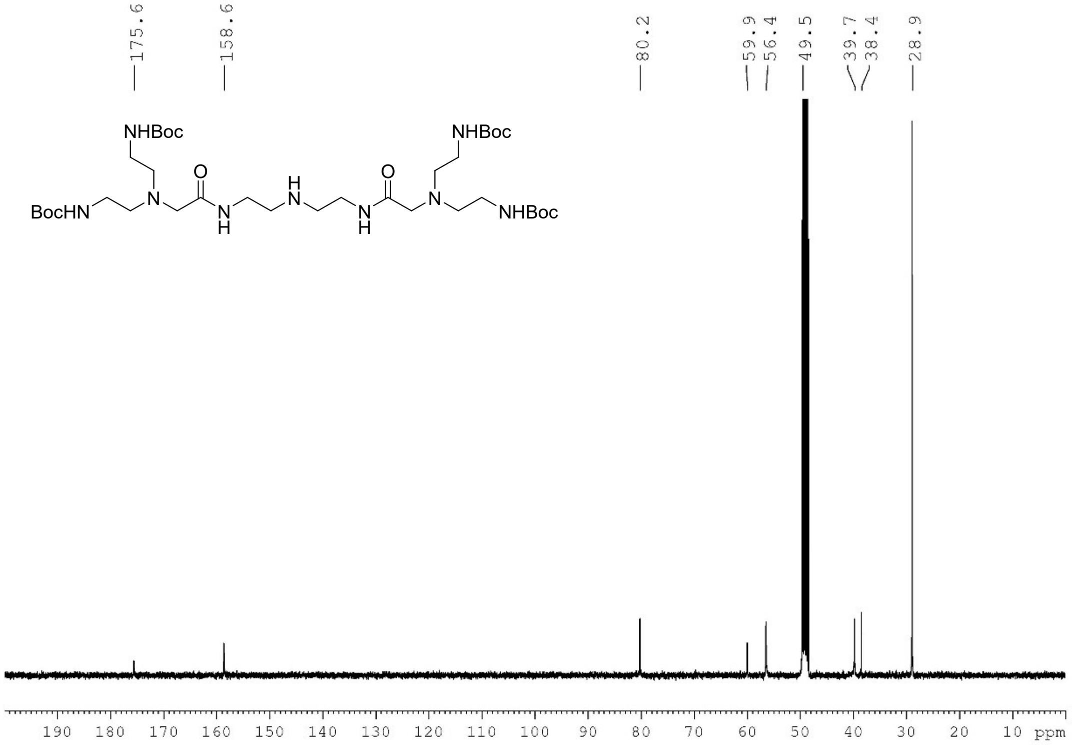

Tetra-*N*-Boc-amine backbone, (alkyne)$_1$ (28)

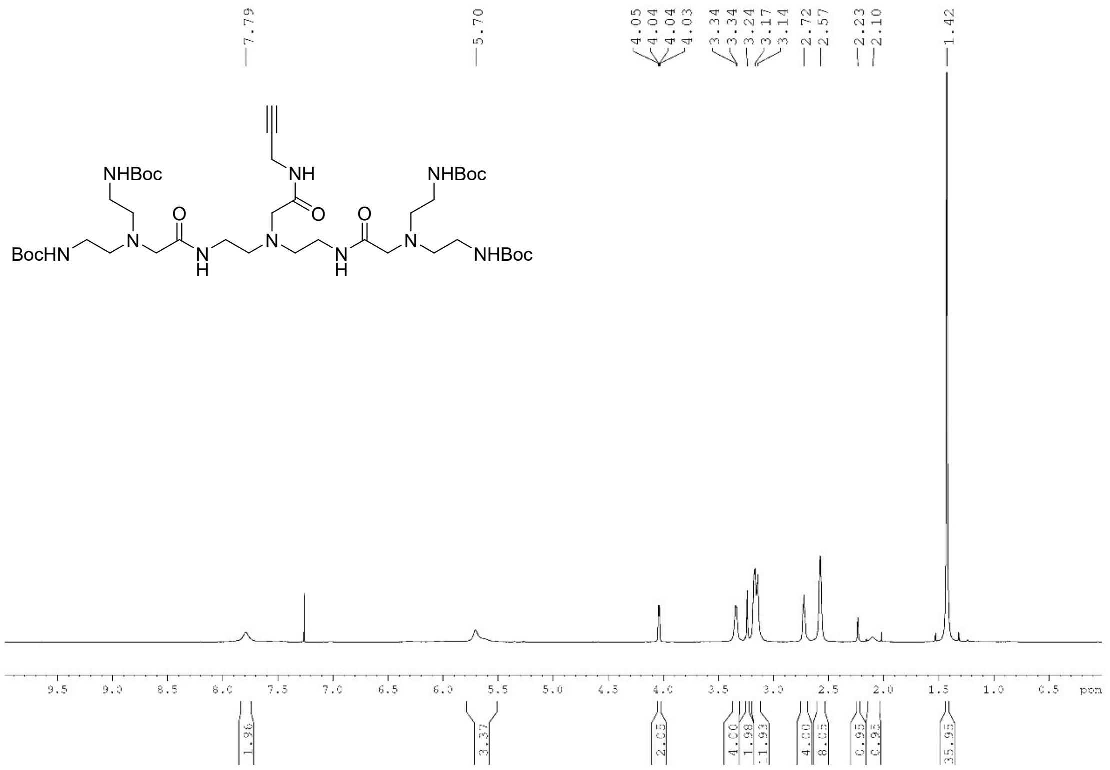

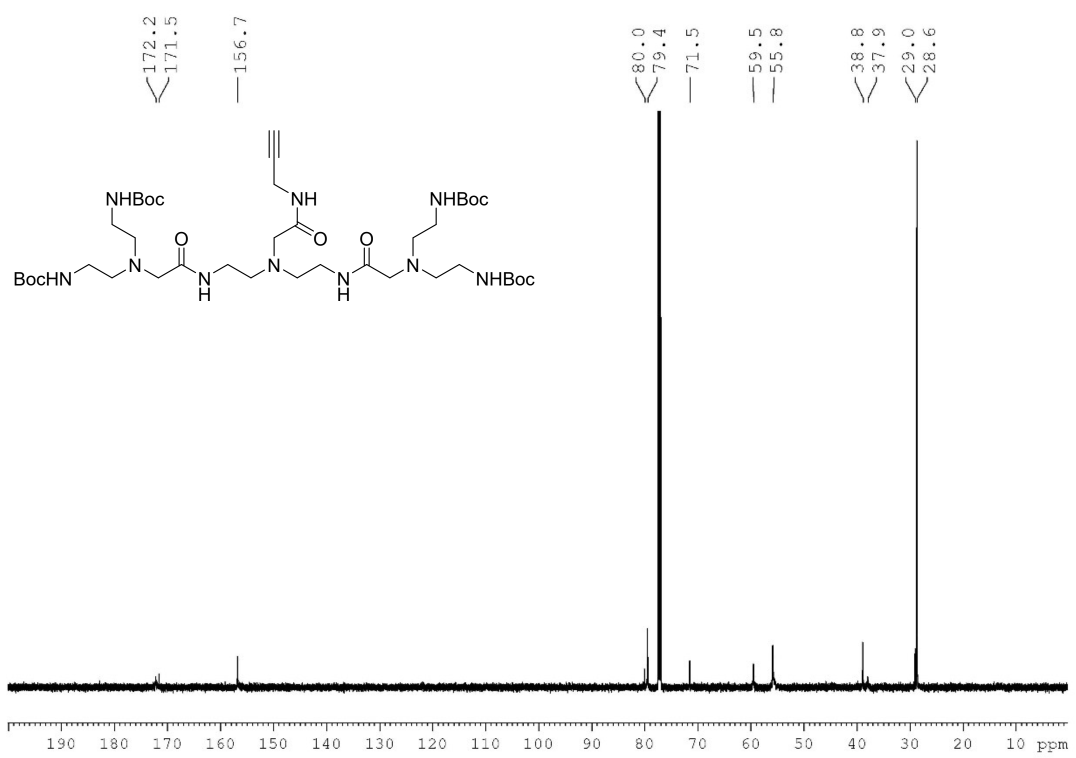

TetraDVP-(alkyne)$_1$ (3)

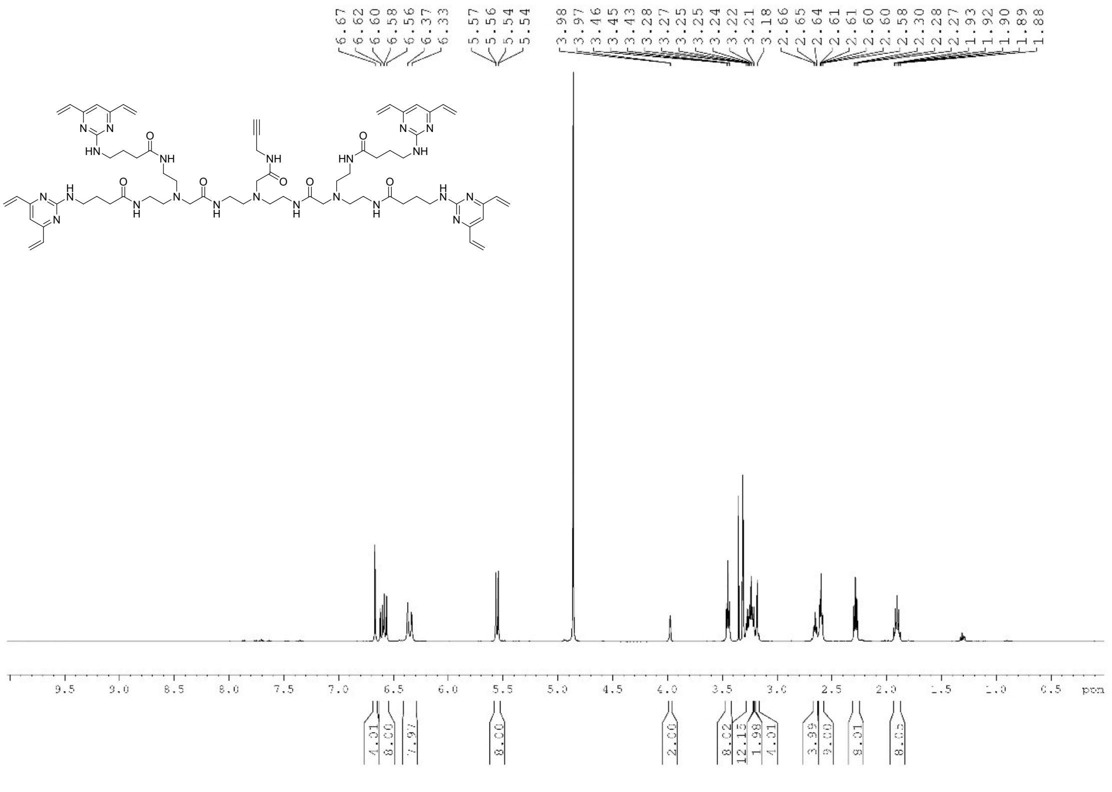

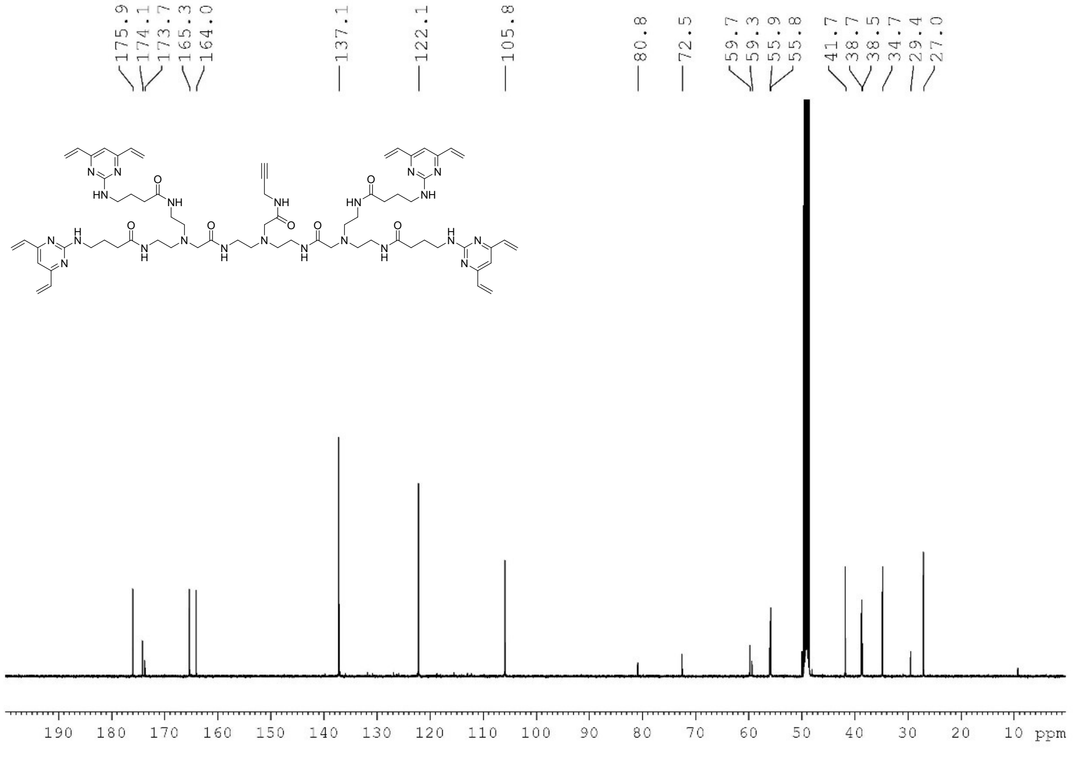

Tetra-*N*-Boc-amine backbone, $(NH)_2$ (29)

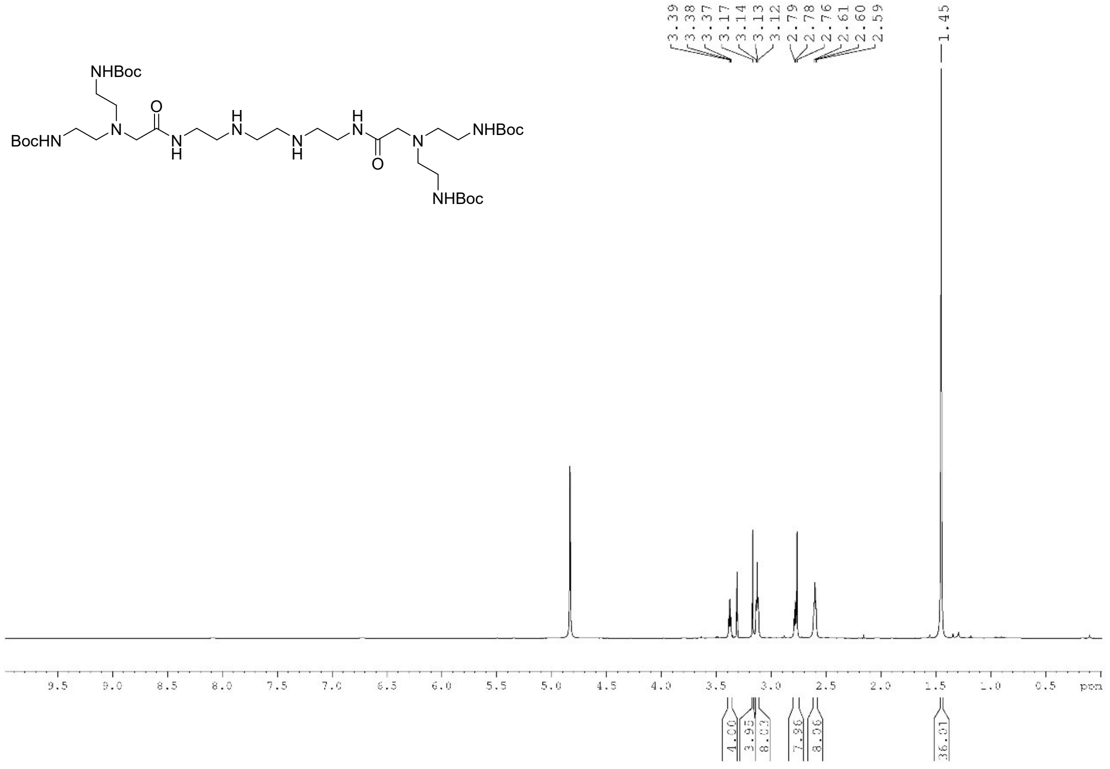

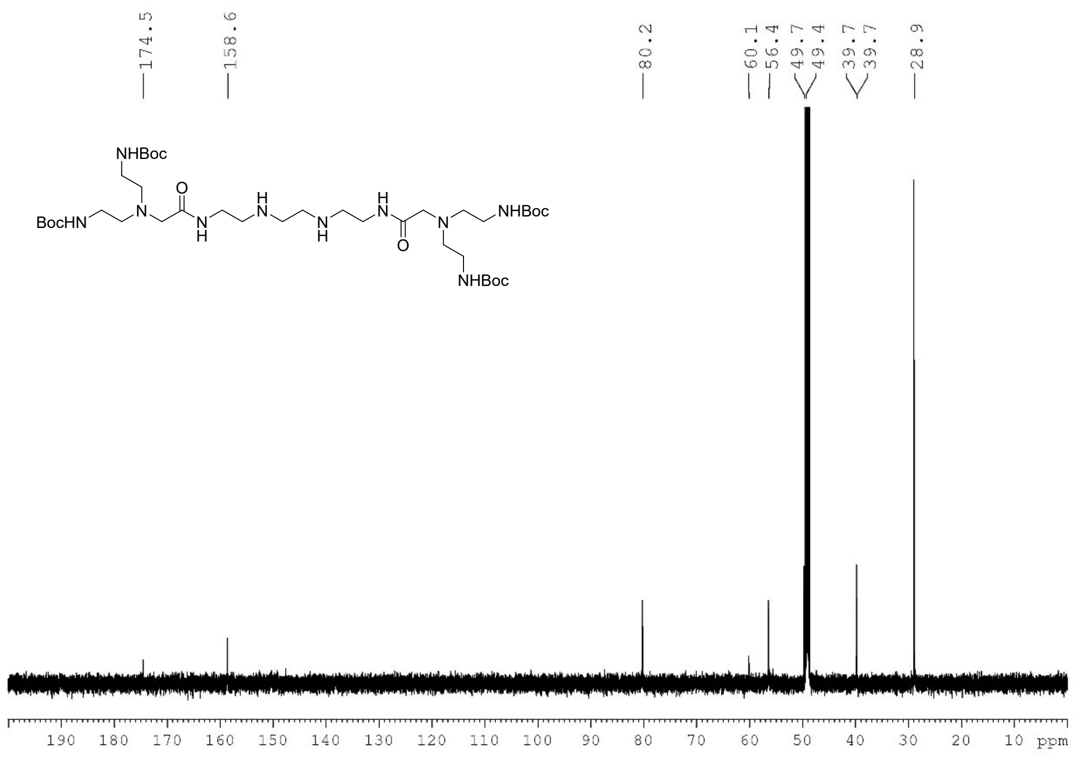

Tetra-*N*-Boc-amine backbone, (alkyne)$_2$ (30)

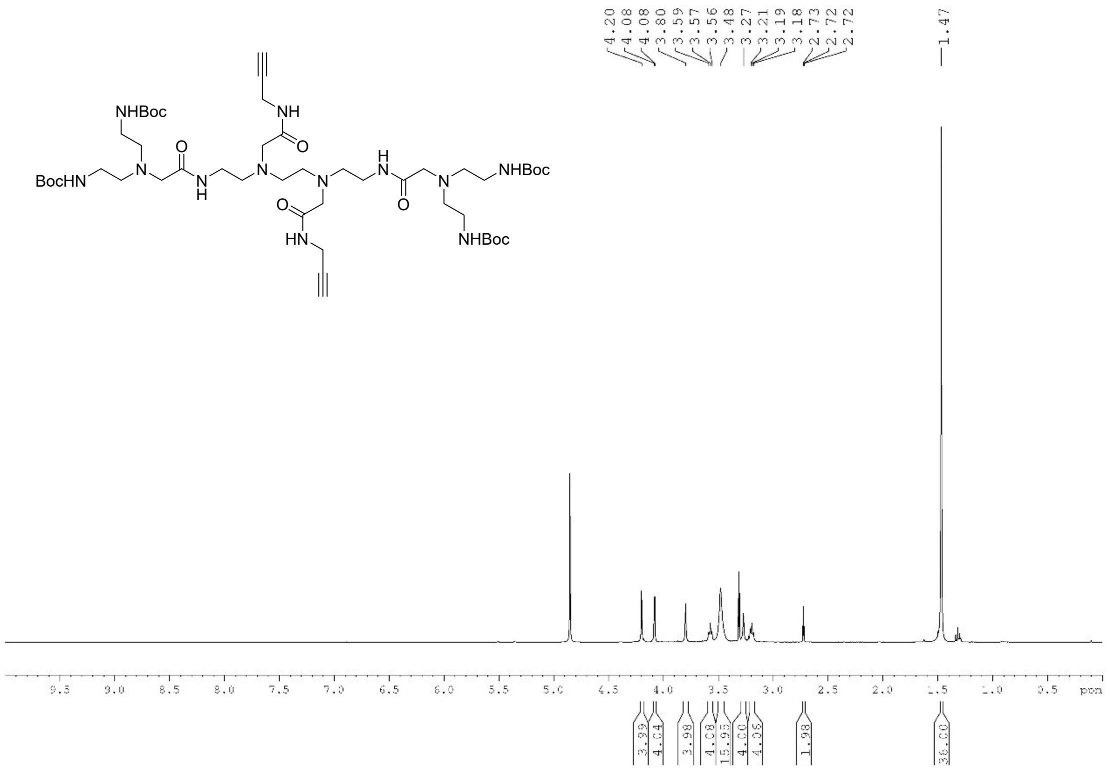

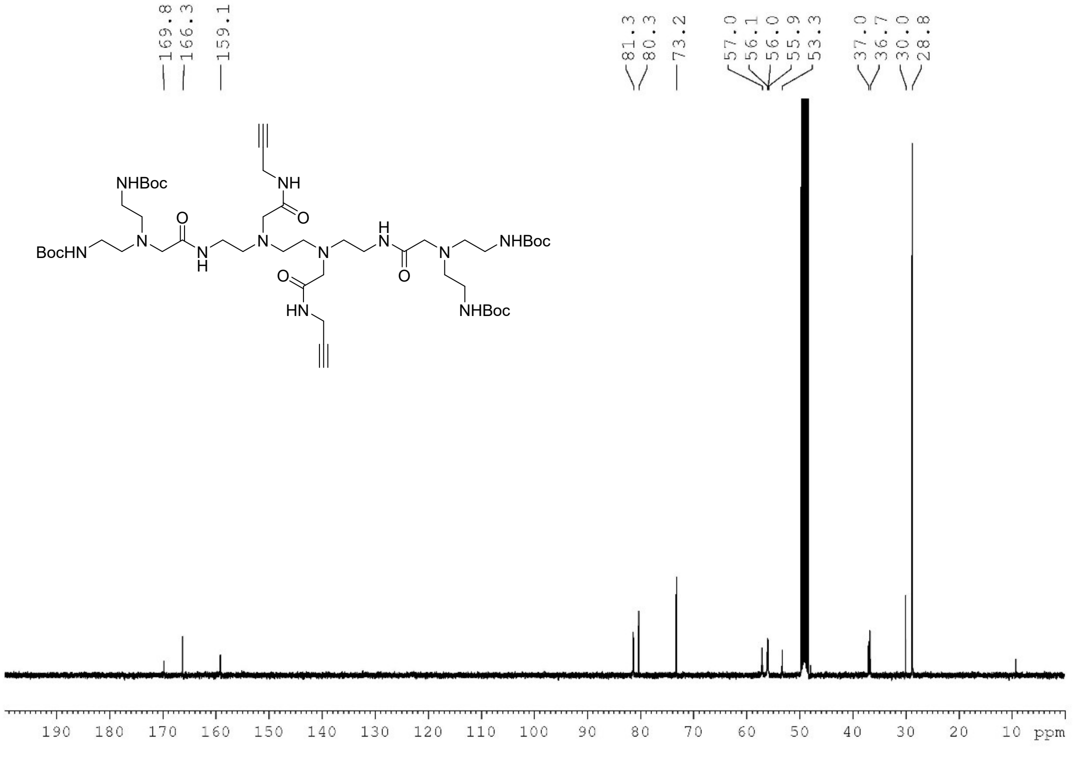

TetraDVP-(alkyne)$_2$ (7)

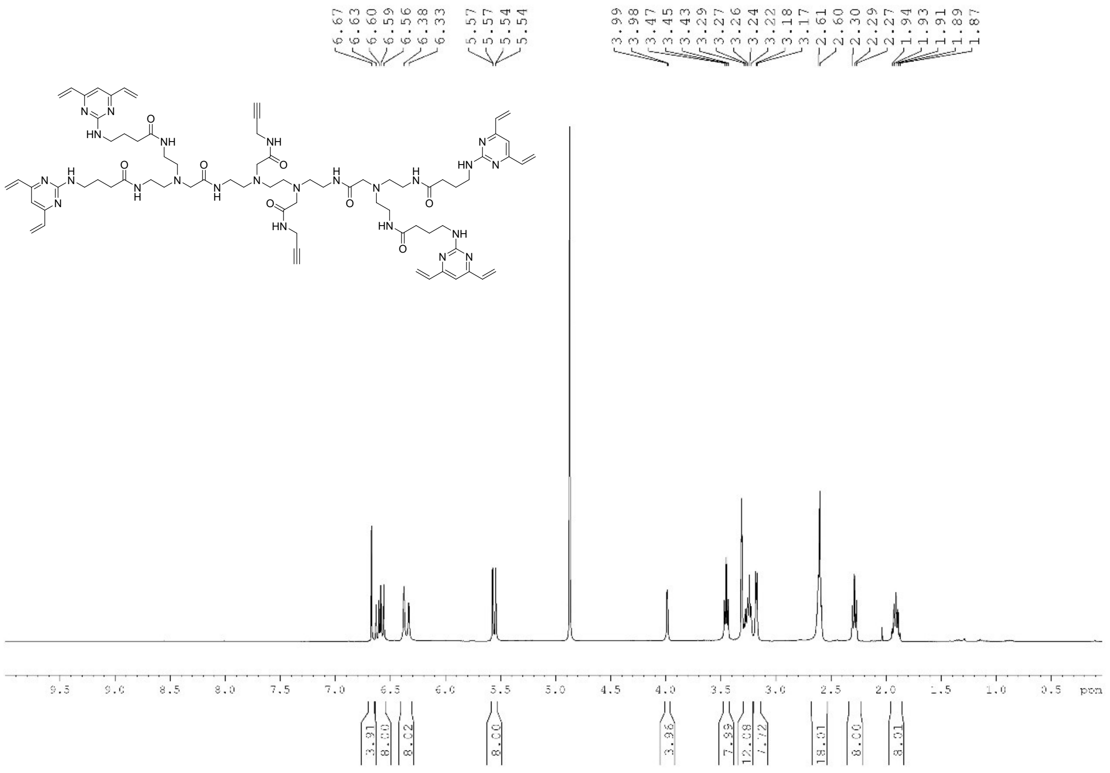

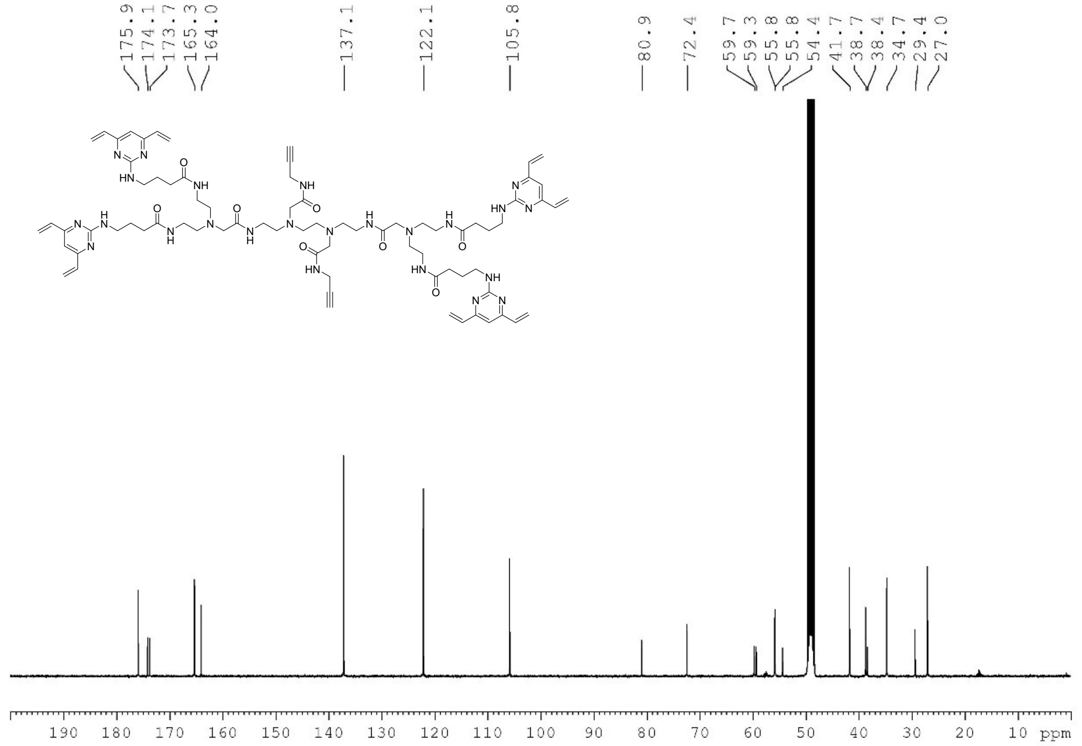

Tetra-*N*-Boc-amine backbone, $(NH)_3$ (31)

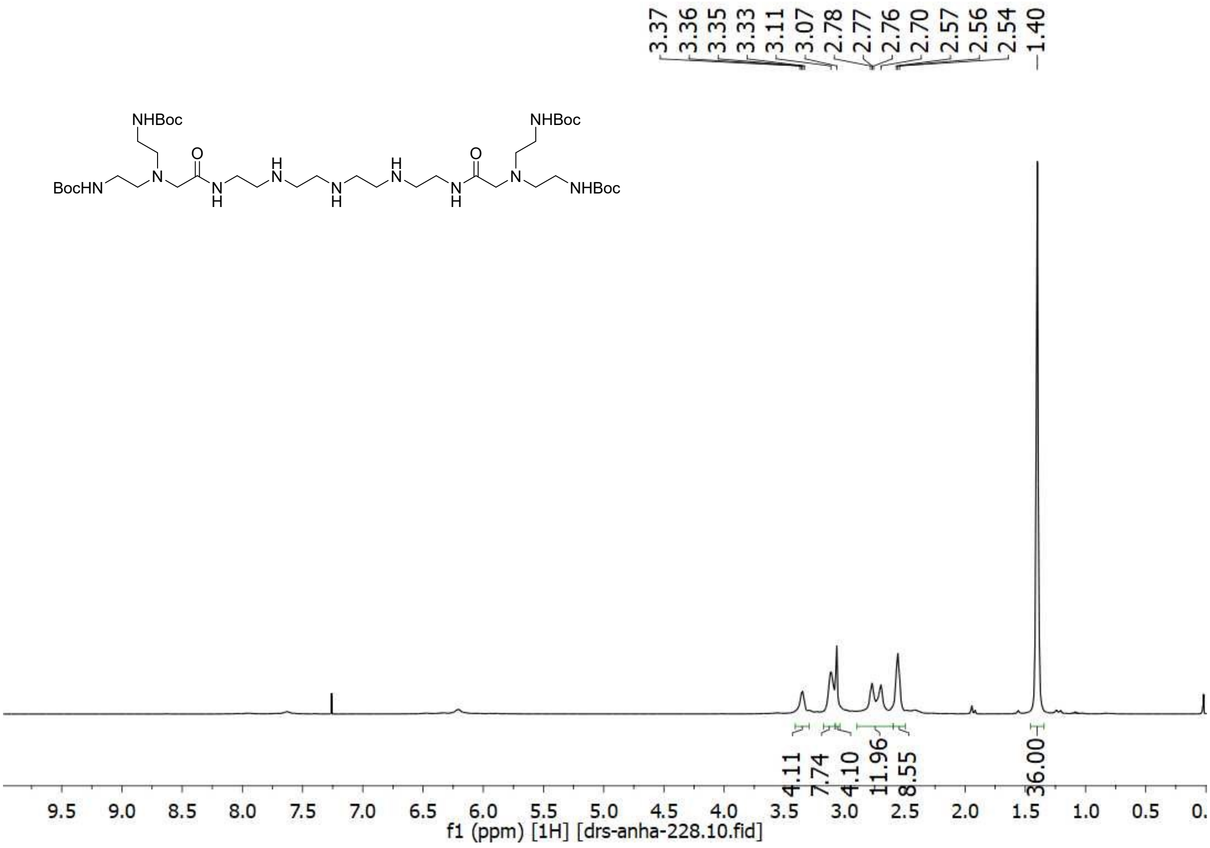

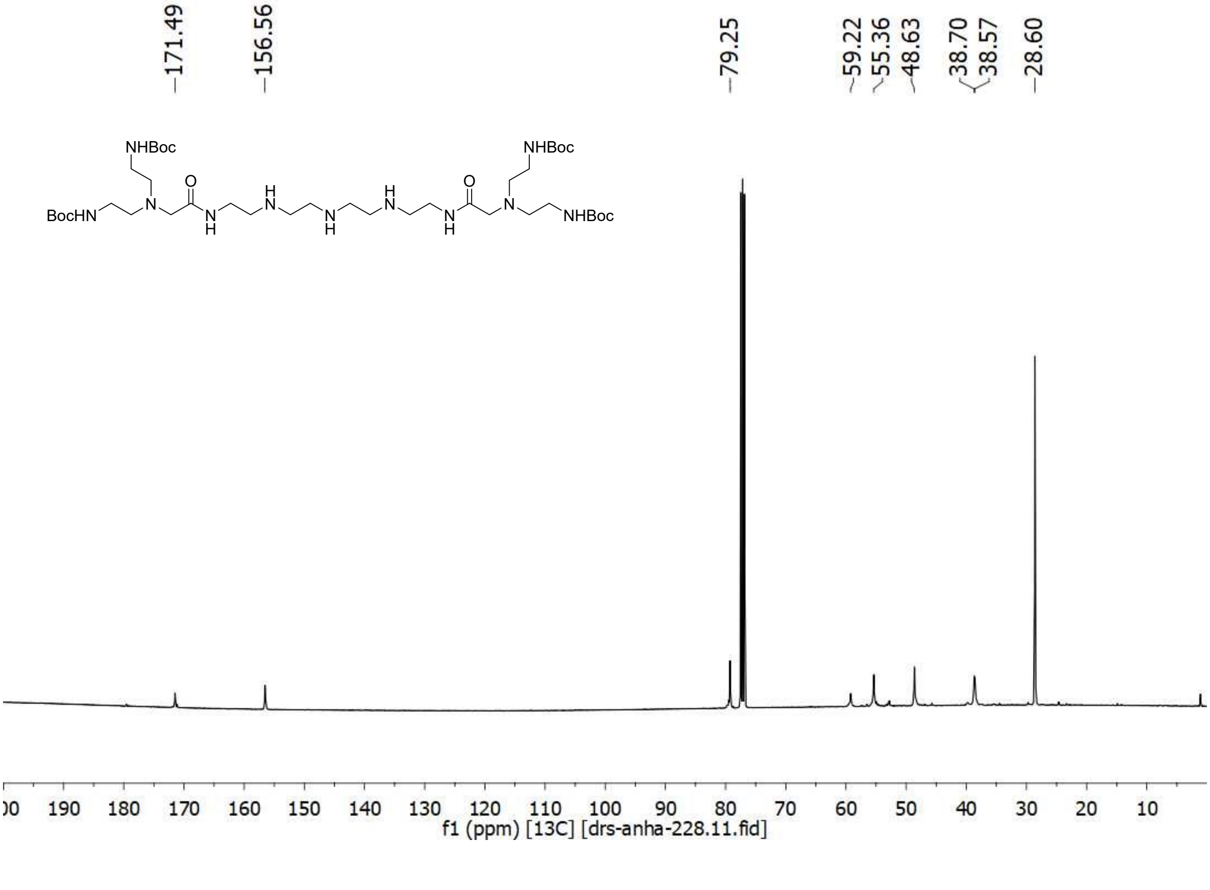

Tetra-*N*-Boc-amine backbone, (alkyne)$_3$ (32)

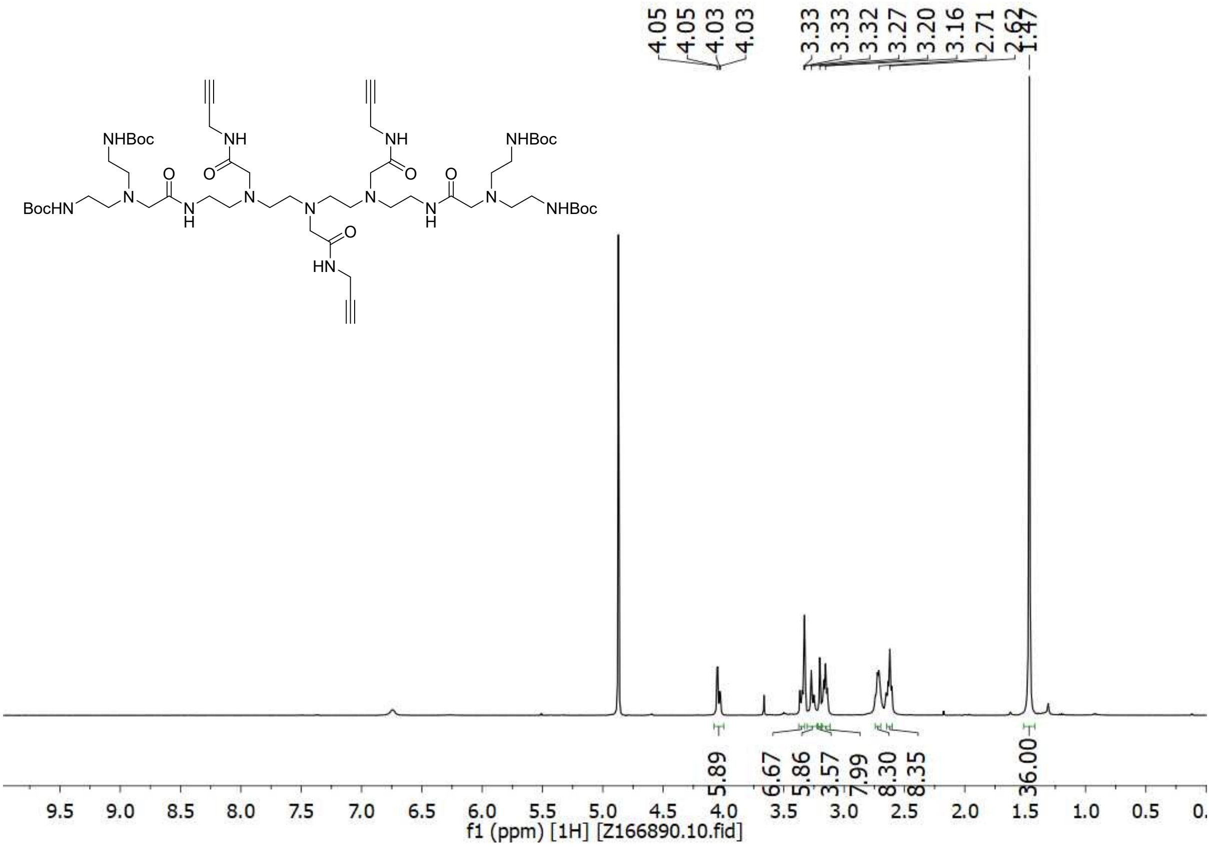

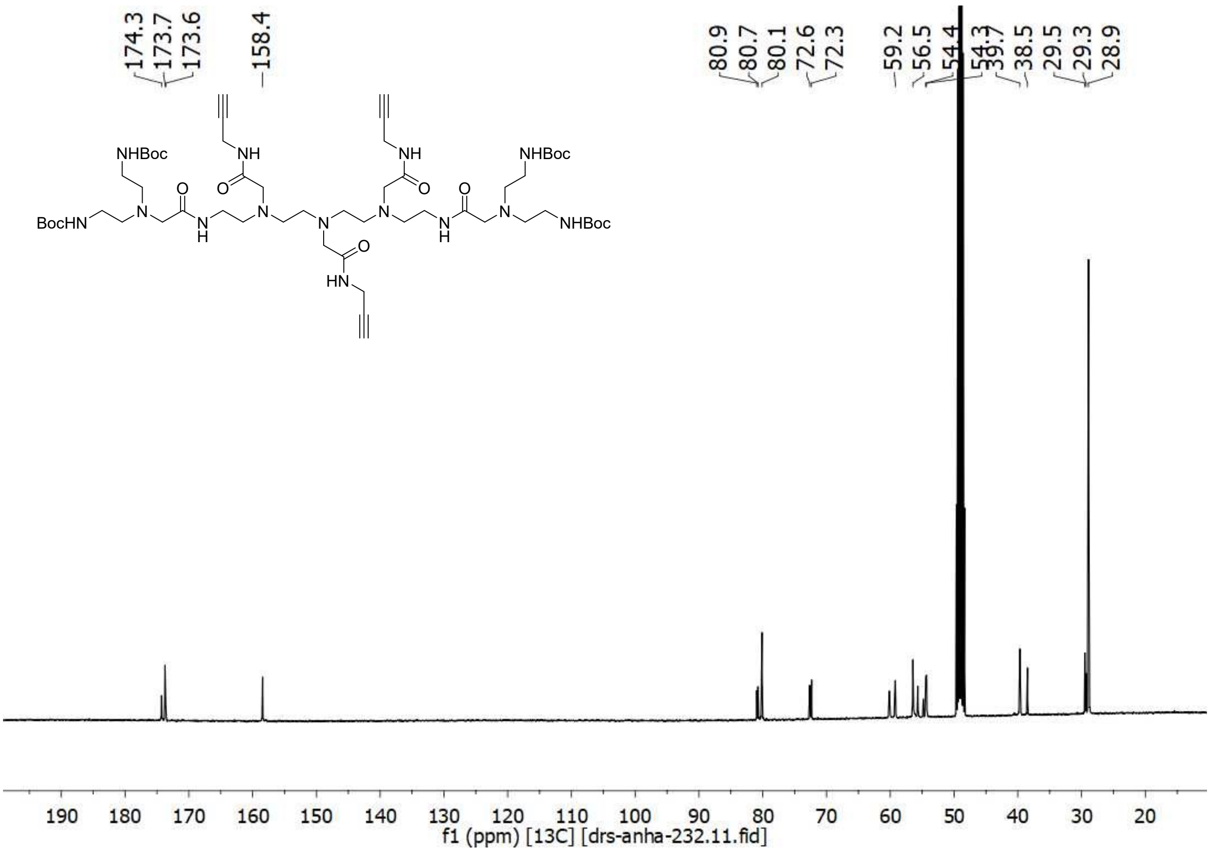

TetraDVP-(alkyne)$_3$ (8)

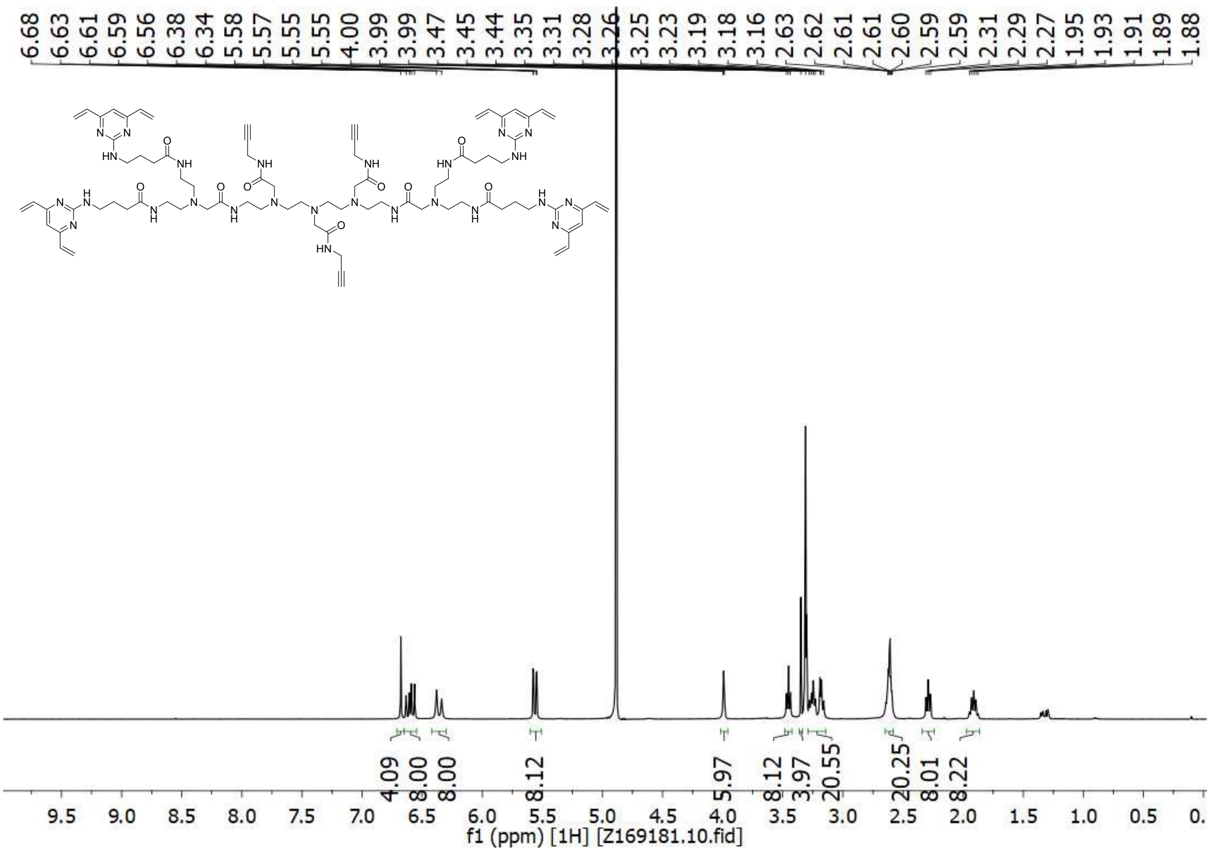

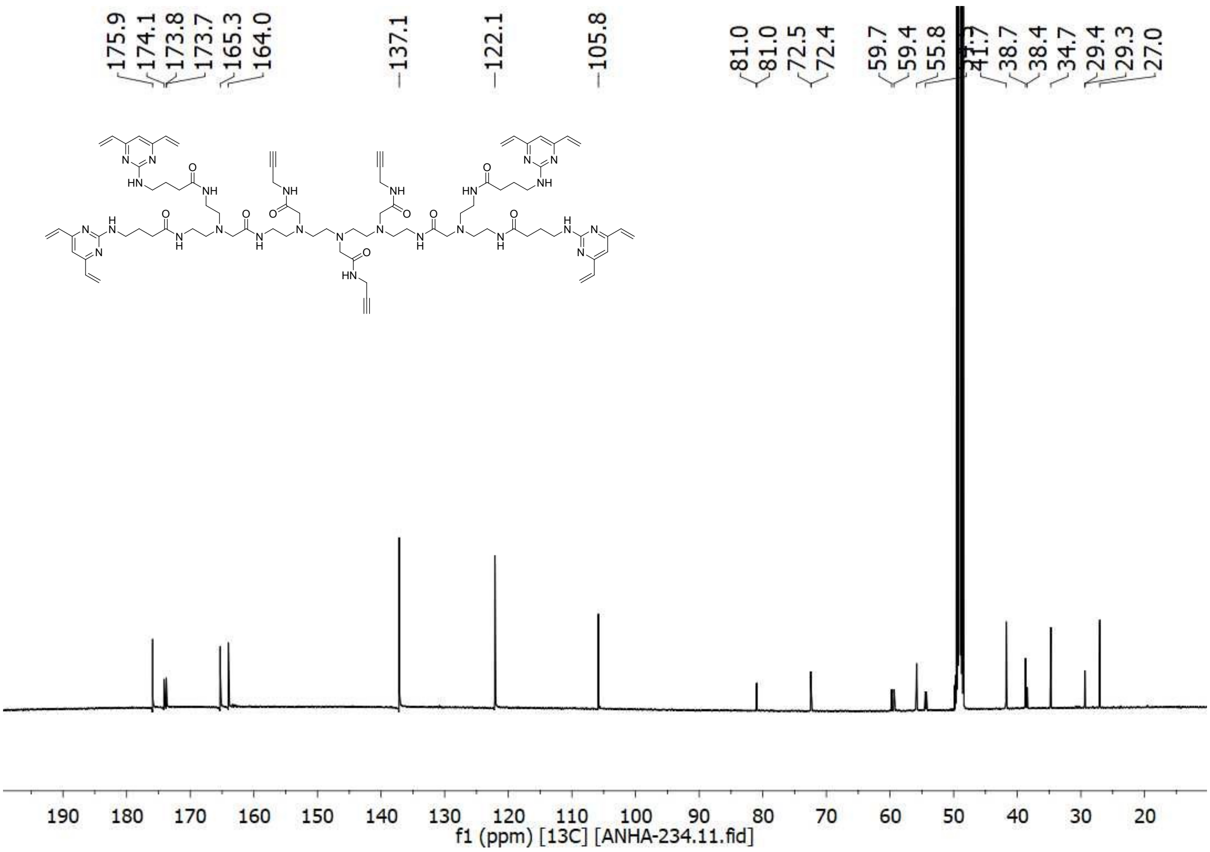

***tert*-butyl bis(2-(prop-2-yn-1-yloxy)ethyl)carbamate (33)**

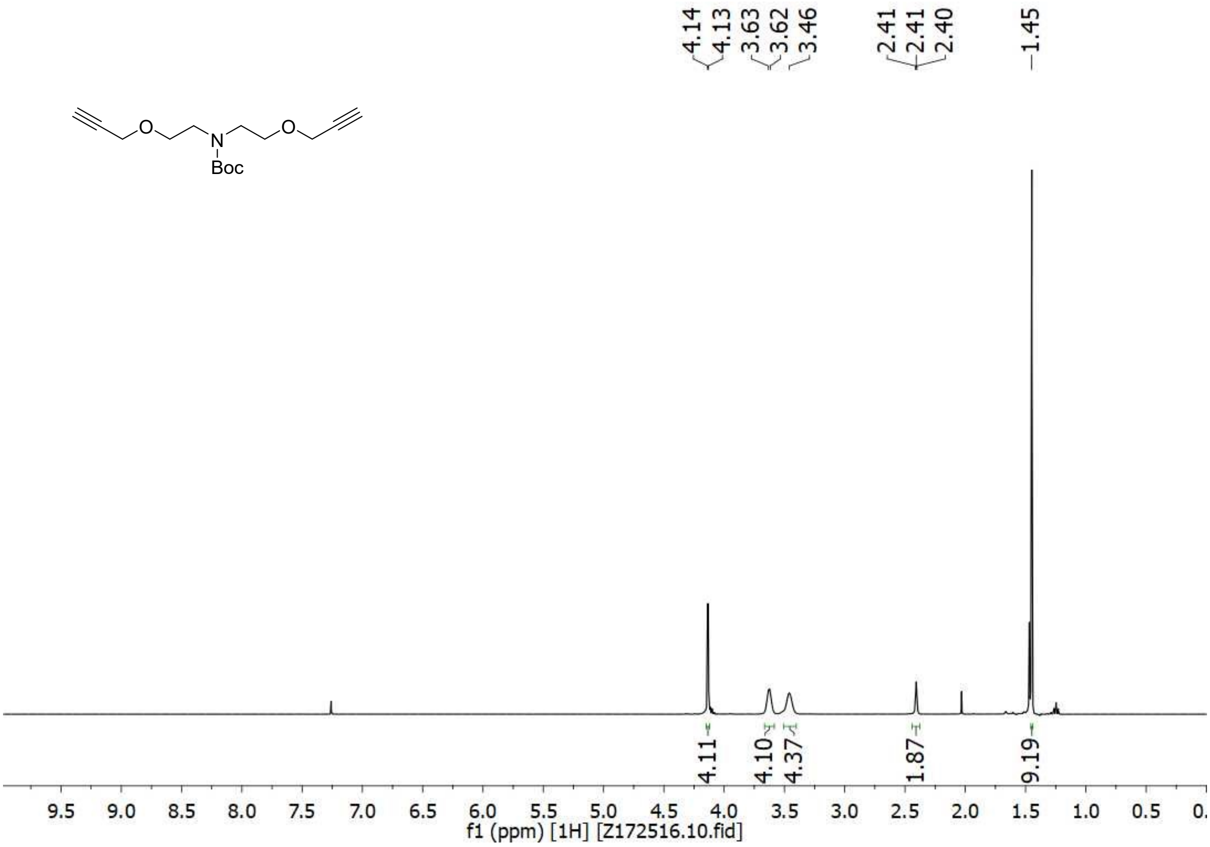

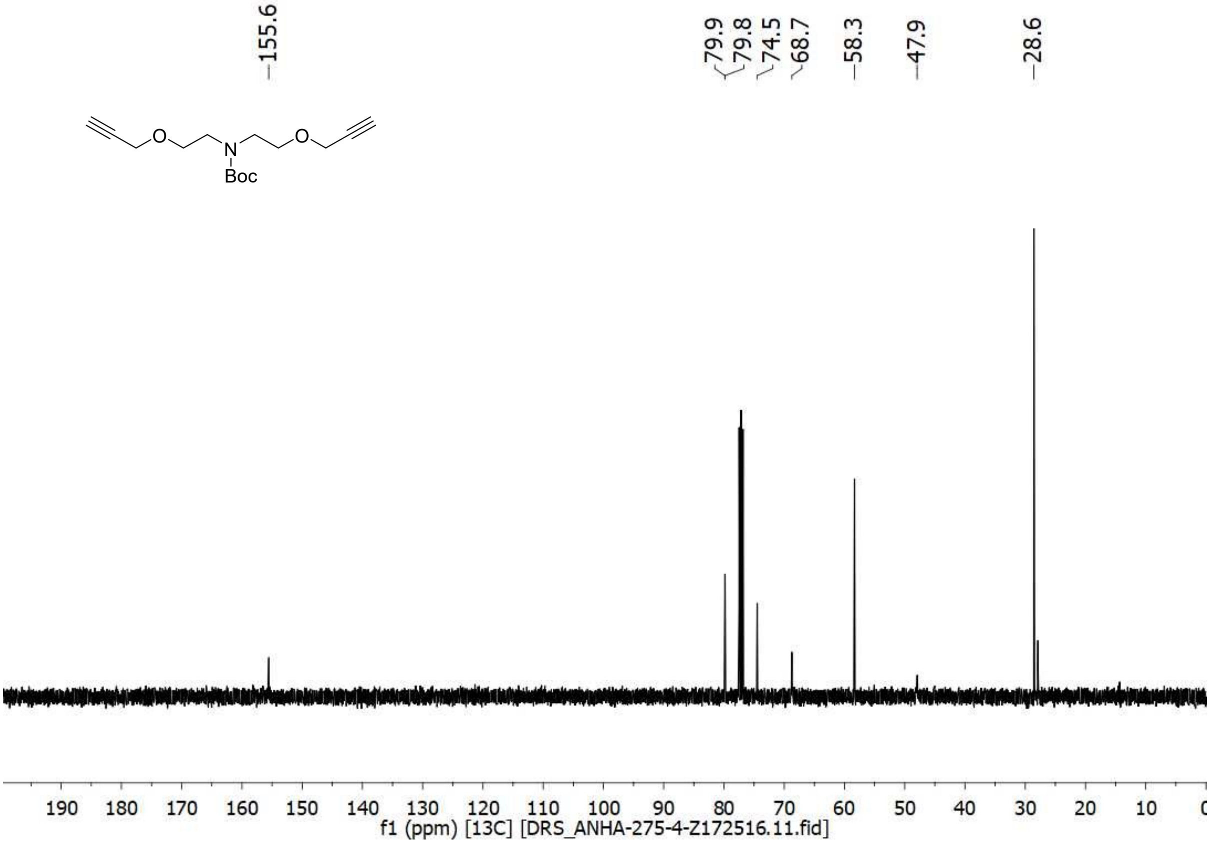

***tert*-butyl (5-(bis(2-(prop-2-yn-1-yloxy)ethyl)amino)pentyl)carbamate (34)**

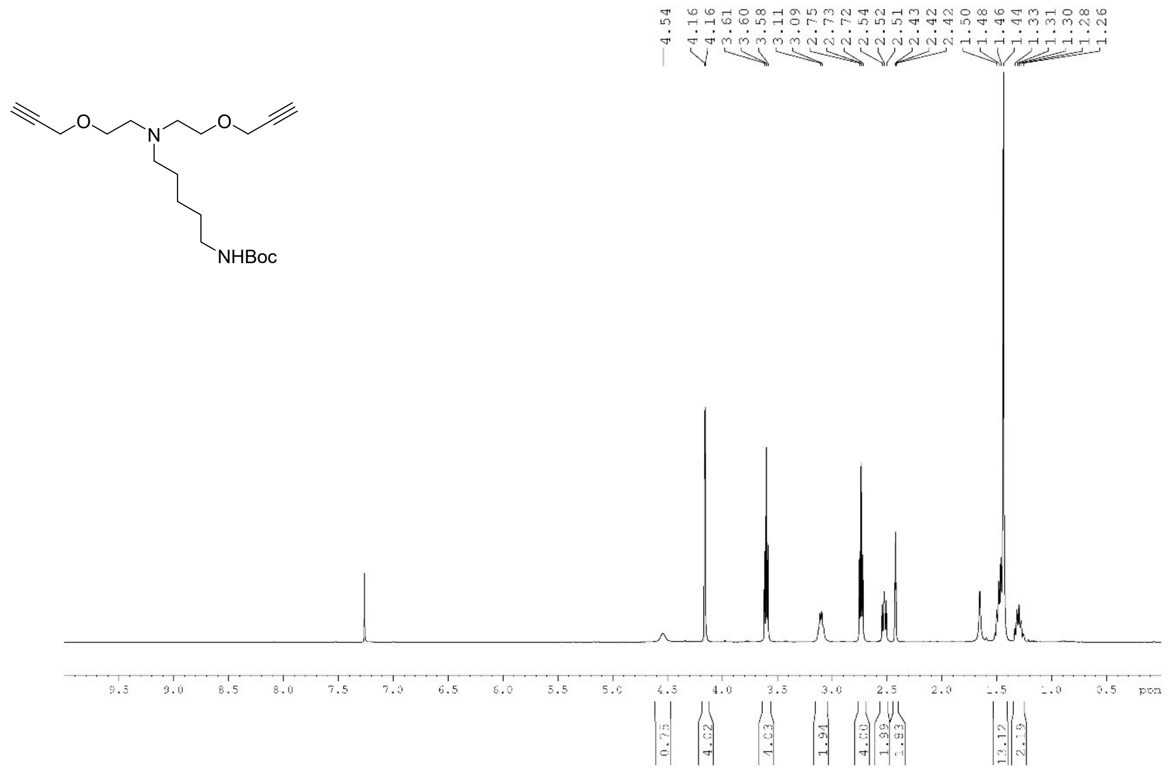

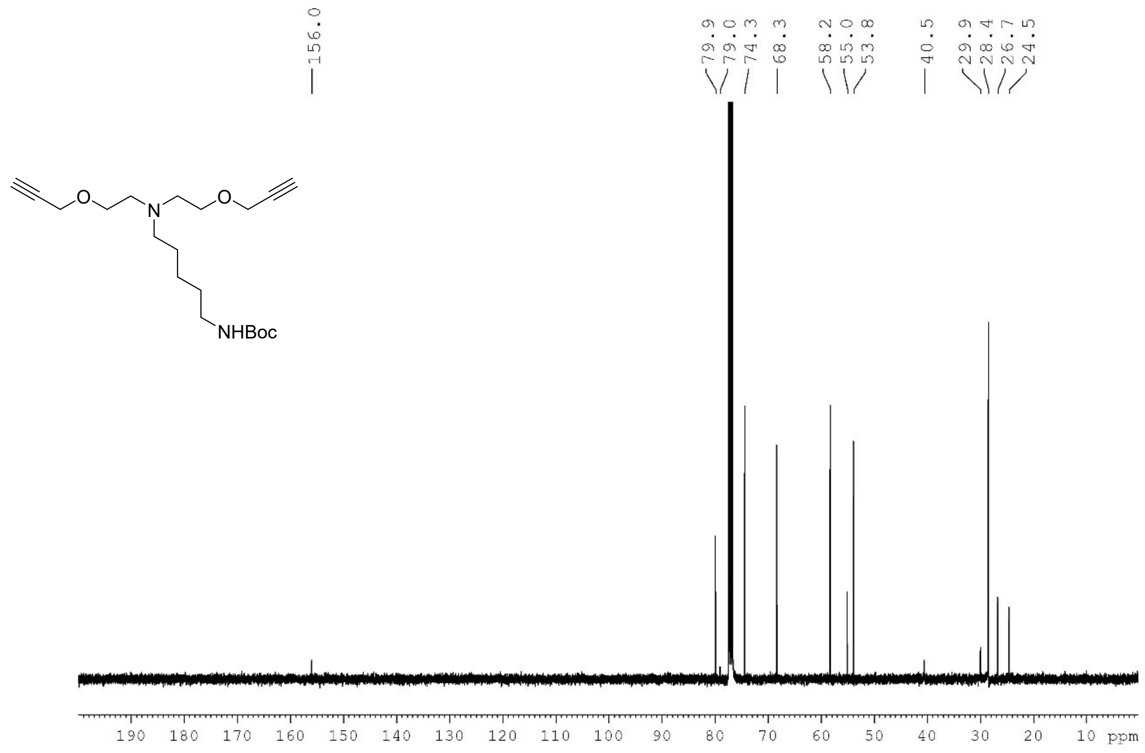

***N*-(5-(bis(2-(prop-2-yn-1-yloxy)ethyl)amino)pentyl)-2-bromoacetamide (35)**

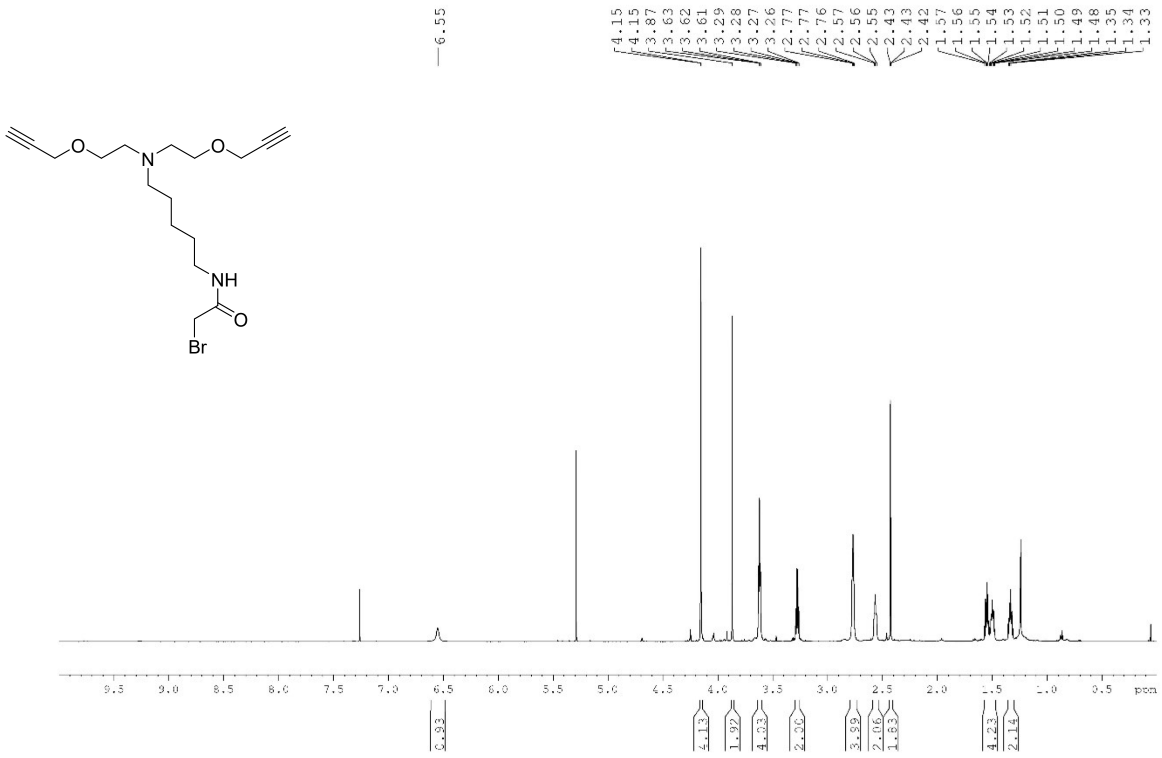

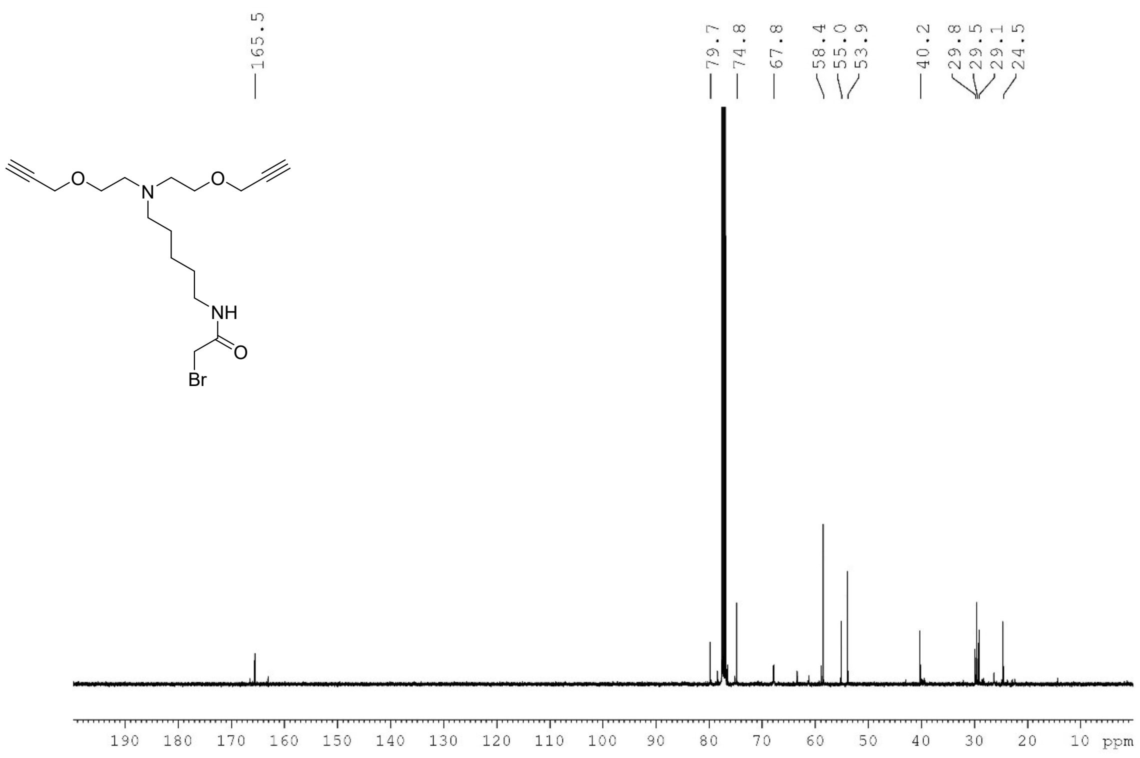

Tetra-*N*-Boc-amine backbone, (alkyne)$_4$ (36)

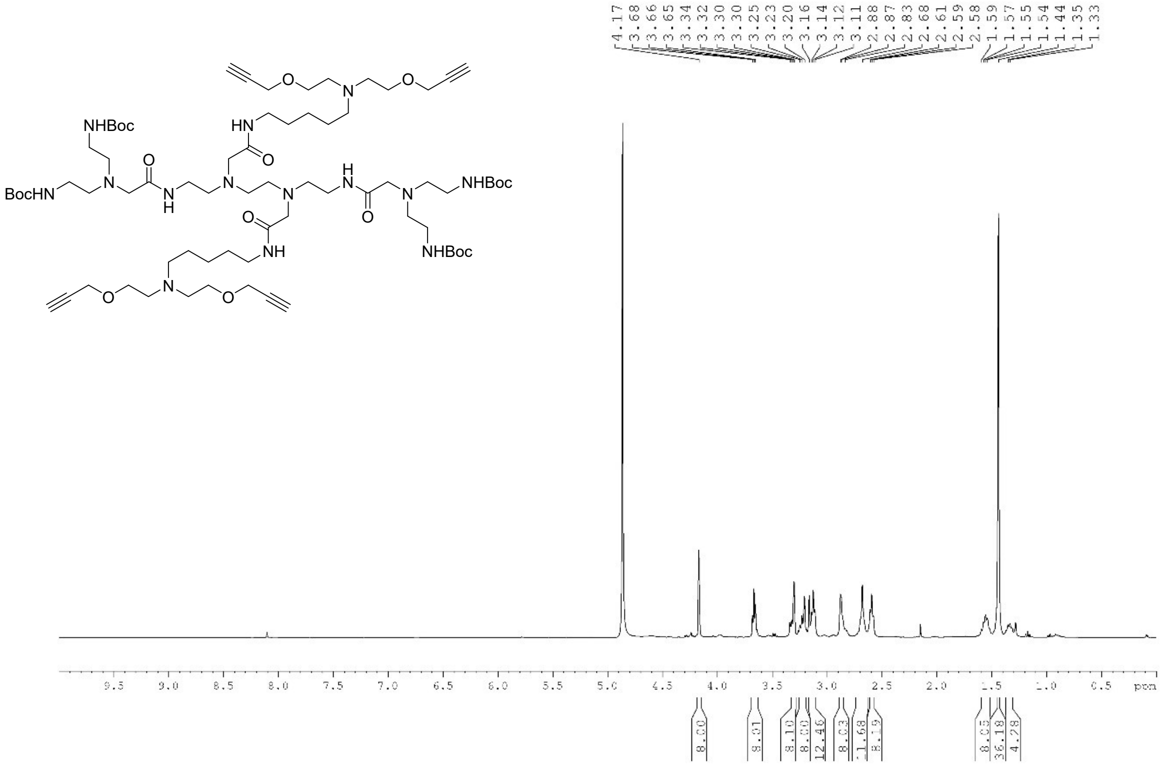

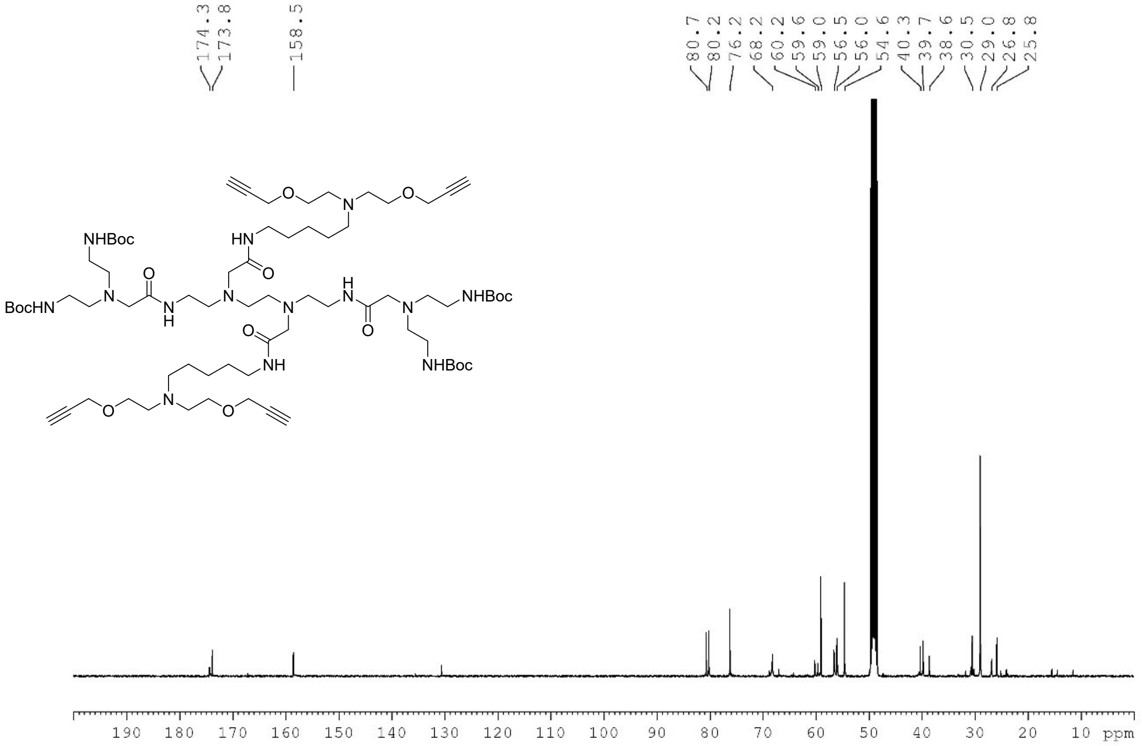

TetraDVP-(alkyne)$_4$ (9)

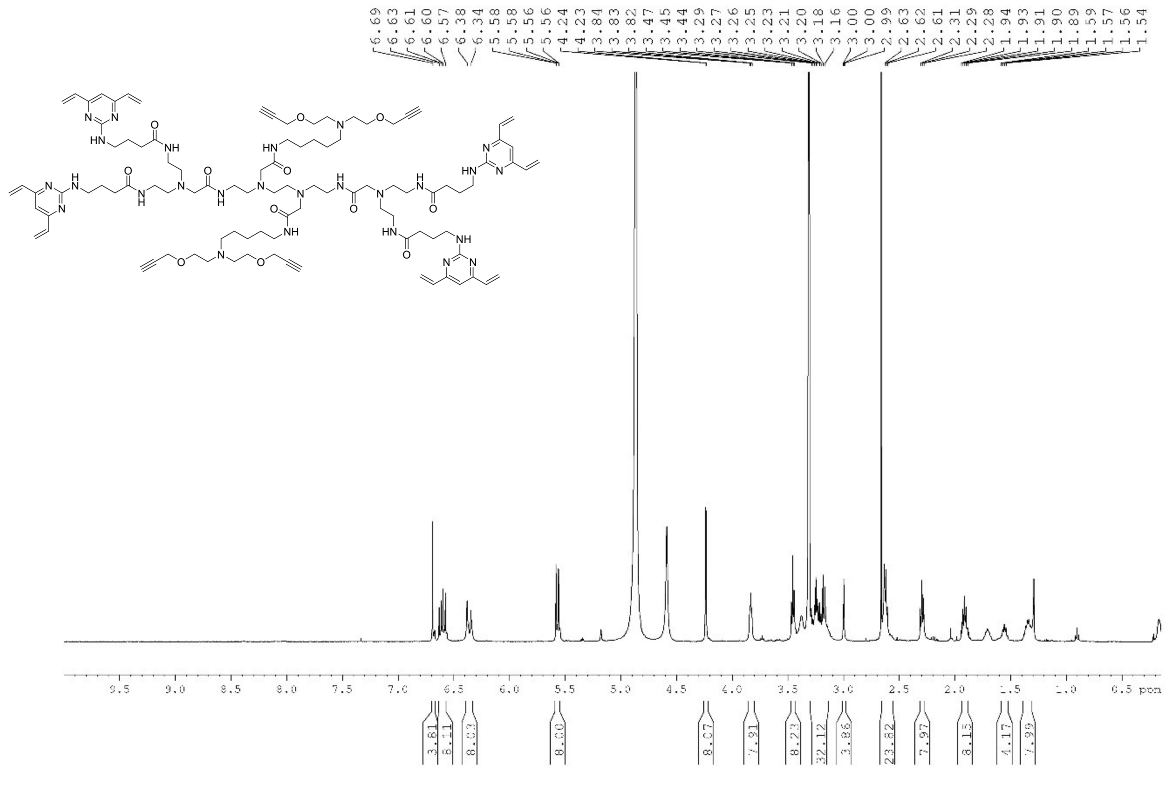
6.69
6.63
6.61
6.60
6.57
6.38
6.34
5.58
5.58
5.56
5.56
4.24
4.23
3.84
3.83
3.82
3.47
3.45
3.44
3.29
3.27
3.26
3.25
3.23
3.21
3.20
3.18
3.16
3.00
2.99
2.63
2.62
2.61
2.31
2.29
2.28
1.94
1.93
1.91
1.90
1.89
1.59
1.57
1.56
1.54
ppm

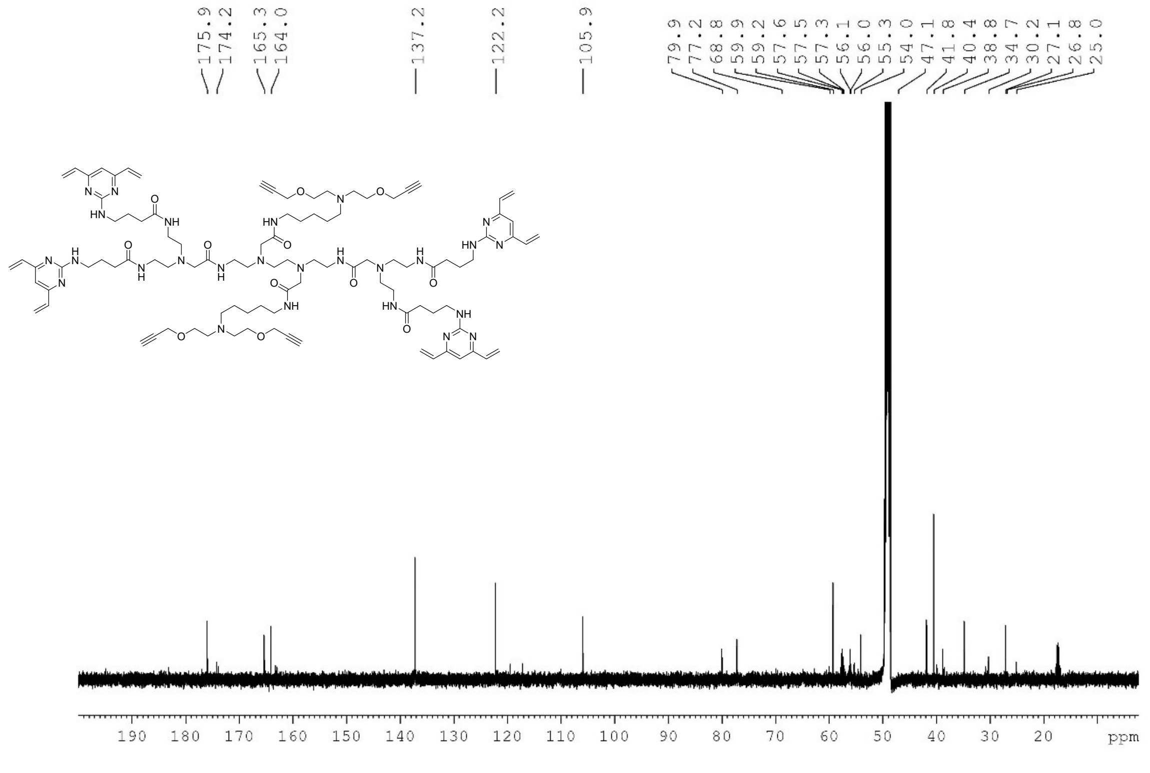
175.9
174.2
165.3
164.0
137.2
122.2
105.9
79.9
77.2
68.8
59.9
59.2
57.6
57.5
57.3
56.1
56.0
55.3
54.0
47.1
41.8
40.4
38.8
34.7
30.2
27.1
26.8
25.0
190 180 170 160 150 140 130 120 110 100 90 80 70 60 50 40 30 20 ppm

HPLC traces

Fmoc-Val-Cit-PABC-MMAE (37)

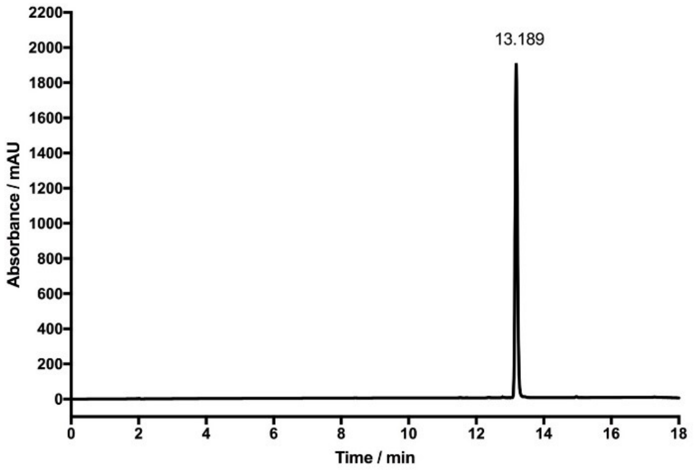

N_3-PEG_4-Val-Cit-PABC-MMAE (38)

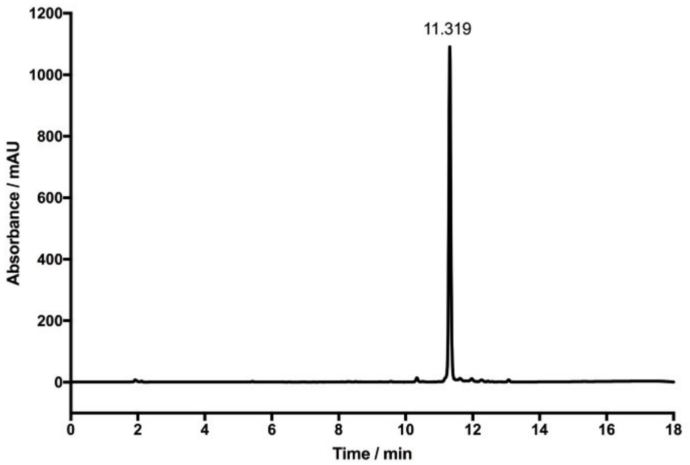

HDX uptake plots

Light chain

T = trastuzumab, T+tetraDVP = TetraDVP conjugate **6**, T+DVP = DVP conjugate **4**

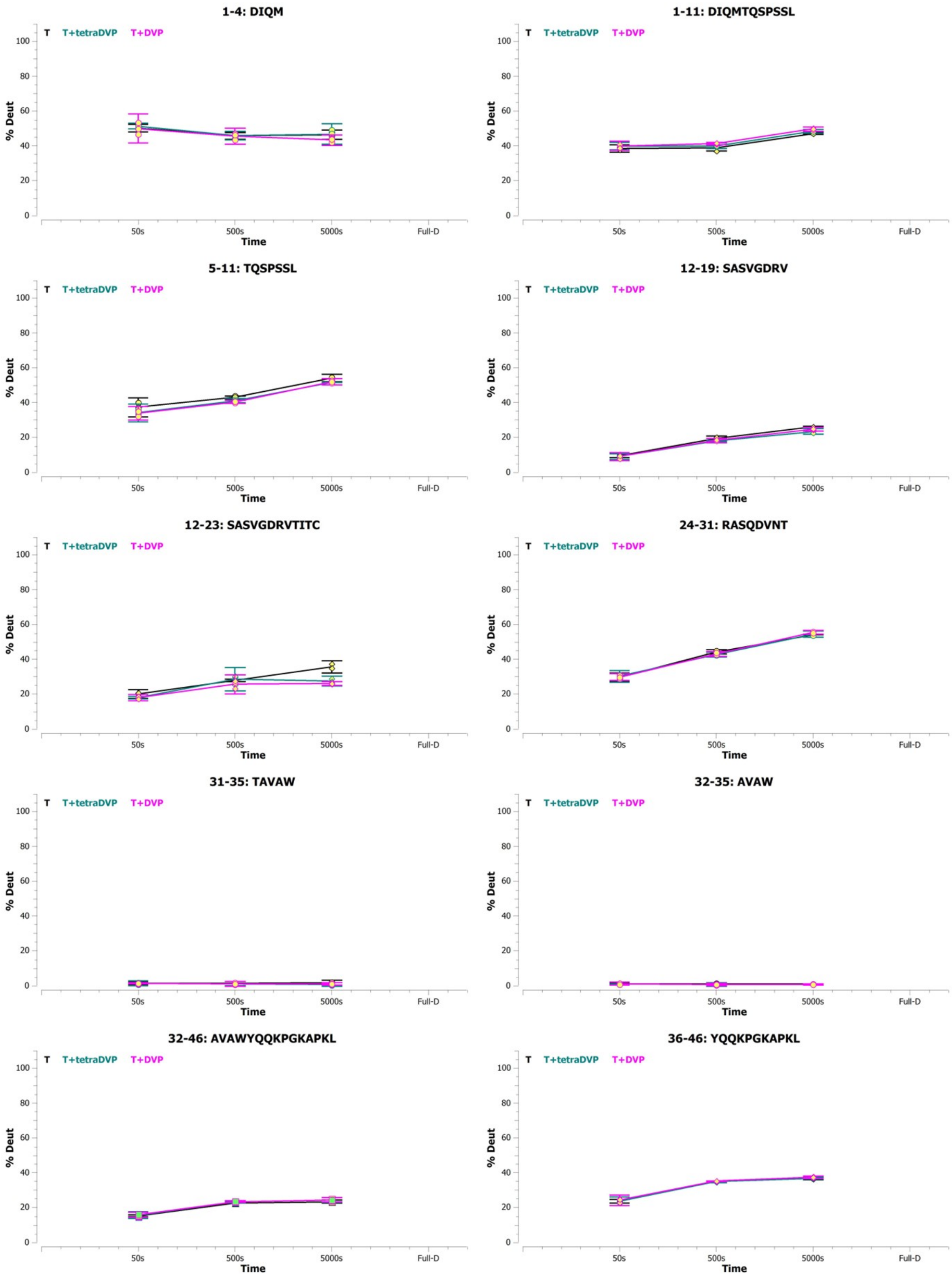

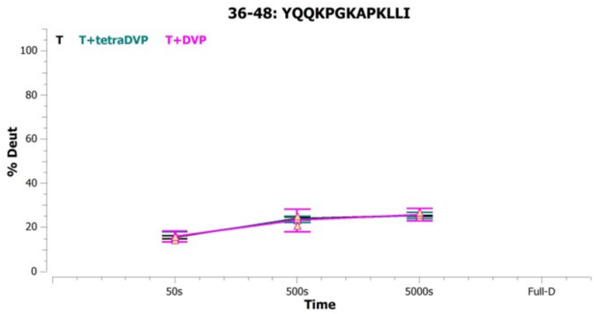
36-48: YQQKPGKAPKLLI
T T+tetraDVP T+DVP
% Deut
0 20 40 60 80 100
50s 500s 5000s Full-D
Time

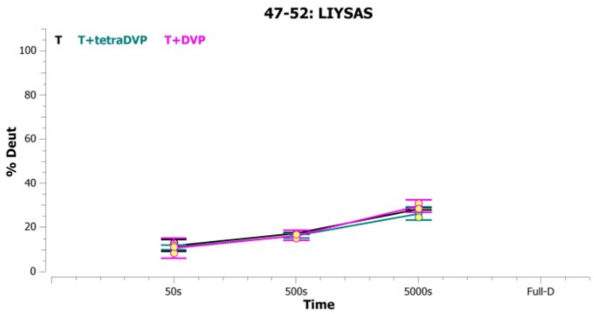
47-52: LIYSAS
T T+tetraDVP T+DVP
% Deut
0 20 40 60 80 100
50s 500s 5000s Full-D
Time

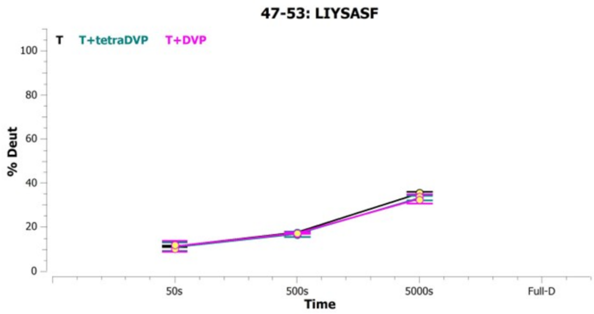
47-53: LIYSASF
T T+tetraDVP T+DVP
% Deut
0 20 40 60 80 100
50s 500s 5000s Full-D
Time

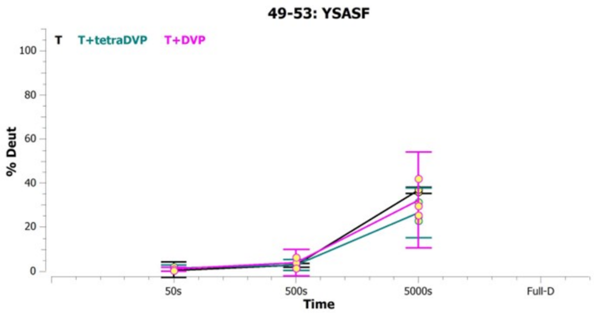
49-53: YSASF
T T+tetraDVP T+DVP
% Deut
0 20 40 60 80 100
50s 500s 5000s Full-D
Time

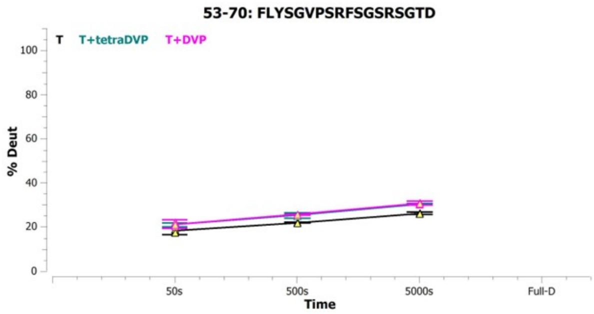
53-70: FLYSGVPSRFSGSRSGTD
T T+tetraDVP T+DVP
% Deut
0 20 40 60 80 100
50s 500s 5000s Full-D
Time

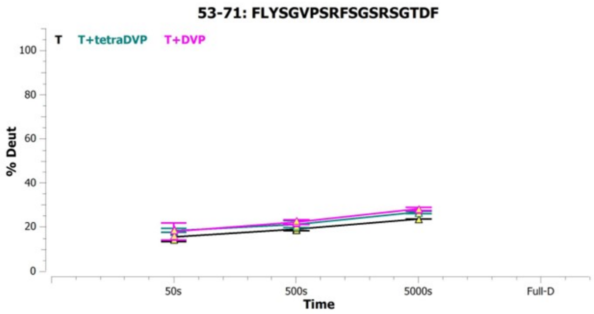
53-71: FLYSGVPSRFSGSRSGTDF
T T+tetraDVP T+DVP
% Deut
0 20 40 60 80 100
50s 500s 5000s Full-D
Time

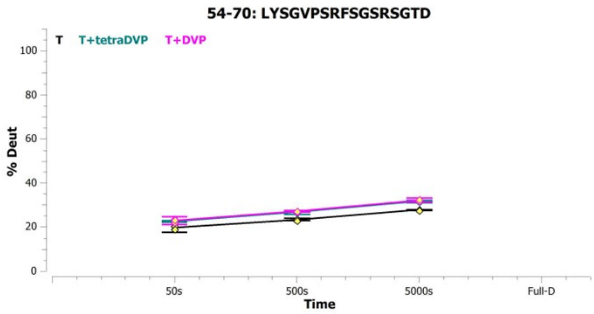
54-70: LYSGVPSRFSGSRSGTD
T T+tetraDVP T+DVP
% Deut
0 20 40 60 80 100
50s 500s 5000s Full-D
Time

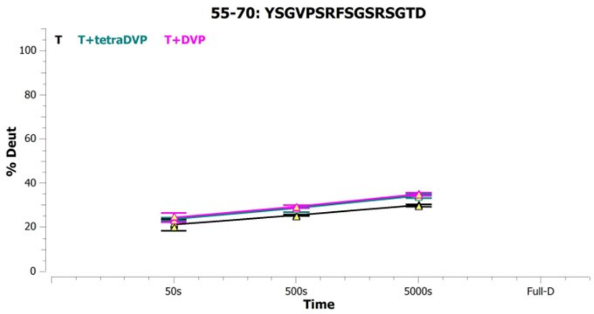
55-70: YSGVPSRFSGSRSGTD
T T+tetraDVP T+DVP
% Deut
0 20 40 60 80 100
50s 500s 5000s Full-D
Time

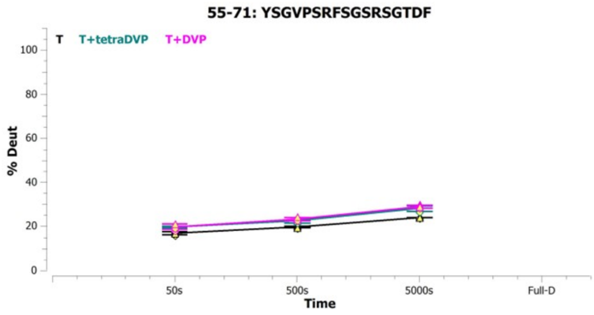
55-71: YSGVPSRFSGSRSGTDF
T T+tetraDVP T+DVP
% Deut
0 20 40 60 80 100
50s 500s 5000s Full-D
Time

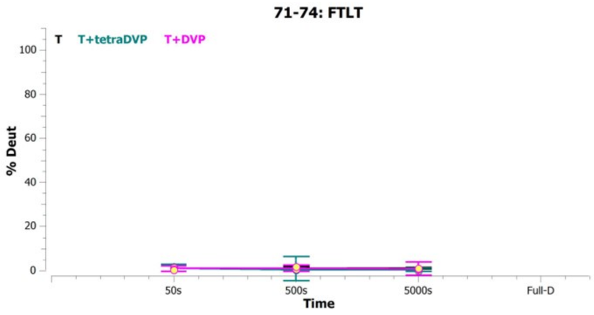
71-74: FTLT
T T+tetraDVP T+DVP
% Deut
0 20 40 60 80 100
50s 500s 5000s Full-D
Time

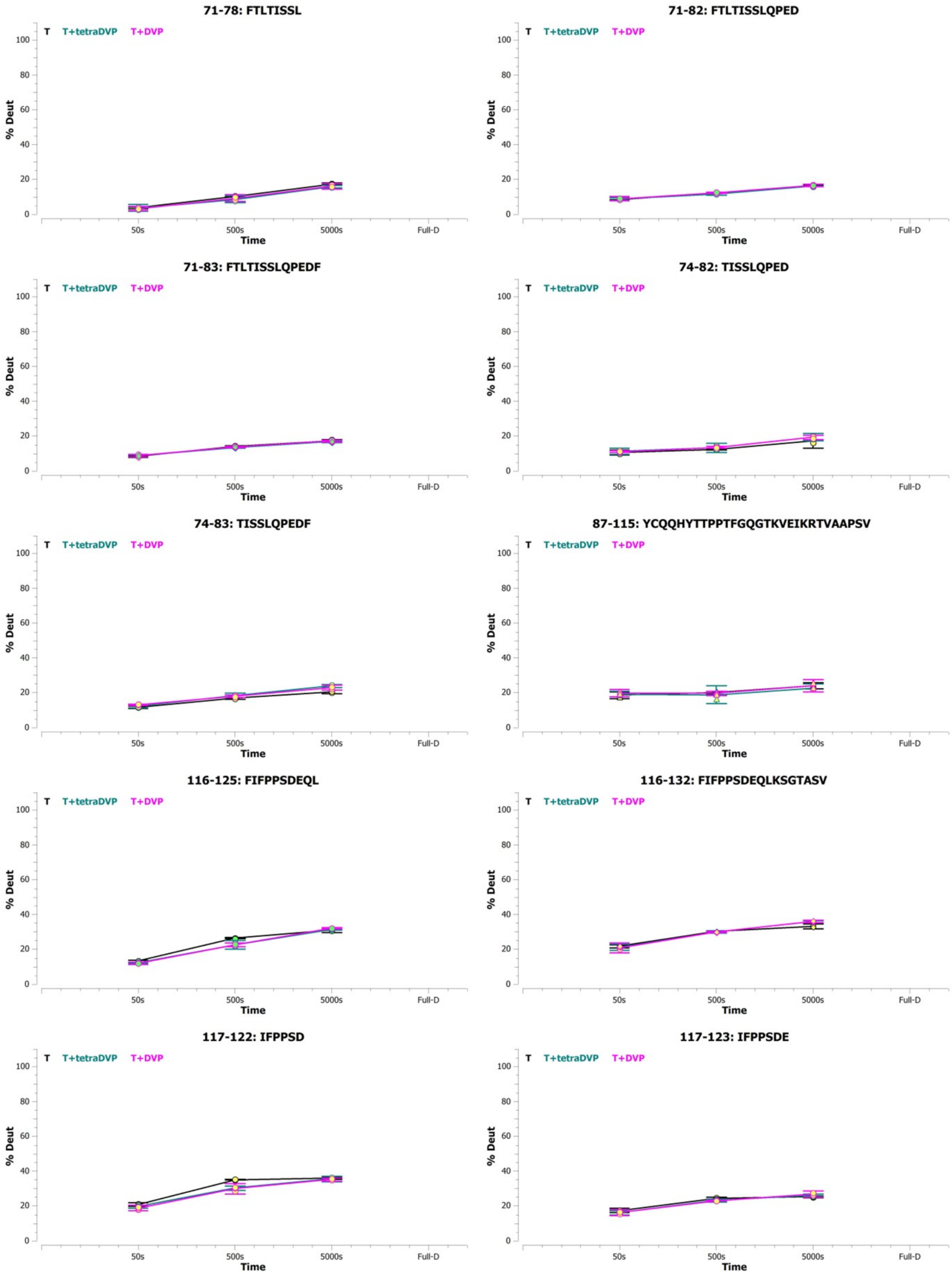
71-78: FTLTISSL
71-82: FTLTISSLQPED
71-83: FTLTISSLQPEDF
74-82: TISSLQPED
74-83: TISSLQPEDF
87-115: YCQQHYTTPPTFGQGTKVEIKRTVAAPSV
116-125: FIFPPSDEQL
116-132: FIFPPSDEQLKSGTASV
117-122: IFPPSD
117-123: IFPPSDE
T
T+tetraDVP
T+DVP
% Deut
Time
50s
500s
5000s
Full-D

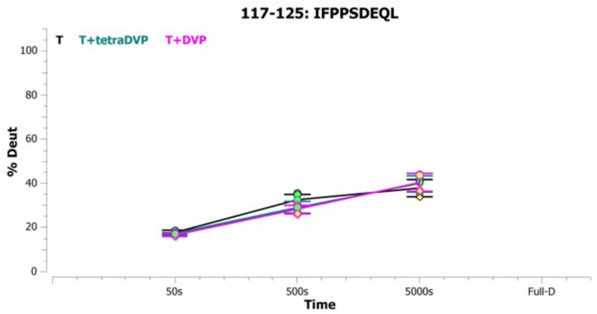
117-125: IFPPSDEQL
T T+tetraDVP T+DVP
% Deut
0 20 40 60 80 100
50s 500s 5000s Full-D
Time

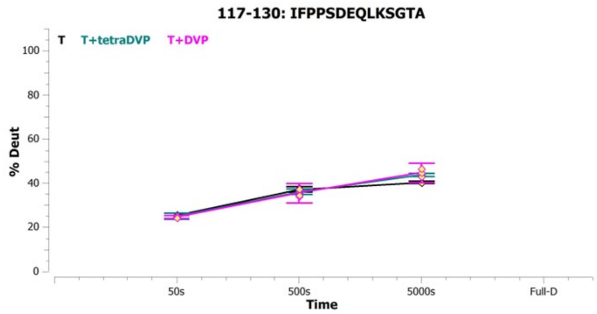
117-130: IFPPSDEQLKSGTA
T T+tetraDVP T+DVP
% Deut
0 20 40 60 80 100
50s 500s 5000s Full-D
Time

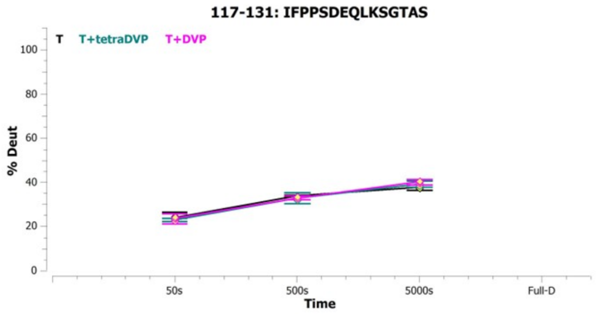
117-131: IFPPSDEQLKSGTAS
T T+tetraDVP T+DVP
% Deut
0 20 40 60 80 100
50s 500s 5000s Full-D
Time

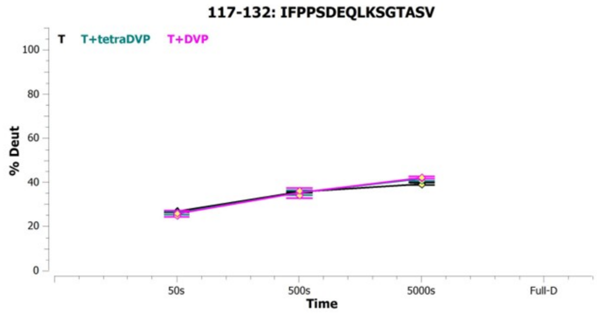
117-132: IFPPSDEQLKSGTASV
T T+tetraDVP T+DVP
% Deut
0 20 40 60 80 100
50s 500s 5000s Full-D
Time

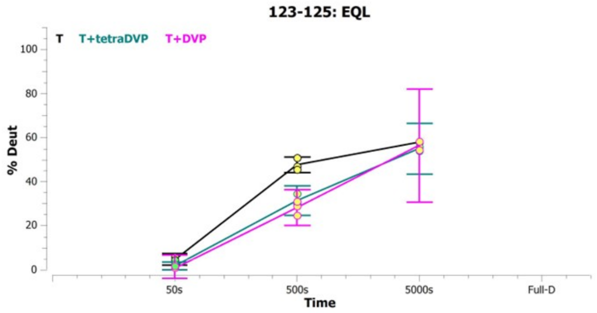
123-125: EQL
T T+tetraDVP T+DVP
% Deut
0 20 40 60 80 100
50s 500s 5000s Full-D
Time

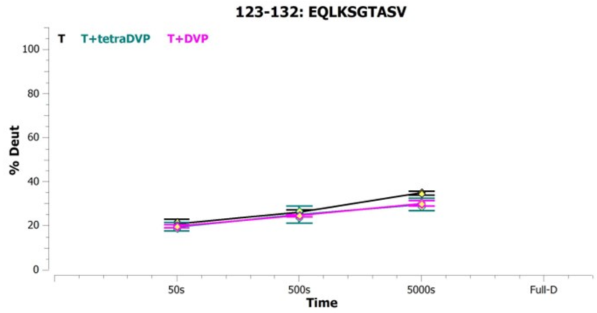
123-132: EQLKSGTASV
T T+tetraDVP T+DVP
% Deut
0 20 40 60 80 100
50s 500s 5000s Full-D
Time

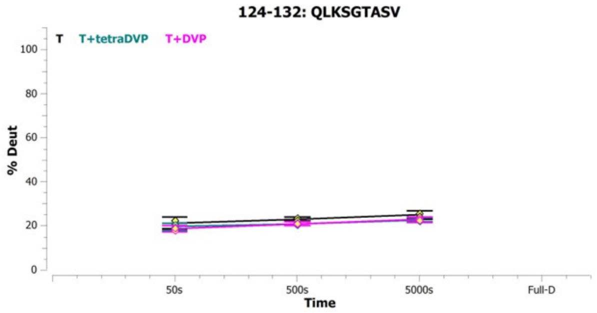
124-132: QLKSGTASV
T T+tetraDVP T+DVP
% Deut
0 20 40 60 80 100
50s 500s 5000s Full-D
Time

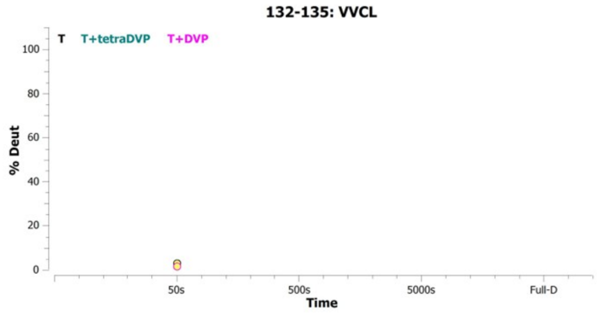
132-135: VVCL
T T+tetraDVP T+DVP
% Deut
0 20 40 60 80 100
50s 500s 5000s Full-D
Time

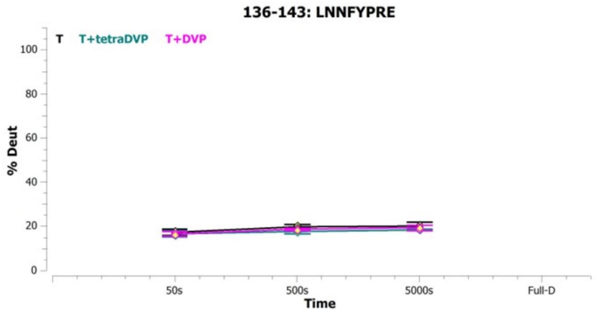
136-143: LNNFYPRE
T T+tetraDVP T+DVP
% Deut
0 20 40 60 80 100
50s 500s 5000s Full-D
Time

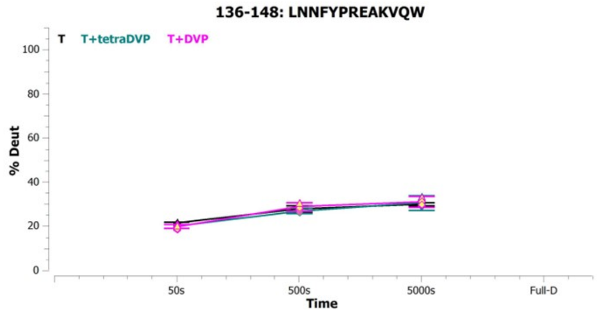
136-148: LNNFYPREAKVQW
T T+tetraDVP T+DVP
% Deut
0 20 40 60 80 100
50s 500s 5000s Full-D
Time

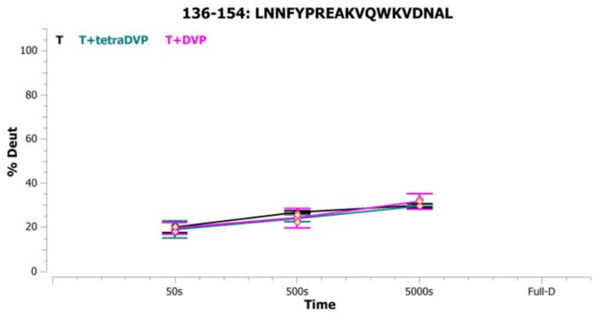
136-154: LNNFYPREAKVQWKVDNAL
T
T+tetraDVP
T+DVP
% Deut
0
20
40
60
80
100
50s
500s
5000s
Full-D
Time

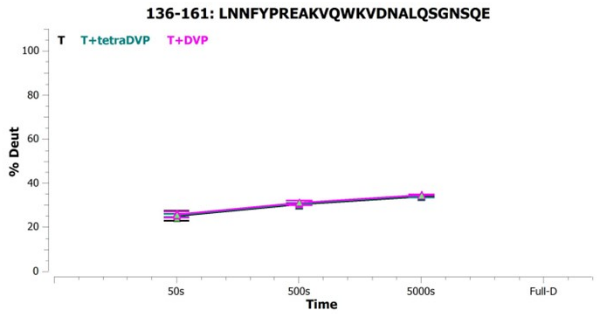
136-161: LNNFYPREAKVQWKVDNALQSGNSQE
T
T+tetraDVP
T+DVP
% Deut
0
20
40
60
80
100
50s
500s
5000s
Full-D
Time

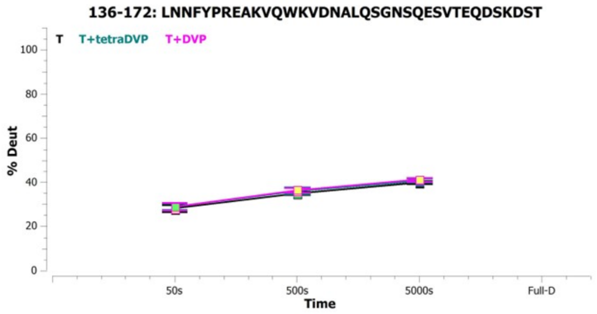
136-172: LNNFYPREAKVQWKVDNALQSGNSQESVTEQDSKDST
T
T+tetraDVP
T+DVP
% Deut
0
20
40
60
80
100
50s
500s
5000s
Full-D
Time

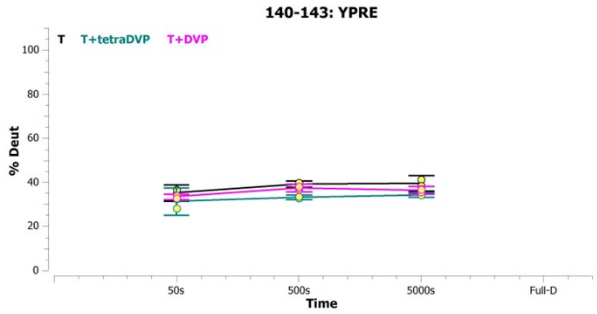
140-143: YPRE
T
T+tetraDVP
T+DVP
% Deut
0
20
40
60
80
100
50s
500s
5000s
Full-D
Time

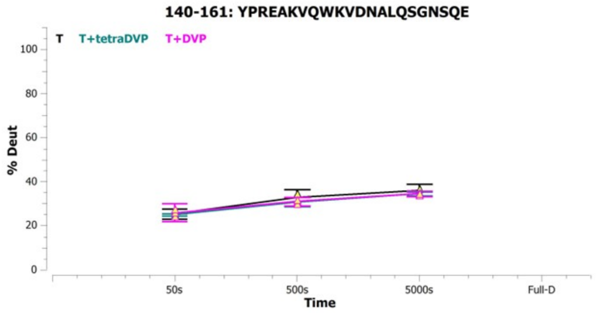
140-161: YPREAKVQWKVDNALQSGNSQE
T
T+tetraDVP
T+DVP
% Deut
0
20
40
60
80
100
50s
500s
5000s
Full-D
Time

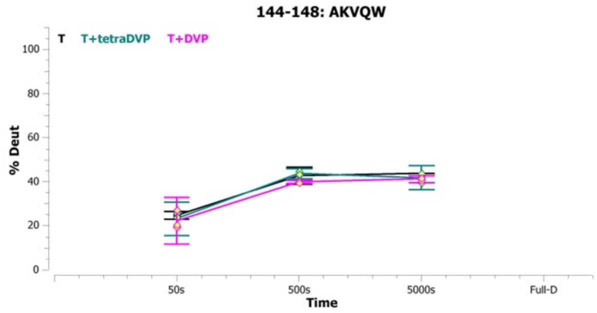
144-148: AKVQW
T
T+tetraDVP
T+DVP
% Deut
0
20
40
60
80
100
50s
500s
5000s
Full-D
Time

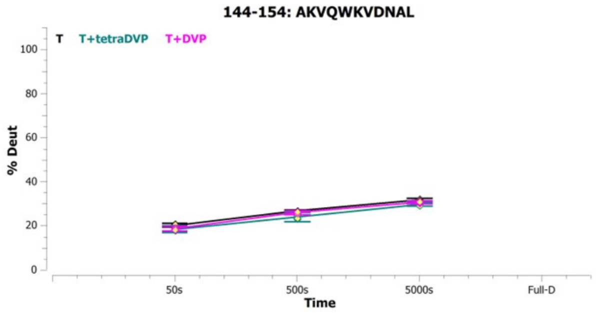
144-154: AKVQWKVDNAL
T
T+tetraDVP
T+DVP
% Deut
0
20
40
60
80
100
50s
500s
5000s
Full-D
Time

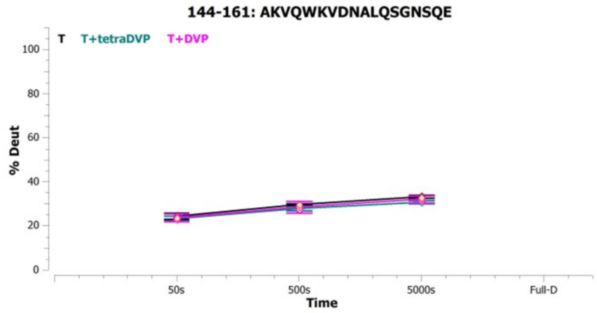
144-161: AKVQWKVDNALQSGNSQE
T
T+tetraDVP
T+DVP
% Deut
0
20
40
60
80
100
50s
500s
5000s
Full-D
Time

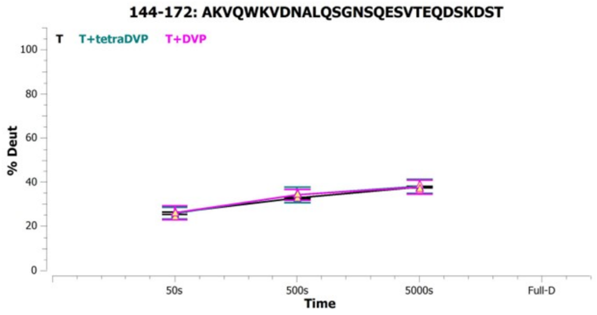
144-172: AKVQWKVDNALQSGNSQESVTEQDSKDST
T
T+tetraDVP
T+DVP
% Deut
0
20
40
60
80
100
50s
500s
5000s
Full-D
Time

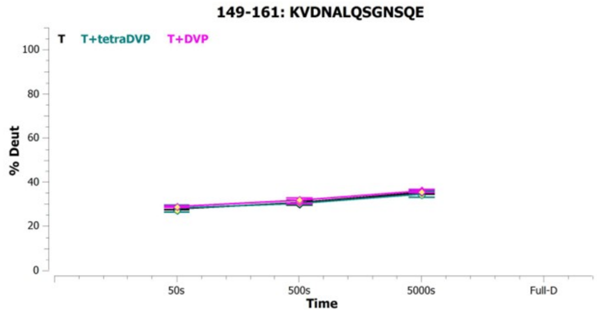
149-161: KVDNALQSGNSQE
T
T+tetraDVP
T+DVP
% Deut
0
20
40
60
80
100
50s
500s
5000s
Full-D
Time

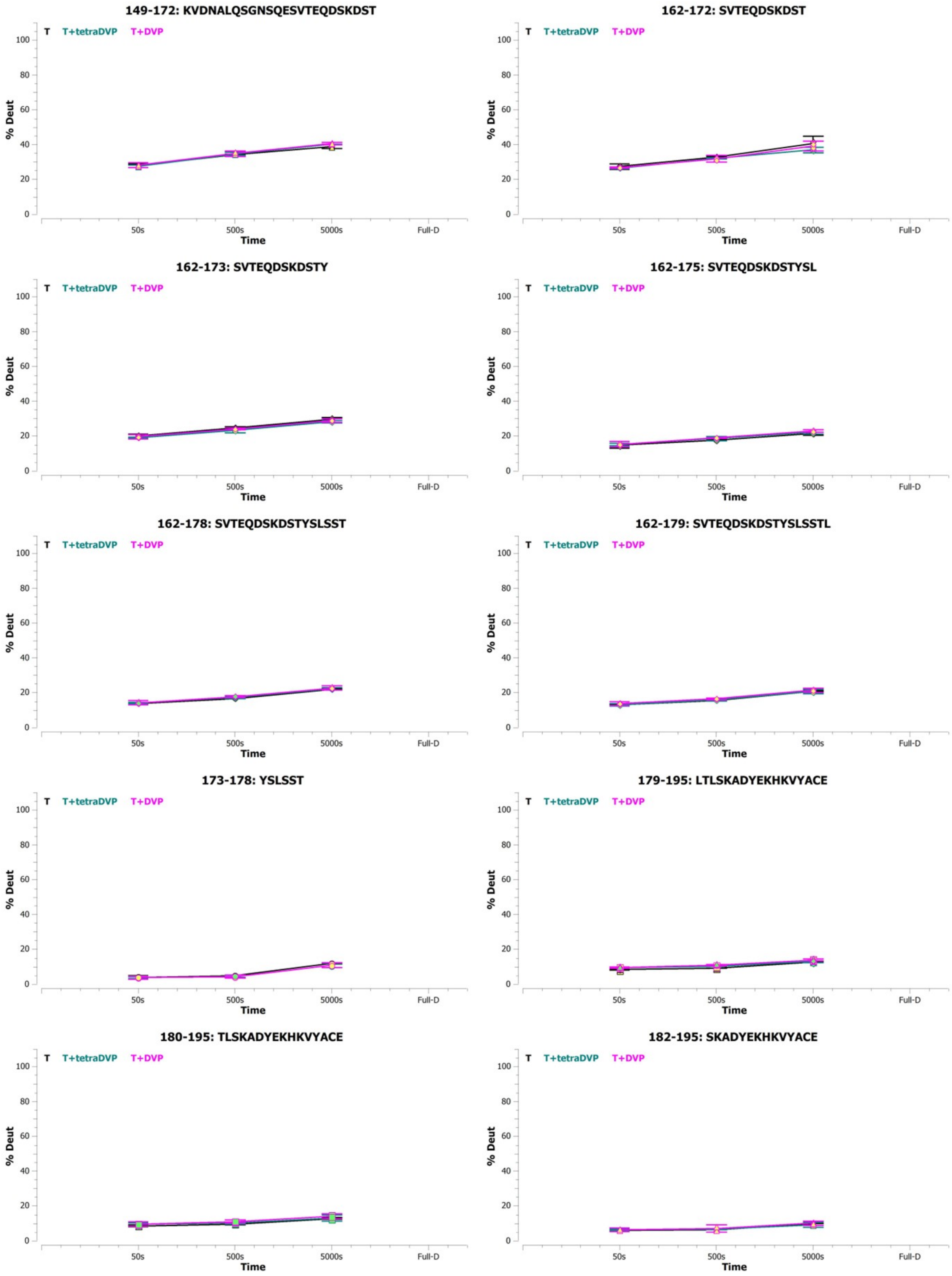
149-172: KVDNALQSGNSQESVTEQDSKDST
162-172: SVTEQDSKDST
162-173: SVTEQDSKDSTY
162-175: SVTEQDSKDSTYSL
162-178: SVTEQDSKDSTYSLSST
162-179: SVTEQDSKDSTYSLSSTL
173-178: YSLSST
179-195: LTLSKADYEKHKVYACE
180-195: TLSKADYEKHKVYACE
182-195: SKADYEKHKVYACE
T
T+tetraDVP
T+DVP
% Deut
0
20
40
60
80
100
Time
50s
500s
5000s
Full-D

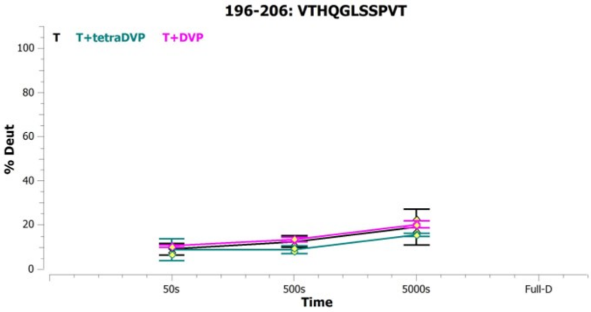
196-206: VTHQGLSSPVT
T
T+tetraDVP
T+DVP
% Deut
Time
50s
500s
5000s
Full-D

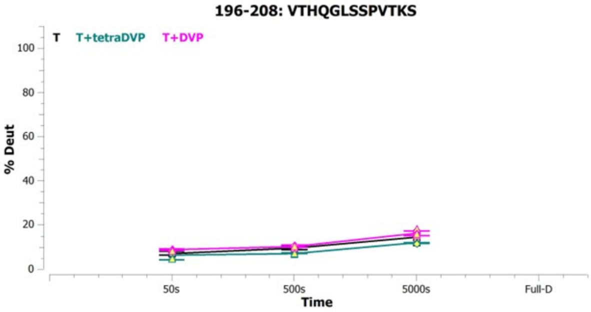
196-208: VTHQGLSSPVTKS
T
T+tetraDVP
T+DVP
% Deut
Time
50s
500s
5000s
Full-D

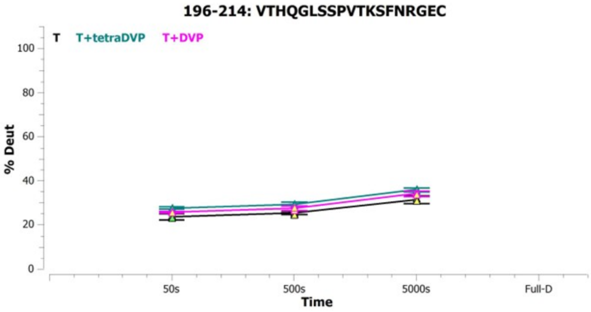
196-214: VTHQGLSSPVTKSFNRGEC
T
T+tetraDVP
T+DVP
% Deut
Time
50s
500s
5000s
Full-D

Heavy chain

T = trastuzumab, T+tetraDVP = TetraDVP conjugate **6**, T+DVP = DVP conjugate **4**

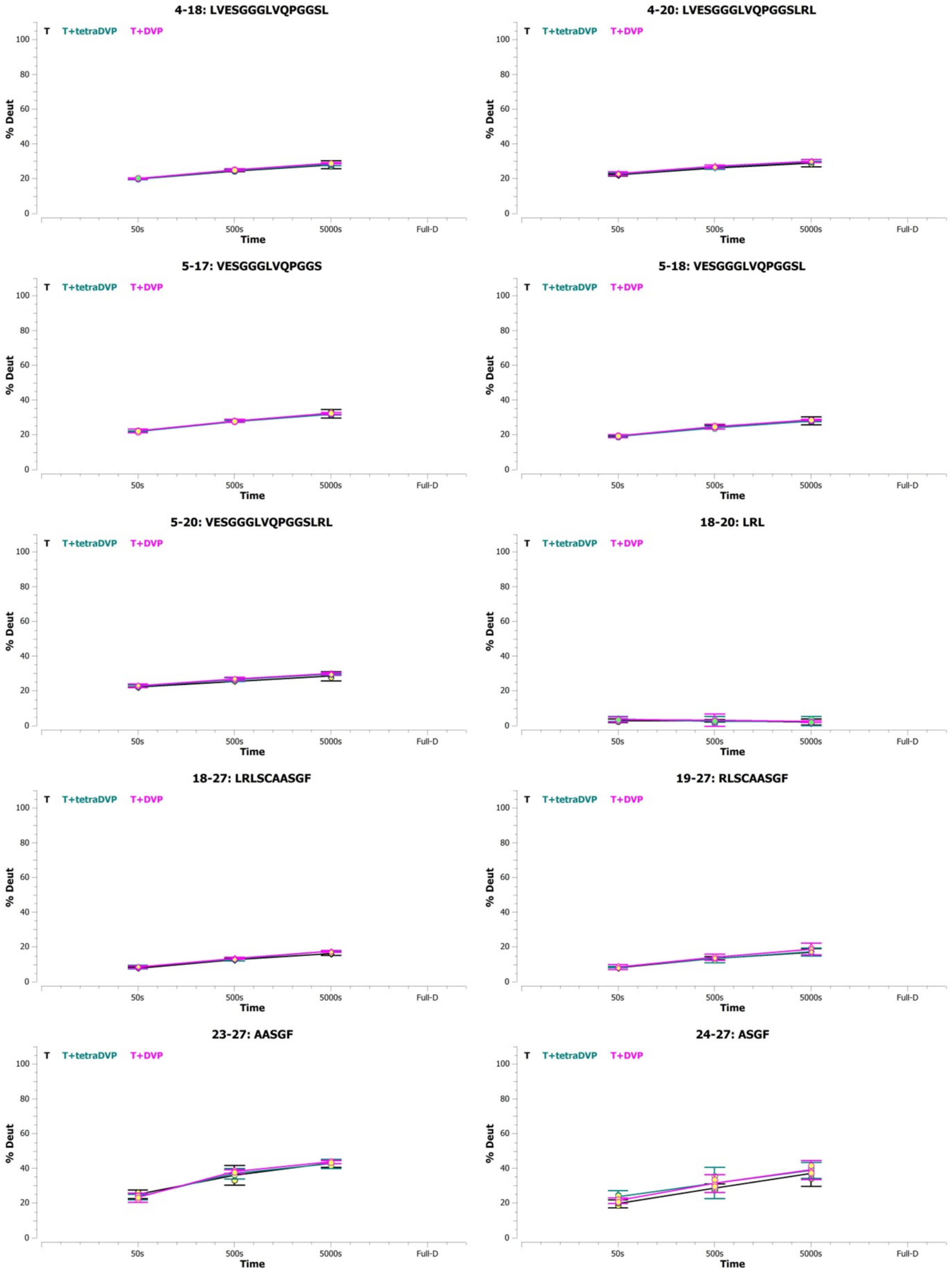

28-36: NIKDTYIHW

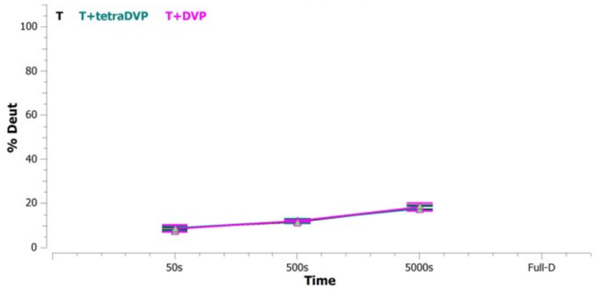

28-46: NIKDTYIHWVRQAPGKGLE

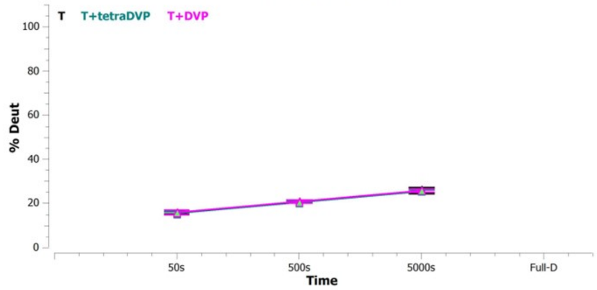

28-47: NIKDTYIHWVRQAPGKGLEW

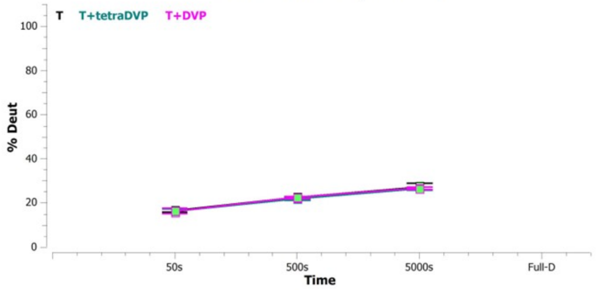

28-48: NIKDTYIHWVRQAPGKGLEWV

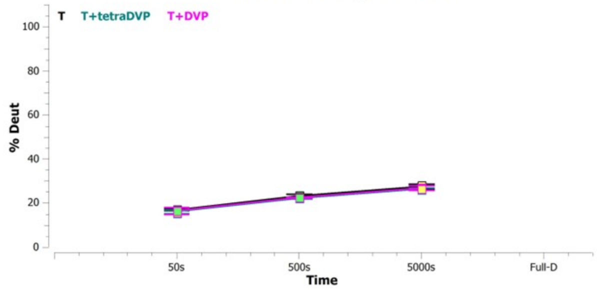

28-49: NIKDTYIHWVRQAPGKGLEWVA

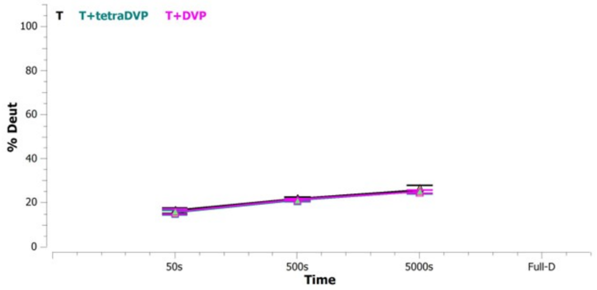

28-56: NIKDTYIHWVRQAPGKGLEWVARIYPTNG

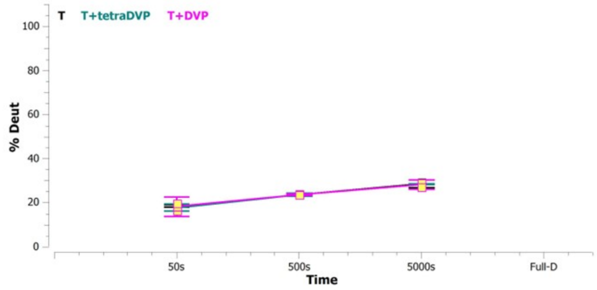

34-46: IHWVRQAPGKGLE

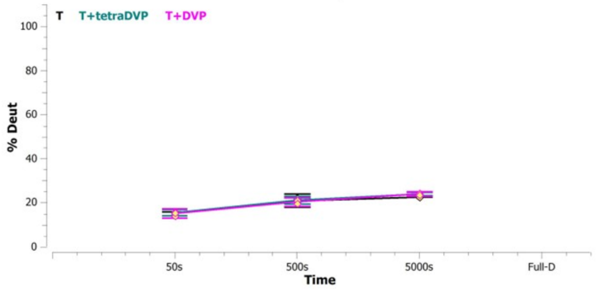

37-46: VRQAPGKGLE

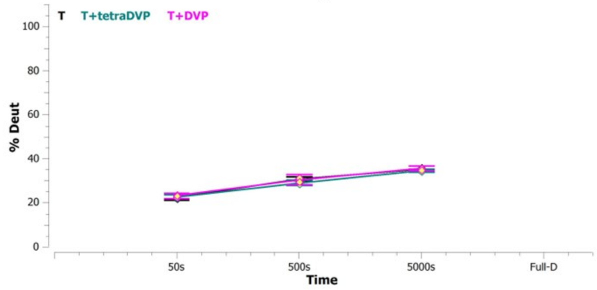

37-47: VRQAPGKGLEW

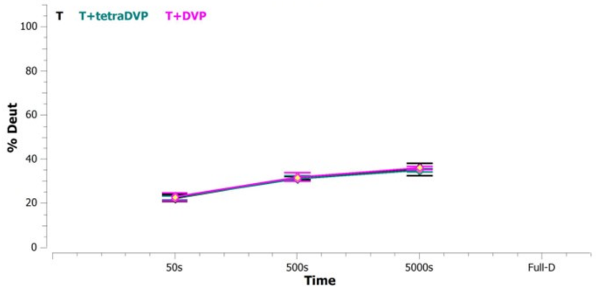

37-49: VRQAPGKGLEWVA

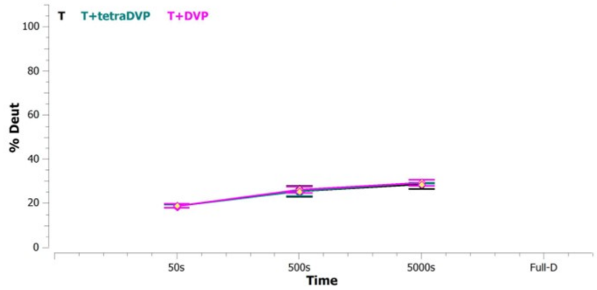

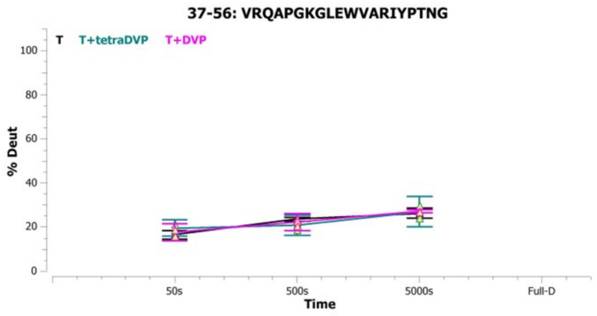
37-56: VRQAPGKGLEWVARIYPTNG
T
T+tetraDVP
T+DVP
% Deut
0
20
40
60
80
100
50s
500s
5000s
Full-D
Time

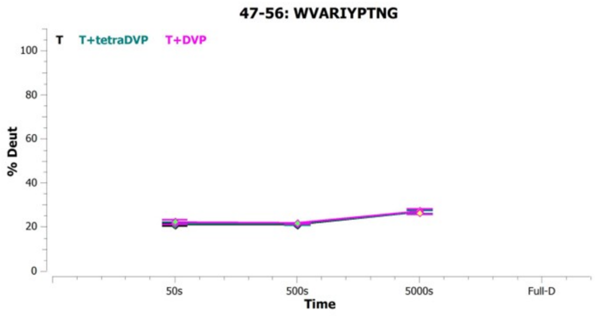
47-56: WVARIYPTNG
T
T+tetraDVP
T+DVP
% Deut
0
20
40
60
80
100
50s
500s
5000s
Full-D
Time

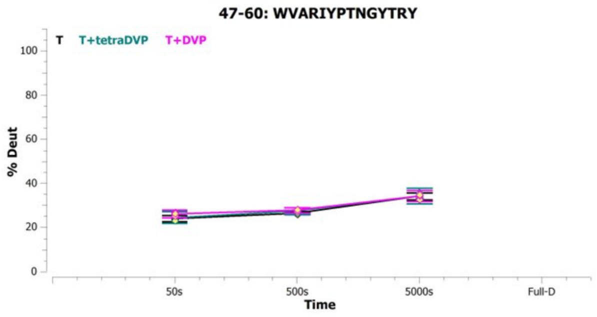
47-60: WVARIYPTNGYTRY
T
T+tetraDVP
T+DVP
% Deut
0
20
40
60
80
100
50s
500s
5000s
Full-D
Time

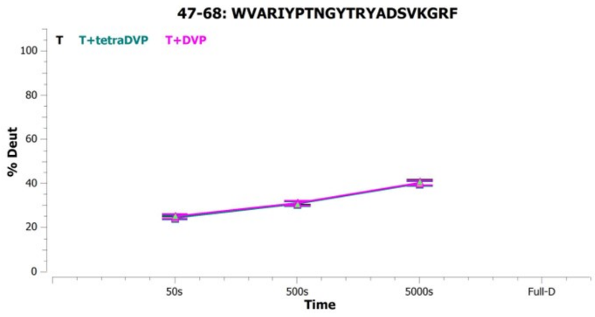
47-68: WVARIYPTNGYTRYADSVKGRF
T
T+tetraDVP
T+DVP
% Deut
0
20
40
60
80
100
50s
500s
5000s
Full-D
Time

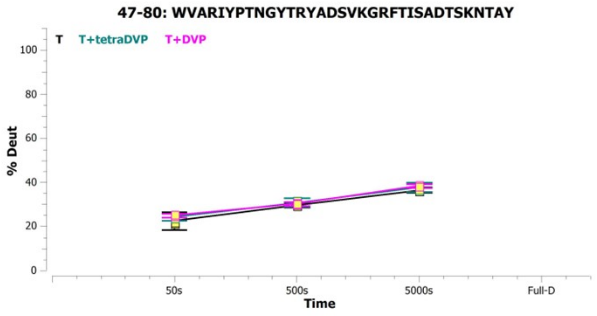
47-80: WVARIYPTNGYTRYADSVKGRFTISADTSKNTAY
T
T+tetraDVP
T+DVP
% Deut
0
20
40
60
80
100
50s
500s
5000s
Full-D
Time

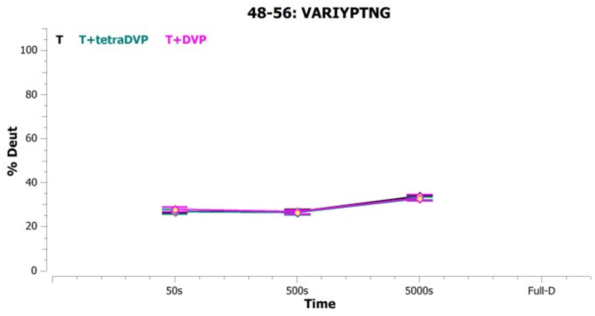
48-56: VARIYPTNG
T
T+tetraDVP
T+DVP
% Deut
0
20
40
60
80
100
50s
500s
5000s
Full-D
Time

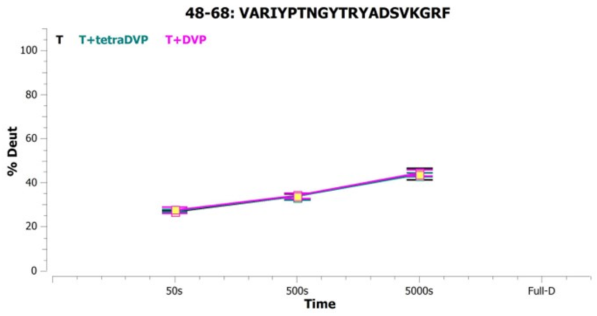
48-68: VARIYPTNGYTRYADSVKGRF
T
T+tetraDVP
T+DVP
% Deut
0
20
40
60
80
100
50s
500s
5000s
Full-D
Time

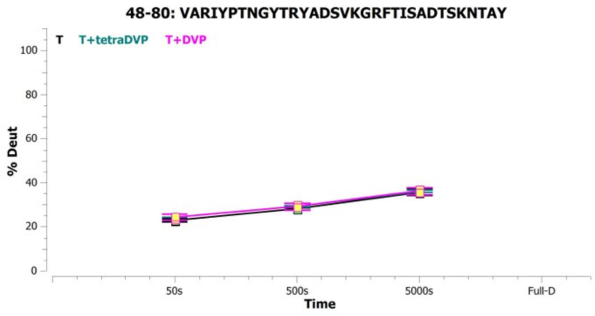
48-80: VARIYPTNGYTRYADSVKGRFTISADTSKNTAY
T
T+tetraDVP
T+DVP
% Deut
0
20
40
60
80
100
50s
500s
5000s
Full-D
Time

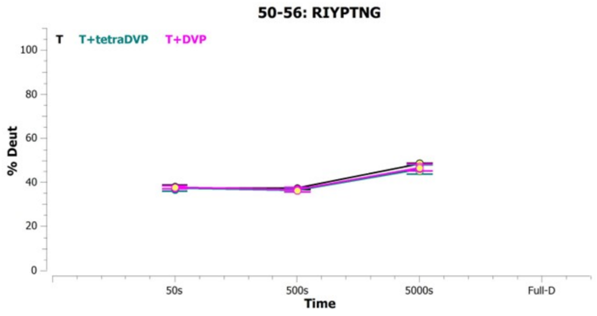
50-56: RIYPTNG
T
T+tetraDVP
T+DVP
% Deut
0
20
40
60
80
100
50s
500s
5000s
Full-D
Time

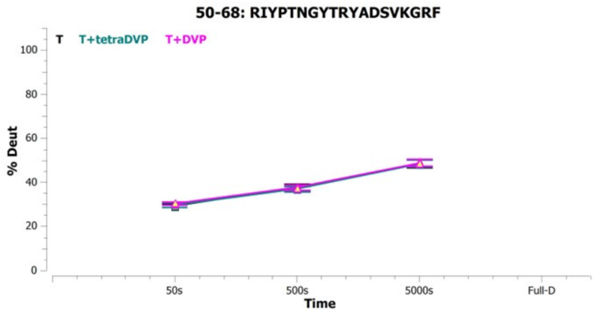
50-68: RIYPTNGYTRYADSVKGRF
T
T+tetraDVP
T+DVP
% Deut
0
20
40
60
80
100
50s
500s
5000s
Full-D
Time

57-68: YTRYADSVKGRF

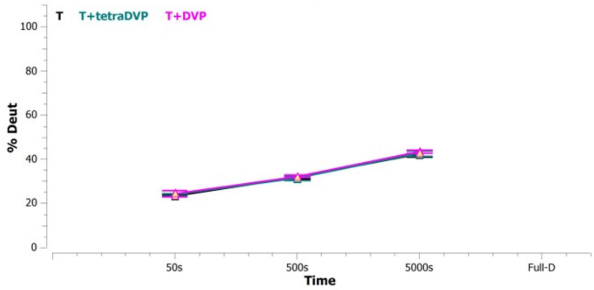

57-80: YTRYADSVKGRFTISADTSKNTAY

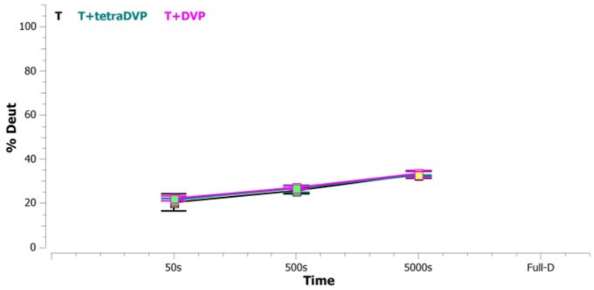

61-68: ADSVKGRF

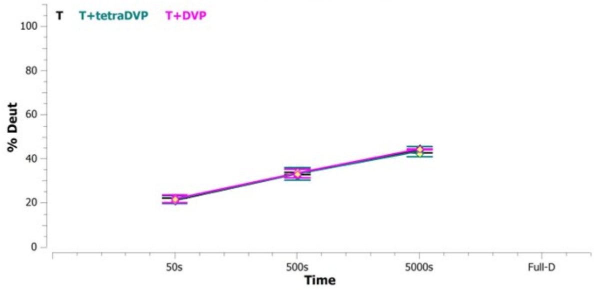

69-80: TISADTSKNTAY

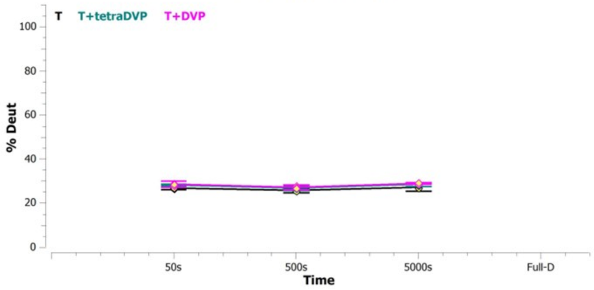

69-81: TISADTSKNTAYL

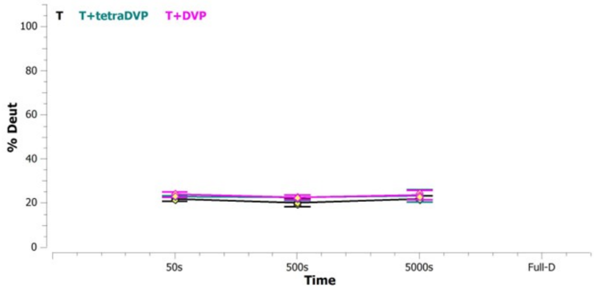

69-83: TISADTSKNTAYLQM

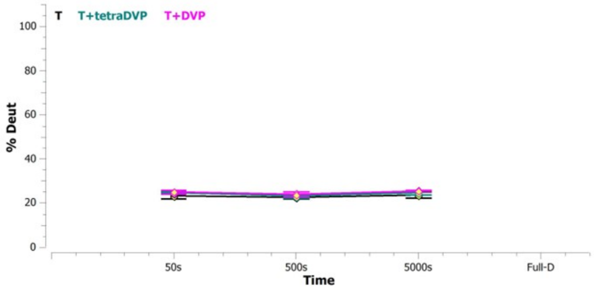

72-80: ADTSKNTAY

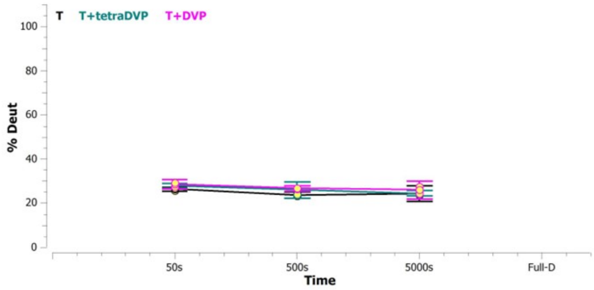

73-80: DTSKNTAY

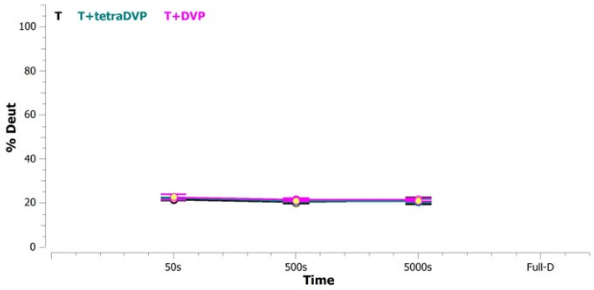

81-86: LQMNSL

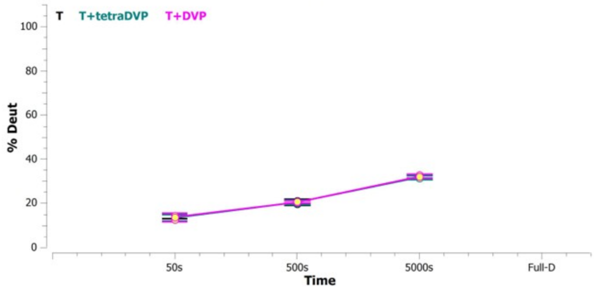

81-93: LQMNSLRAEDTAV

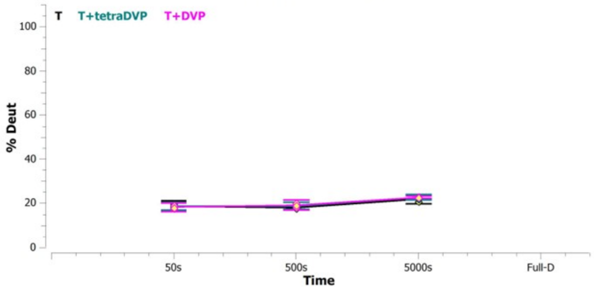

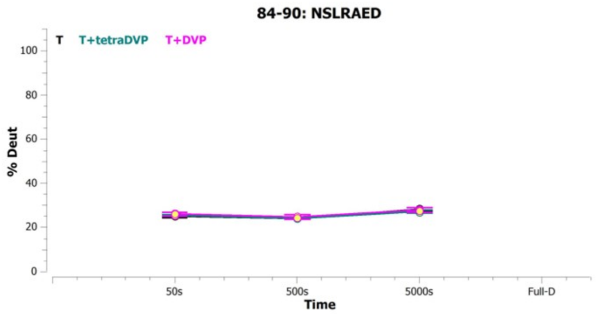
84-90: NSLRAED
T
T+tetraDVP
T+DVP
% Deut
0
20
40
60
80
100
50s
500s
5000s
Full-D
Time

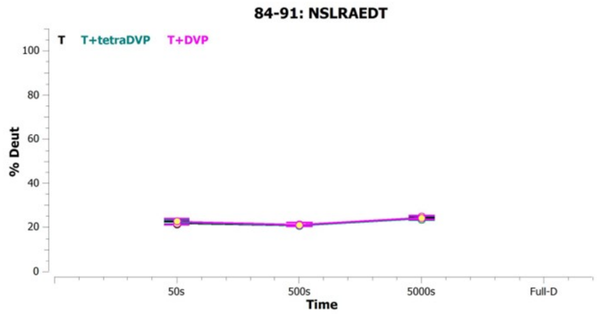
84-91: NSLRAEDT
T
T+tetraDVP
T+DVP
% Deut
0
20
40
60
80
100
50s
500s
5000s
Full-D
Time

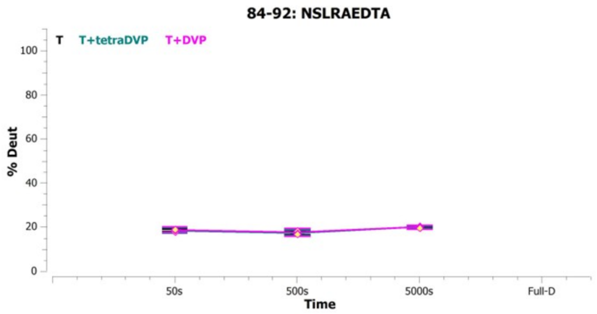
84-92: NSLRAEDTA
T
T+tetraDVP
T+DVP
% Deut
0
20
40
60
80
100
50s
500s
5000s
Full-D
Time

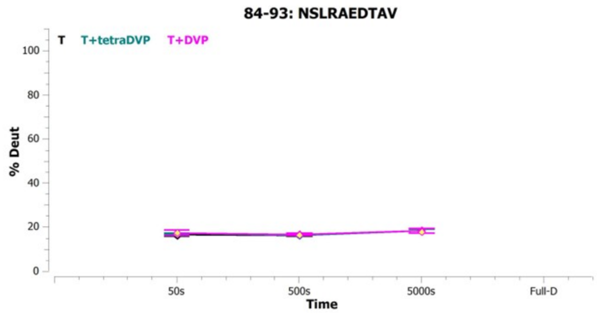
84-93: NSLRAEDTAV
T
T+tetraDVP
T+DVP
% Deut
0
20
40
60
80
100
50s
500s
5000s
Full-D
Time

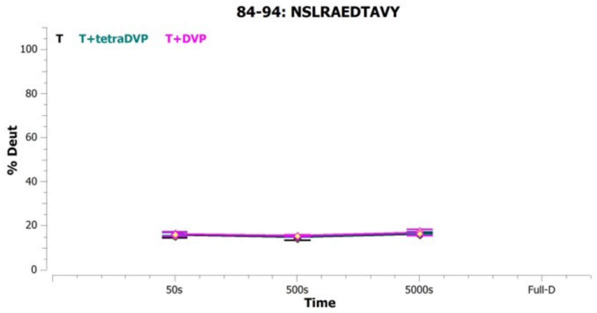
84-94: NSLRAEDTAVY
T
T+tetraDVP
T+DVP
% Deut
0
20
40
60
80
100
50s
500s
5000s
Full-D
Time

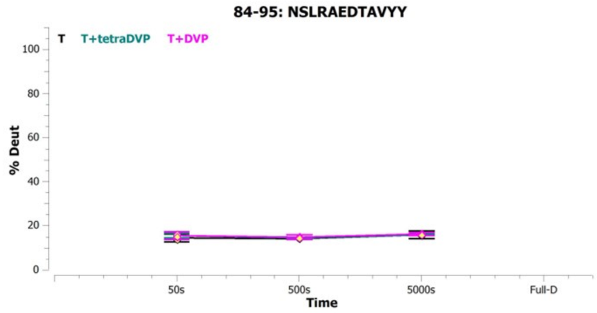
84-95: NSLRAEDTAVYY
T
T+tetraDVP
T+DVP
% Deut
0
20
40
60
80
100
50s
500s
5000s
Full-D
Time

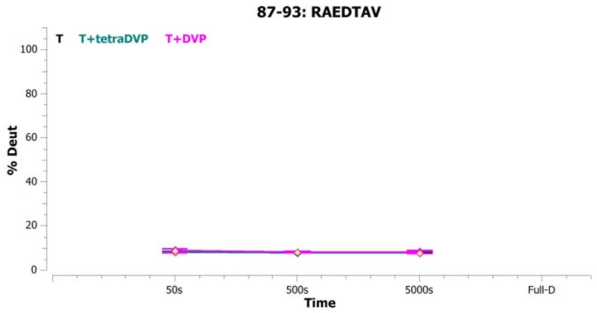
87-93: RAEDTAV
T
T+tetraDVP
T+DVP
% Deut
0
20
40
60
80
100
50s
500s
5000s
Full-D
Time

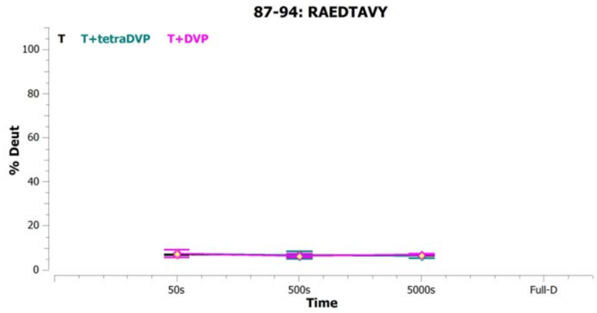
87-94: RAEDTAVY
T
T+tetraDVP
T+DVP
% Deut
0
20
40
60
80
100
50s
500s
5000s
Full-D
Time

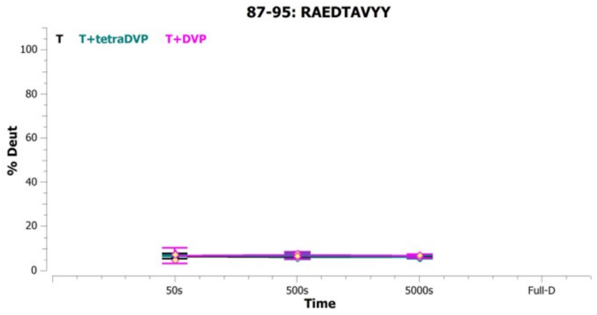
87-95: RAEDTAVYY
T
T+tetraDVP
T+DVP
% Deut
0
20
40
60
80
100
50s
500s
5000s
Full-D
Time

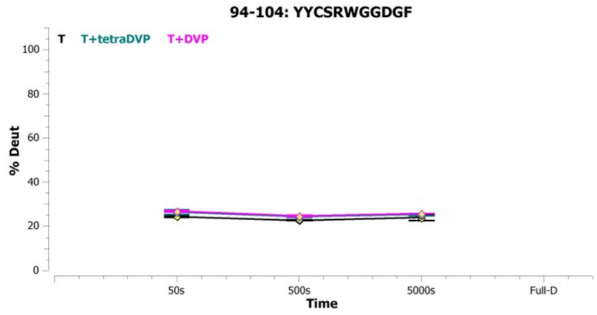
94-104: YYCSRWGGDGF
T
T+tetraDVP
T+DVP
% Deut
0
20
40
60
80
100
50s
500s
5000s
Full-D
Time

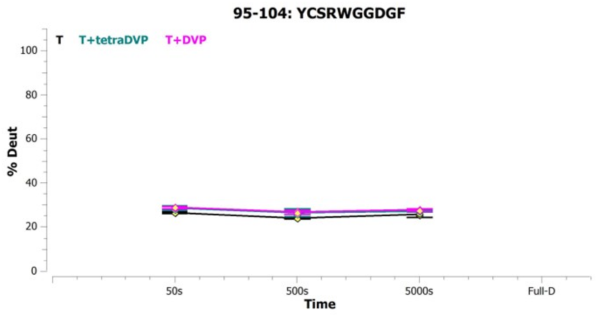
95-104: YCSRWGGDGF
T
T+tetraDVP
T+DVP
% Deut
Time
50s
500s
5000s
Full-D

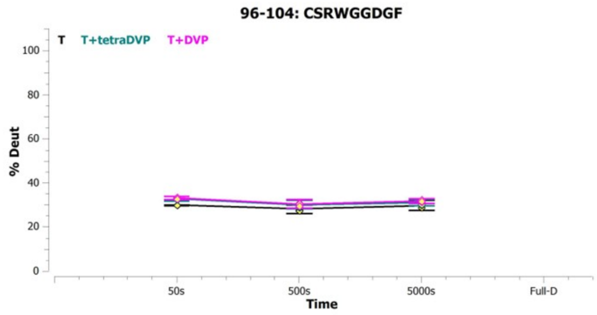
96-104: CSRWGGDGF
T
T+tetraDVP
T+DVP
% Deut
Time
50s
500s
5000s
Full-D

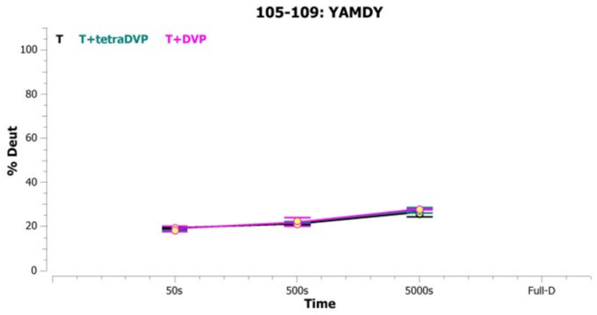
105-109: YAMDY
T
T+tetraDVP
T+DVP
% Deut
Time
50s
500s
5000s
Full-D

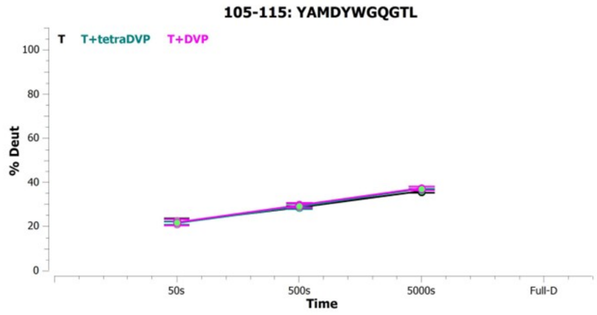
105-115: YAMDYWGQGTL
T
T+tetraDVP
T+DVP
% Deut
Time
50s
500s
5000s
Full-D

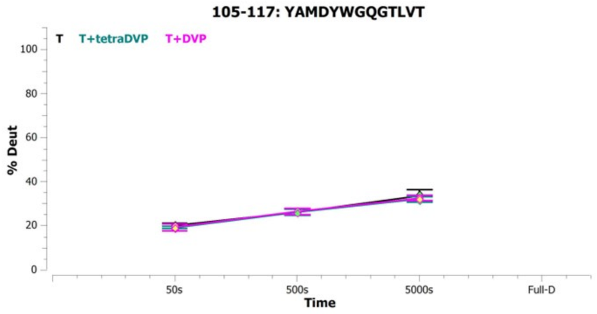
105-117: YAMDYWGQGTLVT
T
T+tetraDVP
T+DVP
% Deut
Time
50s
500s
5000s
Full-D

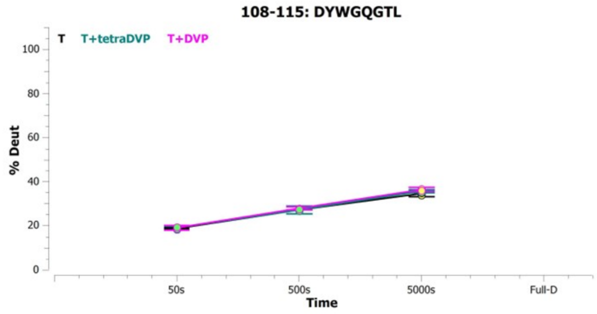
108-115: DYWGQGTL
T
T+tetraDVP
T+DVP
% Deut
Time
50s
500s
5000s
Full-D

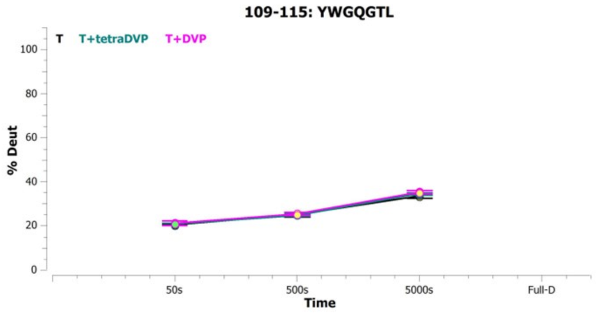
109-115: YWGQGTL
T
T+tetraDVP
T+DVP
% Deut
Time
50s
500s
5000s
Full-D

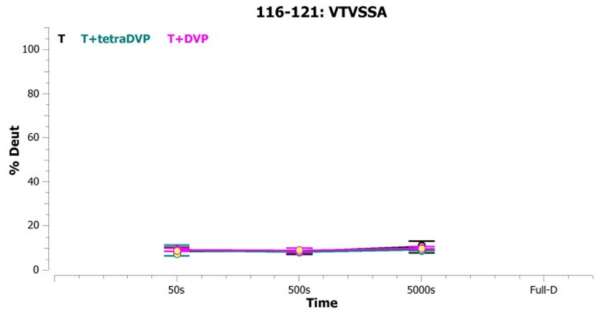
116-121: VTVSSA
T
T+tetraDVP
T+DVP
% Deut
Time
50s
500s
5000s
Full-D

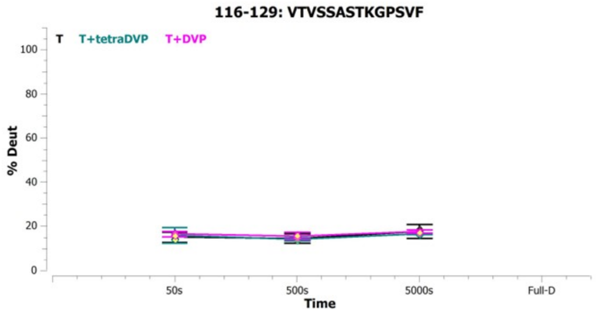
116-129: VTVSSASTKGPSVF
T
T+tetraDVP
T+DVP
% Deut
Time
50s
500s
5000s
Full-D

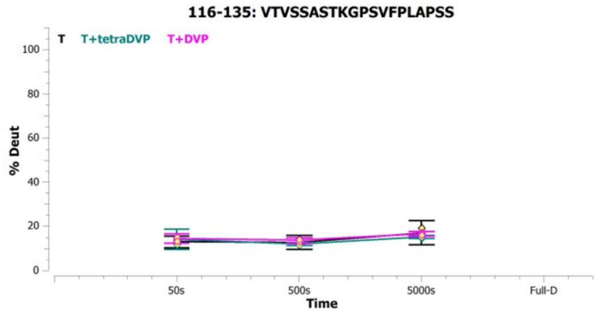
116-135: VTVSSASTKGPSVFPLAPSS
T
T+tetraDVP
T+DVP
% Deut
Time
50s
500s
5000s
Full-D

116-144: VTVSSASTKGPSVFPLAPSSKSTSGGTAA

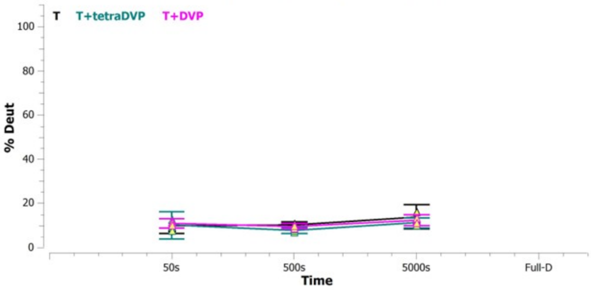

116-145: VTVSSASTKGPSVFPLAPSSKSTSGGTAAL

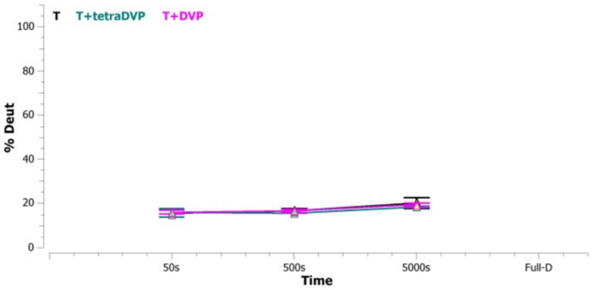

116-147: VTVSSASTKGPSVFPLAPSSKSTSGGTAALGC

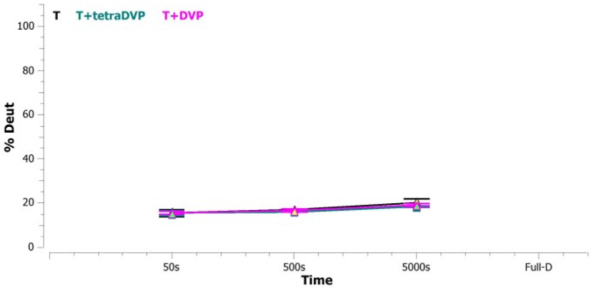

130-145: PLAPSSKSTSGGTAAL

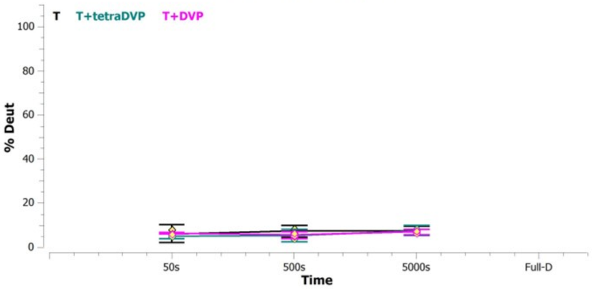

148-158: LVKDYFPEPVT

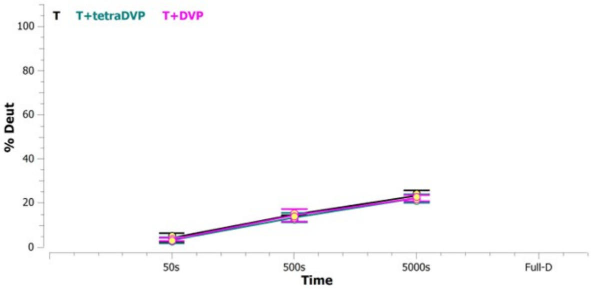

149-158: VKDYFPEPVT

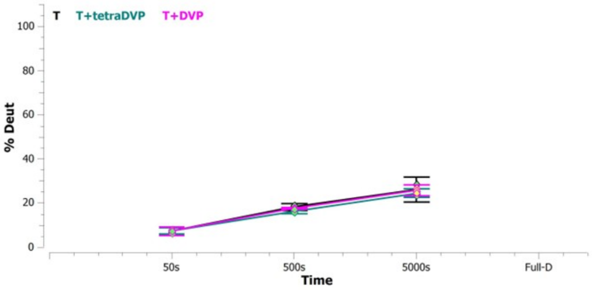

159-161: VSW

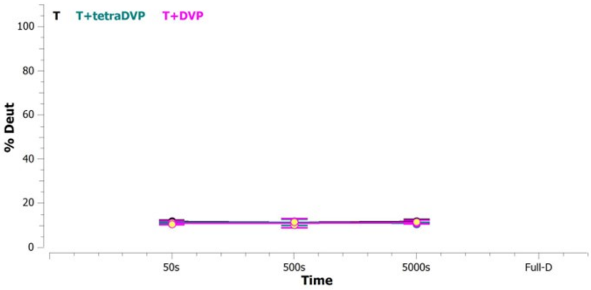

159-177: VSWNSGALTSGVHTFPAVL

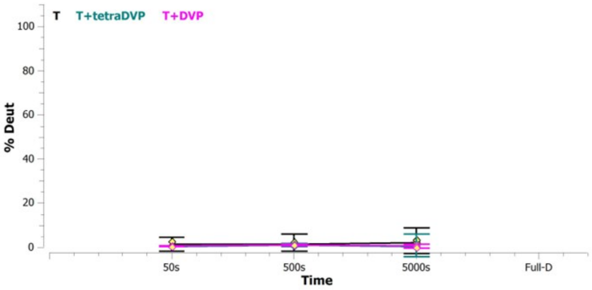

162-177: NSGALTSGVHTFPAVL

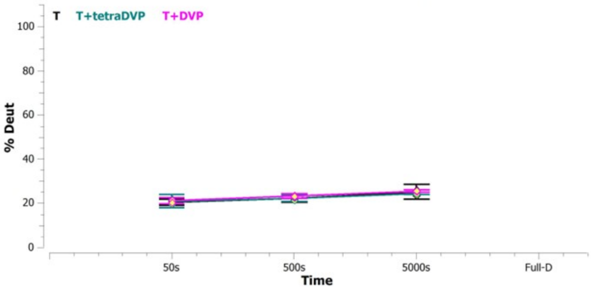

167-177: TSGVHTFPAVL

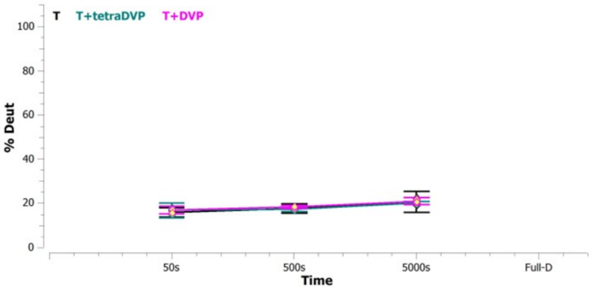

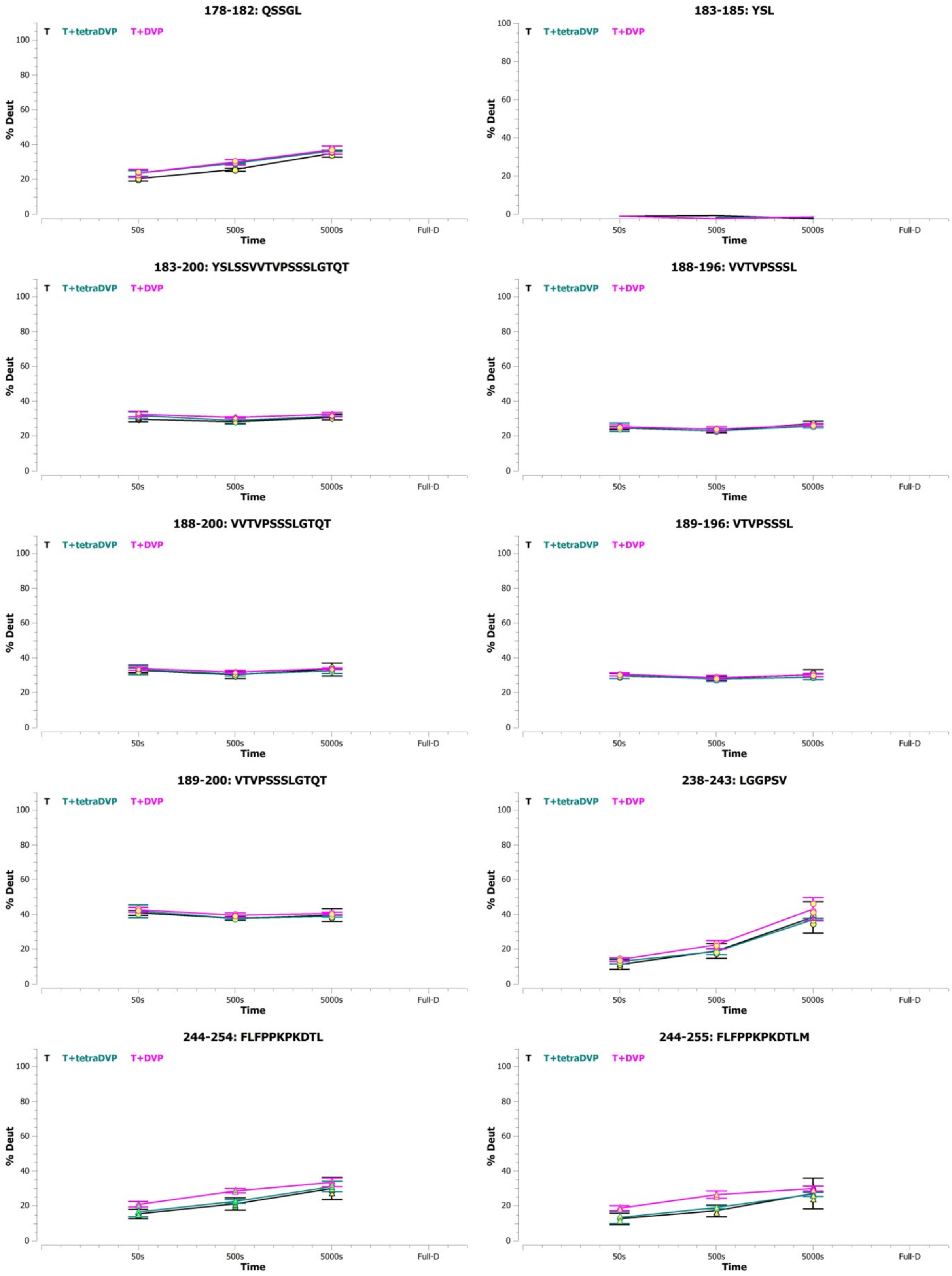
178-182: QSSGL
183-185: YSL
183-200: YSLSSVVTVPSSSLGTQT
188-196: VVTVPSSSL
188-200: VVTVPSSSLGTQT
189-196: VTVPSSSL
189-200: VTVPSSSLGTQT
238-243: LGGPSV
244-254: FLFPPKPKDTL
244-255: FLFPPKPKDTLM
T
T+tetraDVP
T+DVP
% Deut
Time
50s
500s
5000s
Full-D

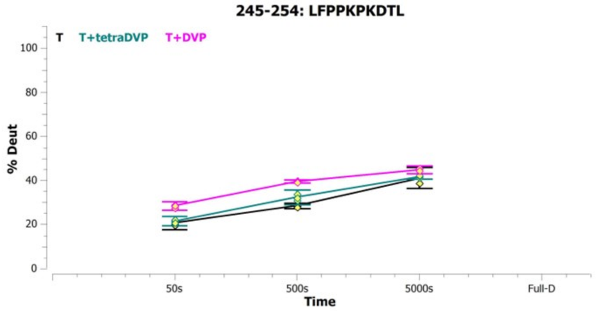
245-254: LFPPKPKDTL
T T+tetraDVP T+DVP
% Deut
0 20 40 60 80 100
50s 500s 5000s Full-D
Time

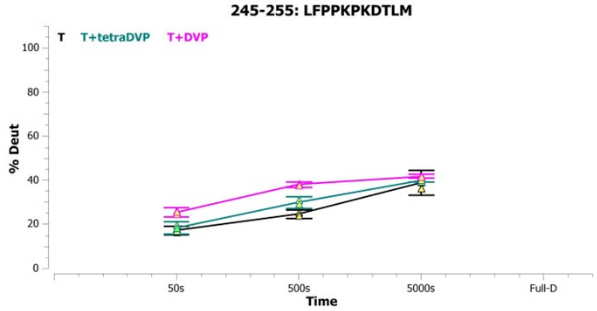
245-255: LFPPKPKDTLM
T T+tetraDVP T+DVP
% Deut
0 20 40 60 80 100
50s 500s 5000s Full-D
Time

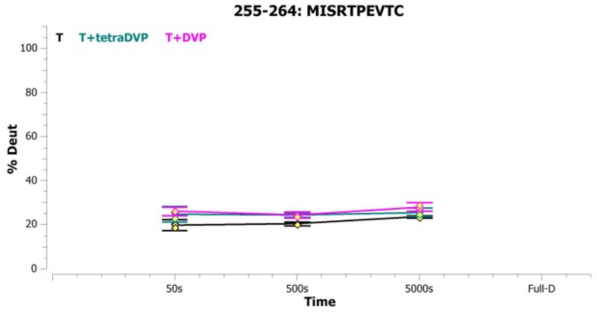
255-264: MISRTPEVTC
T T+tetraDVP T+DVP
% Deut
0 20 40 60 80 100
50s 500s 5000s Full-D
Time

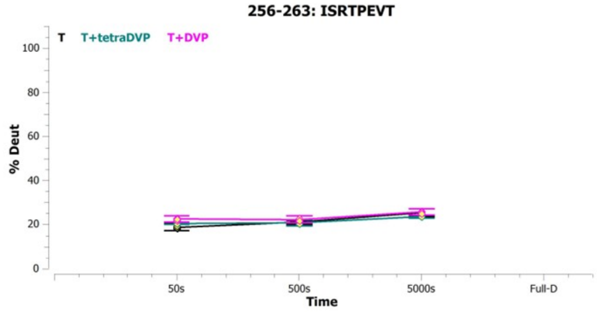
256-263: ISRTPEVT
T T+tetraDVP T+DVP
% Deut
0 20 40 60 80 100
50s 500s 5000s Full-D
Time

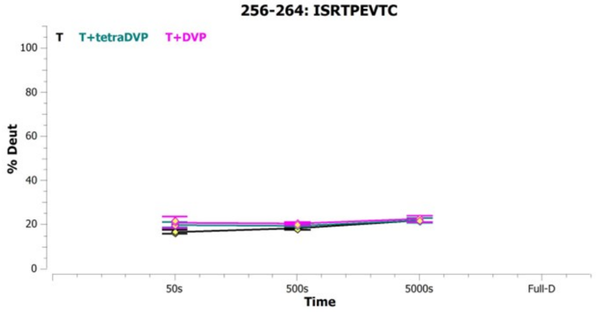
256-264: ISRTPEVTC
T T+tetraDVP T+DVP
% Deut
0 20 40 60 80 100
50s 500s 5000s Full-D
Time

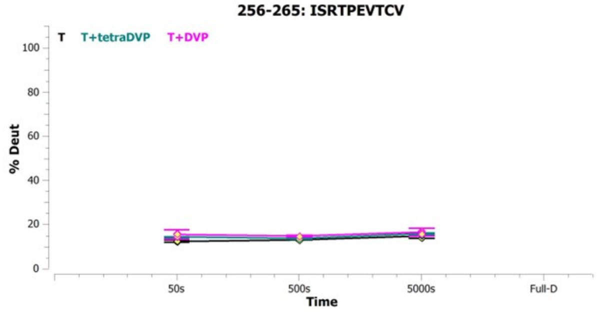
256-265: ISRTPEVTCV
T T+tetraDVP T+DVP
% Deut
0 20 40 60 80 100
50s 500s 5000s Full-D
Time

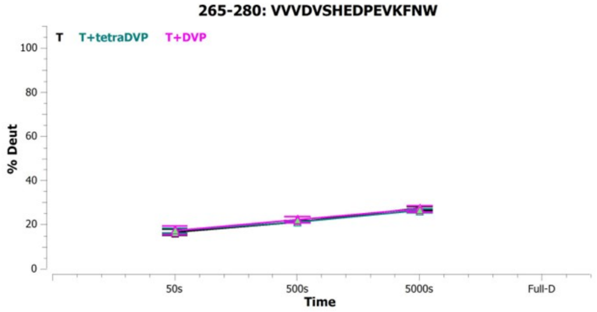
265-280: VVVDVSHEDPEVKFNW
T T+tetraDVP T+DVP
% Deut
0 20 40 60 80 100
50s 500s 5000s Full-D
Time

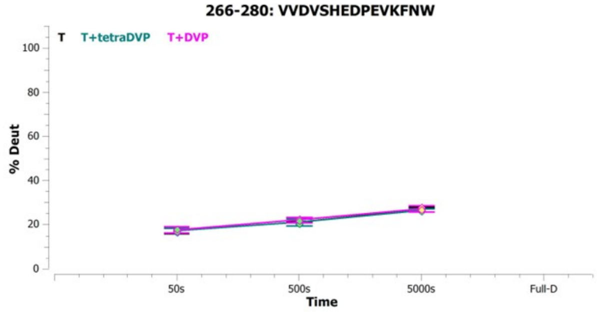
266-280: VVDVSHEDPEVKFNW
T T+tetraDVP T+DVP
% Deut
0 20 40 60 80 100
50s 500s 5000s Full-D
Time

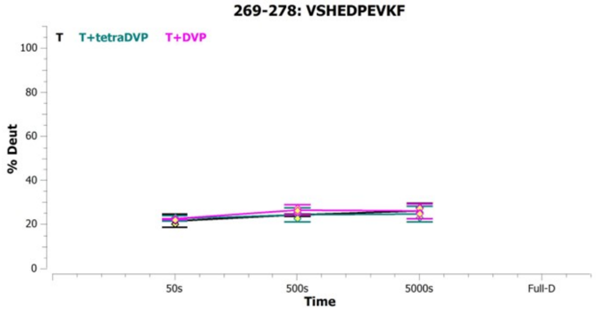
269-278: VSHEDPEVKF
T T+tetraDVP T+DVP
% Deut
0 20 40 60 80 100
50s 500s 5000s Full-D
Time

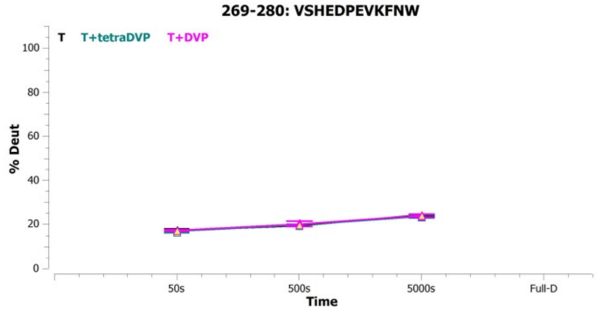
269-280: VSHEDPEVKFNW
T T+tetraDVP T+DVP
% Deut
0 20 40 60 80 100
50s 500s 5000s Full-D
Time

281-309: YVDGVEVHNAKTKPREEQYNSTYRVVSVL

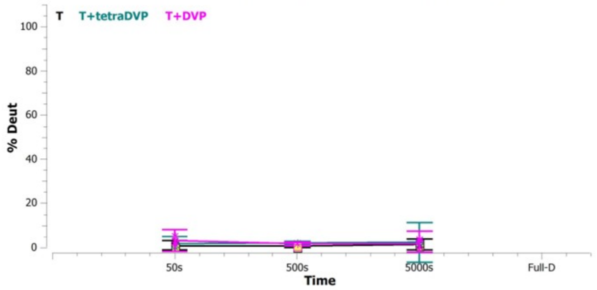

304-308: RVVSV

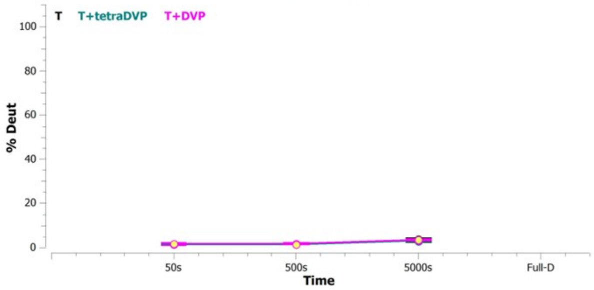

304-309: RVVSVL

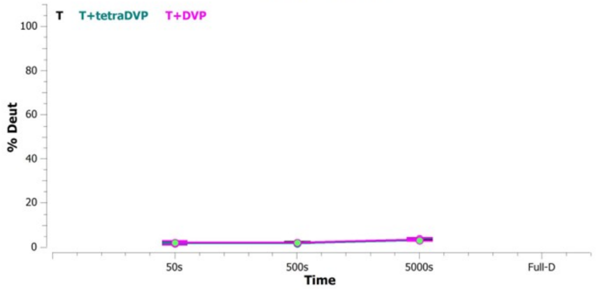

309-312: LTVL

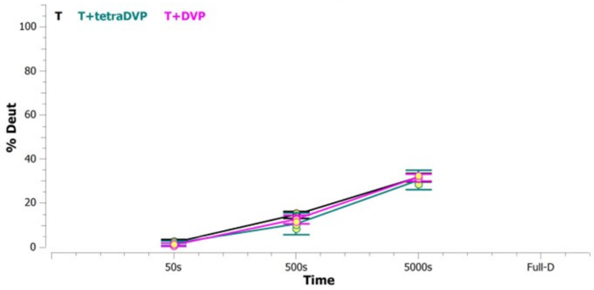

310-317: TVLHQDWL

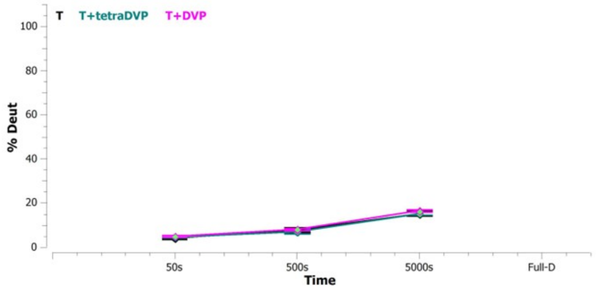

310-321: TVLHQDWLNGKE

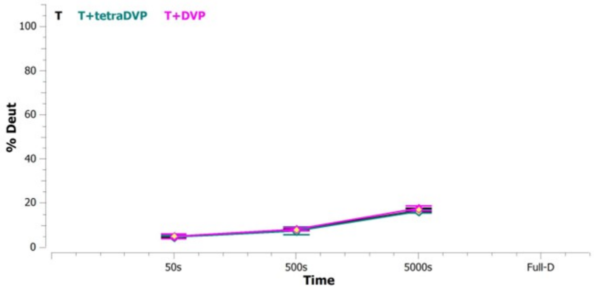

310-351: TVLHQDWLNGKEYKCKVSNKALPAPIEKTISKAKGQPREPQV

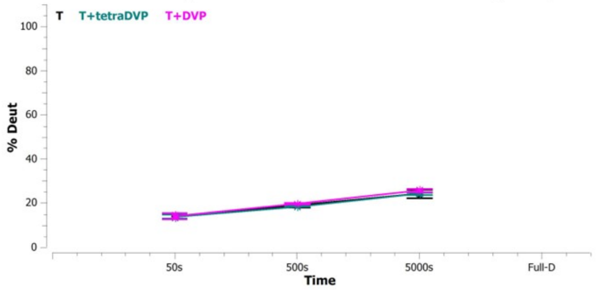

313-321: HQDWLNGKE

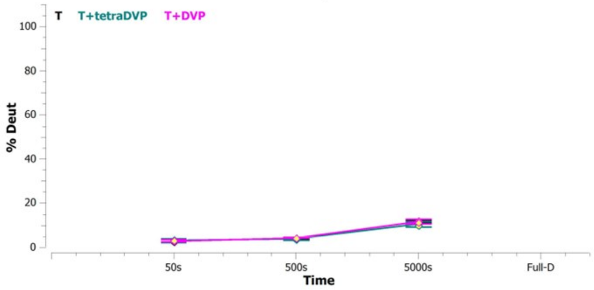

337-351: KTISKAKGQPREPQV

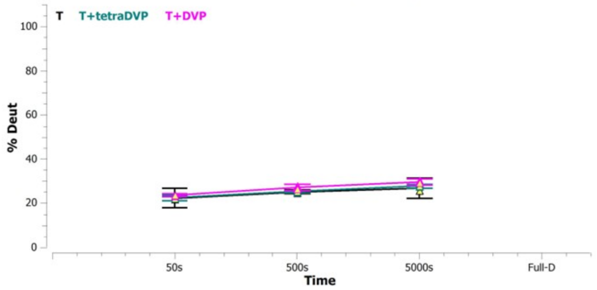

337-359: KTISKAKGQPREPQVYTLPPSRE

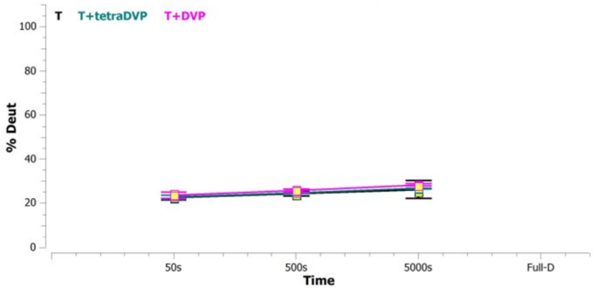

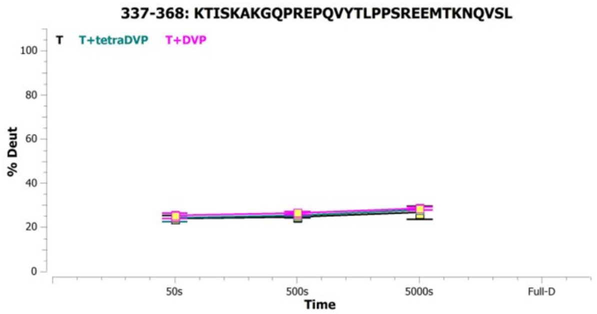
337-368: KTISKAKGQPREPQVYTLPPSREEMTKNQVSL
T
T+tetraDVP
T+DVP
% Deut
0 20 40 60 80 100
50s 500s 5000s Full-D
Time

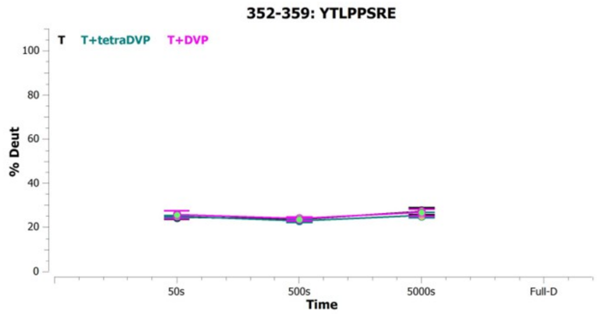
352-359: YTLPPSRE
T
T+tetraDVP
T+DVP
% Deut
0 20 40 60 80 100
50s 500s 5000s Full-D
Time

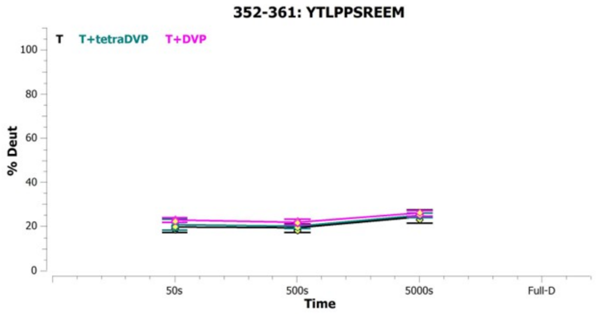
352-361: YTLPPSREEM
T
T+tetraDVP
T+DVP
% Deut
0 20 40 60 80 100
50s 500s 5000s Full-D
Time

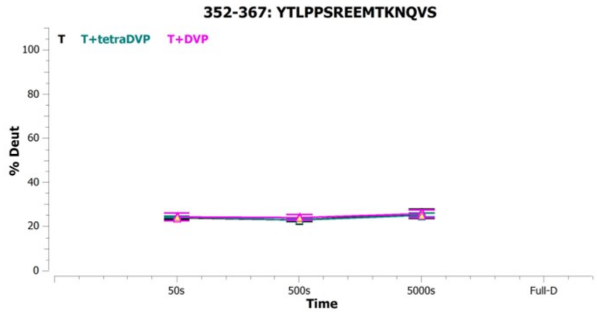
352-367: YTLPPSREEMTKNQVS
T
T+tetraDVP
T+DVP
% Deut
0 20 40 60 80 100
50s 500s 5000s Full-D
Time

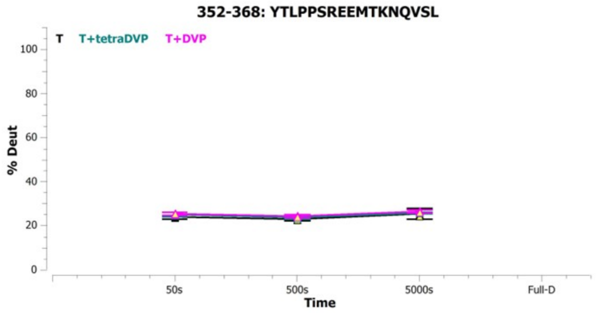
352-368: YTLPPSREEMTKNQVSL
T
T+tetraDVP
T+DVP
% Deut
0 20 40 60 80 100
50s 500s 5000s Full-D
Time

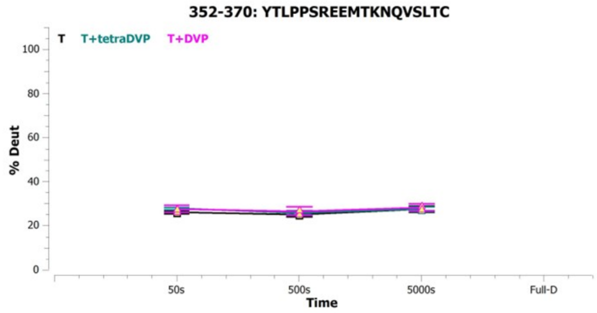
352-370: YTLPPSREEMTKNQVSLTC
T
T+tetraDVP
T+DVP
% Deut
0 20 40 60 80 100
50s 500s 5000s Full-D
Time

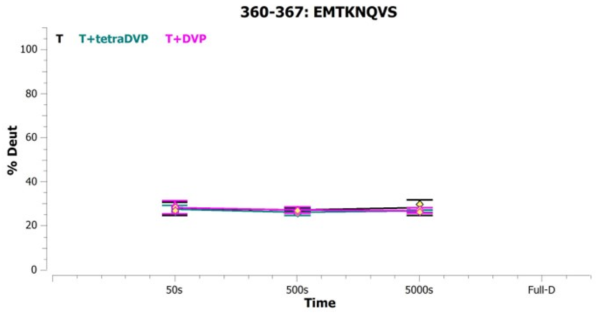
360-367: EMTKNQVS
T
T+tetraDVP
T+DVP
% Deut
0 20 40 60 80 100
50s 500s 5000s Full-D
Time

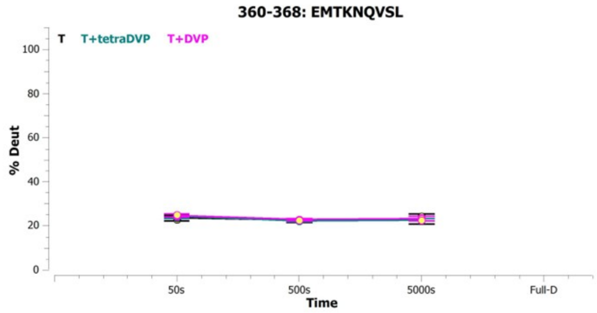
360-368: EMTKNQVSL
T
T+tetraDVP
T+DVP
% Deut
0 20 40 60 80 100
50s 500s 5000s Full-D
Time

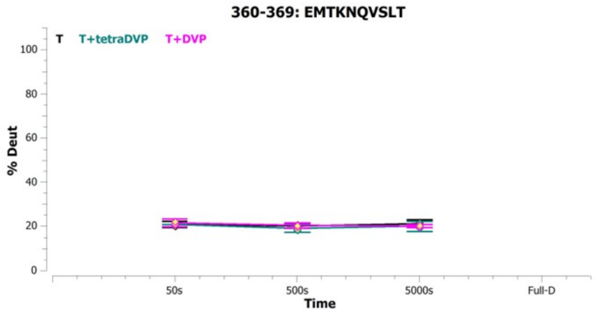
360-369: EMTKNQVSLT
T
T+tetraDVP
T+DVP
% Deut
0 20 40 60 80 100
50s 500s 5000s Full-D
Time

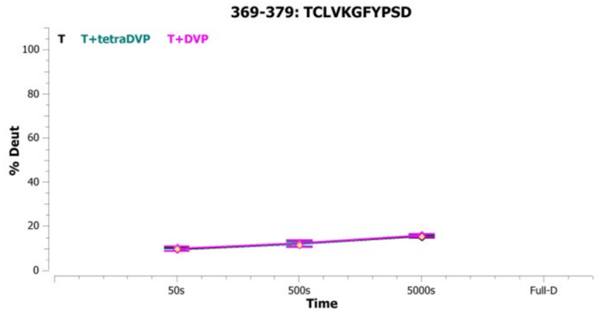
369-379: TCLVKGFYPSD
T
T+tetraDVP
T+DVP
% Deut
0 20 40 60 80 100
50s 500s 5000s Full-D
Time

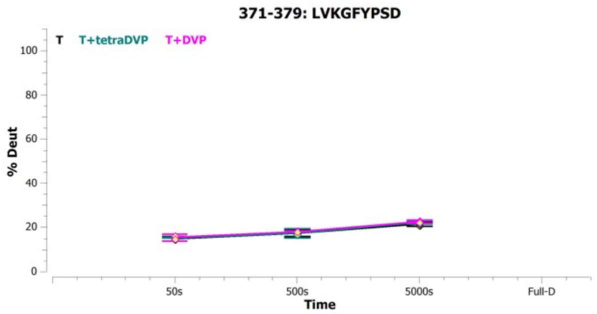
371-379: LVKGFYPSD
T
T+tetraDVP
T+DVP
% Deut
0
20
40
60
80
100
50s
500s
5000s
Full-D
Time

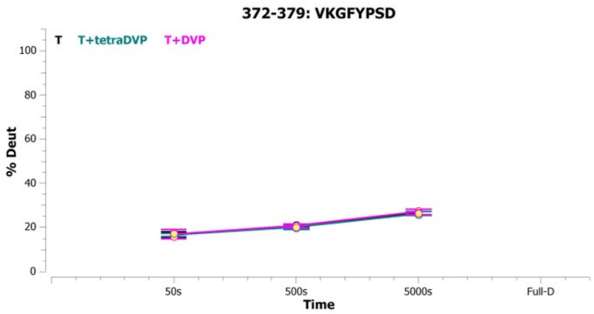
372-379: VKGFYPSD
T
T+tetraDVP
T+DVP
% Deut
0
20
40
60
80
100
50s
500s
5000s
Full-D
Time

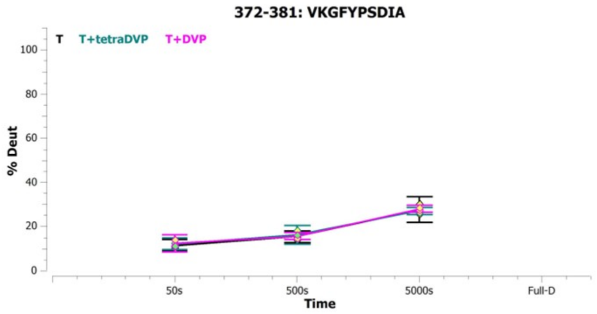
372-381: VKGFYPSDIA
T
T+tetraDVP
T+DVP
% Deut
0
20
40
60
80
100
50s
500s
5000s
Full-D
Time

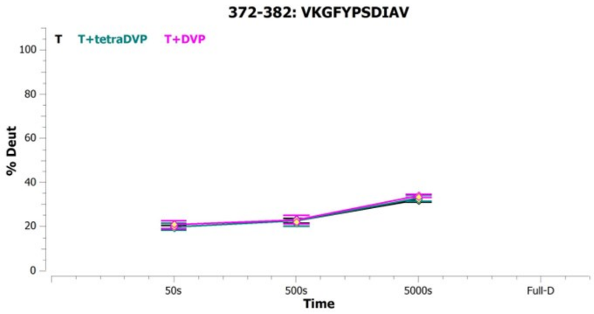
372-382: VKGFYPSDIAV
T
T+tetraDVP
T+DVP
% Deut
0
20
40
60
80
100
50s
500s
5000s
Full-D
Time

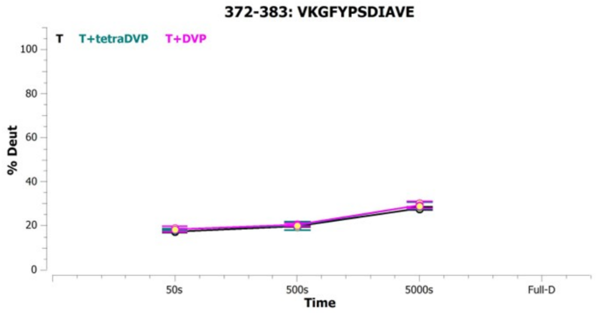
372-383: VKGFYPSDIAVE
T
T+tetraDVP
T+DVP
% Deut
0
20
40
60
80
100
50s
500s
5000s
Full-D
Time

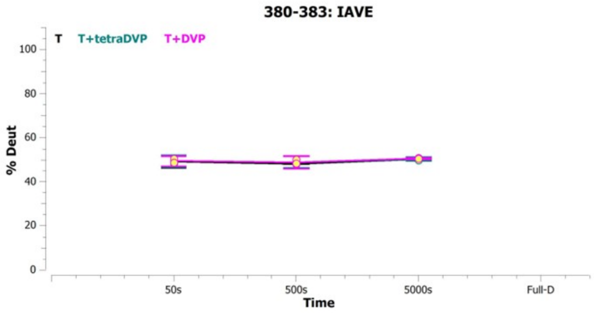
380-383: IAVE
T
T+tetraDVP
T+DVP
% Deut
0
20
40
60
80
100
50s
500s
5000s
Full-D
Time

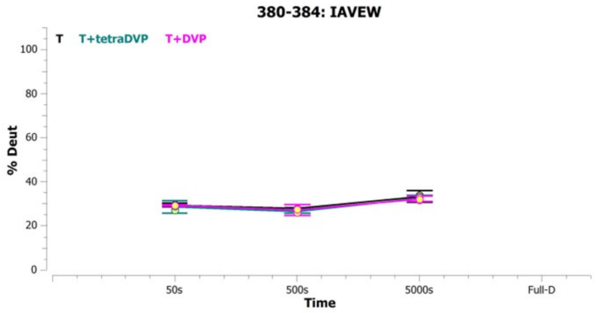
380-384: IAVEW
T
T+tetraDVP
T+DVP
% Deut
0
20
40
60
80
100
50s
500s
5000s
Full-D
Time

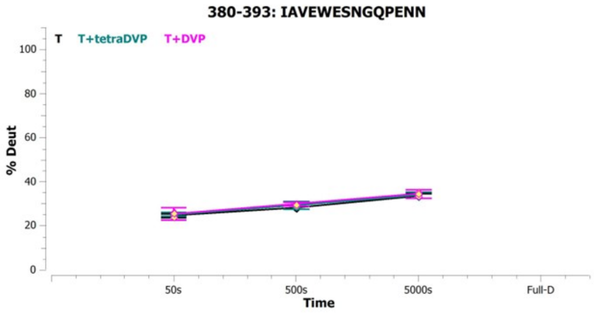
380-393: IAVEWESNGQPENN
T
T+tetraDVP
T+DVP
% Deut
0
20
40
60
80
100
50s
500s
5000s
Full-D
Time

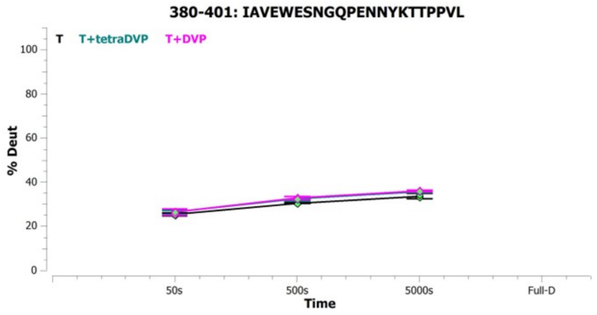
380-401: IAVEWESNGQPENNYKTTPPVL
T
T+tetraDVP
T+DVP
% Deut
0
20
40
60
80
100
50s
500s
5000s
Full-D
Time

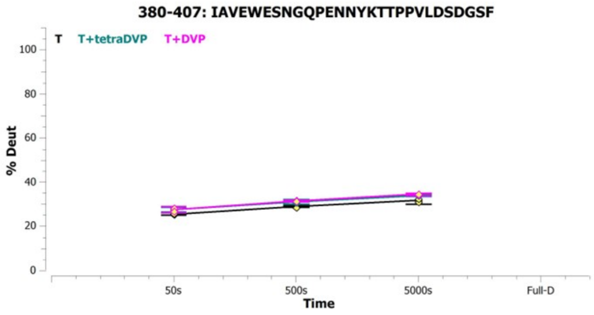
380-407: IAVEWESNGQPENNYKTTPPVLDSDGSF
T
T+tetraDVP
T+DVP
% Deut
0
20
40
60
80
100
50s
500s
5000s
Full-D
Time

382-401: VEWESNGQPENNYKTTPPVL

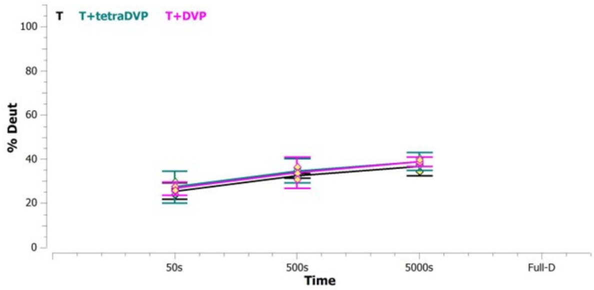

383-401: EWESNGQPENNYKTTPPVL

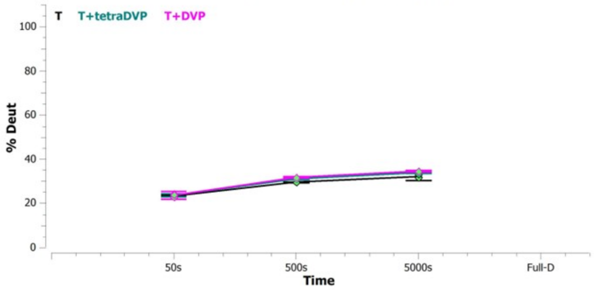

384-393: WESNGQPENN

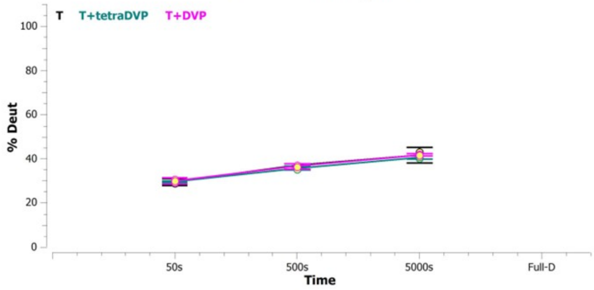

384-401: WESNGQPENNYKTTPPVL

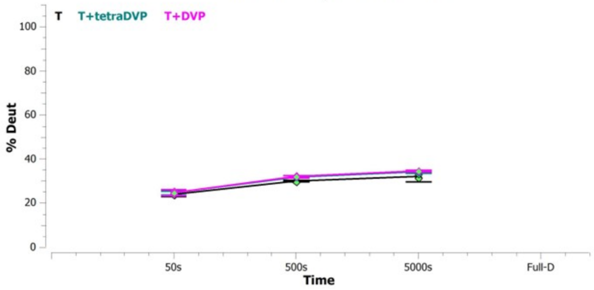

384-407: WESNGQPENNYKTTPPVLDSDGSF

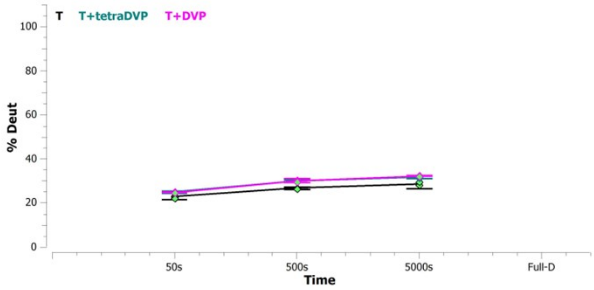

385-401: ESNGQPENNYKTTPPVL

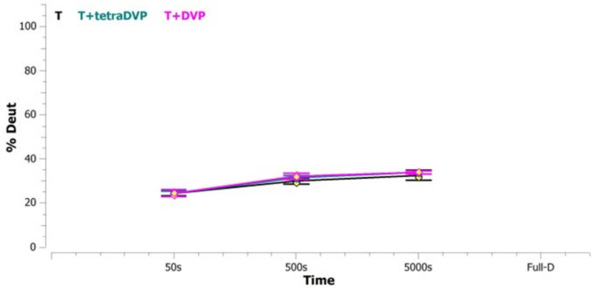

394-401: YKTTPPVL

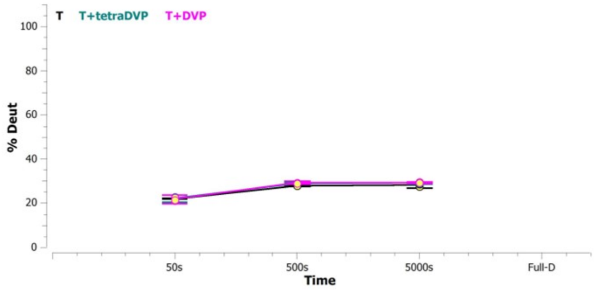

394-407: YKTTPPVLDSDGSF

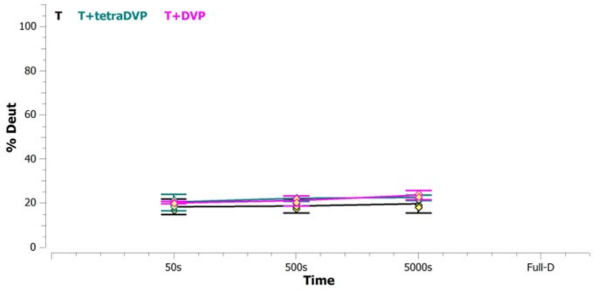

395-401: KTTPPVL

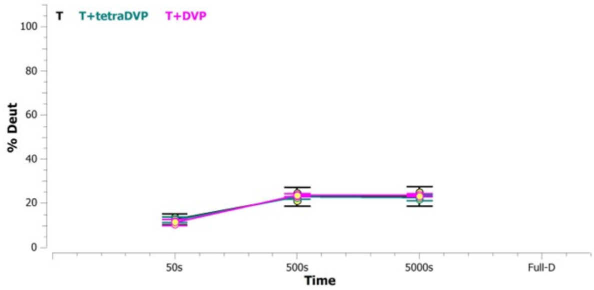

408-413: FLYSKL

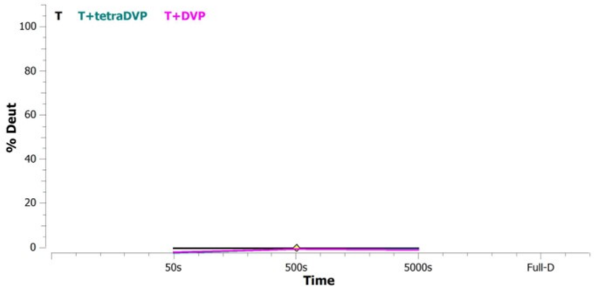

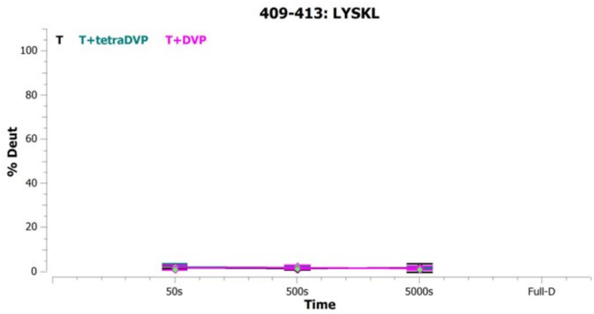
409-413: LYSKL
T
T+tetraDVP
T+DVP
% Deut
0
20
40
60
80
100
50s
500s
5000s
Full-D
Time

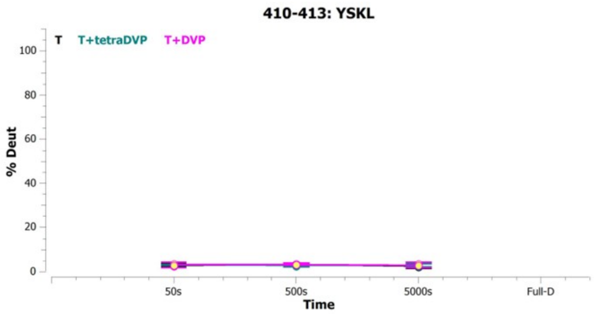
410-413: YSKL
T
T+tetraDVP
T+DVP
% Deut
0
20
40
60
80
100
50s
500s
5000s
Full-D
Time

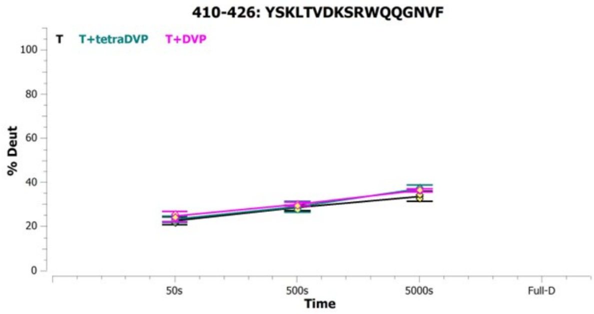
410-426: YSKLTVDKSRWQQGNVF
T
T+tetraDVP
T+DVP
% Deut
0
20
40
60
80
100
50s
500s
5000s
Full-D
Time

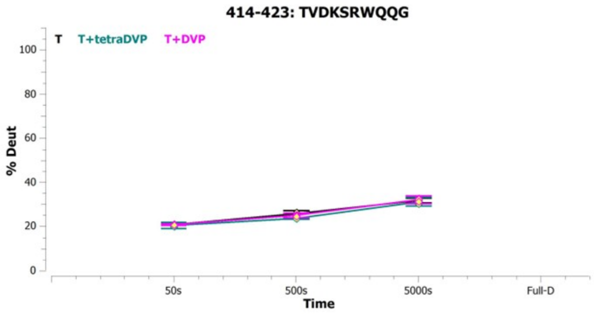
414-423: TVDKSRWQQG
T
T+tetraDVP
T+DVP
% Deut
0
20
40
60
80
100
50s
500s
5000s
Full-D
Time

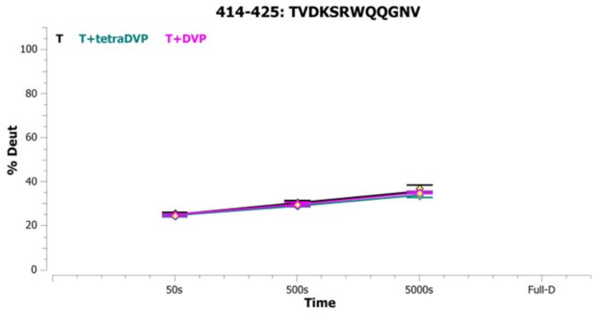
414-425: TVDKSRWQQGNV
T
T+tetraDVP
T+DVP
% Deut
0
20
40
60
80
100
50s
500s
5000s
Full-D
Time

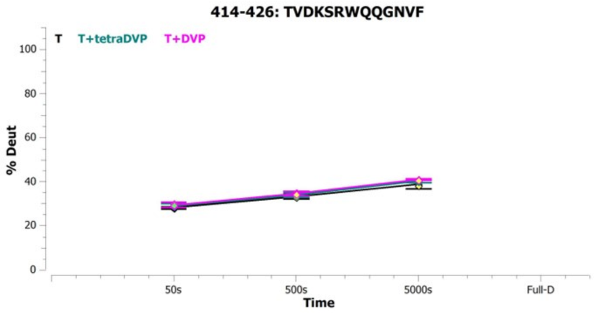
414-426: TVDKSRWQQGNVF
T
T+tetraDVP
T+DVP
% Deut
0
20
40
60
80
100
50s
500s
5000s
Full-D
Time

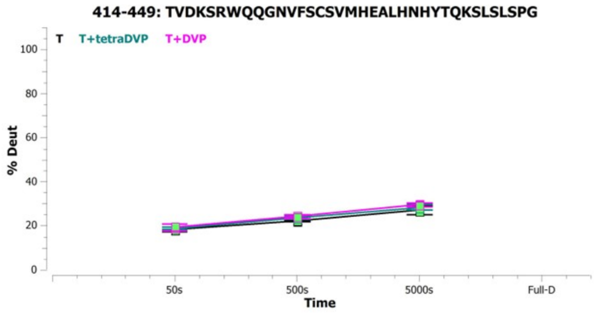
414-449: TVDKSRWQQGNVFSCSVMHEALHNHYTQKSLSLSPG
T
T+tetraDVP
T+DVP
% Deut
0
20
40
60
80
100
50s
500s
5000s
Full-D
Time

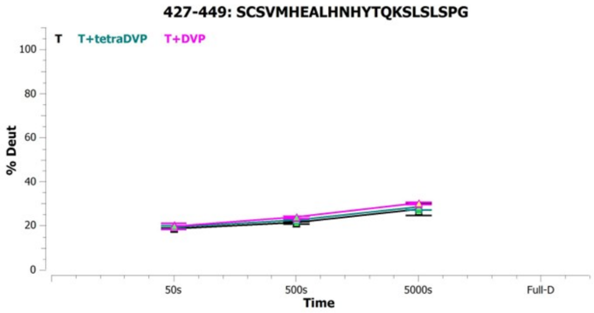
427-449: SCSVMHEALHNHYTQKSLSLSPG
T
T+tetraDVP
T+DVP
% Deut
0
20
40
60
80
100
50s
500s
5000s
Full-D
Time

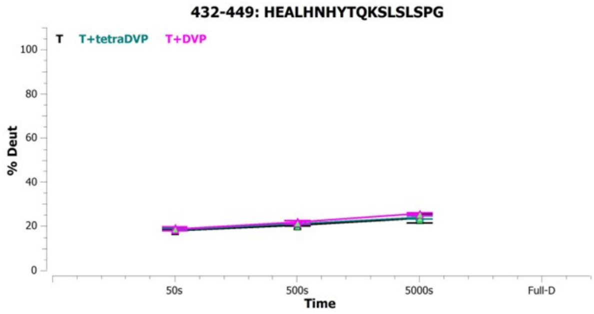
432-449: HEALHNHYTQKSLSLSPG
T
T+tetraDVP
T+DVP
% Deut
0
20
40
60
80
100
50s
500s
5000s
Full-D
Time

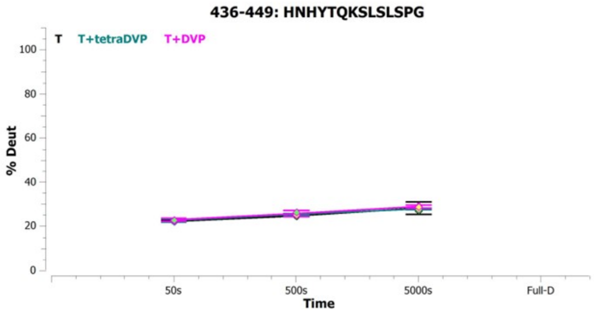
436-449: HNHYTQKSLSLSPG
T
T+tetraDVP
T+DVP
% Deut
0
20
40
60
80
100
50s
500s
5000s
Full-D
Time

References

1 J. D. Bargh, S. J. Walsh, A. Isidro-Llobet, S. Omarjee, J. S. Carroll and D. R. Spring, *Chem. Sci.*, 2020, **11**, 2375–2380.

2 M. Pittelkow, R. Lewinsky and J. B. Christensen, *Synthesis*, 2002, **15**, 2195–2202.

3 A. Makino, E. Hara, I. Hara, E. Ozeki and S. Kimura, *Langmuir*, 2014, **30**, 669–674.

4 L. N. Goswami, Q. Cai, L. Ma, S. S. Jalisatgi and M. F. Hawthorne, *Org. Biomol. Chem.*, 2015, **13**, 8912–8918.

5 C. G. Collins, J. M. Baumes and B. D. Smith, *Chem. Commun.*, 2011, **47**, 12352–12354.

6 B. G. Pasupuleti, K. Khongsti, B. Das and G. Bez, *Eur. J. Med. Chem.*, 2020, **186**, 111908.

7 J. M. Scholtz, G. R. Grimsley and C. N. Pace, *Methods Enzymol.*, 2009, **466**, 549–565.

8 S. E. Jackson and A. R. Fersht, *Biochemistry*, 1991, **30**, 10428–10435.

9 C. L. Dobson, P. W. A. Devine, J. J. Phillips, D. R. Higazi, C. Lloyd, B. Popovic, J. Arnold, A. Buchanan, A. Lewis, J. Goodman, C. F. Van Der Walle, P. Thornton, L. Vinall, D. Lowne, A. Aagaard, L. L. Olsson, A. R. Wollberg, F. Welsh, T. K. Karamanos, C. L. Pashley, M. G. Iadanza, N. A. Ranson, A. E. Ashcroft, A. D. Kippen, T. J. Vaughan, S. E. Radford and D. C. Lowe, *Sci. Rep.*, 2016, **6**, 1–14.
